# A biocatalytic platform for asymmetric alkylation of α-keto acids by mining and engineering of methyltransferases

**DOI:** 10.1038/s41467-023-40980-w

**Published:** 2023-09-14

**Authors:** Shuyun Ju, Kaylee P. Kuzelka, Rui Guo, Benjamin Krohn-Hansen, Jianping Wu, Satish K. Nair, Yang Yang

**Affiliations:** 1 Department of Chemistry and Biochemistry, University of California, Santa Barbara, CA USA; 2 Department of Biochemistry, Carl R. Woese Institute for Genomic Biology, University of Illinois at Urbana−Champaign, Urbana, IL USA; 3 Center for Biophysics and Computational Biology, University of Illinois at Urbana−Champaign, Urbana, IL USA; 4 Biomolecular Science and Engineering (BMSE) Program, University of California, Santa Barbara, CA USA

**Keywords:** Biocatalysis, Synthetic chemistry methodology, Biocatalysis

## Abstract

Catalytic asymmetric α-alkylation of carbonyl compounds represents a long-standing challenge in synthetic organic chemistry. Herein, we advance a dual biocatalytic platform for the efficient asymmetric alkylation of α-keto acids. First, guided by our recently obtained crystal structures, we develop SgvM^VAV^ as a general biocatalyst for the enantioselective methylation, ethylation, allylation and propargylation of a range of α-keto acids with total turnover numbers (TTNs) up to 4,600. Second, we mine a family of bacterial HMTs from *Pseudomonas* species sharing less than 50% sequence identities with known HMTs and evaluated their activities in SAM regeneration. Our best performing HMT from *P. aeruginosa*, *Pa*HMT, displays the highest SAM regeneration efficiencies (TTN up to 7,700) among HMTs characterized to date. Together, the synergistic use of SgvM^VAV^ and *Pa*HMT affords a fully biocatalytic protocol for asymmetric methylation featuring a record turnover efficiency, providing a solution to the notorious problem of asymmetric alkylation.

## Abstract (web summary)

Catalytic asymmetric a-alkylation of carbonyl compounds represents a longstanding challenge in synthetic organic chemistry but general methods for the catalytic symmetric alkylation of carbonyl compounds remain elusive. Here, the authors mine and engineer methyltransferases as synthetically useful biocatalysts for the catalytic asymmetric alkylation reactions of carbonyl compounds.

## Introduction

As catalysts capable of exerting exquisite control over the stereochemical outcome of chemical reactions under mild and benign conditions, enzymes hold the potential to revolutionize the practice of asymmetric synthesis. Over the past three decades, the advent of powerful protein engineering technologies^1–3^ has enabled the rapid development of customized enzymes with excellent activity and stereoselectivity toward non-native substrates, furnishing exciting biocatalytic solutions to synthetic problems^4^. However, compared to the powerful toolbox of abiotic chemistry discovered and optimized by synthetic chemists, the reaction type and substrate scope of biocatalysts still remain limited^5^. In particular, in contrast to the widespread application of enzymatic functional group manipulation, synthetically useful biocatalytic methods to form C–C bonds^6,7^—an essential process to forge the backbone of organic compounds—remains largely underdeveloped.

**Fig. 1:**
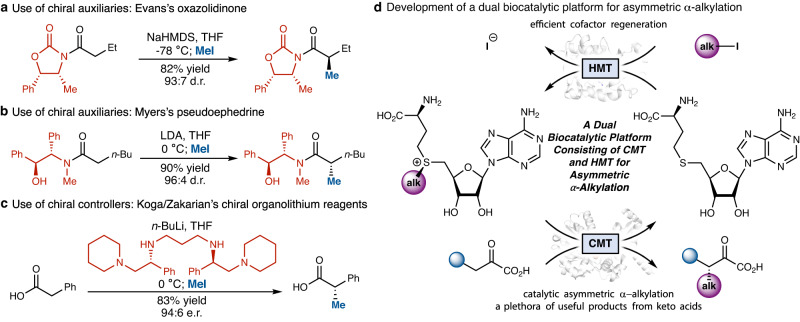
Asymmetric α-methylation and α-alkylation of carboxylic acid derivatives: state-of-the-art methods based on stoichiometric chiral auxiliaries and chiral controllers, and our dual biocatalytic platform for asymmetric α-alkylation. **a** Use of chiral auxiliaries: Evans’s oxazolidinone. **b** Use of chiral auxiliaries: Myers’s pseudoephedrine. **c** Use of chiral controllers: Koga/Zakarian’s chiral polydentate amine-supported organolithium reagents. **d** Development of a dual biocatalytic platform consisting of optimized CMT and HMT for asymmetric α-alkylation using alkyl halides as the alkyl donor. CMT carbon methyltransferase, HMT halogen methyltransferase.

Among the various C–C bond forming reactions, the α-alkylation of carbonyl compounds is a fundamentally important process taught in introductory organic chemistry courses^8,9^. These reactions lead to the formation of a stereogenic center adjacent to the synthetically versatile carbonyl group, and are widely utilized in the construction of complex molecular architectures, including bioactive natural products and clinically important therapeutics^10^. Despite the apparent simplicity and fundamental importance, general methods for the catalytic asymmetric alkylation of carbonyl compounds have eluded synthetic chemists for decades^9^. Previously developed and widely implemented approaches for the asymmetric alkylation of carboxylic acid derivatives typically hinge on the use of a stoichiometric amount of chiral auxiliaries, such as the Evans^8,11^ and the Myers auxiliaries^12–15^, or Koga^16–19^ and Zakarian’s^20–22^ chiral polydentate amines to achieve excellent stereocontrol (Fig. 1a–c). Furthermore, the enantioselective incorporation of the smallest alkyl group, namely the methyl group, into the α-position of carbonyls represents a disproportionately large hurdle facing synthetic chemists^23^. As the introduction of a methyl substituent into medicinal agents can dramatically enhance their bioactivity due to the “magic methyl effect”^24,25^, methods allowing for selective methylation are particularly valuable to medicinal chemists.

To address these challenges, we initiated a research program to mine and engineer methyltransferases as synthetically useful biocatalysts for the catalytic asymmetric α-methylation and other alkylation reactions of carbonyl compounds (Fig. 1d). Nature employs methylation as a central mechanism to regulate biology^26,27^. Thus, a large variety of methyl transfer enzymes has been evolved in the biological world. Our inspiration came from a largely underutilized enzyme family using *S*-adenosylmethionine (SAM), an organic cofactor bearing a highly electrophilic and stereogenic sulfonium ion, to catalyze selective methyl transfer^28–31^. We envisioned a fully biocatalytic platform consisting of an engineered SAM-dependent carbon methyltransferase (CMT)^28^ and a highly efficient halogen methyltransferase (HMT)^32–34^ to facilitate the asymmetric α-alkylation of carbonyl compounds using readily available alkyl electrophiles as the terminal alkyl donor. In this biocatalytic asymmetric alkylation, the CMT enzyme catalyzes the stereocontrolled delivery of the alkyl group using a sulfonium cofactor shuttle. The HMT enzyme then regenerates the sulfonium cofactor from *S*-adenosylhomocysteine (SAH) using alkyl (pseudo)halides, as recently elegantly demonstrated by Seebeck, Bornscheuer and Hammer^35–40^, and completes the catalytic cycle. Herein, we describe the implementation of this proposal through the mining, structural analysis, and protein engineering of carbon methyltransferases and halogen methyltransferases. First, through structure-guided enzyme engineering, we developed a general, selective, and highly efficient biocatalyst SgvM^VAV^ for asymmetric alkylation. Furthermore, through the mining of a family of bacterial HMTs from *Pseudomonas* species, we discovered one of the best SAM regeneration enzymes, *Pa*HMT, allowing record turnover efficiencies to be accomplished.

## Results and discussion

**Fig. 2:**
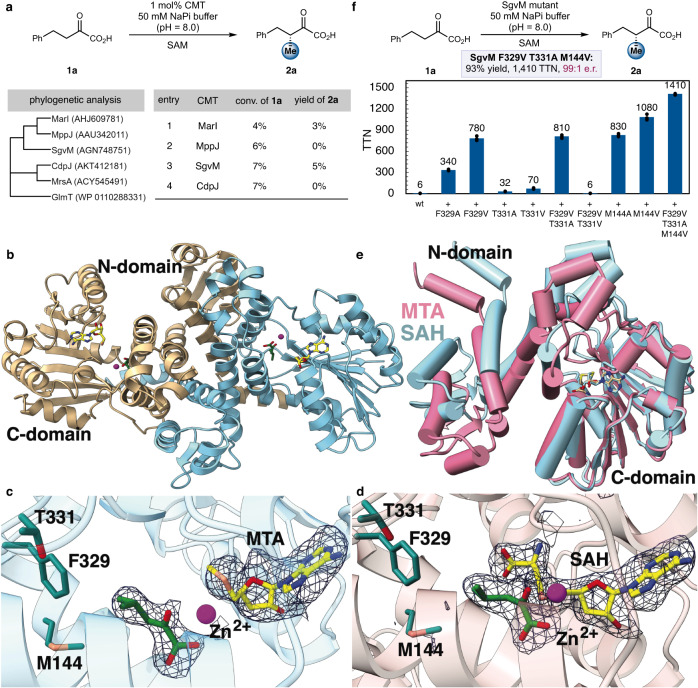
Protein structure and engineering of SgvM toward a general enantioselective methylation biocatalyst. **a** Phylogenetic analysis and evaluation of wt CMTs. Original organism: MarI: *Streptomyces* sp. B9173, MppJ: *S. hygroscopicus*, SgvM: *S. griseoviridis*, CdpJ: *Chondromyces crocatus*. **b** Overview of the structure of wt SgvM. **c** SgvM in complex with α-ketoleucine and methylthioadenosine (MTA). A simulated annealing omit map with ligand coordinates omitted is superimposed at 3σ above background in blue wire-mesh. **d** SgvM in complex with α-ketoleucine and *S*-adenosylhomocysteine (SAH). A simulated annealing omit map with ligand coordinates omitted is superimposed at 3σ above background in blue wire-mesh. **e** Superposition of the SAH- and MTA-bound structures of SgvM. **f** Structure-guided engineering of SgvM: development of the triple mutant SgvM F329V T331A M144V (SgvM^VAV^). Reactions were performed in triplicates and data are shown as mean ± SD (*n* = 3).

We focused our initial efforts on a family of SAM-dependent CMTs, whose native function is to enantioselectively introduce a methyl unit onto the α-position of keto acids (Figs. 1d and 2a). In particular, pioneering research from Müller and coworkers demonstrated the utility of wild-type SgvM^41^ in effecting asymmetric α-methylation and ethylation^42^. From a synthetic perspective, the α-keto acid moiety provides an excellent functional group handle, allowing convenient access to various valuable products bearing an α- and/or β-stereogenic center. Additionally, the α-keto acid substrates are easily accessible from the corresponding carboxylic acids using Bode’s methods^43–45^. Despite the synthetic potential of SAM-dependent CMTs from this class, previously characterized wild-type (wt) enzymes only accept native substrates or a narrow range of α-keto acids which are sterically and electronically similar to the native substrate^41,42,46–51^. Thus, we set out to develop general and synthetically useful biocatalysts for asymmetric alkylation through the mining and engineering of CMTs.

To commence our investigation, we performed phylogenetic analysis and assembled a library of CMTs, including MarI^46^, MppJ^47,48^, SgvM^41,42^, CdpJ^49^, MrsA^50^, and GlmT^51^ with distinct native ketoacid substrates (see Supplementary Fig. 2). While all these CMTs could be successfully expressed using *E. coli* as the microbial host, MppJ and SgvM were produced at higher expression levels ( > 100 mg/L TB culture). To develop generally applicable alkylation biocatalysts, we assayed the activity of these CMTs using 2-oxo-4-phenylbutanoic acid (**1a**), a non-native α-keto acid bearing a large pedant phenyl substituent, as the model substrate (Fig. 2a). In our initial studies, we used methionine adenosyltransferase from *E. coli* (*Ec*MAT) or *Bacillus subtilis* (*Bs*MAT) to generate SAM from ATP and 5′-methylthioadenosine/*S*-adenosylhomocysteine (MTA/SAH) nucleosidase from *E. coli* (*Ec*MTAN) to degrade SAH. Among all the wild-type CMTs we examined (Supplementary Table 1), MarI (Fig. 2a, entry 1) and SgvM (entry 3) furnished measurable activities, with SgvM providing the highest yield of **2a** (5%). SgvM is found in the biosynthetic gene cluster of the antibiotic viridogrisein and catalyzes methyl transfer to the C3 (i.e., β) position of α-ketoleucine (4-methyl-2-oxovalerate)^41^.

In light of its highest initial activity and substantially better expression level (ca. 150 mg/L TB culture) than MarI, we focused our efforts on the engineering of SgvM to further enhance its efficiency to catalyze a wider range of enantioselective biocatalytic methylation processes. To facilitate this protein engineering, we first determined the structure of wild-type SgvM by heavy atom phasing and solved several co-crystal structures with bound cofactors and substrates (Fig. 2b–e). The structure of SgvM captured in a non-productive complex with 5’-methylthioadenosine (MTA), likely derived from SAM hydrolysis, and α-ketoleucine has been reported in a published thesis but the atomic coordinates have not been deposited in any data bank^52^. Hence, we first determined the 2.13 Å resolution structure of wt SgvM in complex with MTA and α-ketoleucine. The overall structure consists of a two-domain fold that assembles into homodimer in both the solution and the crystal (Fig. 2b). The *N*-terminal domain (I6 through H147) consists of helices that mediate dimerization, while the C-terminal domain (F149 through H338) consists of a Rossman fold.

A single metal ion is located at the base of the C-terminal domain and is ligated by H244 and H296 (Fig. 2c). Anomalous difference Fourier maps calculated from data collected at the zinc absorption edge, and the tetrahedral coordination are consistent with a bound zinc ion. The zinc is likely integral to the enzyme as no exogenous metals were added during purification or crystallization. When modeled as zinc ion at full occupancy, the thermal (B) factors of the metal are consistent with those of the rest of the polypeptide. Presumably, the metal ion acts as a Lewis acid to trigger the soft enolization of the ketoacid substrate and facilitates electrophilic methylation. Density corresponding to the α-ketoleucine substrate can be observed directly bound to the metal. However, SAM could not be observed in the structure but rather density for MTA, likely derived from SAM hydrolysis, is seen in the near vicinity of the metal. The distance from the MTA sulfur atom to C3 position of α-ketoleucine is ca. 7 Å, suggesting that this represents a nonproductive complex. This non-productive complex resembles that observed in the previously reported structure of SgvM.

**Fig. 3:**
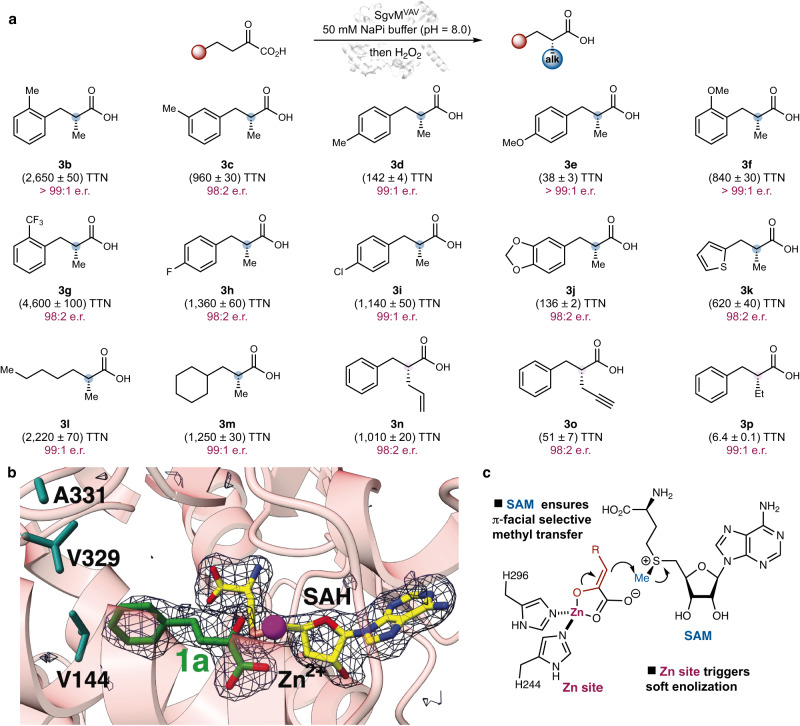
Engineered triple mutant alkyl transfer enzyme SgvM^VAV^: substrate scope and structural insights. **a** SgvM^VAV^-catalyzed enantioselective methylation and various other alkylations of α−keto acids. **b** Crystal structure of triple mutant SgvM^VAV^ in complex with model substrate 1a. A simulated annealing omit map with ligand coordinates omitted is superimposed at 3σ above background in blue wire-mesh. **c** Enantioinduction mechanism with SgvM^VAV^.

We next determined the structure of wt SgvM in complex with SAH and α-ketoleucine (2.2 Å resolution, Fig. 2d). A superposition with the MTA bound structure shows that the *N*-terminal domain has undergone a marked shift resulting in an overall closed conformation (Fig. 2e). The cofactor now resides in proximity to the substrate (3.7 Å distance between the sulfur of SAH and C3 of α-ketoleucine). The (*Re*)-face of the zinc enolate is shielded by the protein scaffold, while the (*Si*)-face is exposed to the methylating cofactor, thereby allowing methyl transfer to occur with excellent π-facial selectivity (Fig. 2d and Fig. 3c).

With these enzyme structures, we embarked on structure-guided protein engineering of SgvM (Fig. 2f). In wt SgvM-SAH-α-ketoleucine complex, the isopropyl chain of the substrate is in close contact with two residues, including S141 and F329. While S141 is located in an α-helix, the bulky aromatic amino acid residue F329 resides in a flexible loop. Upon further examining the crystal structure of SgvM, we found that the F329-containing flexible loop encapsulates the substrate binding pocket. We thus posited that flexible loop engineering^53,54^ can dramatically broaden the substrate scope of SgvM and furnish a general biocatalyst for enantioselective methylation. Additionally, multiple sequence alignment showed that this aromatic residue of SgvM (F329) is not conserved in the flexible loop of CMTs of this family (Supplementary Fig. 3), further supporting our envisioned flexible loop engineering approach.

Indeed, introducing a single F329A mutation into SgvM led to a 57-fold increase in total turnover number (TTN) for the biocatalytic transformation of **1a**, confirming the critical role of residue 329 in modulating protein-substrate interaction (Fig. 2f, wt SgvM: TTN = 6, SgvM F329A: TTN = 340). We subsequently replaced F329 with residues possessing varying side chain sterics (Fig. 2f, Supplementary Fig. 6), including glycine (G), leucine (L), isoleucine (I) and valine (V), leading to the identification of SgvM F329V with the highest activity (TTN = 780). Encouraged by these results, we next evaluated the role of T331 in the same flexible loop (Supplementary Fig. 5). It was found that T331A and T331V had similar activating effects on the conversion of **1a**, giving rise to TTNs of 32 and 70, respectively. Although the recombination of F329V and T331V led to inferior results, double mutant SgvM F329V T331A displayed a higher catalytic activity relative to the corresponding single mutants (TTN = 810). Further active site engineering showed that a single M144V mutation significantly improved enzyme activity (TTN = 1080). M144 resides in an α-helix proximal to the phenyl group of **1a** (Supplementary Fig. 5). Ultimately, triple mutant SgvM F329V T331A M144V (SgvM^VAV^) exhibited the highest activity, furnishing excellent yield and enantiomeric ratio (e.r.) of **1a** (TTN = 1,410, 93% yield and 99:1 e.r.). Compared to wt SgvM, SgvM^VAV^ provided a 235-fold improvement in TTN, demonstrating its substantially enhanced catalytic efficiency.

With this engineered SgvM^VAV^ variant, we examined the substrate scope of this enantioselective α-methylation process (Fig. 3a). In this study, alkylated α-ketoacids (**2a**−**2p**) were converted into the corresponding carboxylic acids (**3a**−**3p**) (*vide infra*). It was found that keto acids bearing an *ortho*- and a *meta*-substituent (**1b** and **1c**) were excellent substrates, affording methylated products **3b** and **3c** with good to excellent TTN and e.r. For *para*-substituted substrates **1d** and **1e**, SgvM^VAV^ displayed reduced activities but high levels of enantioselectivity (**3d** and **3e**). Electron-donating methoxy (**3f**) and electron-withdrawing trifluoromethyl (**3g**) groups of the aromatic ring were found to be compatible. Other functional groups, including a fluorine (**3h**) and a chlorine (**3i**), were also tolerated. Additionally, heterocycles such as a benzodioxole (**3j**) and a thiophene (**3k**) were successfully converted with excellent enantiocontrol. Finally, other aliphatic substrates underwent smooth enzymatic transformation to furnish methylated products (**3l** and **3m**) in excellent enantioselectivity. To our knowledge, the total turnover numbers of our engineered SgvM triple mutant described herein (up to 4,600 TTN, **3g**) represented a record high for SAM-dependent carbon methyltransferases.

Moreover, we found that without additional protein engineering, SgvM^VAV^ readily allowed asymmetric α-allylation to occur with excellent activity and enantiocontrol using the *S*-allyl analog of SAM (**3n**)^28^. In light of the synthetic versatility of the allyl moiety and the central role of enantioselective allylation in asymmetric synthesis^55,56^, this result represented a synthetically useful advance. Furthermore, enantioselective α-propargylation and ethylation were accomplished with excellent enantioselectivity, although with lower activity. Together, these results demonstrated the excellent potential of SgvM^VAV^ variant for diverse asymmetric α-alkylative functionalization processes. Further engineering of alkyl transfer biocatalysts derived from SgvM^VAV^ with enhanced catalytic activities is currently underway in our laboratory.

In order to provide further insights into the broader substrate scope and enhanced total turnover number of SgvM^VAV^, we determined its crystal structure in complex with SAH and **1a** (2.04 Å resolution, Fig. 3b). Protein crystallography showed that although the three amino acid substitutions led to limited alterations in the overall structure, they resulted in a substantially widening of the binding pocket to accommodate larger substrates (Supplementary Fig. 7). Meanwhile, interactions between the protein, the metal ion and the bound SAH cofactor are sufficient to accommodate these dramatic changes in the active site contours with minimal perturbations to the overall scaffold. Taken together, the expanded substrate binding pocket and the retained overall enzyme scaffold allowed for a broad range of substrates to be effectively transformed, leading to a generally useful enantioselective alkylation biocatalyst.

**Fig. 4:**
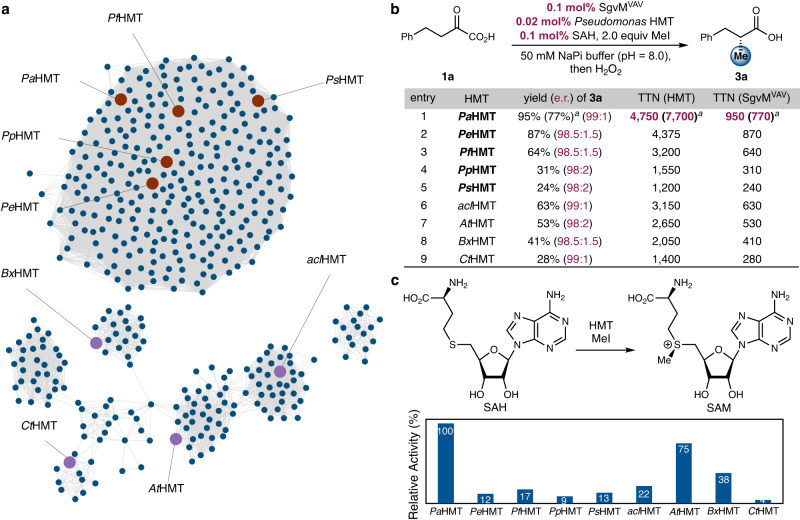
Development of a fully biocatalytic platform for asymmetric α-methylation with a record turnover efficiency. **a** Mining of *Pseudomonas* HMTs: sequence similarity network (SSN) of selected putative HMTs. **b** Dual biocatalytic enantioselective methylation using SgvM^VAV^ and HMTs mined from *Pseudomonas* species. *Pa*HMT: *P. aeruginosa* HMT, *Pe*HMT: *P. entomophila* HMT, *Pf*HMT: *P. fluorescens* HMT, *Pp*HMT: *P. putida* HMT, *Ps*HMT: *P. syringae* HMT. ^a^0.01 mol% *Pa*HMT was used. **c** SAM regeneration efficiency with HMTs from *Pseudomonas* sp.

Next, we sought to couple this engineered CMT with a halogen methyl transferase (HMT)-based cofactor regeneration system to develop a fully biocatalytic asymmetric methylation system using methyl iodide as the methyl donor (Fig. 4). Based on sequence similarity network (SSN) analysis, we focused on the mining of previously unexplored putative halogen methyl transferases from *Pseudomonas* species (Fig. 4a). Annotated as thiopurine *S*-methyltransferase (TPMT), these SAM-dependent methyltransferases display less than 50% sequence identities with other previously mined and biochemically characterized HMTs and are thus distinct SAM-dependent enzymes (Fig. 4a, see Supplementary Fig. 4 for a phylogenetic tree). We cloned five putative HMTs from this subfamily, and expressed them in our engineered *E. coli* BL21(DE3)Δ*mtn* strain (Supplementary Fig. 1). Compared to previously investigated bacterial, fungal and plant HMTs, these *Pseudomonas* HMTs showed higher production levels when heterologously expressed in *E. coli*, thus indicating their potential as practical biocatalysts in biotechnology.

Furthermore, it was found that our *Pseudomonas* HMTs exhibited promising activities toward SAM regeneration from SAH and methyl iodide (Fig. 4b, c). In particular, HMT from *P. aeruginosa* (Fig. 4b, entry 1) showed the highest SAM regeneration efficiency in the dual enzyme system among mined HMTs of this family (entries 1−5) as well as previously studied bacterial, fungal and plant HMTs (entries 6−9)^35,37,39,40^. Notably, in the presence of 0.1 mol% SgvM^VAV^, 0.02 mol% *Pa*HMT, 0.1 mol% SAH and 2 equiv of methyl iodide, enantioenriched **2a** formed in 95% yield and 99:1 e.r. (entry 1). This corresponds to a TTN of 4,750 for *Pa*HMT and a TTN of 950 for SgvM^VAV^. By further lowering the *Pa*HMT loading to 0.01 mol%, its TTN could be further increased to 7,700 (entry 1). To our knowledge, these TTNs represented the highest turnover efficiencies for dual biocatalytic methylation processes of this type. Additionally, with 0.1 mol% engineered SgvM^VAV^ and 0.02 mol% *Pa*HMT, the total turnover number of the SAH cofactor approached 1,000, which is of the same order of magnitude for practical NAD(P)H-regeneration systems^57^. Furthermore, *Pa*HMT also showed the highest relative activity in SAM regeneration from SAH and methyl iodide among HMTs studied herein (Fig. 4c). Taken together, the excellent catalytic efficiency and enantioselectivity of our optimized dual biocatalytic system consisting of SgvM^VAV^ and *Pa*HMT showcased the synthetic potential of SAM-dependent methyl transfer enzymes for asymmetric C−C bond formation.

**Fig. 5:**
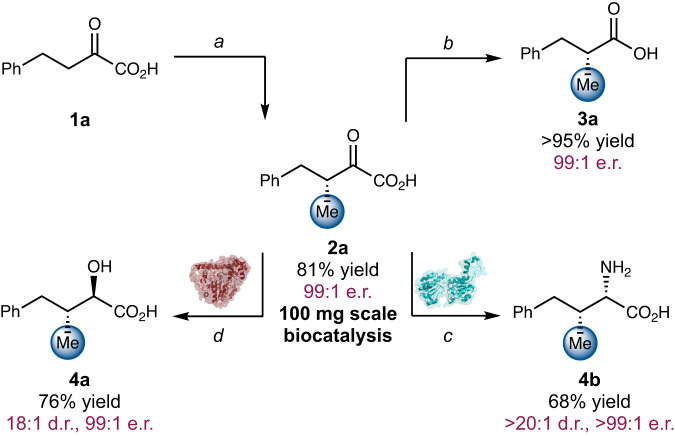
Preparative-scale biotransformations and biocatalytic cascade reactions of α-keto acids. Conditions: **a** SgvM^VAV^, *Pa*HMT, MeI. **b** H_2_O_2_, then catalase. **c** *Km*AT, L-glutamate, NaPi buffer (pH = 8.0), 30 °C, 24 h. **d** YiaE, NADP^+^, NaPi buffer (pH = 7.5), Codex cofactor recycling mix, 30 °C, 24 h.

To further demonstrate the synthetic utility of this fully biocatalytic asymmetric alkylation system, we carried out preparative scale biocatalytic synthesis and cascades (Fig. 5). Enantioenriched keto acid **2a** was prepared on a 100 mg scale with 91% yield and 99:1 e.r., showcasing the synthetic utility of this enzymatic process. On the basis of this result, a range of derivatization reactions was successfully carried out. First, oxidative decarboxylation of α-keto acid **2a** using hydrogen peroxide furnished carboxylic acid **3a** in quantitative yield and 99:1 e.r.. Moreover, taking advantage of the synthetically versatile ketoacid moiety, biocatalytic cascades were successfully carried out to afford stereochemical dyads with excellent diastereo- and enantioselectivities (**4a** and **4b**). By coupling the SgvM^VAV^-*Pa*HMT dual enzyme system with a ketoacid reductase^58^, α-hydroxy-β-methyl acid **4a** with adjacent stereogenic centers formed in 76% yield, 19:1 d.r. and >99:1 e.r.. Additionally, cascade enantioselective methylation/diastereoselective transamination^59^ furnished α-amino-β-methyl acid **4b** with vicinal stereocenters in 68% yield, >20:1 d.r. and >99:1 e.r. We note that using chemical methods, low levels of diastereoselectivity (d.r. = ca. 1:1) were observed for hydroxyl acid and amino acid synthesis, thus highlighting the power of biocatalytic cascades to access well-defined stereochemical dyads. Together, these chemical and biocatalytic transformations demonstrated the value of the α-keto acid functional group handle to access a wide range of synthetically and medicinally valuable scaffolds.

In conclusion, we developed a dual biocatalytic platform composed of our engineered SAM-dependent CMT and mined HMT for the efficient catalytic asymmetric alkylation of α-keto acids. First, SgvM^VAV^ was engineered as a general biocatalyst for the asymmetric α-alkylation of α-keto acids, displaying record total turnover numbers (TTN up to 4,600) and excellent enantioselectivities across a diverse range of substrates. In this effort, the crystal structures of both the wild-type and engineered biocatalysts were solved, revealing the presence of two essential catalytic components, including a Lewis acidic Zn site to trigger substrate enolization and an adjacent SAM to ensure stereoselective methyl transfer. Second, we mined, cloned, and assessed a family of HMTs from *Pseudomonas* species that share less than 50% sequence identity to previously evaluated HMTs. In particular, HMT from *P. aeruginosa* exhibited the highest catalytic efficiency in the dual biocatalytic asymmetric methylation, allowing a record turnover efficiency (TTN of HMT up to 7,700, TTN of SAH up to 1,000) to be accomplished, thereby demonstrating the synthetic potential of this fully biocatalytic platform. Furthermore, based on this dual enzyme platform, a range of valuable enantioenriched scaffolds, including α-alkyl carboxylic acids, β-alkyl,α-hydroxy acids and β-alkyl,α-amino acids, could be conveniently prepared through biocatalytic cascades. Collectively, these enzyme mining and engineering efforts set the stage for advancing a general biocatalytic platform for catalytic asymmetric α-alkylation using easily available alkyl (pseudo)halides, providing a solution to this long-standing challenging facing synthetic organic chemists.

## Methods

### Analytical scale biocatalytic asymmetric methylation of α-ketoacids using SgvM^VAV^

To a 2 mL vial were added NaPi buffer (50 mM, pH 8.0), SAM (80 μL of 100 mM stock solution in 50 mM NaPi buffer, pH 8.0), SgvM^VAV^, *Ec*MTAN, and substrate **1** (250 μL of 10 mM stock solution in 50 mM NaPi buffer, pH 8.0) in succession. Final reaction volume was 1 mL; final concentrations were 2.5 mM **1**, 8.0 mM SAM, 0.4 – 1.6 μM SgvM^VAV^, and 2.5 μM *Ec*MTAN. The vials were sealed and shaken at 30 °C and 250 rpm for 10 – 14 h and analyzed by HPLC as detailed in the Supplementary Information.

### Analytical scale biocatalytic asymmetric methylation with methyl iodide as the terminal methyl donor using SgvM^VAV^ and HMT

To a 2 mL vial were added NaPi buffer (50 mM, pH 8.0), SAH (2.5 μL of 1 mM stock solution in DMSO), methyl iodide (25 μL of 200 mM stock solution in DMSO), SgvM^VAV^, HMT, and substrate **1a** (250 μL of 10 mM stock solution in 50 mM NaPi buffer, pH 8.0) in succession. Final reaction volume was 1 mL; final concentrations were 2.5 mM **1a**, 2.5 μM SAH, 5.0 mM methyl iodide, 0.25 μM SgvM^VAV^, 0.25–2.5 μM HMT. The vials were sealed and shaken at 30 °C and 250 rpm for 6–24 h. The reactions were then analyzed by HPLC as detailed in the Supplementary Information.

### Preparative scale biocatalytic asymmetric methylation with methyl iodide as the terminal methyl donor using SgvM^VAV^ and *Pa*HMT

To a 100 mL flask were added NaPi buffer (50 mM, pH 8.0), SAH (60 μL of 10 mM stock solution in DMSO), methyl iodide (3 mL of 400 mM stock solution in DMSO), SgvM^VAV^, *Pa*HMT, and substrate **1a** (15 mL of 40 mM stock solution in NaPi buffer) in succession. Final reaction volume was 30 mL; final concentrations were 20 mM **1a**, 20 μM SAH, 40 mM methyl iodide, 20 μM SgvM^VAV^, 20 μM *Pa*HMT. The reaction flask was sealed and shaken at 30 °C and 250 rpm for 14 h.

Upon the completion of the reaction, the reaction mixture was then treated with H_2_O_2_ (30% w/w) and allowed to stir at room temperature for 2 h. Excess H_2_O_2_ was decomposed with catalase. The resulting mixture was concentrated *in vacuo*, and the pH of the aqueous layer was adjusted to 3–4 using 1.0 M HCl (aq.). This mixture was then shaken vigorously and centrifuged (12,000 × g, 20 min).

The supernatant containing the product was filtered and loaded onto a Biotage Sfär C18 cartridge (12 g) that had been equilibrated to 10% MeOH/H_2_O with 0.1% formic acid. The column was flushed with 2 column volumes (CV) of 10% MeOH/H_2_O (0.1% formic acid). Then, the product was eluted with a gradient from 10% to 100% methanol with 0.1% formic acid over 8 CV. Product-containing fractions were combined and concentrated in vacuo. The residual aqueous solution was lyophilized to afford the analytically pure product as a white powder in 81% yield (99:1 e.r.).

### Reporting summary

Further information on research design is available in the Nature Portfolio Reporting Summary linked to this article.

### Supplementary information

Supplementary Information

Peer Review File

Reporting Summary

## Data Availability

All data are available in the main text and the Supplementary Information. Plasmids encoding carbon and halogen methyltransferases reported in this study are available for research purposes from Y.Y. under a material transfer agreement (MTA) with the University of California Santa Barbara.
