# Supplementary material for: A biocatalytic platform for asymmetric alkylation of α-keto acids by mining and engineering of methyltransferases
Source: Nat Commun. 2023 Sep 14;14:5704. doi: 10.1038/s41467-023-40980-w (PMC10502145; doi:10.1038/s41467-023-40980-w)
Supplement: Supplementary file 1 — Supplementary Information [file 41467_2023_40980_MOESM1_ESM.pdf]

Supplementary Information for

A biocatalytic platform for asymmetric alkylation of α-keto acids by mining and engineering of methyltransferases

Shuyun Ju,[†] Kaylee P. Kuzelka,[†] Rui Guo, Benjamin Krohn-Hansen, Jianping Wu, Satish K. Nair[*] and Yang Yang[*]

[*]E-mail: snair@illinois.edu; yang@chem.ucsb.edu

Table of Contents

I. General methods 2

II. Evaluation of enzymes for biocatalytic asymmetric alkylation 10

III. Evaluation of SgvM variants in the biocatalytic asymmetric methylation: complete data..... 15

IV. Kinetic studies 18

V. SDS-PAGE analysis of purified enzymes used in the current study 21

VI. Nucleotide and amino acid sequences 21

VII. Synthesis and characterization of substrates 36

VIII. Characterization data for asymmetric alkylation products 43

IX. Procedure for derivatization reactions and characterization data. 50

X. HPLC/GC calibration curves of asymmetric alkylation products 57

XI. Chiral HPLC and GC traces 66

XII. Protein crystallization and structural determination 83

XIII. ^{1}H, ^{19}F and ^{13}C NMR spectra of compounds 87

XIV. References 124

I. General methods

General. Unless otherwise noted, all chemicals and reagents were obtained from commercial suppliers (Sigma-Aldrich, VWR, Alfa Aesar, Combi-Blocks) and used without further purification. *S*-Adenosyl-L-methionine disulfate tosylate was obtained from Jincheng Pharmaceutical Group Co., Ltd. Silica gel chromatography was carried out using AMD Silica Gel 60, 230-400 mesh. ^{1}H, and ^{13}C NMR spectra were recorded on a Bruker 400 or 500 MHz instrument and are referenced to residual protio solvent signals. ^{19}F NMR spectra (where applicable) were recorded on a Bruker 400 or 500 MHz (^{1}H decoupled) and are referenced to $CFCl_3$ as the external standard. Data for ^{1}H NMR are reported as follows: chemical shift (δ ppm), multiplicity (s = singlet, d = doublet, t = triplet, q = quartet, p = pentet, sext = sextet, m = multiplet, dd = doublet of doublets, dt = doublet of triplets, ddd = doublet of doublet of doublets), coupling constant (Hz), integration. Sonication on a small scale was performed using a BioLogics ultrasonic homogenizer (model 150VT) equipped with a stepped microtip. Sonication on a large scale was performed using a Branson Digital Sonifier 450 Ultrasonic Processor. All IR spectra were taken on a Thermo Scientific Nicolet iS5 spectrometer (iD5 ATR, diamond). High-resolution mass spectrometry data were obtained at the University of California Santa Barbara Mass Spectral Facility. High-resolution accurate mass ESI data was acquired using a Waters Micromass LCT Premier time-of-flight mass spectrometer. Masses of positively charged ions were calibrated using methanol solutions of polyethylene glycol or polyethylene glycol monomethyl ether as an internal standard. Masses of negatively charged ions were calibrated using aqueous sodium formate or sodium/cesium iodide as an internal standard as appropriate. All samples were dissolved in methanol and were directly infused unless otherwise noted. Samples were analyzed by liquid chromatography mass spectrometry (LCMS-ESI) or fast atom bombardment (FAB) mass spectrometry. LC-MS samples were run on a Kromasil 100-5-C18 column (4.6 × 50 mm, 5 µm) with a gradient of water (0.1% formic acid) and acetonitrile (0.1% formic acid). Synthetic reactions were monitored by thin layer chromatography (TLC, Merck 60 gel plates) using a UV-lamp or an appropriate TLC stain for visualization. Na_2HPO_4-NaH_2PO_4 buffer (abbreviated as NaPi buffer; 50 mM, pH 8.0) and Tris-HCl buffer (50 mM, pH 8.0) were used as buffering systems for purified enzymes, unless otherwise specified.

E. coli cells were grown using Luria-Bertani medium (LB) or Terrific Broth (TB). pET-28a(+) was used as the cloning and expression vector for all enzymes described in this study. Primer sequences are available upon request. T5 exonuclease, Phusion polymerase, and ligase were purchased from New England Biolabs (NEB, Ipswich, MA).

Chromatography. Analytical reverse-phase high-performance liquid chromatography (HPLC) was carried out using a YMC-Pack Pro C18 column (4.6 × 250 mm, 5 μm) with NaPi buffer (50 mM, pH 7.5) and MeCN as the mobile phase. Analytical chiral HPLC was conducted using an Agilent or Shimadzu i-series (66 MPa) instrument with hexanes and isopropanol as the mobile phase. Enantiomers were separated using one of the following chiral columns: CHIRALCEL® OJ-H (4.6 × 250 mm, 5 μm), IG (4.6 × 250 mm, 5 μm), and AD-H (4.6 × 250 mm, 5 μm). Chiral GC analysis was performed using a Shimadzu GC-2030 GC system equipped with an FID detector and an Agilent CycloSil-B column (30 m × 0.32 mm, 0.25 μm film). Gas chromatography-mass spectrometry (GC-MS) analyses were carried out using a Shimadzu GCMS-QP2020NX system with a GC-2030 front end and a J&W HP-5ms column (30 m × 0.25 mm, 0.25 μm film).

Construction of the MTA/SAH nucleosidase knockout strain *E. coli* BL21(DE3) Δ*mtn* by CRISPR-Cas9. The endogenous 5′-methylthioadenosine/*S*-adenosyl homocysteine (MTA/SAH) nucleosidase (GenBank ID: WP_000689844.1 [https://www.ncbi.nlm.nih.gov/protein/WP_000689844.1]) from *E. coli* BL21(DE3) transforms *S*-adenosyl homocysteine (SAH) into adenine and *S*-ribosyl-L-homocysteine, thereby complicating the biocatalytic enantioselective alkyl transfer processes studied in this work[1]. Thus, an MTA/SAH nucleosidase knockout strain, *E. coli* BL21(DE3) Δ*mtn*, was constructed. The corresponding gene *mtn* in *E. coli* BL21(DE3) was knocked out using pEcCas/pEcgRNA system described by Yang et al[2]. The N20 sequence in pEcgRNA was replaced by the predesigned N20 sequence for the target gene *mtn* via PCR with primers N20-mtn-Fwd/Rev. Overlap extension PCR with primers mtn-UP-Fwd/Rev and mtn-Down-Fwd/Rev (listed in Supplementary Table 1) was performed to construct the donor DNA. With new pEcgRNA and donor DNA in hand, additional genetic manipulations were completed according to Yang's method. The identity of this engineered *E. coli* BL21(DE3) Δ*mtn* strain was validated by PCR with primers validation-Fwd/Rev and DNA sequencing.

Supplementary Table 1. Primers used in this study

Primer name	Primers sequence (5' to 3')
N20-mtn-Fwd	AGTGGGCGATATCGTTGTCTGTTTTAGAGCTAGAAATAGC
N20-mtn-Rev	AGACAACGATATCGCCCACTACTAGTATTATACCTAGGAC
mtn-UP-Fwd	GAAACCAGTCATTTATCGCTGC
mtn-UP-Rev	CTGAACAGTGACTTAGCCATAGATTTACTCGCGATAAGCC
mtn-Down-Fwd	GGCTTATCGCGAGTAAATCTATGGCTAAGTCACTGTTCAG
mtn-Down-Rev	GCGGATTAATGCCGAATTGCAG
validation-Fwd	ATCGTCAGTCAGAGGCTGGCTTTC
validation-Rev	GAACCCGGCTGTCTTTAAAGATG

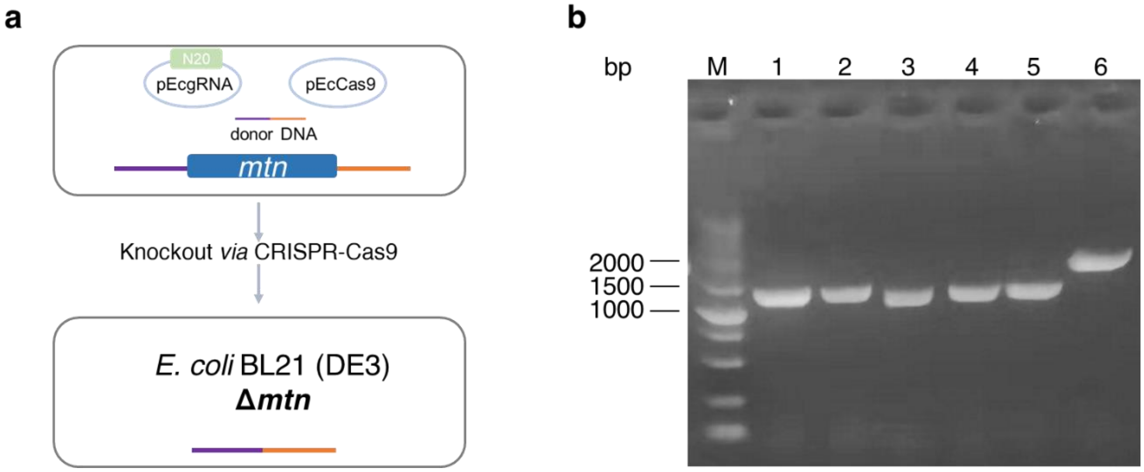

Supplementary Figure 1. Construction of *E. coli* BL21(DE3) Δ*mtn*. a Diagram of CRISPR-Cas9 mediated gene knockout (KO) of *mtn* (699 bp). **b** Analysis of PCR fragments to confirm the gene *mtn* knockout strain *E. coli* BL21(DE3) Δ*mtn*. PCR was performed with primers validation-Fwd/Rev (Supplementary Table 1). Lane M: DNA ladder marker; Lane 1-5: PCR product (theoretical size: 1125 bp) amplified with *E. coli* BL21(DE3) Δ*mtn* genomic DNA as template; Lane 6: PCR product (theoretical size: 1816 bp) amplified with *E. coli* BL21(DE3) genomic DNA as template. This result suggested that gene *mtn* was successfully knocked out.

Cloning. The genes encoding *C*-methyltransferases (CMTs, listed in Supplementary Table 2), halide methyltransferases (*Ct*HMT, *Bx*HMT, *At*HMT, *acl*-MT, listed in Supplementary Table 3),

thiopurine *S*-methyltransferase from *Pseudomonas syringae* pv. pisi (*Ps*HMT, listed in Supplementary Table 3), and aromatic amino acid transaminase from *Klebsiella michiganensis* (*Km*AT, GenBank ID: WP_194247410.1 [https://www.ncbi.nlm.nih.gov/protein/WP_194247410.1/]) were codon-optimized, chemically synthesized by GeneralBio, and inserted into pET-28a(+). The genes encoding 5'-methylthioadenosine/*S*-adenosylhomocysteine nucleosidase from *E. coli* (*Ec*MTAN, GenBank ID: WP_000689844.1 [https://www.ncbi.nlm.nih.gov/protein/WP_000689844.1]), glyoxylate/hydroxypyruvate reductase from *E. coli* (YiaE, GenBank ID: WP_000805027.1 [https://www.ncbi.nlm.nih.gov/protein/446727714]), *S*-adenosylmethionine synthetase from *Bacillus subtilis* 168 (*Bs*MAT, GenBank ID: WP_003229102.1 [https://www.ncbi.nlm.nih.gov/protein/WP_003229102.1]), and thiopurine *S*-methyltransferases from *P. aeruginosa* PAO1, *P. entomophila*, *P. fluorescens* Pf0-1, *P. putida* KT2440, (*Pa*HMT, *Pe*HMT, *Pf*HMT, *Pp*HMT, listed in Supplementary Table 3) were amplified from the corresponding genomic DNA and ligated into the pET-28a(+). *E. coli* BL21 (DE3) or BL21(DE3) Δ*mtn* was subsequently transformed with recombinant plasmids using the heat shock method.

Site-directed mutagenesis. Plasmids containing the SgvM gene were extracted using the Plasmid Miniprep Kit (Zymo Research, USA). The PCR products were digested with DpnI, gel purified, and ligated using Gibson Mix prepared from 5X isothermal (ISO) reaction buffer (25% PEG-8000, 500 mM Tris-HCl pH 7.5, 50 mM $MgCl_2$, 50 mM DTT, 1 mM each of the dNTPs, and 5 mM NAD^+), T5 exonuclease, Phusion DNA polymerase, and *Taq* DNA ligase[3]. The ligation mixture was used to transform electrocompetent *E. coli* BL21(DE3) or *E. coli* BL21(DE3) Δ*mtn* cells.

Protein expression. All enzymes described in this study were expressed with a *N*-terminal 6 × His-tag. A colony was used to inoculate LB media containing kanamycin (50 μg/mL). The culture was grown at 37 °C and 200 rpm for 12−14 h. 1−2% of this overnight culture was used to inoculate expression culture in TB media supplemented with kanamycin (50 μg/mL) until OD_{600} reached 0.8, and subsequently induced by addition of isopropylthio-β-D-galactoside (IPTG, final concentration = 0.1 mM). Protein expression was carried out at 18 °C and 200 rpm for 15 h. The cells were finally harvested by centrifugation (4,000 x g, 10 min, 4 °C).

Protein purification. *E. coli* cells were resuspended in the NaPi buffer (50 mM NaPi buffer, pH 7.5, 500 mM NaCl, 20 mM imidazole). Cells were disrupted by sonication. To pellet cell debris, the resulting lysate was then centrifuged at 12,000 x g and 4 ℃ for 20 min using a Lynx 6000 superspeed centrifuge. The supernatant was filtered through a 0.45 μm PES syringe filter and loaded onto a HisTrap HP column (5 mL, GE Healthcare, Piscataway, NJ) using an ÄKTA Start protein purification system (GE healthcare). Proteins were eluted with an increasing gradient of imidazole from 20 to 500 mM in NaPi buffer at a flow rate of 1.0 mL/min. Fractions containing the desired protein were pooled, and subjected to three exchanges of NaPi buffer (50 mM, pH 8.0) using ultracentrifugal filters (10 kDa molecular weight cut-off, Amicon Ultra, Sigma Millipore) to remove excess salt and imidazole. Concentrated proteins were aliquoted, flash-frozen in liquid nitrogen and stored at -80 °C with 20% glycerol as the cryoprotectant until further use. The purity of the protein was further confirmed by SDS-PAGE analysis. Protein concentrations were determined by the Bradford method using the Bradford Protein Assay Kit (Quick Start™, Bio-Rad, USA) prior to use.

Evaluation of SgvM mutants. To a 2 mL vial were added NaPi buffer (50 mM, pH 8.0), SAM (80 μL of 100 mM stock solution in NaPi buffer), purified MTs, purified *Ec*MTAN, and substrate 2-oxo-4-phenylbutanoic acid (**1a**) (250 μL of 10 mM stock solution in NaPi buffer) in succession. Final reaction volume was 1 mL; final concentrations were 2.5 mM substrate **1a**, 8.0 mM SAM, 1.6 – 25 μM CMT, and 2.5 μM *Ec*MTAN. The vials were sealed and shaken at 4 ℃ and 250 rpm for 6 – 24 h. The reaction mixture was then analyzed by chromatographic analysis for the determination of yield, TTN, and enantiomeric ratio (e.r.) as detailed below.

(A) **Product formation analysis using reverse phase HPLC.** After 6 – 24 h, the reaction mixtures were quenched with 2 μL of H_2O_2 (30% w/w) and stirred for 2 h for decarboxylation. Remaining H_2O_2 was decomposed with 1 μL of catalase (Sigma Aldrich, 10 mg/mL stock solution in NaPi buffer). In a 2 mL Eppendorf tube, the reaction mixture (50 μL) was diluted with a mixed solvent system (NaPi buffer : MeCN = 80 : 20, 200 – 950 μL). After centrifugation (12,000 x g, 5 min), the supernatant was filtered through a PTFE membrane

syringe filter (0.22 μm) and transferred to a 500 μL vial insert, which was then placed in a 2 mL HPLC vial and analyzed by reverse-phase HPLC.

(B) Enantioselectivity analysis using chiral normal phase HPLC or chiral GC. After 6 – 24 h, the reaction mixtures were quenched with 2 μL of H_2O_2 (30% w/w) and stirred for 2 h for decarboxylation. Remaining H_2O_2 was decomposed with 1 μL of catalase (Sigma Aldrich, 10 mg/mL stock solution in NaPi buffer). The residual aqueous solution was lyophilized to give the crude carboxylic acid product. A mixed solvent system (hexanes: EtOH: formic acid = 80: 20: 0.1, 500 – 1000 μL) was added. The mixtures were shaken vigorously by hand to uptake the carboxylic acid product and then centrifuged (12,000 x g, 5 min). The supernatant was filtered through a PTFE membrane syringe filter (0.22 μm) and transferred to a 500 μL vial insert, which was then placed in a 2 mL HPLC vial and analyzed by normal phase HPLC. Alternatively, a solution of 1 mM 1,2,3- trimethoxybenzene (internal standard) in EtOAc (0.1% formic acid, 600 μL) was added to the lyophilized crude product to uptake the carboxylic acid product. The mixture was centrifuged (12,000 x g, 5 min), filtered a PTFE membrane syringe filter (0.22 μm) and analyzed by chiral GC.

(C) Product formation screening using HPLC-MS. After 6 – 24 h, the reaction mixtures were quenched by the addition of MeCN (equal volume), shaken vigorously and centrifuged (12,000 x g, 5 min). The supernatant was filtered through a PTFE membrane syringe filter (0.22 μm) and analyzed by HPLC-MS.

HPLC and GC calibration curve development. For reverse phase HPLC analysis, stock solutions of authentic products (10 mM in NaPi buffer: MeCN = 80: 20) were prepared (at least 20 mg of sample was weighed to make a 10 mM stock solution in a mixture of NaPi buffer: MeCN = 80: 20). Product solutions with varying concentrations (0.03125 – 1.0 mM) were then prepared by using a 25 mL volumetric flask. The calibration curves plot the product peak area (y-axis) from reverse phase HPLC analysis against product concentration (mM). For normal phase chiral HPLC analysis or chiral GC analysis, stock solutions of products (100 mM in EtOAc) were prepared. A stock solution of internal standard was freshly prepared (1 mmol, 168 mg of 1,3,5-

trimethoxybenzene was added to 1.0 L EtOAc). To a microcentrifuge tube were added 1 – 100 μL product stock solution and 600 μL internal standard solution to furnish samples for calibration curve development. The mixed solutions were then analyzed by GC. The calibration curves plot product concentration in mM (y-axis) against the ratio of the peak area of product to the peak area of internal standard from GC analysis (x-axis).

Analytical scale biocatalytic asymmetric methylation and other alkylation reactions.

(A) Biocatalytic asymmetric methylation of α-ketoacids. To a 2 mL vial were added NaPi buffer (50 mM, pH 8.0), SAM (80 μL of 100 mM stock solution in NaPi buffer), SgvM F329V T331A M144V (SgvM^{VAV}), *Ec*MTAN, and substrate **1a**–**1m** (250 μL of 10 mM stock solution in NaPi buffer) in succession. Final reaction volume was 1 mL; final concentrations were 2.5 mM substrate, 8.0 mM SAM, 0.4 – 1.6 μM SgvM$^{\mathbf{VAV}}$, 2.5 μM *Ec*MTAN. The vials were sealed and shaken at 30 °C and 250 rpm for 10 – 14 h. The reactions were analyzed using methods described above.

	Substrate **1**	SgvM^{VAV} (μM)	Reaction time (h)
General method	**1a**, **1c**, **1d**, **1e**, **1f**, **1h**, **1i**, **1j**, **1k**	1.6	14
Optimized method I	**1b**, **1l**, **1m**	0.8	14
Optimized method II	**1g**	0.4	10

(B) Biocatalytic asymmetric α-allylation, propargylation and ethylation of α-ketoacids. To a 2 mL vial were added the buffer (pH 8.0, 50 mM Tris-HCl, 20 mM $MgCl_2$, and 200 mM KCl), L-Methionine analogue (40 μL of 100 mM stock solution in buffer), ATP (40 μL of 100 mM stock solution in buffer), *Bs*MAT I317A, SgvM^{VAV}, *Ec*MTAN, and substrate **1a** (250 μL of 10 mM stock solution in assay buffer) in succession. Final reaction volume was 1 mL;

For asymmetric α-allylation, final concentrations were 2.5 mM substrate **1a**, 4.0 mM *S*-allyl-L-homocysteine, 4.0 mM ATP, 50 μM *Bs*MAT I317A, 1.6 μM SgvM^{VAV}, 2.5 μM *Ec*MTAN. The vials were sealed and shaken at 30 °C and 250 rpm for 14 h;

For asymmetric α-propargylation, final concentrations were 2.5 mM substrate **1a**, 4.0 mM *S*-(prop-2-yn-1-yl)-L-homocysteine, 4.0 mM ATP, 50 μM *Bs*MAT I317A, 25 μM SgvM^{VAV}, 2.5 μM *Ec*MTAN. The vials were sealed and shaken at 30 °C and 250 rpm for 8 h;

For asymmetric α-ethylation, final concentrations were 2.5 mM substrate **1a**, 4.0 mM L-ethionine, 4.0 mM ATP, 125 μM *Bs*MAT I317A, 125 μM SgvM^{VAV}, 2.5 μM *Ec*MTAN. The vials were sealed and shaken at 30 °C and 250 rpm for 14 h. The reactions were then analyzed using methods described above.

Sequence similarity network of HMTs. The SSN was created using the Enzyme Similarity Tool developed by the Enzyme Function Initiative An all-by-all BLAST (e-value: 10^{-5}) produced alignment scores for *Pseudomonas sp.* HMTs, previously reported HMTs, and a representative library of sequences from the thiopurine *S*-methyltransferases family (PfamPF05724). Only one in thirty randomly selected sequences from the thiopurine *S*-methyltransferases family were included to reduce the network from 12,188 sequences to a more manageable 408 sequences. Following initial calculations, the SSN was finalized with an alignment score threshold of 30. The full network was visualized in Cytoscape 3.9.1 using the 'yFiles Organic' layout. Clusters containing two nodes or less were deleted.

HMT enzymatic activity assay. To a 2 mL vial were added NaPi buffer (50 mM, pH 8.0, 370 μL), SAH (100 mM stock solution in DMSO, 4 μL), and HMT (diluted purified enzyme, 6 μL). After incubation at 30 °C with 250 rpm shaking for 5 min, MeI (200 mM stock solution in DMSO, 20 μL) was added to the solution. The reaction mixture was incubated at 30 °C with 250 rpm shaking for another 5 min. The reaction mixture was quickly quenched by adding an equal volume of MeCN and centrifuged (12,000 x g, 5 min). The supernatant was filtered through a PTFE membrane syringe filter (0.22 μm) and transferred to a 500 μL vial insert, which was then placed in a 2 mL HPLC vial and analyzed by reverse phase HPLC. Conditions: YMC-Pack Pro C18 column; mobile phase: 10 mM sodium dihydrogen phosphate containing 5 mM sodium 1-heptanesulfonate (pH 3.5):MeCN=9:1; 1 mL/min; 40 °C; 260 nm. One unit (U) of HMT was defined as the amount of protein that catalyzed the formation of 1 μmol SAM per min. For the calculation of relative activity, the value of *Pa*HMT specific activity was set as 100%.

Biocatalytic asymmetric methylation with methyl iodide using SgvM^{VAV} and HMT. To a 2 mL vial were added NaPi buffer (50 mM, pH 8.0), SAH (2.5 μL of 1 mM stock solution in DMSO), MeI (25 μL of 200 mM stock solution in DMSO), SgvM^{VAV}, HMT, and substrate **1a** (250 μL of 10 mM stock solution in NaPi buffer) in succession. Final reaction volume was 1 mL; final concentrations were 2.5 mM substrate, 2.5 μM SAH, 5.0 mM MeI, 0.25 – 2.5 μM SgvM^{VAV}, 0.25 – 2.5 μM HMT. The vials were sealed and shaken at 30 °C and 250 rpm for 6 – 24 h. The reactions were then analyzed using methods described above.

II. Evaluation of enzymes for biocatalytic asymmetric alkylation

Supplementary Table 2. CMTs evaluated in this study.

Entry	MT	Source	GenBank ID	Identity
1	SgvM	*Streptomyces griseoviridis*	AGN74875.1 [https://www.ncbi.nlm.nih.gov/protein/AGN74875.1]	100%
2	MarI	*Streptomyces* sp. B9173	AHJ60978.1 [https://www.ncbi.nlm.nih.gov/protein/AHJ60978.1]	23%
3	MppJ	*Streptomyces hygroscopicus*	AAU34201.1 [https://www.ncbi.nlm.nih.gov/protein/AAU34201.1]	28%
4	CdpJ	*Chondromyces crocatus*	AKT41218.1 [https://www.ncbi.nlm.nih.gov/protein/AKT41218.1]	25%

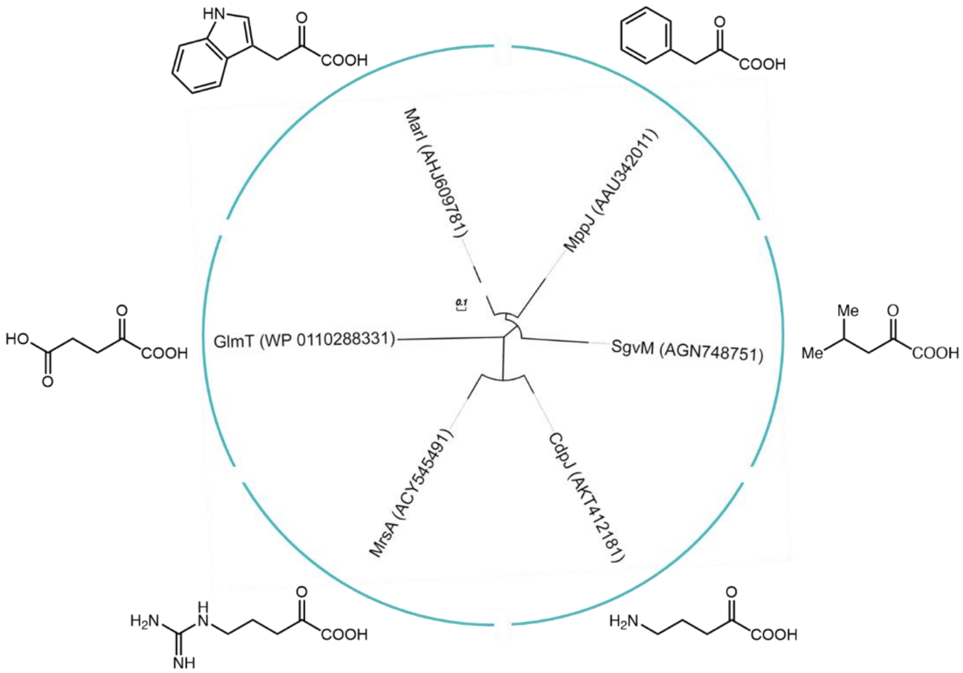

Supplementary Figure 2. Phylogenetic analysis of representative CMTs for asymmetric methylation of α-keto acids. The phylogenetic tree was constructed using the neighbor-joining method. These enzymes were mined from *Streptomyces griseoviridis* (SgvM), *Streptomyces hygroscopicus* (MppJ), *Streptomyces* sp. B9173 (MarI), *Streptomyces coelicolor* A3(2) (GlmT), *Pseudomonas syringae* (MrsA), *Chondromyces crocatus* (CdpJ). Structure of natural substrates are shown.

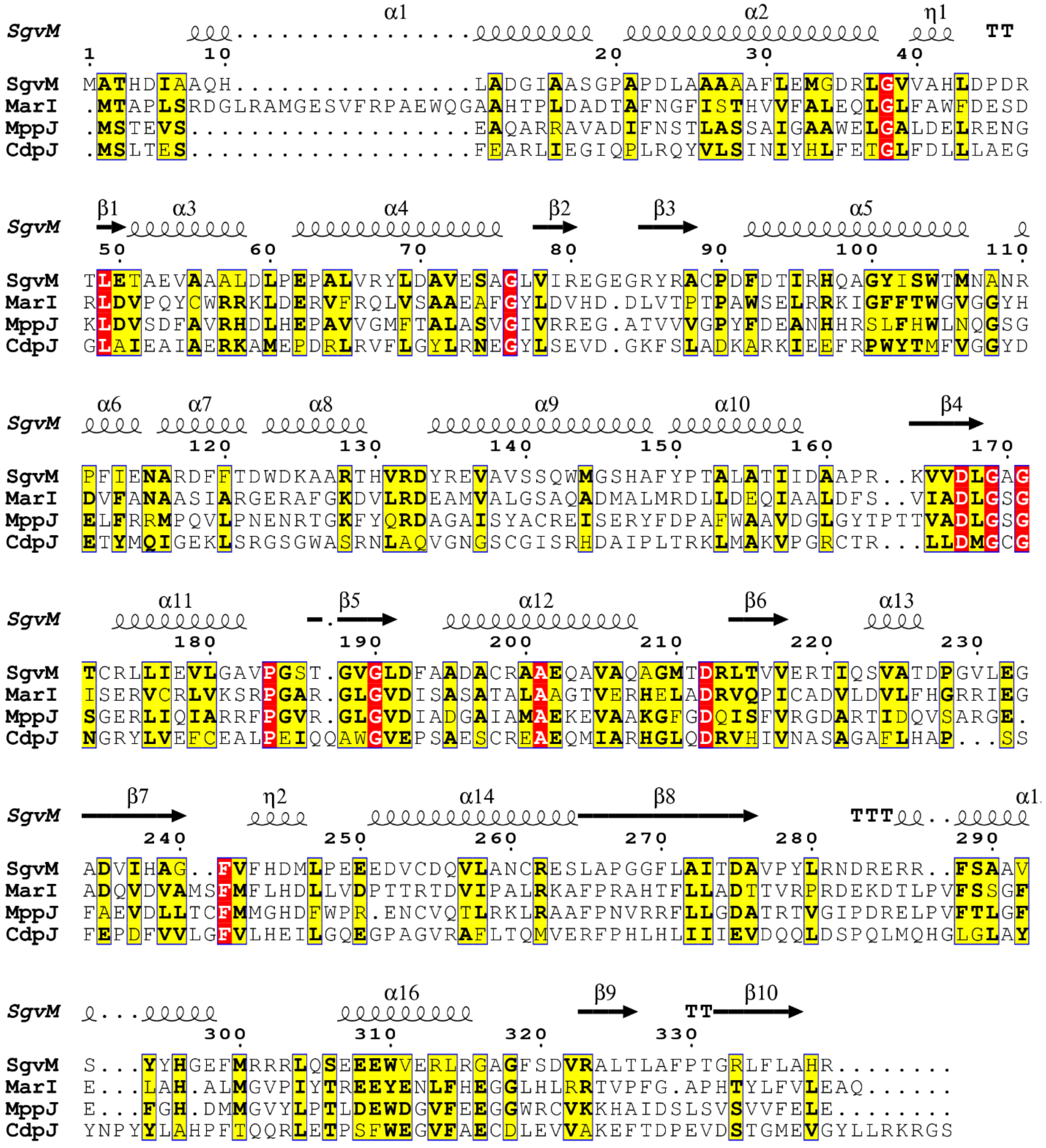

Supplementary Figure 3. Multiple sequence alignment (MSA) of four CMTs. MSA was performed using the MUSCLE server (https://www.ebi.ac.uk/Tools/msa/muscle/) and displayed using Esprit (http://espript.ibcp.fr).

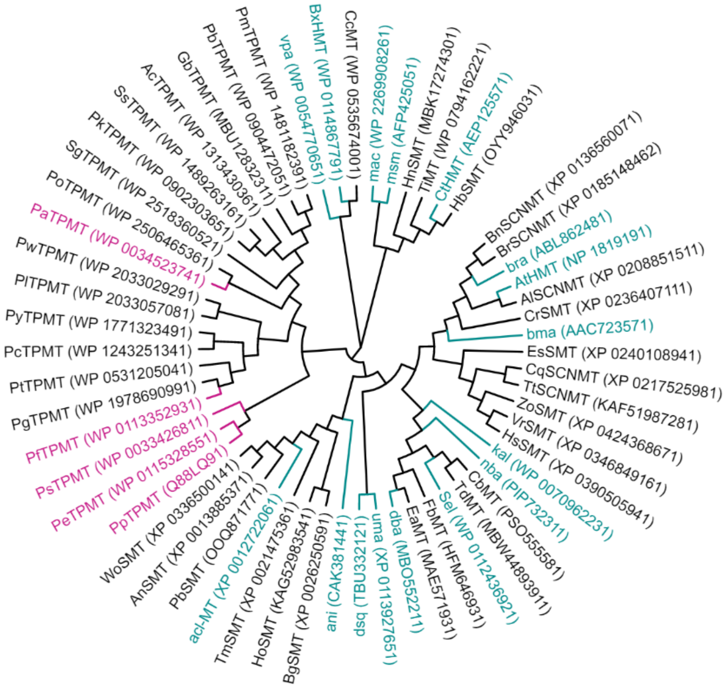

Supplementary Figure 4. Phylogenetic analysis of putative HMTs. The phylogenetic tree was constructed using the neighbor-joining method. MT: methyltransferase, SMT: thiol methyltransferase, SCNMT: thiocyanate methyltransferase, TPMT: thiopurine *S*-methyltransferase. *Pseudomonas* sp. HMTs investigated in this study were shown as MediumVioletRed, while the other HMTs previously reported were shown as DarkCyan. This result suggested that *Pseudomonas* sp. HMTs are in a clade distinct from those HMTs previously known.

Supplementary Table 3. Analysis of *Pa*HMT and other HMTs

Entry	HMT	Source	GenBank ID	Identity	ref.
1	*Pa*HMT	*Pseudomonas aeruginosa* PAO1	WP_003114764.1 [https://www.ncbi.nlm.nih.gov/protein/WP_003114764.1]	100%	This work
2	*Pe*HMT	*Pseudomonas entomophila*	WP_011532855.1 [https://www.ncbi.nlm.nih.gov/protein/WP_011532855.1]	55%	This work
3	*Pf*HMT	*Pseudomonas fluorescens* Pf0-1	WP_011335293.1 [https://www.ncbi.nlm.nih.gov/protein/WP_011335293.1]	56%	This work
4	*Pp*HMT	*Pseudomonas putida* KT2440	WP_010952898.1 [https://www.ncbi.nlm.nih.gov/protein/WP_010952898.1]	53%	This work
5	*Ps*HMT	*Pseudomonas syringae* pv. Pisi	WP_003342681.1 [https://www.ncbi.nlm.nih.gov/protein/WP_003342681.1]	46%	This work
6	*Ct*HMT	*Chloracidobacterium thermophilum* B	AEP12557.1 [https://www.ncbi.nlm.nih.gov/protein/AEP12557.1]	27%	[1]
7	*Bx*HMT	*Paraburkholderia xenovorans* LB400	WP_011486779.1 [https://www.ncbi.nlm.nih.gov/protein/WP_011486779.1]	30%	[4]
8	*At*HMT	*Arabidopsis thaliana*	NP_181919.1 [https://www.ncbi.nlm.nih.gov/protein/NP_181919.1]	23%	[5]
9	*acl*-MT	*Aspergillus clavatus* NRRL 1	XP_001272206.1 [https://www.ncbi.nlm.nih.gov/protein/XP_001272206.1]	31%	[6]

III. Evaluation of SgvM variants in the biocatalytic asymmetric methylation: complete data

According to the crystal structure of wt SgvM in complex with SAH and α-ketoleucine, 10 amino acid residues that lie in proximity to α-ketoleucine were selected and subjected to alanine scanning (Supplementary Figure 5a). These variants were screened against substrate **1a**. It was found that mutations M144A, F329A, and T331A could significantly increase enzyme activity (Supplementary Figure 5b).

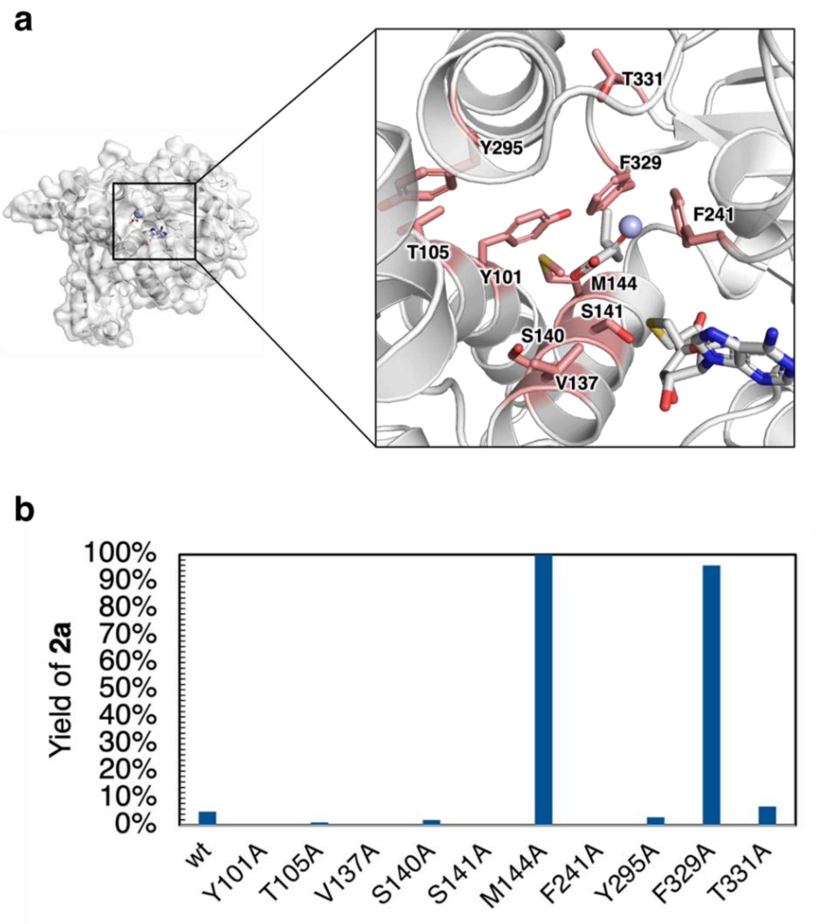

Supplementary Figure 5. Summary of alanine scanning of SgvM. a Residues (Y101, T105, V137, S140, S141, M144, F241, Y295, F329, T331) selected for alanine scanning are shown. **b** Screening results of alanine scanning library.

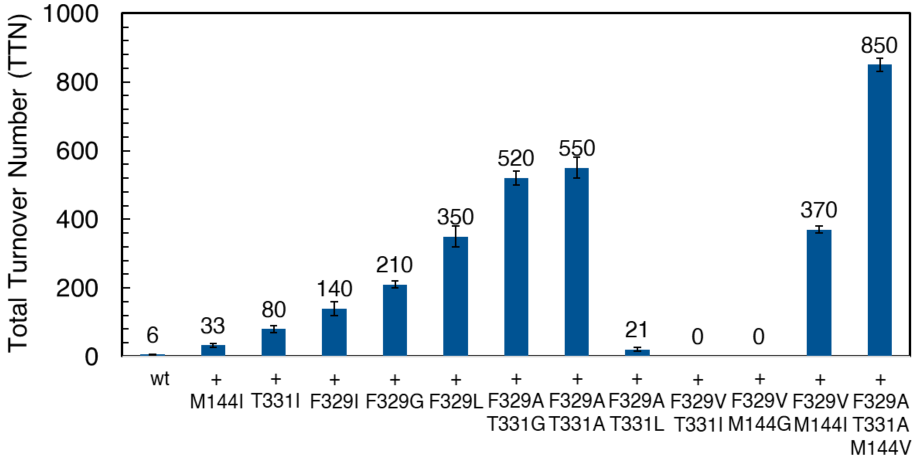

Supplementary Figure 6. Additional data enzyme engineering.

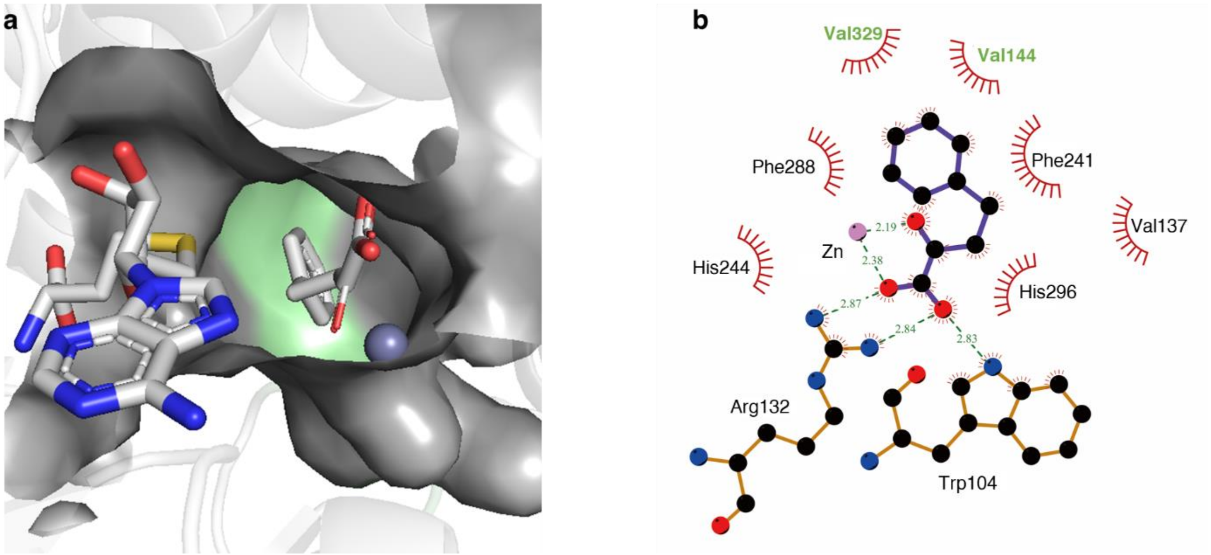

Supplementary Figure 7. Structure analysis of SgvM^{VAV} in complex with substrate 1a. **a** The substrate (atom-coloured sticks, carbon grey) is well accommodated within hydrophobic active site pocket (shown as grey and green surface). The grey surface is composed of residuals W104, V137, A138, F149, F241, A276, V277, F288, A291, V292, L304, P330, G332. The green surface is composed of residuals V144, V329, A331. **b** LigPlot+[7] diagram for the interactions between SgvM^{VAV} and substrate **1a**. Hydrogen bonds and salt bridges are shown as green, dashed lines and distances given. Hydrophobic interactions are indicated by red spoked arcs. V329 and V144 are shown as green.

Supplementary Discussion

The crystal structure suggests that substrate **1a** resides in a hydrophobic activity site pocket with its aromatic ring buried in a cavity surrounded by V144, V329, A331. These three amino acid residuals may play an important role in shaping the active site cavity and controlling the enzyme activity.

IV. Kinetic studies

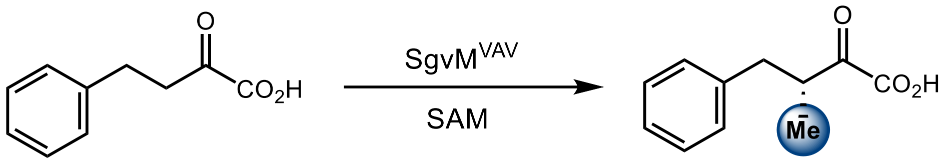

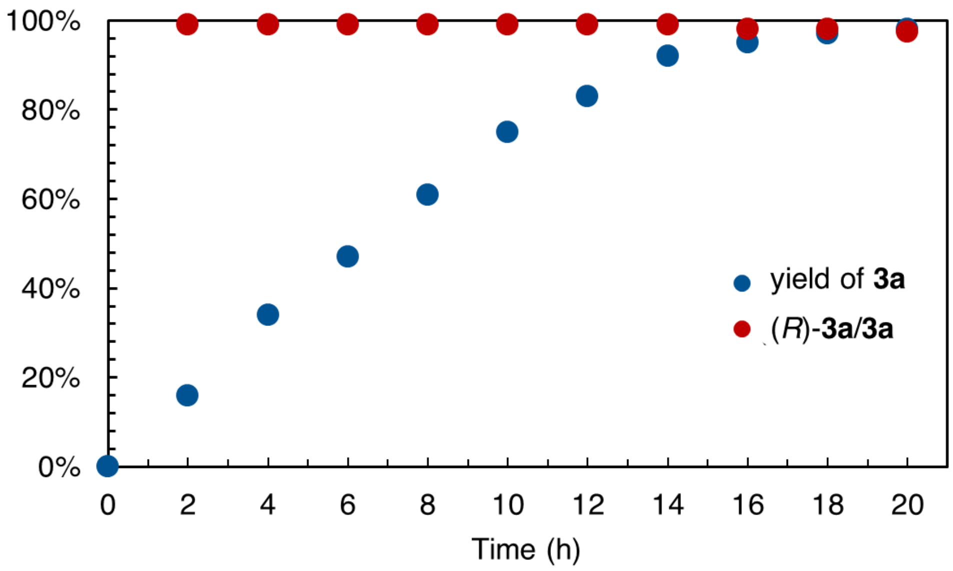

Supplementary Figure 8. Time-course study of SgvM^{VAV} catalyzed asymmetric methylation of substrate **1a**.

Supplementary Discussion

As can be seen from this time-course study, this biocatalytic asymmetric methylation reached >90% conversion after 14 h. Under these conditions, the enantioenriched methylated product remains relatively configurationally stable. No significant racemization was observed at 92% conversion. A low level of racemization was observed after 20 h (97.5:2.5 e.r.).

Supplementary Table 4. pH effect

buffer	14 h		20 h	
	yield (**2a**)	e.r. (**2a**)	yield (**2a**)	e.r. (**2a**)
Tris-HCl (50 mM, pH 8.0)	94%	99:1	99%	97:3
Tris-HCl (50 mM, pH 7.5)	82%	99:1	92%	99:1
Tris-HCl (50 mM, pH 7.0)	75%	>99:1	84%	99:1
Na_2HPO_4-NaH_2PO_4 (50 mM, pH 6.5)	63%	>99:1	71%	99:1
Na_2HPO_4-NaH_2PO_4 (50 mM, pH 6.0)	35%	>99:1	42%	99:1

Supplementary Discussion

As can be seen from these results, engineered enzyme SgvM^{VAV} displayed higher activity under basic conditions. At pH = 8, a low level of racemization was observed after 20 h. At pH = 7.5, similar yields could be obtained and minimal racemization was observed. Thus, these biocatalytic asymmetric methylation reactions can be carried out at pH 7.5 to circumvent the racemization issue.

Michaelis-Menten kinetics

To a 1.5 mL Eppendorf tube was added NaPi buffer (50 mM, pH 8.0, 367 μL), SAM (20 mM stock solution in NaPi buffer, 4 μL), *Ec*MTAN (purified enzyme, 3 μL), and SgvM^{VAV} (diluted purified enzyme, 6 μL). After incubation at 30 °C with 250 rpm shaking for 5 min, substrate **1a** (different concentrations, 20 μL) was added to the solution. Final concentrations were 0~2 mM substrate **1a**, 0.2 mM SAM, 2.5 μM *Ec*MTAN, and 3.0 mM SgvM^{VAV}. The reaction mixture was incubated at 30 °C with 250 rpm shaking for another 5 min. The reaction mixtures were quickly quenched with 8 μL of H_2O_2 (30% w/w) and stirred for 10 min. Remaining H_2O_2 was decomposed with 1 μL of catalase (Sigma Aldrich, 10 mg/mL stock solution in NaPi buffer). An equal volume of MeCN was then added to the reaction mixture. After centrifugation for 2 min, the supernatant was filtered through a PTFE membrane syringe filter (0.22 μm) and transferred to a 500 μL vial insert, which was then placed in a 2 mL HPLC vial and analyzed by reverse phase HPLC. The kinetic constants were calculated by fitting the data point to the Michaelis-Menten equation. The k_{cat} and K_M of SgvM^{VAV} on **1a** were determined to be (4.46 ± 0.53) min^{-1} and (220 ± 30) μM, respectively.

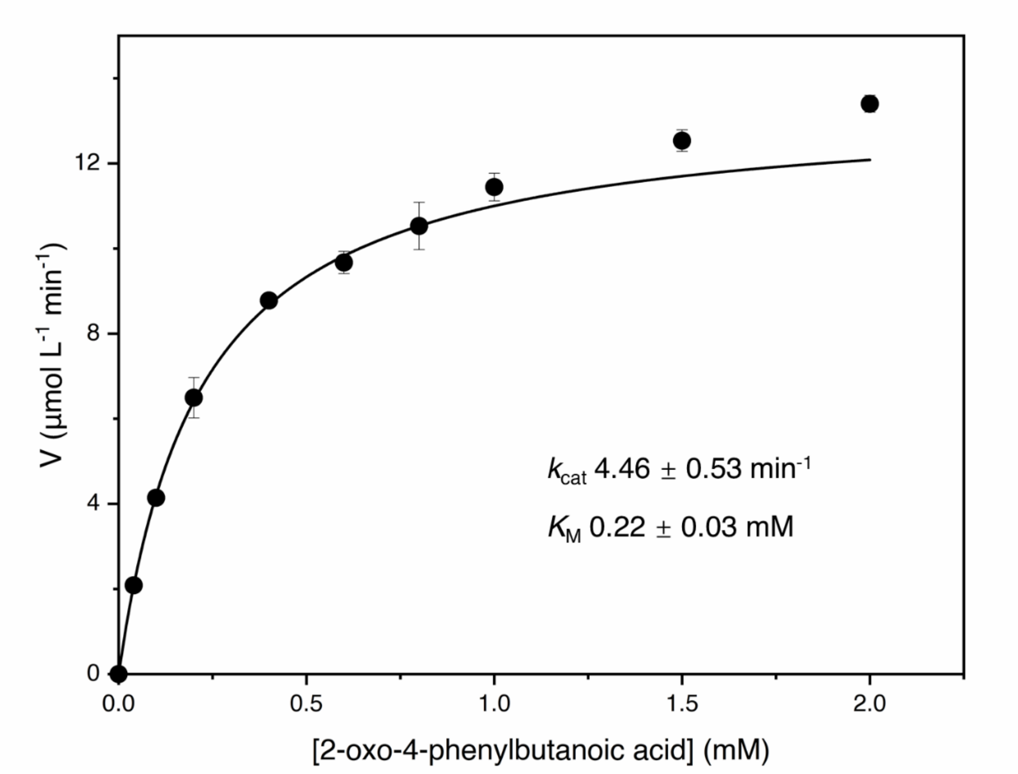

Supplementary Figure 9. Kinetic parameters of SgvM^{VAV}.

V. SDS-PAGE analysis of purified enzymes used in the current study

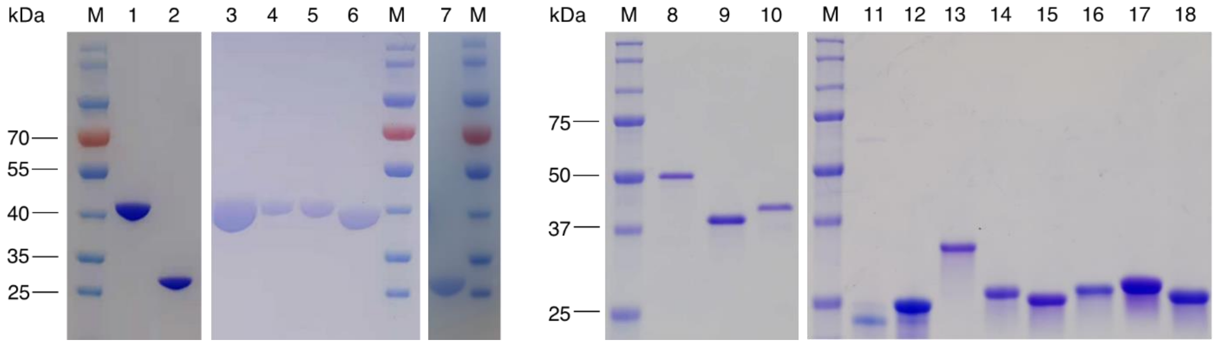

Supplementary Figure 10. SDS-PAGE analysis of SAM-dependent enzymes described in the current study. Lane M, protein marker; lane 1, SgvM^{VAV}; lane 2, *Pa*HMT; lane 3, SgvM; lane 4, CdpJ; lane 5, MarI; lane 6, MppJ; lane 7, *Ec*MTAN; lane 8, *Bs*MAT I317A; lane 9, YiaE; lane 10, *Km*AT; lane 11, *Ct*HMT; lane 12, *Bx*HMT, lane 13, *acl*-MT; lane 14, *At*HMT; lane 15, *Ps*HMT; lane 16, *Pe*HMT; lane 17, *Pf*HMT; lane 18, *Pp*HMT.

VI. Nucleotide and amino acid sequences

DNA and amino acid sequence of SgvM:

```
ATGGGCAGCAGCCATCATCATCATCATCACAGCAGCGGCCTGGTGCCGCGCGGCAGCCATATGGC
CACCCATGATATTGCAGCACAGCATCTGGCAGATGGTATTGCCGCAAGCGGTCCGGCACCGGATC
TGGCAGCAGCAGCCGCATTTCTGGAAATGGGTGATCGTCTGGGTGTTGTTGCACATCTGGATCCT
GATCGTACCCTGGAAACCGCAGAAGTTGCAGCAGCACTGGATCTGCCGGAACCGGCACTGGTTCG
TTATCTGGATGCAGTTGAAAGCGCAGGTCTGGTTATTCGTGAAGGTGAAGGTCGTTATCGTGCAT
GTCCGGATTTTGATACCATTCGTCATCAGGCAGGTTATATTAGCTGGACCATGAATGCAAATCGT
CCGTTTATTGAAAATGCCCGTGATTTTTTCACCGATTGGGATAAAGCAGCACGTACCCATGTTCG
TGATTATCGTGAAGTTGCCGTTAGCAGCCAGTGGATGGGTAGCCATGCATTTTATCCGACCGCAC
TGGCAACCATTATTGATGCAGCACCGCGTAAAGTTGTTGATCTGGGTGCAGGCACCTGTCGTCTG
CTGATTGAAGTTCTGGGTGCCGTTCCGGGTAGCACCGGTGTTGGTCTGGATTTTGCAGCAGATGC
ATGTCGTGCAGCAGAACAGGCAGTTGCACAGGCAGGTATGACCGATCGTCTGACCGTTGTTGAAC
GTACCATTCAGAGCGTTGCAACCGATCCGGGTGTTCTGGAAGGTGCAGATGTTATTCATGCAGGT
TTTGTGTTTCATGATATGCTGCCGGAAGAGGAAGATGTTTGCGATCAGGTTCTGGCAAATTGTCG
```

TGAAAGCCTGGCACCGGGTGGTTTTCTGGCAATTACCGATGCAGTTCCGTATCTGCGTAATGATC
GTGAACGTCGTTTTAGCGCAGCAGTTAGCTATTATCATGGTGAATTTATGCGTCGTCGTCTGCAG
AGCGAAGAAGAATGGGTTGAACGTCTGCGTGGTGCCGGTTTTAGTGATGTTCGTGCACTGACCCT
GGCATTTCCGACAGGTCGTCTGTTTCTGGCACATCGTTAA

MGSSHHHHHHSSGLVPRGSHMATHDIAAQHLADGIAASGPAPDLAAAAAFLEMGDRLGVVAHLDP
DRTLETAEVAAALDLPEPALVRYLDAVESAGLVIREGEGRYRACPDFDTIRHQAGYISWTMNANR
PFIENARDFFTDWDKAARTHVRDYREVAVSSQWMGSHAFYPTALATIIDAAPRKVVDLGAGTCRL
LIEVLGAVPGSTGVGLDFAADACRAAEQAVAQAGMTDRLTVVERTIQSVATDPGVLEGADVIHAG
FVFHDMLPEEEDVCDQVLANCRESLAPGGFLAITDAVPYLRNDRERRFSAAVSYYHGEFMRRRLQ
SEEEWVERLRGAGFSDVRALTLAFPTGRLFLAHR

DNA and amino acid sequence of SgvM^{VAV}:

ATGGGCAGCAGCCATCATCATCATCATCACAGCAGCGGCCTGGTGCCGCGCGGCAGCCATATGGC
CACCCATGATATTGCAGCACAGCATCTGGCAGATGGTATTGCCGCAAGCGGTCCGGCACCGGATC
TGGCAGCAGCAGCCGCATTTCTGGAAATGGGTGATCGTCTGGGTGTTGTTGCACATCTGGATCCT
GATCGTACCCTGGAAACCGCAGAAGTTGCAGCAGCACTGGATCTGCCGGAACCGGCACTGGTTCG
TTATCTGGATGCAGTTGAAAGCGCAGGTCTGGTTATTCGTGAAGGTGAAGGTCGTTATCGTGCAT
GTCCGGATTTTGATACCATTCGTCATCAGGCAGGTTATATTAGCTGGACCATGAATGCAAATCGT
CCGTTTATTGAAAATGCCCGTGATTTTTTCACCGATTGGGATAAAGCAGCACGTACCCATGTTCG
TGATTATCGTGAAGTTGCCGTTAGCAGCCAGTGGGTTGGTAGCCATGCATTTTATCCGACCGCAC
TGGCAACCATTATTGATGCAGCACCGCGTAAAGTTGTTGATCTGGGTGCAGGCACCTGTCGTCTG
CTGATTGAAGTTCTGGGTGCCGTTCCGGGTAGCACCGGTGTTGGTCTGGATTTTGCAGCAGATGC
ATGTCGTGCAGCAGAACAGGCAGTTGCACAGGCAGGTATGACCGATCGTCTGACCGTTGTTGAAC
GTACCATTCAGAGCGTTGCAACCGATCCGGGTGTTCTGGAAGGTGCAGATGTTATTCATGCAGGT
TTTGTGTTTCATGATATGCTGCCGGAAGAGGAAGATGTTTGCGATCAGGTTCTGGCAAATTGTCG
TGAAAGCCTGGCACCGGGTGGTTTTCTGGCAATTACCGATGCAGTTCCGTATCTGCGTAATGATC
GTGAACGTCGTTTTAGCGCAGCAGTTAGCTATTATCATGGTGAATTTATGCGTCGTCGTCTGCAG
AGCGAAGAAGAATGGGTTGAACGTCTGCGTGGTGCCGGTTTTAGTGATGTTCGTGCACTGACCCT
GGCAGTTCCGGCAGGTCGTCTGTTTCTGGCACATCGTTAA

MGSSHHHHHHSSGLVPRGSHMATHDIAAQHLADGIAASGPAPDLAAAAAFLEMGDRLGVVAHLDP
DRTLETAEVAAALDLPEPALVRYLDAVESAGLVIREGEGRYRACPDFDTIRHQAGYISWTMNANR
PFIENARDFFTDWDKAARTHVRDYREVAVSSQWVGSHAFYPTALATIIDAAPRKVVDLGAGTCRL
LIEVLGAVPGSTGVGLDFAADACRAAEQAVAQAGMTDRLTVVERTIQSVATDPGVLEGADVIHAG
FVFHDMLPEEEDVCDQVLANCRESLAPGGFLAITDAVPYLRNDRERRFSAAVSYYHGEFMRRRLQ
SEEEWVERLRGAGFSDVRALTLAVPAGRLFLAHR

DNA and amino acid sequence of MarI:

ATGGGCAGCAGCCATCATCATCATCATCACAGCAGCGGCCTGGTGCCGCGCGGCAGCCATATGAC
CGCACCGCTGAGTCGCGATGGCCTGCGCGCTATGGGTGAAAGTGTTTTTCGCCCGGCCGAATGGC
AGGGCGCCGCACACACACCGCTGGATGCAGATACCGCATTCAATGGCTTTATTAGCACCCATGTT
GTTTTTGCCCTGGAACAGCTGGGTCTGTTTGCATGGTTTGATGAAAGTGATCGTCTGGATGTTCC
GCAGTATTGTTGGCGCCGCAAACTGGATGAACGTGTTTTTCGTCAGCTGGTGAGCGCAGCAGAAG
CCTTTGGCTATCTGGATGTGCATGATGATCTGGTGACCCCGACCCCGGCATGGAGTGAACTGCGT
CGCAAAATTGGTTTCTTTACCTGGGGTGTTGGTGGCTATCATGATGTTTTTGCCAATGCAGCAAG
CATTGCACGTGGTGAACGCGCATTTGGCAAAGATGTGCTGCGCGATGAAGCAATGGTTGCACTGG
GCAGTGCACAGGCCGATATGGCACTGATGCGTGATCTGCTGGATGAACAGATTGCAGCACTGGAT
TTTAGTGTTATTGCCGATCTGGGTAGCGGTATTAGCGAACGCGTTTGTCGTCTGGTTAAAAGCCG
CCCGGGTGCCCGCGGTCTGGGTGTGGACATTAGCGCAAGCGCAACCGCCCTGGCAGCCGGTACCG
TGGAACGTCATGAACTGGCAGATCGCGTGCAGCCGATTTGCGCCGATGTGCTGGATGTTCTGTTT
CATGGCCGTCGTATTGAAGGTGCAGATCAGGTGGATGTGGCAATGAGCTTTATGTTTCTGCATGA
TCTGCTGGTGGACCCTACCACCCGTACCGATGTTATTCCGGCACTGCGTAAAGCCTTTCCGCGCG
CCCATACCTTTCTGCTGGCAGATACCACCGTGCGTCCGCGCGATGAAAAAGATACCCTGCCGGTG
TTTAGCAGCGGTTTTGAACTGGCCCATGCCCTGATGGGCGTTCCGATCTATACCCGCGAAGAATA
TGAAAATCTGTTTCATGAAGGCGGCCTGCATCTGCGTCGTACCGTTCCGTTTGGCGCACCGCATA
CCTATCTGTTTGTGCTGGAAGCCCAGTAA

MGSSHHHHHHSSGLVPRGSHMTAPLSRDGLRAMGESVFRPAEWQGAAHTPLDADTAFNGFISTHV
VFALEQLGLFAWFDESDRLDVPQYCWRRKLDERVFRQLVSAAEAFGYLDVHDDLVTPTPAWSELR

RKIGFFTWGVGGYHDVFANAASIARGERAFGKDVLRDEAMVALGSAQADMALMRDLLDEQIAALD
FSVIADLGSGISERVCRLVKSRPGARGLGVDISASATALAAGTVERHELADRVQPICADVLDVLF
HGRRIEGADQVDVAMSFMFLHDLLVDPTTRTDVIPALRKAFPRAHTFLLADTTVRPRDEKDTLPV
FSSGFELAHALMGVPIYTREEYENLFHEGGLHLRRTVPFGAPHTYLFVLEAQ

DNA and amino acid sequence of MppJ:

ATGGGCAGCAGCCATCATCATCATCATCACAGCAGCGGCCTGGTGCCGCGCGGCAGCCATATGAG
TACCGAAGTTAGTGAAGCCCAGGCCCGTCGCGCAGTGGCCGATATTTTTAATAGCACCCTGGCCA
GTAGCGCAATTGGTGCAGCATGGGAACTGGGCGCCCTGGATGAACTGCGCGAAAATGGCAAACTG
GATGTGAGCGATTTTGCAGTTCGTCATGATCTGCATGAACCGGCCGTGGTGGGTATGTTTACCGC
CCTGGCCAGCGTTGGTATTGTTCGTCGCGAAGGTGCAACCGTTGTGGTGGGTCCGTATTTTGATG
AAGCAAATCATCATCGTAGTCTGTTTCATTGGCTGAATCAGGGTAGCGGTGAACTGTTTCGCCGC
ATGCCGCAGGTTCTGCCGAATGAAAATCGCACCGGTAAATTTTATCAGCGCGATGCAGGTGCCAT
TAGTTATGCCTGCCGTGAAATTAGCGAACGCTATTTTGATCCGGCCTTTTGGGCAGCCGTTGATG
GCCTGGGCTATACCCCGACCACCGTGGCAGATCTGGGCAGTGGTAGTGGTGAACGTCTGATTCAG
ATTGCCCGCCGTTTTCCGGGCGTGCGCGGTTTAGGCGTTGATATTGCCGATGGTGCAATTGCCAT
GGCCGAAAAAGAAGTGGCAGCCAAAGGCTTTGGCGATCAGATTAGCTTTGTGCGCGGCGATGCAC
GCACCATTGATCAGGTTAGCGCACGCGGCGAATTTGCCGAAGTGGATCTGCTGACCTGCTTTATG
ATGGGTCATGATTTTTGGCCGCGCGAAAATTGCGTTCAGACCCTGCGCAAACTGCGTGCCGCATT
TCCGAATGTGCGTCGCTTTCTGCTGGGCGATGCCACCCGCACCGTGGGTATTCCGGATCGTGAAC
TGCCGGTGTTTACCCTGGGTTTTGAATTTGGTCATGATATGATGGGTGTTTATCTGCCGACCCTG
GATGAATGGGATGGTGTGTTTGAAGAAGGTGGTTGGCGCTGTGTTAAAAAACATGCAATTGATAG
TCTGAGTGTTAGCGTTGTGTTTGAACTGGAATAA

MGSSHHHHHHSSGLVPRGSHMSTEVSEAQARRAVADIFNSTLASSAIGAAWELGALDELRENGKL
DVSDFAVRHDLHEPAVVGMFTALASVGIVRREGATVVVGPYFDEANHHRSLFHWLNQGSGELFRR
MPQVLPNENRTGKFYQRDAGAISYACREISERYFDPAFWAAVDGLGYTPTTVADLGSGSGERLIQ
IARRFPGVRGLGVDIADGAIAMAEKEVAAKGFGDQISFVRGDARTIDQVSARGEFAEVDLLTCFM
MGHDFWPRENCVQTLRKLRAAFPNVRRFLLGDATRTVGIPDRELPVFTLGFEFGHDMMGVYLPTL
DEWDGVFEEGGWRCVKKHAIDSLSVSVVFELE

DNA and amino acid sequence of CdpJ:

ATGGGCAGCAGCCATCATCATCATCATCACAGCAGCGGCCTGGTGCCGCGCGGCAGCCATATGAG
CCTGACCGAAAGTTTTGAAGCACGTCTGATTGAAGGCATTCAGCCGCTGCGCCAGTATGTGCTGA
GTATTAATATCTATCATCTGTTTGAGACCGGCCTGTTTGATCTGCTGCTGGCCGAAGGCGGTCTG
GCCATTGAAGCAATTGCAGAACGTAAAGCAATGGAACCGGATCGCCTGCGCGTTTTTCTGGGTTA
TCTGCGTAATGAAGGTTATCTGAGCGAAGTTGATGGTAAATTTTCACTGGCAGATAAAGCCCGCA
AAATTGAAGAATTTCGCCCGTGGTATACCATGTTTGTGGGTGGTTATGATGAAACCTATATGCAG
ATTGGTGAAAAACTGAGCCGCGGCAGTGGTTGGGCCAGCCGTAATCTGGCACAGGTGGGTAATGG
CAGCTGTGGTATTAGTCGCCATGATGCCATTCCGCTGACCCGTAAACTGATGGCCAAAGTTCCGG
GTCGTTGTACCCGTCTGCTGGATATGGGCTGCGGTAATGGCCGCTATCTGGTTGAATTTTGTGAA
GCCCTGCCGGAAATTCAGCAGGCATGGGGTGTGGAACCGAGTGCCGAAAGTTGTCGTGAAGCCGA
ACAGATGATTGCCCGCCACGGTCTGCAGGATCGTGTTCATATTGTTAATGCCAGCGCCGGTGCCT
TTCTGCATGCACCGAGCAGCTTTGAACCGGATTTTGTGGTGCTGGGCTTTGTTCTGCATGAAATT
CTGGGTCAGGAAGGCCCGGCCGGCGTGAGAGCTTTTCTGACCCAGATGGTTGAACGTTTTCCGCA
TCTGCATCTGATTATTATTGAAGTGGATCAGCAGCTGGATAGCCCGCAGCTGATGCAGCATGGCC
TGGGCCTGGCATATTATAATCCGTATTATCTGGCACATCCGTTTACCCAGCAGCGCCTGGAAACC
CCGAGCTTTTGGGAAGGCGTTTTTGCCGAATGTGATCTGGAAGTGGTGGCCAAAGAATTCACTGA
TCCGGAAGTGGATAGCACCGGCATGGAAGTTGGCTATCTGCTGCGCAAACGTGGCAGTTAA

MGSSHHHHHHSSGLVPRGSHMSLTESFEARLIEGIQPLRQYVLSINIYHLFETGLFDLLLAEGGL
AIEAIAERKAMEPDRLRVFLGYLRNEGYLSEVDGKFSLADKARKIEEFRPWYTMFVGGYDETYMQ
IGEKLSRGSGWASRNLAQVGNGSCGISRHDAIPLTRKLMAKVPGRCTRLLDMGCGNGRYLVEFCE
ALPEIQQAWGVEPSAESCREAEQMIARHGLQDRVHIVNASAGAFLHAPSSFEPDFVVLGFVLHEI
LGQEGPAGVRAFLTQMVERFPHLHLIIIEVDQQLDSPQLMQHGLGLAYYNPYYLAHPFTQQRLET
PSFWEGVFAECDLEVVAKEFTDPEVDSTGMEVGYLLRKRGS

DNA and amino acid sequence of *Bs*MAT:

ATGGGCAGCAGCCATCATCATCATCATCACAGCAGCGGCCTGGTGCCGCGCGGCAGCCATATGGC
TAGCATGACTGGTGGACAGCAAATGGGTCGCGGATCCATGAGTAAAAATCGTCGTTTATTTACAT
CAGAATCTGTTACGGAGGGGCATCCGGATAAAATCTGTGACCAGATTTCTGACAGCATTTTAGAT

GAAATTTTAAAGAAAGACCCTAACGCGCGTGTTGCTTGTGAAACATCTGTGACAACAGGTTTGGT
TCTTGTAAGCGGAGAGATCACAACTTCTACGTATGTTGACATTCCGAAAACGGTTCGCCAAACCA
TTAAAGAAATCGGATACACACGTGCAAAATACGGATTTGATGCGGAAACTTGTGCGGTTTTAACA
TCAATTGATGAGCAGTCTGCTGATATCGCGATGGGCGTAGACCAAGCGCTTGAAGCCCGTGAAGG
CACAATGAGCGACGAAGAAATTGAAGCGATTGGTGCGGGTGACCAAGGATTAATGTTCGGTTATG
CGTGCAACGAAACGAAAGAGCTTATGCCTCTTCCAATTTCACTTGCCCATAAATTAGCCCGCCGC
CTAAGTGAAGTCCGTAAAGAAGATATTCTTCCGTACCTTCGCCCTGACGGCAAAACACAGGTAAC
GGTTGAGTACGATGAAAATAACAAACCAGTCCGCATTGACGCGATTGTTATTTCAACTCAGCATC
ACCCTGAAATTACACTTGAGCAAATTCAGCGCAACATTAAAGAACATGTAATCAATCCGGTTGTT
CCTGAAGAGCTGATTGATGAAGAAACAAAATATTTCATCAACCCTACAGGACGTTTCGTAATCGG
AGGCCCTCAAGGGGATGCGGGACTTACAGGACGCAAAATCATCGTTGATACGTACGGCGGCTATG
CACGCCACGGCGGAGGCGCGTTCTCAGGTAAGGACGCGACGAAGGTAGACCGTTCTGCAGCTTAT
GCGGCAAGATACGTTGCGAAAAACATCGTTGCGGCTGAGCTTGCTGATTCTTGCGAAGTACAGCT
TGCTTACGCGATCGGTGTTGCACAGCCTGTGTCAATCTCAATCAACACATTCGGTTCAGGAAAAG
CTTCTGAGGAAAAACTGATTGAAGTTGTTCGCAATAACTTTGATTTACGACCTGCCGGCATTATC
AAAATGCTTGATTTGCGCCGTCCGATCTATAAACAAACTGCTGCGTACGGCCACTTTGGACGTCA
CGATGTTGACCTTCCATGGGAGCGCACAGACAAAGCGGAGCAGCTGCGTAAAGAAGCGTTAGGAG
AATAA

MGSSHHHHHHSSGLVPRGSHMASMTGGQQMGRGSMSKNRRLFTSESVTEGHPDKICDQISDSILD
EILKKDPNARVACETSVTTGLVLVSGEITTSTYVDIPKTVRQTIKEIGYTRAKYGFDAETCAVLT
SIDEQSADIAMGVDQALEAREGTMSDEEIEAIGAGDQGLMFGYACNETKELMPLPISLAHKLARR
LSEVRKEDILPYLRPDGKTQVTVEYDENNKPVRIDAIVISTQHHPEITLEQIQRNIKEHVINPVV
PEELIDEETKYFINPTGRFVIGGPQGDAGLTGRKIIVDTYGGYARHGGGAFSGKDATKVDRSAAY
AARYVAKNIVAAELADSCEVQLAYAIGVAQPVSISINTFGSGKASEEKLIEVVRNNFDLRPAGII
KMLDLRRPIYKQTAAYGHFGRHDVDLPWERTDKAEQLRKEALGE

DNA and amino acid sequence of *Bs*MAT I317A[8]:

ATGGGCAGCAGCCATCATCATCATCATCACAGCAGCGGCCTGGTGCCGCGCGGCAGCCATATGGC
TAGCATGACTGGTGGACAGCAAATGGGTCGCGGATCCATGAGTAAAAATCGTCGTTTATTTACAT

CAGAATCTGTTACGGAGGGGCATCCGGATAAAATCTGTGACCAGATTTCTGACAGCATTTTAGAT
GAAATTTTAAAGAAAGACCCTAACGCGCGTGTTGCTTGTGAAACATCTGTGACAACAGGTTTGGT
TCTTGTAAGCGGAGAGATCACAACTTCTACGTATGTTGACATTCCGAAAACGGTTCGCCAAACCA
TTAAAGAAATCGGATACACACGTGCAAAATACGGATTTGATGCGGAAACTTGTGCGGTTTTAACA
TCAATTGATGAGCAGTCTGCTGATATCGCGATGGGCGTAGACCAAGCGCTTGAAGCCCGTGAAGG
CACAATGAGCGACGAAGAAATTGAAGCGATTGGTGCGGGTGACCAAGGATTAATGTTCGGTTATG
CGTGCAACGAAACGAAAGAGCTTATGCCTCTTCCAATTTCACTTGCCCATAAATTAGCCCGCCGC
CTAAGTGAAGTCCGTAAAGAAGATATTCTTCCGTACCTTCGCCCTGACGGCAAAACACAGGTAAC
GGTTGAGTACGATGAAAATAACAAACCAGTCCGCATTGACGCGATTGTTATTTCAACTCAGCATC
ACCCTGAAATTACACTTGAGCAAATTCAGCGCAACATTAAAGAACATGTAATCAATCCGGTTGTT
CCTGAAGAGCTGATTGATGAAGAAACAAAATATTTCATCAACCCTACAGGACGTTTCGTAATCGG
AGGCCCTCAAGGGGATGCGGGACTTACAGGACGCAAAATCATCGTTGATACGTACGGCGGCTATG
CACGCCACGGCGGAGGCGCGTTCTCAGGTAAGGACGCGACGAAGGTAGACCGTTCTGCAGCTTAT
GCGGCAAGATACGTTGCGAAAAACATCGTTGCGGCTGAGCTTGCTGATTCTTGCGAAGTACAGCT
TGCTTACGCGGCAGGTGTTGCACAGCCTGTGTCAATCTCAATCAACACATTCGGTTCAGGAAAAG
CTTCTGAGGAAAAACTGATTGAAGTTGTTCGCAATAACTTTGATTTACGACCTGCCGGCATTATC
AAAATGCTTGATTTGCGCCGTCCGATCTATAAACAAACTGCTGCGTACGGCCACTTTGGACGTCA
CGATGTTGACCTTCCATGGGAGCGCACAGACAAAGCGGAGCAGCTGCGTAAAGAAGCGTTAGGAG
AATAA

MGSSHHHHHHSSGLVPRGSHMASMTGGQQMGRGSMSKNRRLFTSESVTEGHPDKICDQISDSILD
EILKKDPNARVACETSVTTGLVLVSGEITTSTYVDIPKTVRQTIKEIGYTRAKYGFDAETCAVLT
SIDEQSADIAMGVDQALEAREGTMSDEEIEAIGAGDQGLMFGYACNETKELMPLPISLAHKLARR
LSEVRKEDILPYLRPDGKTQVTVEYDENNKPVRIDAIVISTQHHPEITLEQIQRNIKEHVINPVV
PEELIDEETKYFINPTGRFVIGGPQGDAGLTGRKIIVDTYGGYARHGGGAFSGKDATKVDRSAAY
AARYVAKNIVAAELADSCEVQLAYAAGVAQPVSISINTFGSGKASEEKLIEVVRNNFDLRPAGII
KMLDLRRPIYKQTAAYGHFGRHDVDLPWERTDKAEQLRKEALGE

DNA and amino acid sequence of *Ec*MTAN:

ATGGGCAGCAGCCATCATCATCATCATCACAGCAGCGGCCTGGTGCCGCGCGGCAGCCATATGGC
TAGCATGACTGGTGGACAGCAAATGGGTCGCGGATCCATGAAAATCGGCATCATTGGTGCAATGG
AAGAAGAAGTTACGCTGCTGCGTGACAAAATCGAAAACCGTCAAACTATCAGTCTCGGCGGTTGC
GAAATCTATACCGGCCAACTGAATGGAACCGAGGTTGCGCTTCTGAAATCGGGCATCGGTAAAGT
CGCTGCGGCGCTGGGTGCCACTCTGCTGTTGGAACACTGCAAGCCAGATGTGATTATTAACACCG
GTTCTGCCGGTGGCCTGGCACCAACGTTGAAAGTGGGCGATATCGTTGTCTCGGACGAAGCACGT
TATCACGACGCGGATGTCACGGCATTTGGTTATGAATACGGTCAGTTACCAGGCTGTCCGGCAGG
CTTTAAAGCTGACGATAAACTGATCGCTGCCGCTGAGGCCTGCATTGCCGAACTGAATCTTAACG
CTGTACGTGGCCTGATTGTTAGCGGCGACGCTTTCATCAACGGTTCTGTTGGTCTGGCGAAAATC
CGCCACAACTTCCCACAGGCCATTGCTGTAGAGATGGAAGCGACGGCAATCGCCCATGTCTGCCA
CAATTTCAACGTCCCGTTTGTCGTAGTACGCGCCATCTCCGACGTGGCCGATCAACAGTCTCATC
TTAGCTTCGATGAGTTCCTGGCTGTTGCCGCTAAACAGTCCAGCCTGATGGTTGAGTCACTGGTG
CAGAAACTTGCACATGGCTAA

MGSSHHHHHHSSGLVPRGSHMASMTGGQQMGRGSMKIGIIGAMEEEVTLLRDKIENRQTISLGGC
EIYTGQLNGTEVALLKSGIGKVAAALGATLLLEHCKPDVIINTGSAGGLAPTLKVGDIVVSDEAR
YHDADVTAFGYEYGQLPGCPAGFKADDKLIAAAEACIAELNLNAVRGLIVSGDAFINGSVGLAKI
RHNFPQAIAVEMEATAIAHVCHNFNVPFVVVRAISDVADQQSHLSFDEFLAVAAKQSSLMVESLV
QKLAHG

DNA and amino acid sequence of *Pa*HMT:

ATGGGCAGCAGCCATCATCATCATCATCACAGCAGCGGCCTGGTGCCGCGCGGCAGCCATATGGC
TAGCATGACTGGTGGACAGCAAATGGGTCGCGGATCCATGCAGGCGGATTTTTGGCACGCCCGCT
GGGCGAACAACCAGATCGGCTTCCACCTGGACGAGATCAATCCCTACCTGATGCGCCACCTGTCG
CGGCTGCGACTGCAAGCGGGCGAACAGATCCTGGTGCCGTTGTGCGGCAAGACCCTGGACCTGGC
CTGGCTGGCCGCCCAGGGACTGGAGGTGCTGGGGGTGGAGCTTTCGGAAAAGGCCGTGAGCGACT
TCTTCGAGGAGCACGACCTGCACCCCGAGATCGATCAACTGGATGGTTTCCGCCGCTACCGGGTC
GCCGGCATCACCCTGCTGCAGGGTGATTTCTTCGCCTTGCAGGCAGAGCACCTGGCGCAGTGCAG
GGCGTTCTACGACCGCGCCGCGCTGATCGCCCTGCCGCCGGAGATGCGCGAGCGCTATGCCGGGC

ATCTCCAGGCGGTCCTGCCGACGCGCAGCCTCGGCTTGCTGGTCACCATCGACTACCCGCAGGCG
GAGATGGCCGGTCCGCCGTTCGCCGTGCCCGACGAGGAGGTGCGTGGCTACTACGCTGGCGGTTG
GCGGATCGAGGAACTGGAGCGCGGCGACGTGCTCGGCGTCAACTGGAAATTCCTCGAGCGCGGGG
TGTCCTGGCTGGACGAGGCCGTCTACCTGCTGGAGAGAGGCTGA

MGSSHHHHHHSSGLVPRGSHMASMTGGQQMGRGSMQADFWHARWANNQIGFHLDEINPYLMRHLS
RLRLQAGEQILVPLCGKTLDLAWLAAQGLEVLGVELSEKAVSDFFEEHDLHPEIDQLDGFRRYRV
AGITLLQGDFFALQAEHLAQCRAFYDRAALIALPPEMRERYAGHLQAVLPTRSLGLLVTIDYPQA
EMAGPPFAVPDEEVRGYYAGGWRIEELERGDVLGVNWKFLERGVSWLDEAVYLLERG

DNA and amino acid sequence of *Pe*HMT:

ATGGGCAGCAGCCATCATCATCATCATCACAGCAGCGGCCTGGTGCCGCGCGGCAGCCATATGGC
TAGCATGACTGGTGGACAGCAAATGGGTCGCGGATCCATGGAGCCAGCGTTCTGGCACAAGCGGT
GGGCGGACAACCAGATCGGCTTTCACCAACTCCAGGTGAACCCGTACCTGCAGGCGCACTGGCCG
GCGTTGGGGCTGGCCCCCGGCGCGCGGGTGCTGGTGCCGTTGTGCGGCAAGAGCCTGGACATGCT
CTGGCTGGCCGCGCAGGGGTATCGGGTGTTGGGCGTGGAGTTGTCGCGGCGCGCCGTGGAGGATT
TCTTCACCGAGCATGGGCTGCCGGCGCAGGTGACGCAGCACGGCGCGTTCGAGGCCTGGCGCAGC
GATGAGGTGGAGATCTGGTGTGGGGATGTGTTTGCCTTGCGTGCCGAGGATCTTGCCGATTGCGC
GGGGGTGTATGACCGGGCGGCCTTGATTGCGCTGCCGCCTGAGATGCGCGAGCGCTACATGGCGT
TGCTGGGCGCGAAGTTGCCGACGGCGTGTCGCGGGGTGCTGGTGACCCTGGATTACGACCAGGCG
TTGATTGACGGGCCGCCGTTCTCGGTGCCGGATGCCGAAGTGCGTGCAGGGTTTTCCGGGTGGCA
GGTCGACGAGGTCGAGGGCCTGGAGATTCTCGAGGATAGCCCGAAGTTCATCAAGGCTGGGGTGT
CGAGCCTGGTCGAGCGGGCCTACCGCCTGACTCGGTAG

MGSSHHHHHHSSGLVPRGSHMASMTGGQQMGRGSMEPAFWHKRWADNQIGFHQLQVNPYLQAHWP
ALGLAPGARVLVPLCGKSLDMLWLAAQGYRVLGVELSRRAVEDFFTEHGLPAQVTQHGAFEAWRS
DEVEIWCGDVFALRAEDLADCAGVYDRAALIALPPEMRERYMALLGAKLPTACRGVLVTLDYDQA
LIDGPPFSVPDAEVRAGFSGWQVDEVEGLEILEDSPKFIKAGVSSLVERAYRLTR

DNA and amino acid sequence of *Pf*HMT:

ATGGGCAGCAGCCATCATCATCATCATCACAGCAGCGGCCTGGTGCCGCGCGGCAGCCATATGGC
TAGCATGACTGGTGGACAGCAAATGGGTCGCGGATCCATGCAGCCGGAGTTTTGGCACAAGAAGT
GGGAATCGAACCAGATCGGCTTTCACCAGCTTGAGGTGAACCCGTATTTGCAGCGGCACTGGCCC
GATCTGGCCATCCCGGTGCAGGCGCGTGTGTTGGTGCCGTTGTGCGGTAAAAGTCTGGATCTGCT
GTGGCTCGCCGGCCGTGGTCATCAGGTGCTGGGCGTCGAGCTTTCGGAAAAGGCAGTGGAAGACT
TTTTCCATGAGCAGCAATTGCAGCCGCAGGTGAGCGAGCAGGGCGACTTCAAGGTTTATCGCGCC
GACGCGGTCGAGTTGTGGTGCGGGGATTTCTTCTCGCTGACGATGGCTGACGTGGCGGGTTGCAC
CGCGTTGTATGACCGTGCGGCAGTGATTGCCTTGCCGCCAGCGATGCGCGAGCGTTATGCGGCGC
ATCTGCAGAGCATTCTGCCGGCGTGTCGAGGGCTGTTGGTGACGCTGGATTATGACCAGTCGCAG
ATGCCAGGGCCGCCATTTTCCGTAGACGATGCCGAAGTGCAGCGTTTGCTGGGCAGTGTCTGGCG
CGTCGAGATGCTGGAGCAGCAGGATGTACTCGGTGACAGCTGGAAGTTCGTGCAGGCCGGCGTAA
CCCGGCTTGAGGAACGGGTTTACCGGATTCGCGGGGTGTAA

MGSSHHHHHHSSGLVPRGSHMASMTGGQQMGRGSMQPEFWHKKWESNQIGFHQLEVNPYLQRHWP
DLAIPVQARVLVPLCGKSLDLLWLAGRGHQVLGVELSEKAVEDFFHEQQLQPQVSEQGDFKVYRA
DAVELWCGDFFSLTMADVAGCTALYDRAAVIALPPAMRERYAAHLQSILPACRGLLVTLDYDQSQ
MPGPPFSVDDAEVQRLLGSVWRVEMLEQQDVLGDSWKFVQAGVTRLEERVYRIRGV

DNA and amino acid sequence of *Pp*HMT:

ATGGGCAGCAGCCATCATCATCATCATCACAGCAGCGGCCTGGTGCCGCGCGGCAGCCATATGGC
TAGCATGACTGGTGGACAGCAAATGGGTCGCGGATCCATGGAACCAGCGTTCTGGCAGCAGCGGT
GGGCCGACAACCAGATCGGCTTTCACCAAGCGCAGGTGAACCCCTATCTGCAGACGTATTGGCCA
CAGTTGCAGTTGGCGCCCGGCAGCCGCGTGCTGGTGCCGTTGTGCGGCAAAAGCCTGGACTTGGC
CTGGCTGGCAGGGCAGGGCCATCGCGTATTGGGTGTGGAGCTGTCGCGGCGGGCGGTGGAGGATT
TTTTCCGTGAGCACGGGCTTGAGGCCGAGGTGCGGCAGCAGGGCGCATTTGAGGTTTGGCGCAGT
GGAGATGTGCAGCTGTGGTGTGGCGACTTCTTTGCCTTGCGGGCAGAGGATGTGGCTGACTGCGT
AGGGCTGTATGACCGGGCGGCGGTGATTGCGCTGCCGGTGCAGATGCGTGCGCGGTATATGCAGC
TGTTGTCGGGTTTGCTGCCGGCGAATTGCCGTGGGTTGGTGGTGACGCTGGAGTATGACCAGTCG
TTGTTGGCCGGGCCGCCGTTTTCGGTCAGGGATGAAGAGCTGAGGCAGGGCTTTGCGGGGTGGCA

GGTGGAGCAACTGGAGGCTGTGGATGTGATTGAGGACAGCCCGAAGTTTGTGCAGGCCGGGGCGT
CGAGTTTGTTGGAGCGGGTGTATCAGGTCAGCCGATAA

MGSSHHHHHHSSGLVPRGSHMASMTGGQQMGRGSMEPAFWQQRWADNQIGFHQAQVNPYLQTYWP
QLQLAPGSRVLVPLCGKSLDLAWLAGQGHRVLGVELSRRAVEDFFREHGLEAEVRQQGAFEVWRS
GDVQLWCGDFFALRAEDVADCVGLYDRAAVIALPVQMRARYMQLLSGLLPANCRGLVVTLEYDQS
LLAGPPFSVRDEELRQGFAGWQVEQLEAVDVIEDSPKFVQAGASSLLERVYQVSR

DNA and amino acid sequence of *Ps*HMT:

ATGGGCAGCAGCCATCATCATCATCATCACAGCAGCGGCCTGGTGCCGCGCGGCAGCCATATGAA
GGCAGATTTTTGGCTGCAGCGCTGGAGCGCAGGTCAGATTGGCTTTCATCAGAGCGAAGTTAATA
AGGATCTGCAGCAGTATTGGAGCAGTCTGAATGTTGTTCCGGGTGCCCGTGTTCTGGTGCCGCTG
TGCGGCAAAAGTCAGGATATGAGCTGGCTGAGTGGCCAGGGCTATCATGTTGTTGGCGCAGAACT
GAGTGAAGCCGCAGTGGAACGTTATTTTACCGAACGTGGCGAACAGCCGCATATTACCAGCCAGG
GCGATTTTAAAGTTTATGCAGCCCCGGGTATTGAAATTTGGTGCGGCGATTTCTTTGCACTGACC
GCCCGTGATATTGGTCATTGCGCCGCCTTTTATGATCGCGCAGCAATGATTGCACTGCCGGCCGA
TATGCGTGAACGCTATGTTCAGCATCTGGAAGCCCTGATGCCGCAGGCATGTAGTGGTCTGCTGA
TTACCCTGGAATATGATCAGGCCCTGCTGGAAGGCCCGCCGTTTAGTGTTCCGCAGACCTGGCTG
CATCGTGTTATGAGCGGCAATTGGGAAGTTACCAAAGTTGGTGGCCAGGATACCCTGCATAGTAG
TGCACGTGGTCTGAAAGCAGGCCTGGAACGTATGGATGAACATGTTTATGTTCTGGAACGTGTTT
AA

MGSSHHHHHHSSGLVPRGSHMKADFWLQRWSAGQIGFHQSEVNKDLQQYWSSLNVVPGARVLVPL
CGKSQDMSWLSGQGYHVVGAELSEAAVERYFTERGEQPHITSQGDFKVYAAPGIEIWCGDFFALT
ARDIGHCAAFYDRAAMIALPADMRERYVQHLEALMPQACSGLLITLEYDQALLEGPPFSVPQTWL
HRVMSGNWEVTKVGGQDTLHSSARGLKAGLERMDEHVYVLERV

DNA and amino acid sequence of *Ct*HMT:

ATGGGCAGCAGCCATCATCATCATCATCACAGCAGCGGCCTGGTGCCGCGCGGCAGCCATATGCT
GGGTATGGATGCCGATACCGCCAGCTTTTGGGAAGAAAAATATCGTGCAGATCTGACCGCCTGGG

ATCGCGGTGGTGTTAGTCCGGCCCTGGAACATTGGCTGGCAGAAGGTGCACTGAAACCGGGCCGT
ATTCTGATTCCGGGCTGCGGTTATGGCCATGAAGTGCTGGCACTGGCACGCCGCGGCTTTGAAGT
GTGGGGCCTGGATATTGCACTGACCCCGGTTCGTCGTCTGCAGGAAAAACTGGCCCAGGCAGGCC
TGACCGCACATGTTGTTGAAGGTGACGTTCGTACCTGGCAGCCGGAACAGCCGTTTGATGCAGTT
TATGAACAGACCTGTCTGTGTGCACTGAGTCCGGAAGATTGGCCGCGTTATGAAGCACAGCTGTG
CCGTTGGCTGCGTCCGGGTGGTCGCCTGTTTGCACTGTGGATGCAGACCGATCGTCCGGGTGGCC
CGCCGTATCATTGTGGTCTGGAAGCAATGCGTGTGCTGTTTGCATTAGAACGCTGGCGCTGGGTG
GAACCGCCGCAGAGAACCGTTCCGCATCCGACCGGTTTCTTTGAATATGCCGCAATTCTGGAACG
CCTGGTGTAA

MGSSHHHHHHSSGLVPRGSHMLGMDADTASFWEEKYRADLTAWDRGGVSPALEHWLAEGALKPGR
ILIPGCGYGHEVLALARRGFEVWGLDIALTPVRRLQEKLAQAGLTAHVVEGDVRTWQPEQPFDAV
YEQTCLCALSPEDWPRYEAQLCRWLRPGGRLFALWMQTDRPGGPPYHCGLEAMRVLFALERWRWV
EPPQRTVPHPTGFFEYAAILERLV

DNA and amino acid sequence of *Bx*HMT:

ATGGGCAGCAGCCATCATCATCATCATCACAGCAGCGGCCTGGTGCCGCGCGGCAGCCATATGAG
TGATCCGACCCAGCCGGCCGTGCCGGATTTTGAAACCCGCGATCCGAATAGCCCGGCCTTTTGGG
ATGAACGCTTTGAACGCCGTTTTACCCCGTGGGATCAGGCAGGTGTTCCGGCAGCCTTTCAGAGC
TTTGCCGCACGCCATAGCGGCGCAGCAGTTCTGATTCCGGGTTGCGGCAGTGCATACGAAGCAGT
GTGGCTGGCAGGTCAGGGTAATCCGGTTCGTGCAATTGATTTTAGCCCGGCCGCAGTGGCCGCAG
CACATGAACAGCTGGGTGCCCAGCATGCACAGCTGGTTGAACAGGCAGATTTCTTTACCTATGAA
CCGCCGTTTACCCCGGCATGGATCTATGAACGTGCATTTCTGTGCGCCCTGCCGCTGGCACGCCG
TGCAGATTATGCACATCGCATGGCAGATCTGCTGCCGGGTGGCGCACTGCTGGCCGGTTTCTTTT
TCCTGGGCGCAACCCCGAAAGGCCCGCCGTTTGGTATTGAACGTGCAGAACTGGATGCACTGCTG
ACCCCGTATTTTGATCTGATTGAAGATGAAGCAGTTCATGATAGTATTGCCGTGTTTGCCGGCCG
TGAACGCTGGCTGACCTGGCGTCGTCGTGCCTAA

MGSSHHHHHHSSGLVPRGSHMSDPTQPAVPDFETRDPNSPAFWDERFERRFTPWDQAGVPAAFQS
FAARHSGAAVLIPGCGSAYEAVWLAGQGNPVRAIDFSPAAVAAAHEQLGAQHAQLVEQADFFTYE

PPFTPAWIYERAFLCALPLARRADYAHRMADLLPGGALLAGFFFLGATPKGPPFGIERAELDALL
TPYFDLIEDEAVHDSIAVFAGRERWLTWRRRA

DNA and amino acid sequence of *At*HMT:

ATGGGCAGCAGCCATCATCATCATCATCACAGCAGCGGCCTGGTGCCGCGCGGCAGCCATATGGC
CGAAGAACAGCAGAATAGTGATCAGAGTAATGGCGGTAATGTGATTCCGACCCCGGAAGAAGTTG
CAACCTTTCTGCATAAAACCGTTGAAGAAGGCGGTTGGGAAAAATGCTGGGAAGAAGAAATTACC
CCGTGGGATCAGGGTCGTGCAACCCCGCTGATTGTGCATCTGGTGGATACCAGCAGCCTGCCGCT
GGGTCGCGCCTTAGTGCCGGGTTGCGGTGGTGGTCATGATGTGGTTGCCATGGCCAGTCCGGAAC
GCTTTGTTGTTGGTCTGGATATTAGTGAAAGCGCCCTGGCAAAAGCCAATGAAACCTATGGTAGC
AGTCCGAAAGCAGAATATTTTAGCTTTGTTAAAGAGGATGTGTTCACCTGGCGTCCGACCGAACT
GTTTGATCTGATTTTTGATTATGTGTTCTTCTGTGCAATCGAACCGGAAATGCGCCCGGCATGGG
CAAAAAGTATGTATGAACTGCTGAAACCGGATGGCGAACTGATTACCCTGATGTATCCGATTACC
GATCATGTTGGTGGCCCGCCGTATAAAGTTGATGTTAGCACCTTTGAAGAAGTGCTGGTGCCGAT
TGGCTTTAAAGCCGTGAGCGTGGAAGAAAATCCGCATGCCATTCCGACCCGTAAAGGTAAAGAAA
AACTGGGCCGCTGGAAAAAGATTAATTAA

MGSSHHHHHHSSGLVPRGSHMAEEQQNSDQSNGGNVIPTPEEVATFLHKTVEEGGWEKCWEEEIT
PWDQGRATPLIVHLVDTSSLPLGRALVPGCGGGHDVVAMASPERFVVGLDISESALAKANETYGS
SPKAEYFSFVKEDVFTWRPTELFDLIFDYVFFCAIEPEMRPAWAKSMYELLKPDGELITLMYPIT
DHVGGPPYKVDVSTFEEVLVPIGFKAVSVEENPHAIPTRKGKEKLGRWKKIN

DNA and amino acid sequence of *acl*-MT:

ATGGGCAGCAGCCATCATCATCATCATCACAGCAGCGGCCTGGTGCCGCGCGGCAGCCATATGTC
AACGCCCAGTTTAATTCCATCCGGTGTGCACGAAGTATTAGCAAAATACAAAGACGGTAACTATG
TCGACGGGTGGGCCGAACTGTGGGATAAATCTAAGGGTGACCGTCTTCCATGGGATCGTGGTTTT
CCGAACCCTGCGTTGGAAGATACCCTGATTCAGAAACGTGCCATCATCGGAGGTCCCTTGGGCCA
AGACGCTCAGGGCAAGACATACCGCAAAAAAGCTCTGGTCCCAGGCTGCGGTCGTGGAGTAGATG
TGTTGTTATTGGCATCGTTCGGTTATGACGCTTATGGTTTAGAATACTCTGCAACGGCGGTCGAT
GTATGCCAAGAGGAGCAAGCCAAGAACGGGGATCAATATCCTGTACGCGATGCAGAAATCGGCCA

GGGCAAGATTACGTTCGTTCAAGGTGATTTCTTTGAGGACACGTGGCTTGAAAAGCTTAACCTTA
CCCGCAATTGTTTCGACGTTATCTACGACTACACGTTCTTCTGTGCATTGAACCCGAGCATGCGC
CCTCAGTGGGCTTTGCGCCATACCCAACTTCTTGCCGACTCTCCGCGCGGACATTTAATCTGTTT
GGAGTTTCCCCGCCATAAAGACCCTTCTGTACAGGGGCCACCATGGGGCTCGGCGTCAGAAGCCT
ACCGCGCCCATCTGTCACATCCAGGGGAGGAAATTCCCTACGATGCGTCACGTCAATGCCAGTTT
GACAGCAGCAAAGCACCCAGTGCGCAGGGCTTGGAACGTGTAGCATATTGGCAACCAGAGCGCAC
GCATGAAGTAGGGAAAAACGAGAAAGGTGAGGTTCAAGATCGCGTATCCATCTGGCAGCGTCCTC
CCCAAAGTTCCCTGTAA

MGSSHHHHHHSSGLVPRGSHMSTPSLIPSGVHEVLAKYKDGNYVDGWAELWDKSKGDRLPWDRGF
PNPALEDTLIQKRAIIGGPLGQDAQGKTYRKKALVPGCGRGVDVLLLASFGYDAYGLEYSATAVD
VCQEEQAKNGDQYPVRDAEIGQGKITFVQGDFFEDTWLEKLNLTRNCFDVIYDYTFFCALNPSMR
PQWALRHTQLLADSPRGHLICLEFPRHKDPSVQGPPWGSASEAYRAHLSHPGEEIPYDASRQCQF
DSSKAPSAQGLERVAYWQPERTHEVGKNEKGEVQDRVSIWQRPPQSSL

DNA and amino acid sequence of YiaE:

ATGGGCAGCAGCCATCATCATCATCATCACAGCAGCGGCCTGGTGCCGCGCGGCAGCCATATGGC
TAGCATGACTGGTGGACAGCAAATGGGTCGCGGATCCATGAAGCCGTCCGTTATCCTCTACAAAG
CCTTACCTGATGATTTACTGCAACGCCTGCAAGAGCATTTCACCGTTCACCAGGTGGCAAACCTC
AGCCCACAAACCGTCGAACAAAATGCAGCAATTTTTGCCGAAGCTGAAGGTTTACTGGGTTCAAA
CGAGAATGTTGATGCCGCATTGCTGGAAAAAATGCCGAAACTGCGTGCCACATCAACGATCTCCG
TCGGCTATGACAATTTTGATGTCGATGCGCTTACCGCCCGAAAAATTCTGCTGATGCACACGCCA
ACCGTATTAACAGAAACCGTCGCCGATACGCTGATGGCGCTGGTGTTGTCTACCGCTCGTCGGGT
TGTGGAGGTAGCAGAACGGGTAAAAGCAGGCGAATGGACCGCGAGCATAGGCCCGGACTGGTACG
GCACTGACGTTCACCATAAAACACTGGGCATTGTCGGGATGGGACGGATCGGTATGGCGCTGGCA
CAACGTGCGCACTTTGGCTTCAACATGCCCATCCTCTATAACGCGCGCCGCCACCATAAAGAAGC
AGAAGAACGCTTCAACGCCCGCTACTGCGATTTGGATACTCTGTTACAAGAGTCAGATTTCGTTT
GCCTGATCCTGCCGTTAACTGATGAGACGCATCATCTGTTTGGCGCAGAACAATTCGCCAAAATG
AAATCCTCCGCCATTTTCATTAATGCCGGACGTGGCCCGGTGGTTGATGAAAATGCACTGATTGC
TGCATTGCAGAAAGGCGAAATTCACGCCGCCGGGCTGGATGTCTTCGAACAAGAGCCACTGTCCG

TAGATTCGCCGTTGCTCTCAATGGCCAACGTCGTCGCAGTACCGCATATTGGATCTGCCACCCAT
GAGACGCGTTATGGCATGGCCGCCTGTGCCGTGGATAATTTGATTGATGCGTTACAAGGAAAGGT
TGAGAAGAACTGTGTGAATCCGCACGTCGCGGACTAA

MGSSHHHHHHSSGLVPRGSHMASMTGGQQMGRGSMKPSVILYKALPDDLLQRLQEHFTVHQVANL
SPQTVEQNAAIFAEAEGLLGSNENVDAALLEKMPKLRATSTISVGYDNFDVDALTARKILLMHTP
TVLTETVADTLMALVLSTARRVVEVAERVKAGEWTASIGPDWYGTDVHHKTLGIVGMGRIGMALA
QRAHFGFNMPILYNARRHHKEAEERFNARYCDLDTLLQESDFVCLILPLTDETHHLFGAEQFAKM
KSSAIFINAGRGPVVDENALIAALQKGEIHAAGLDVFEQEPLSVDSPLLSMANVVAVPHIGSATH
ETRYGMAACAVDNLIDALQGKVEKNCVNPHVAD

DNA and amino acid sequence of *Km*AT:

ATGGGCAGCAGCCATCATCATCATCATCACAGCAGCGGCCTGGTGCCGCGCGGCAGCCATATGCT
GGAGAAACATCGCGTGTTTCAGAAAGTTGACGCCTACGCTGGCGACCCCATTCTCTCCCTGATGG
AGCGTTTTAAAGACGATCCGCGTAGTGACAAAGTAAATCTCAGCATCGGTCTTTATTACAACGAA
GACGGCATCATTCCGCAGCTGAAGGCGGTTGCCGAAGCGGAAGCACGTCTTAACGCCCAGCCGCA
CGGCGCCTCAATCTATTTACCGATGGAAGGGCTGAACACCTATCGCCACGCAATTGCGTCGCTGC
TGTTCGGGGCGGATCATCCGGTACTGGCGCAGCATCGCGTGGCCTCTATTCAGACGCTGGGCGGC
TCCGGAGCCCTGAAAGTCGGCGCTGACTTCCTGAAACGCTACTTCCCAGGCTCGCGCGTATGGGT
CAGCGACCCGACGTGGGAAAACCACATCGCTATATTTGAAGGGGCTGGATTCGAAGTAAGTACTT
ACCCTTGGTTTGATGATAAAACCAATGGCGTGCGTTTCGCGGCTTTCCTTGAAAAACTCAATACG
CTACCGGAACGCGATATCGTGCTACTGCATCCCTGCTGCCATAACCCAACGGGAGCCGATCTTAC
CAACGCTCAGTGGGATCAAGTGGTTGAGGTGCTGAAAGCGCGTAATTTGATTCCTTTCCTCGACA
TCGCCTATCAGGGCTTTGGCGCCGGCATGGAAGAGGATGCCTACGCTATCCGCGCCATAGCGAAC
GCCGGGATGCCCATGCTGGTGAGCAACTCGTTCTCGAAAATCTTCTCCCTGTACGGTGAGCGCGT
TGGCGGGCTGTCGATCGTTTGTGAAGATACTGAAACCGCGGGACGCGTACTGGGGCAATTAAAAG
CGACCGTACGTCGCAACTACTCCAGCCCGCCGAACTTTGGCGCGCAGGTGGTTGCGGCGGTGTTG
GGCGACAGCGCGCTGAAAGCCTCATGGCTGGCGGAAGTGGAAGGGATGCGCACCCGTATTCTGGC
CATGCGTCAGGAGCTGGTTGATGTGCTGAAAGAGACGGTTCCGGGCGGTGATTTTGACTATCTGC
TCAAGCAGCGCGGCATGTTTAGCTATACCGGTTTCAGCGCGGCCCAGGTTGACCGCCTGCGCGAT

GAGTTTGGCGTCTACCTGATTGCCAGCGGCCGCATGTGCGTCGCGGGGCTGAATGCAGGAAACGT
GCGCCGCGTAGCGCAGGCGTTTGCTGCGGTAATATAA

MGSSHHHHHHSSGLVPRGSHMLEKHRVFQKVDAYAGDPILSLMERFKDDPRSDKVNLSIGLYYNE
DGIIPQLKAVAEAEARLNAQPHGASIYLPMEGLNTYRHAIASLLFGADHPVLAQHRVASIQTLGG
SGALKVGADFLKRYFPGSRVWVSDPTWENHIAIFEGAGFEVSTYPWFDDKTNGVRFAAFLEKLNT
LPERDIVLLHPCCHNPTGADLTNAQWDQVVEVLKARNLIPFLDIAYQGFGAGMEEDAYAIRAIAN
AGMPMLVSNSFSKIFSLYGERVGGLSIVCEDTETAGRVLGQLKATVRRNYSSPPNFGAQVVAAVL
GDSALKASWLAEVEGMRTRILAMRQELVDVLKETVPGGDFDYLLKQRGMFSYTGFSAAQVDRLRD
EFGVYLIASGRMCVAGLNAGNVRRVAQAFAAVI

VII. Synthesis and characterization of substrates

General procedure for the synthesis of α-keto acid substrates 1

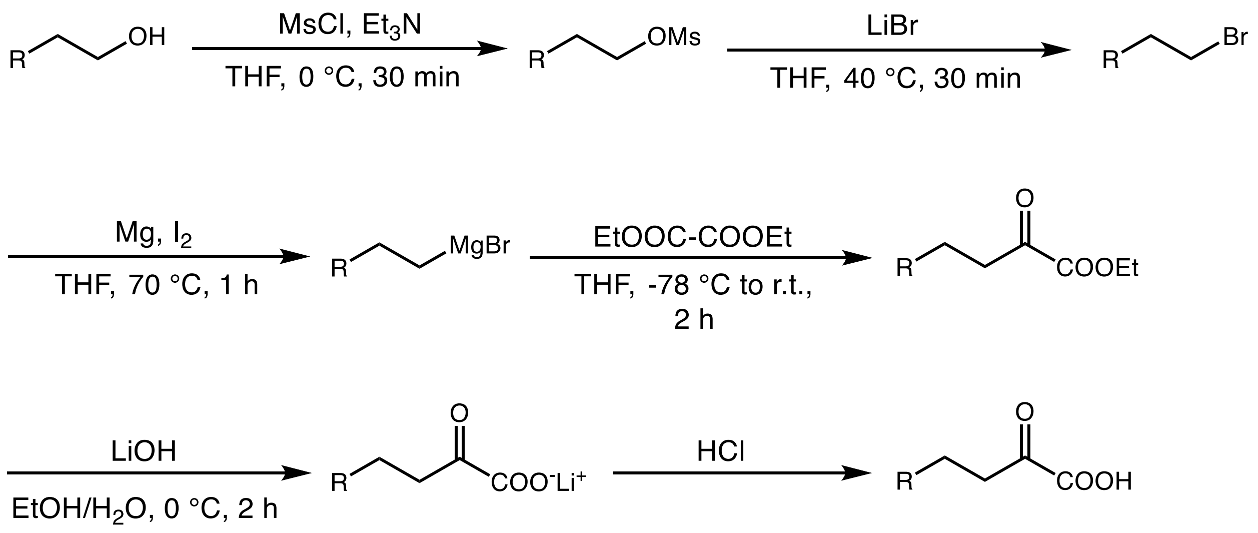

This procedure is modified from a published procedure[9].

At 0 °C, Et_3N (1.5 equiv) and methanesulfonyl chloride (1.2 equiv) were slowly added to a solution of the alcohol (1.0 equiv, 0.4 M in THF). The reaction mixture was allowed to stir at 0 °C until TLC analysis indicated complete conversion of the alcohol starting material and white precipitation formed (generally 30 min). Lithium bromide (8.0 equiv) was then added. The reaction mixture was stirred at 40 ℃ for 2 h, then diluted with H_2O and extracted with ethyl acetate (3×).

The organic layer was dried over Na_2SO_4 and concentrated. The crude alkyl bromide was purified by flash column chromatography with the aid of a Biotage Isolera.

At 70 °C, a solution of the alkyl bromide product (1.0 equiv, 0.4 M in anhydrous THF) was slowly added to a mixture of magnesium turnings (2.0 equiv) and I_2 (a few crystals) in anhydrous THF under N_2 atmosphere. The reaction mixture was stirred for 1 h. At -78 °C, the generated Grignard reagent was then slowly added to a solution of diethyl oxalate (0.9 equiv, 0.5 M in anhydrous THF) under N_2 atmosphere. After 1 h, the reaction mixture was allowed to stir at room temperature for another 1 h and then quenched with 1M HCl. The aqueous layer was extracted with ethyl acetate (3×). The organic layer was washed with brine, dried over Na_2SO_4 and concentrated *in vacuo* to afford the crude α-keto acid esters, which was purified by flash column chromatography with the aid of a Biotage Isolera.

At 0 ℃, a solution of α-keto acid ester (1.0 equiv, 2.0 M in EtOH) was added dropwise to a solution of lithium hydroxide (1.2 equiv, 0.5 M in H_2O). The reaction mixture was stirred for 30 minutes. The solid precipitated out was filtered on a Buchner funnel under suction and washed with chilled EtOH followed by diethyl ether to afford analytically pure lithium salt of the α-ketoacid as a white solid. This lithium salt can be used directly to prepare aqueous solutions of the substrate. If needed, this lithium salt can be converted to the corresponding α-keto acid. At 80 ℃, a saturated solution of salt was poured into a solution of HCl (1.0 M). The aqueous layer was extracted with ethyl acetate (3×). The organic layer was washed with brine, dried over Na_2SO_4 and concentrated *in vacuo* to afford the corresponding α-keto acid.

General procedure for the synthesis of L-Methionine analogues

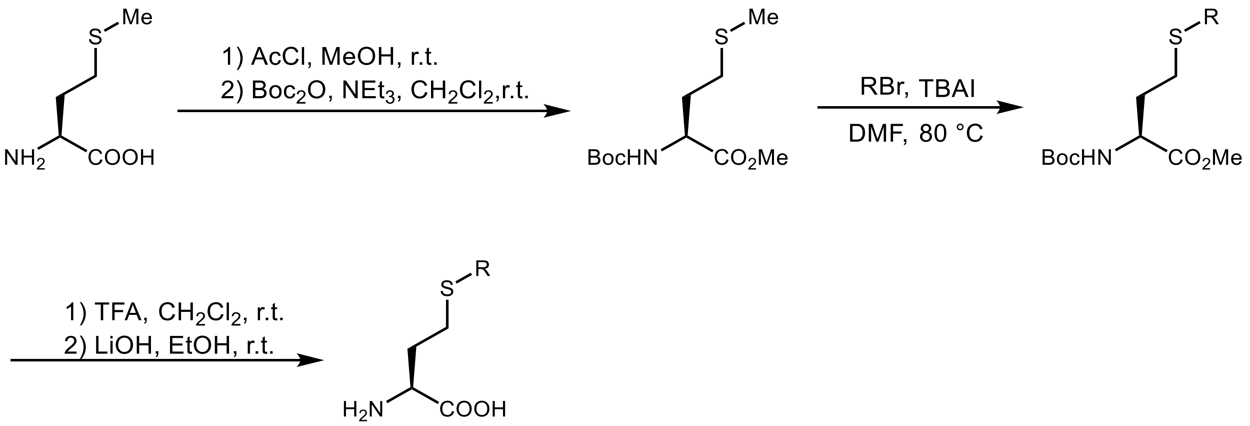

This procedure is modified from a published procedure[10].

At 0 ℃, acetyl chloride (11.9 mL, 167 mmol) was added dropwise to MeOH (30 mL). The solution was stirred at room temperature for 30 min. L-Methionine (10.0 g, 67 mmol) was then added. The reaction was allowed to warm to room temperature and stirred overnight. The solvent was removed with the aid of a rotary evaporator. The resulting yellow solid was directly used in the next step without purification. The crude ester was suspended in CH_2Cl_2 (150 mL), stirred and cooled to 0 °C. Et_3N (18.7 mL, 130 mmol) was added slowly. Boc_2O (17.5 g, 80 mmol) was added and the reaction mixture was stirred at room temperature for 5 h. Solvent was removed *in vacuo* with the aid of a rotary evaporator, and the crude *N*-Boc-*L*-Methionine methyl ester was purified by flash column chromatography with the aid of a Biotage Isolera to afford analytically pure product in 95% yield.

Tert-butylammonium iodide (TBAI, 7.0 g, 19 mmol), haloalkane (38 mmol) and *N*-Boc-L-Methionine methyl ester (1.0 g, 3.8 mmol) were added to DMF (50 mL). The reaction mixture was heated to 80 °C, stirred overnight, diluted with water and extracted with Et_2O (3×). The combined organic layer was washed with water and brine and dried over Na_2SO_4. Solvents were removed *in vacuo* with the aid of a rotary evaporator, and the crude methyl ester was purified by flash column chromatography with the aid of a Biotage Isolera to afford analytically pure product in 60 –70% yield.

The purified ester (3.5 mmol) was then dissolved in CH_2Cl_2 (10 mL), followed by the addition of trifluoroacetic acid (TFA, 7 mL). The reaction mixture was allowed to stir at room temperature for 2 h. Solvents were removed *in vacuo* with the aid of a rotary evaporator. The product was redissolved in EtOH (10 mL). At 0 °C, this solution was added slowly to an aqueous solution of lithium hydroxide (10 mL of a 5 M solution in H_2O). The reaction mixture was stirred at room temperature for 30 min. The pH of the reaction mixture was carefully adjusted to 2.0 – 4.0 by adding 1.0 M HCl (aq.). Organic solvents were then removed *in vacuo* with the aid of a rotary evaporator. The residue was dissolved in 10% MeOH/H_2O with 0.1% formic acid, and loaded onto a C18 column (12 g) that had been equilibrated to 10% MeOH/H_2O with 0.1% formic acid. The column was washed with 2 column volumes (CV) of 10% MeOH/H_2O with 0.1% formic acid. Then, the product was eluted with a gradient from 10% to 100% methanol with 0.1% formic acid over 8 CV. Product-containing fractions were combined and concentrated *in vacuo* with the aid of a rotary evaporator. The residual aqueous solution was lyophilized to give L-methionine analogues

as analytically pure products.

Characterization data for α-keto acid substrates

2-Oxo-4-phenylbutanoic acid (1a)

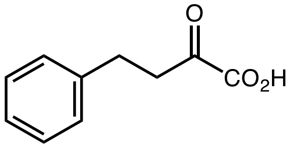

White solid. ^1H NMR (400 MHz, $CDCl_3$) δ: 8.81 (s, 1H), 7.38 – 7.27 (m, 2H), 7.26 – 7.17 (m, 3H), 3.28 (t, *J* = 7.4 Hz, 2H), 2.99 (t, *J* = 7.4 Hz, 2H) ppm. ^{13}C NMR (101 MHz, $CDCl_3$) δ: 194.9, 160.6, 139.8, 128.7, 128.4, 126.6, 39.7, 29.0 ppm. Spectral data match those previously reported[11].

2-Oxo-4-(*o*-tolyl)butanoic acid (1b)

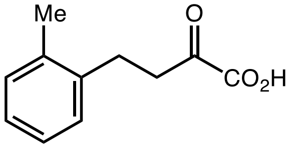

White solid. ^1H NMR (500 MHz, $CDCl_3$) δ: 8.55 (s, 1H), 7.24 – 7.04 (m, 4H), 3.24 (t, *J* = 7.6 Hz, 2H), 2.98 (t, *J* = 7.6 Hz, 2H), 2.34 (s, 3H) ppm. ^{13}C NMR (126 MHz, $CDCl_3$) δ: 194.9, 160.0, 137.7, 136.1, 130.6, 128.7, 126.9, 126.4, 38.1, 26.5, 19.4 ppm. IR: 3743, 2921, 2852, 1724, 1652, 1541, 1456, 1370, 1263, 1076 cm^{-1}. HRMS (ESI): calcd. for $C_{11}H_{11}O_3^-$ $[M-H]^-$: 191.0714; found: 191.0715.

2-Oxo-4-(*m*-tolyl)butanoic acid (1c)

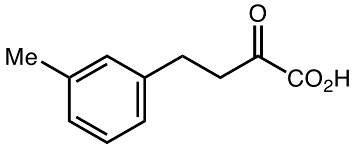

White solid. ^1H NMR (500 MHz, $CDCl_3$) δ: 7.19 (t, *J* = 7.5 Hz, 1H), 7.09 – 6.98 (m, 3H), 6.83 (s, 1H), 3.25 (t, *J* = 7.5 Hz, 2H), 2.94 (t, *J* = 7.5 Hz, 2H), 2.34 (s, 3H) ppm. ^{13}C NMR (126 MHz, $CDCl_3$) δ: 94.9, 160.6, 139.7, 138.4, 129.2, 128.6, 127.3, 125.4, 39.7, 28.9, 21.5 ppm. IR: 3744, 3448, 2922, 2852, 1724, 1653, 1542, 1457, 1260, 1076 cm^{-1}. HRMS (ESI): calcd. for $C_{11}H_{11}O_3^-$ $[M-H]^-$: 191.0714; found: 191.0714.

2-Oxo-4-(*p*-tolyl)butanoic acid (1d)

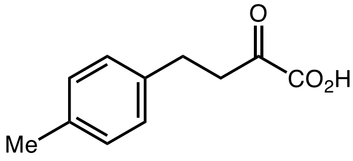

White solid. ^1H NMR (500 MHz, $CDCl_3$) δ: 7.13 – 7.07 (m, 4H), 5.82 (s, 1H), 3.25 (t, *J* = 7.5 Hz, 2H), 2.94 (t, *J* = 7.4 Hz, 2H), 2.32 (s, 3H) ppm. ^{13}C NMR (126 MHz, $CDCl_3$) δ: 195.1, 160.2, 136.7,

136.2, 129.4, 128.3, 39.6, 28.6, 21.1 ppm. Spectral data match those previously reported[9].

4-(4-Methoxyphenyl)-2-oxobutanoic acid (1e)

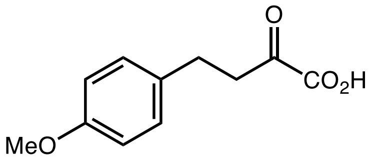

White solid. ^{1}H NMR (500 MHz, $CDCl_3$) δ: 8.73 (s, 1H), 7.12 (d, J = 8.5 Hz, 2H), 6.84 (d, J = 8.6 Hz, 2H), 3.79 (s, 3H), 3.24 (t, J = 7.4 Hz, 2H), 2.93 (t, J = 7.4 Hz, 2H) ppm. ^{13}C NMR (126 MHz, $CDCl_3$) δ: 194.9, 160.1, 158.2, 131.8, 129.4, 114.2, 55.4, 39.6, 28.2 ppm. Spectral data match those previously reported[9].

Lithium 4-(2-methoxyphenyl)-2-oxobutanoate (1f)

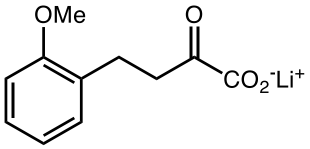

White solid. ^{1}H NMR (400 MHz, DMSO-d_6) δ: 7.25 – 7.07 (m, 2H), 6.92 (d, J = 8.2 Hz, 1H), 6.83 (t, J = 7.4 Hz, 1H), 3.77 (s, 3H), 2.84 – 2.60 (m, 4H) ppm. ^{13}C NMR (101 MHz, DMSO-d_6) δ: 206.0, 168.8, 157.0, 129.4, 129.3, 127.1, 120.2, 110.4, 55.2, 38.7, 23.6 ppm. IR: 3058, 2983, 2718, 1599, 1494, 1401, 1247, 1204, 1071, 1049 cm^{-1}. HRMS (ESI): calcd. for $C_{11}H_{11}LiO_4Na^+$ $[M+Na]^+$: 237.0715; found: 237.0718.

Lithium 2-oxo-4-(2-(trifluoromethyl)phenyl)butanoate (1g)

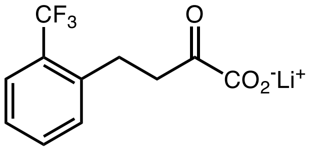

White solid. ^{1}H NMR (400 MHz, DMSO-d_6) δ: 7.78 – 7.25 (m, 4H), 3.15 – 2.60 (m, 4H) ppm. ^{13}C NMR (101 MHz, DMSO-d_6) δ: 204.8, 168.3, 140.2, 132.6, 131.1, 126.5, 126.9 (q, J = 28.9 Hz), 125.7 (q, J = 5.7 Hz), 124.7 (q, J = 272.1 Hz), 40.3, 25.5 ppm. ^{19}F NMR (376 MHz, DMSO-d_6) δ: -58.6 (s) ppm. IR: 3086, 2997, 2907, 2717, 1603, 1404, 1313, 1167, 1082, 1036 cm^{-1}. HRMS (ESI): calcd. for $C_{11}H_8F_3O_3LiNa^+$ $[M+Na]^+$: 275.0478; found: 275.0475.

Lithium 4-(4-fluorophenyl)-2-oxobutanoate (1h)

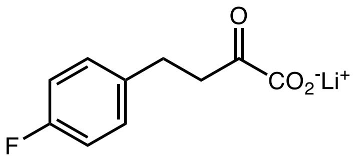

White solid. ^{1}H NMR (400 MHz, DMSO-d_6) δ: 7.29 – 7.18 (m, 2H), 7.11 – 7.00 (m, 2H), 2.84 – 2.68 (m, 4H) ppm. ^{13}C NMR (101 MHz, DMSO-d_6) δ: 205.6, 168.7, 160.6 (d, J = 240.9 Hz), 137.8 (d, J = 3.2 Hz), 130.0 (d, J = 7.9 Hz), 114.8 (d, J = 20.8 Hz), 40.4, 28.0 ppm.

^{19}F NMR (376 MHz, DMSO-d_6) δ: -117.76 – -117.86 (m, 1F) ppm. IR: 2992, 2932, 2711, 1595, 1512, 1418, 1242, 1198, 1136, 1084 cm^{-1}. HRMS (ESI): calcd. for $C_{10}H_8LiO_3Na$ [M+Na]$^+$: 225.0515; found: 225.0512.

4-(4-Chlorophenyl)-2-oxobutanoic acid (1i)

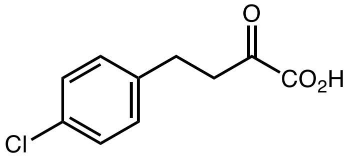

White solid. ^{1}H NMR (500 MHz, $CDCl_3$) δ: 7.21 – 7.14 (m, 2H), 7.10 – 6.99 (m, 2H), 5.82 (s, 1H), 3.15 (t, *J* = 7.4 Hz, 2H), 2.86 (t, *J* = 7.4 Hz, 2H) ppm. ^{13}C NMR (126 MHz, $CDCl_3$) δ: 194.7, 160.5, 138.2, 132.4, 129.8, 128.8, 39.5, 28.3 ppm. Spectral data match those previously reported[9].

4-(Benzo[*d*][1,3]dioxol-5-yl)-2-oxobutanoic acid (1j)

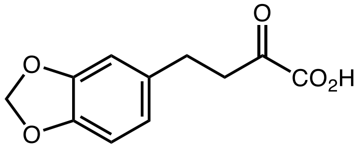

White solid. ^{1}H NMR (500 MHz, $CDCl_3$) δ: 6.73 (d, *J* = 7.9 Hz, 1H), 6.69 (d, *J* = 1.7 Hz, 1H), 6.66 – 6.63 (m, 1H), 5.93 (s, 2H), 3.24 (t, *J* = 7.4 Hz, 2H), 2.91 (t, *J* = 7.4 Hz, 2H) ppm. ^{13}C NMR (126 MHz, $CDCl_3$) δ: 195.0, 159.4, 147.9, 146.3, 133.4, 121.4, 109.0, 108.5, 101.1, 39.4, 28.9 ppm. Spectral data match those previously reported[9].

Lithium 2-oxo-4-(thiophen-2-yl)butanoate (1k)

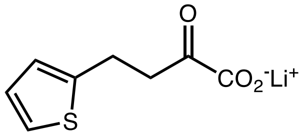

White solid. ^{1}H NMR (400 MHz, DMSO-d_6) δ: 7.27 (d, *J* = 5.1 Hz, 1H), 7.03 – 6.68 (m, 2H), 2.97 (t, *J* = 7.4 Hz, 2H), 2.84 (t, *J* = 7.3 Hz, 2H) ppm. ^{13}C NMR (101 MHz, DMSO-d_6) δ: 205.0, 168.4, 144.2, 126.8, 124.4, 123.5, 40.6, 23.1 ppm. IR: 3076, 2998, 2926, 2711, 1742, 1594, 1415, 1352, 1275, 1083 cm^{-1}. HRMS (ESI): calcd. for $C_8H_7LiO_3SNa^+$ [M+Na]$^+$: 213.0174; found: 213.0174.

Lithium 2-oxooctanoate (1l)

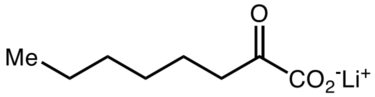

White solid. ^{1}H NMR (400 MHz, DMSO-d_6) δ: 2.43 (t, *J* = 7.3 Hz, 2H), 1.51 – 1.36 (m, 2H), 1.30 – 1.16 (m, 6H), 0.85 (t, *J* = 6.7 Hz, 3H) ppm. ^{13}C NMR (101 MHz, DMSO-d_6) δ: 206.8, 169.1, 38.7, 31.2, 28.5, 23.0, 22.0, 13.9 ppm.

IR: 2956, 2855, 1726, 1654, 1461, 1376, 1261, 1168, 1122, 1070 cm^{-1}. HRMS (ESI): calcd. for $C_8H_{13}LiO_3Na^+$ [M+Na]$^+$: 187.0917; found: 187.0917.

Lithium 4-cyclohexyl-2-oxobutanoate (1m)

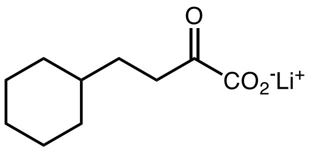

White solid. ^{1}H NMR (400 MHz, DMSO-d_6) δ: 2.44 (t, J = 7.6 Hz, 2H), 1.86 – 1.46 (m, 5H), 1.46 – 1.26 (m, 2H), 1.21 – 1.03 (m, 4H), 0.93 – 0.72 (m, 2H) ppm. ^{13}C NMR (101 MHz, DMSO-d_6) δ: 207.0, 169.2, 36.8, 36.2, 32.7, 30.5, 26.2, 25.8 ppm. IR: 2923, 2851, 1727, 1449, 1400, 1261, 1095, 1067, 1023 cm^{-1}. HRMS (ESI): calcd. for $C_{10}H_{15}LiO_3Na^+$ [M+Na]$^+$: 213.1073; found: 213.1076.

Characterization data for L-Methionine analogues

***S*-allyl-L-homocysteine (SI-1a)**

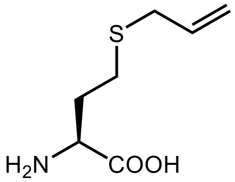

Purified by Biotage (Sfär C18 Duo, 0-100% MeOH/H_2O with 0.1% formic acid for 8 CV) to afford the product as a white solid. ^{1}H NMR (400 MHz, D_2O) δ: 5.97 – 5.69 (m, 1H), 5.26 – 5.09 (m, 2H), 4.19 (t, J = 6.3 Hz, 1H), 3.23 (d, J = 7.2 Hz, 2H), 2.67 (t, J = 7.5 Hz, 2H), 2.31 – 2.12 (m, 2H) ppm. ^{13}C NMR (101 MHz, D_2O) δ: 171.8, 133.8, 117.8, 52.0, 33.3, 29.3, 25.0 ppm. Spectral data match those previously reported[12].

***S*-(prop-2-yn-1-yl)-L-homocysteine (SI-1b)**

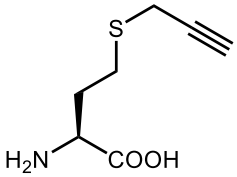

Purified by Biotage (Sfär C18 Duo, 0-100% MeOH/H_2O with 0.1% formic acid for 8 CV) to afford the product as a white solid. ^{1}H NMR (400 MHz, D_2O) δ: 4.04 (t, J = 6.3 Hz, 1H), 3.40 (d, J = 2.5 Hz, 2H), 2.88 (t, J = 7.5 Hz, 2H), 2.69 (t, J = 2.0 Hz, 1H), 2.35 – 2.14 (m, 2H) ppm. ^{13}C NMR (101 MHz, D_2O) δ: 173.0, 80.3, 72.2, 52.9, 29.5, 26.4, 18.0 ppm. Spectral data match those previously reported[12].

VIII. Characterization data for asymmetric alkylation products

General procedure for the synthesis of racemic products

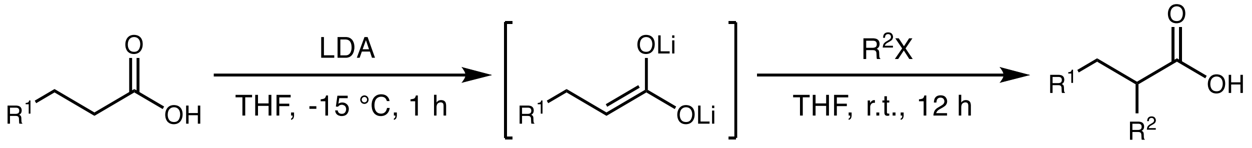

This procedure is modified from a published procedure[13].

The carboxylic acid (10-20 mmol, 1.0 equiv) was dissolved in anhydrous THF and added slowly to a solution of LDA (0.4 M in anhydrous THF, 2.5 equiv) at -15 ℃ under an argon atmosphere. The resulting mixture was allowed to stir at -15 ℃ for 1 h. Haloalkane (2.2 equiv) was then added dropwise to the solution. The reaction mixture was then warmed to room temperature, stirred overnight, and quenched with water. This mixture was treated with 1 M HCl and extracted with EtOAc (3×). The combined organic layer was washed with brine, dried over Na_2SO_4, and concentrated *in vacuo*. The crude product was purified by flash column chromatography with the aid of Biotage Isolera to afford analytically pure product in 75 – 95% yield.

Enantioenriched enzymatic products were synthesized by enzymatic reactions using the general procedure described above.

Characterization data for biocatalytic asymmetric alkylation products

(*R*)-2-methyl-3-phenylpropanoic acid (3a)

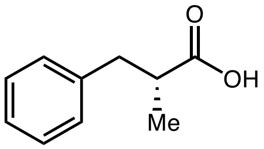

Purified by Biotage (Sfär C18 Duo, 0-100% MeOH/H_2O with 0.1% HCOOH for 8 CV) to afford the product as a white solid. ^{1}H NMR (400 MHz, $CDCl_3$) δ: 11.99 (s, 1H), 7.37 – 7.28 (m, 2H), 7.27 – 7.15 (m, 3H), 3.11 (dd, J = 13.3, 6.3 Hz, 1H), 2.84 – 2.75 (m, 1H), 2.70 (dd, J = 13.3, 8.0 Hz, 1H), 1.21 (d, J = 6.9 Hz, 3H) ppm. ^{13}C NMR (101 MHz, $CDCl_3$) δ: 183.0, 139.1, 129.1, 128.6, 126.6, 41.4, 39.4, 16.6 ppm. Spectral data match those previously reported[14].

HPLC analysis (OJ-H column, 98: 2: 0.1 hexanes: *i*-PrOH: TFA, 1.0 mL/min) indicated 99:1 e.r. using SgvM^{VAV}: t_R = 11.7 (major), 13.6 (minor) min.

(*R*)-2-methyl-3-(*o*-tolyl)propanoic acid (3b)

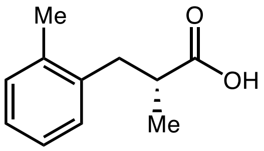

Purified by Biotage (Sfär C18 Duo, 0-100% MeOH/H_2O with 0.1% HCOOH for 8 CV) to afford the product as a white solid. ^{1}H NMR (500 MHz, $CDCl_3$) δ: 11.45 (s, 1H), 7.23 – 7.13 (m, 4H), 3.15 (dd, *J* = 13.8, 6.3 Hz, 1H), 2.85 – 2.74 (m, 1H), 2.69 (dd, *J* = 13.8, 8.4 Hz, 1H), 2.37 (s, 3H), 1.24 (d, *J* = 6.9 Hz, 3H) ppm. ^{13}C NMR (126 MHz, $CDCl_3$) δ: 183.1, 137.4, 136.4, 130.5, 129.8, 126.7, 126.0, 40.1, 36.7, 19.5, 16.7 ppm. IR: 3445, 2931, 1706, 1458, 1415, 1285, 1235, 1102, 1025 cm^{-1}. HRMS (ESI): calcd. for $C_{11}H_{13}O_2^-$ [M-H]$^-$: 177.0921; found: 177.0921.

HPLC analysis (OJ-H column, 98: 2: 0.1 hexanes: *i*-PrOH: TFA, 1.0 mL/min) indicated > 99:1 e.r. using SgvM^{VAV}: t_R = 8.7 (major), 9.2 (minor) min.

(*R*)-2-methyl-3-(*m*-tolyl)propanoic acid (3c)

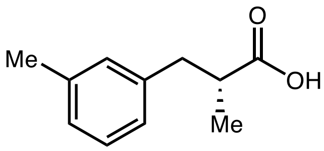

Purified by Biotage (Sfär C18 Duo, 0-100% MeOH/H_2O with 0.1% HCOOH for 8 CV) to afford the product as a white solid. ^{1}H NMR (500 MHz, $CDCl_3$) δ: 11.35 (s, 1H), 7.20 (t, *J* = 7.5 Hz, 1H), 7.10 – 6.93 (m, 3H), 3.07 (dd, *J* = 13.5, 6.4 Hz, 1H), 2.83 – 2.70 (m, 1H), 2.64 (dd, *J* = 13.5, 8.2 Hz, 1H), 2.35 (s, 3H), 1.19 (d, *J* = 7.0 Hz, 3H) ppm. ^{13}C NMR (126 MHz, $CDCl_3$) δ: 182.9, 139.1, 138.1, 129.9, 128.4, 127.3, 126.1, 41.4, 39.3, 21.5, 16.6 ppm. IR: 3450, 2973, 2925, 2659, 1706, 1460, 1378, 1292, 1239, 1095 cm^{-1}. HRMS (ESI): calcd. for $C_{11}H_{13}O_2^-$ [M-H]$^-$: 177.0921; found: 177.0923.

HPLC analysis (OJ-H column, 98: 2: 0.1 hexanes: *i*-PrOH: TFA, 1.0 mL/min) indicated 98:2 e.r. using SgvM^{VAV}: t_R = 9.4 (major), 10.4 (minor) min.

(*R*)-2-methyl-3-(*p*-tolyl)propanoic acid (3d)

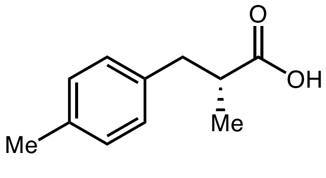

Purified by Biotage (Sfär C18 Duo, 0-100% MeOH/H_2O with 0.1% HCOOH for 8 CV) to afford the product as a white solid. ^{1}H NMR (500 MHz, $CDCl_3$) δ: 11.61 (s, 1H), 7.15 – 7.06 (m, 4H), 3.06 (dd, *J* = 13.5, 6.5 Hz, 1H), 2.81 – 2.70 (m, 1H), 2.65 (dd, *J* = 13.5, 8.0 Hz, 1H), 2.34 (s, 3H), 1.19 (d, *J* = 6.9 Hz, 3H) ppm. ^{13}C NMR (126 MHz, $CDCl_3$) δ: 182.9, 136.0, 129.2,

129.0, 41.5, 39.0, 21.2, 16.6 ppm. IR: 3385, 2921, 2850, 2371, 2340, 1706, 1650, 1465, 1108, 1031 cm^{-1}. HRMS (ESI): calcd. for $C_{11}H_{13}O_2^-$ $[M-H]^-$: 177.0921; found: 177.0920.

HPLC analysis (OJ-H column, 98: 2: 0.1 hexanes: *i*-PrOH: TFA, 1.0 mL/min) indicated 99:1 e.r. using $SgvM^{VAV}$: t_R = 11.3 (major), 12.6 (minor) min.

(*R*)-3-(4-methoxyphenyl)-2-methylpropanoic acid (3e)

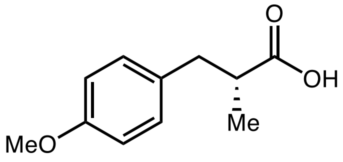

Purified by Biotage (Sfär C18 Duo, 0-100% MeOH/H_2O with 0.1% HCOOH for 8 CV) to afford the product as a white solid. ^1H NMR (500 MHz, $CDCl_3$) δ: 11.40 (s, 1H), 7.14 – 7.04 (m, 2H), 6.88 – 6.80 (m, 2H), 3.79 (s, 3H), 3.02 (dd, *J* = 13.6, 6.5 Hz, 1H), 2.79 – 2.67 (m, 1H), 2.63 (dd, *J* = 13.6, 7.9 Hz, 1H), 1.18 (d, *J* = 6.9 Hz, 3H) ppm. ^{13}C NMR (126 MHz, $CDCl_3$) δ: 182.8, 158.3, 131.2, 130.1, 113.9, 55.3, 41.6, 38.6, 16.5 ppm. IR: 3446, 2926, 1705, 1612, 1513, 1461, 1246, 1178, 1110, 1034 cm^{-1}. HRMS (ESI): calcd. for $C_{11}H_{13}O_3^-$ $[M-H]^-$: 193.0870; found: 193.0866.

HPLC analysis (OJ-H column, 98: 2: 0.1 hexanes: *i*-PrOH: TFA, 1.0 mL/min) indicated >99:1 e.r. using $SgvM^{VAV}$: t_R = 27.0 (major), 29.6 (minor) min.

(*R*)-3-(2-methoxyphenyl)-2-methylpropanoic acid (3f)

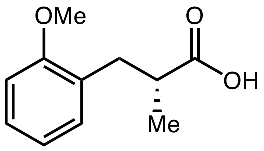

Purified by Biotage (Sfär C18 Duo, 0-100% MeOH/H_2O with 0.1% HCOOH for 8 CV) to afford the product as a white solid. ^1H NMR (400 MHz, $CDCl_3$) δ: 7.24 – 7.16 (m, 1H), 7.13 (dd, *J* = 7.4, 1.7 Hz, 1H), 6.93 – 6.78 (m, 2H), 3.81 (s, 3H), 3.05 (dd, *J* = 13.2, 6.7 Hz, 1H), 2.92 – 2.80 (m, 1H), 2.71 (dd, *J* = 13.2, 7.6 Hz, 1H), 1.16 (d, *J* = 7.0 Hz, 3H) ppm. ^{13}C NMR (101 MHz, $CDCl_3$) δ: 182.8, 157.8, 131.0, 127.9, 127.6, 120.4, 110.3, 55.3, 39.4, 34.4, 16.9 ppm. IR: 3446, 2926, 2854, 2363, 1705,1462, 1290, 1244, 1123, 1028 cm^{-1}. HRMS (ESI): calcd. for $C_{11}H_{13}O_3^-$ $[M-H]^-$: 193.0870; found: 177.0865.

HPLC analysis (OJ-H column, 98: 2: 0.1 hexanes: *i*-PrOH: TFA, 1.0 mL/min) indicated > 99:1 e.r. using $SgvM^{VAV}$: t_R = 11.9 (major), 13.0 (minor) min.

(*R*)-2-methyl-3-(2-(trifluoromethyl)phenyl)propanoic acid (3g)

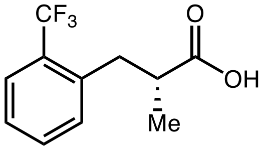

Purified by Biotage (Sfär C18 Duo, 0-100% MeOH/H_2O with 0.1% HCOOH for 8 CV) to afford the product as a white solid. ^{1}H NMR (400 MHz, $CDCl_3$) δ: 10.84 (s, 1H), 7.65 (d, *J* = 7.8 Hz, 1H), 7.47 (t, *J* = 7.5 Hz, 1H), 7.41 – 7.28 (m, 2H), 3.27 (dd, *J* = 13.8, 6.7 Hz, 1H), 2.95 – 2.80 (m, 2H), 1.24 (d, *J* = 6.7 Hz, 3H) ppm. ^{13}C NMR (101 MHz, $CDCl_3$) δ: 182.7, 137.9 (q, *J* = 1.7 Hz), 131.9, 131.5, 129.1 (q, *J* = 29.7 Hz), 126.8, 126.4 (q, *J* = 5.8 Hz), 124.7 (q, *J* = 273.8 Hz), 41.0, 35.8, 17.1 ppm. ^{19}F NMR (376 MHz, $CDCl_3$) δ: -59.3 (s) ppm. IR: 2998, 2884, 1693, 1454, 1310, 1241, 1169, 1106, 1075, 1059 cm^{-1}. HRMS (ESI): calcd. for $C_{11}H_{10}F_3O_2Na_2^+$ [M-H+2Na]$^+$: 277.0428; found: 277.0434.

HPLC analysis (IG column, 98: 2 hexanes: *i*-PrOH, 1.0 mL/min) indicated 98: 2 e.r. using SgvM^{VAV}: t_R = 4.8 (minor), 5.4 (major) min.

(*R*)-3-(4-fluorophenyl)-2-methylpropanoic acid (3h)

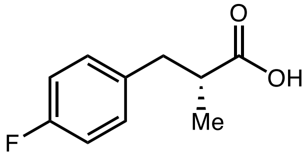

Purified by Biotage (Sfär C18 Duo, 0-100% MeOH/H_2O with 0.1% HCOOH for 8 CV) to afford the product as a white solid. ^{1}H NMR (400 MHz, $CDCl_3$) δ: 11.17 (s, 1H), 7.21 – 7.06 (m, 2H), 7.05 – 6.83 (m, 2H), 3.03 (dd, *J* = 13.2, 6.4 Hz, 1H), 2.78 – 2.63 (m, 2H), 1.18 (d, *J* = 6.8 Hz, 3H) ppm. ^{13}C NMR (101 MHz, $CDCl_3$) δ: 182.3, 161.6 (d, *J* = 244.3 Hz), 134.6 (d, *J* = 3.3 Hz), 130.4 (d, *J* = 7.8 Hz), 115.2 (d, *J* = 21.1 Hz), 41.4, 38.5, 16.5 ppm. ^{19}F NMR (376 MHz, $CDCl_3$) δ: -116.67 – -116.76 (m, 1F) ppm. IR: 3447, 2925, 1707, 1608, 1510, 1460, 1416, 1226, 1069, 1025 cm^{-1}. HRMS (ESI): calcd. for $C_{10}H_{10}FO_2^-$ [M-H]$^-$: 181.0670; found: 181.0668.

HPLC analysis (OJ-H column, 98: 2: 0.1 hexanes: *i*-PrOH: TFA, 1.0 mL/min) indicated 98:2 e.r. using SgvM^{VAV}: t_R = 12.0 (major), 13.6 (minor) min.

(*R*)-3-(4-chlorophenyl)-2-methylpropanoic acid (3i)

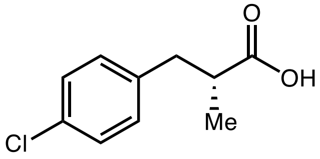

Purified by Biotage (Sfär C18 Duo, 0-100% MeOH/H_2O with 0.1% HCOOH for 8 CV) to afford the product as a white solid. ^{1}H NMR (400 MHz, $CDCl_3$) δ: 11.74 (s, 1H), 7.33 – 7.20 (m, 2H), 7.19 – 7.06 (m, 2H), 3.04 (dd, *J* = 13.3, 6.5 Hz, 1H), 2.81 – 2.71 (m, 1H), 2.67 (dd, *J* =

13.3, 7.7 Hz, 1H), 1.20 (d, J = 6.9 Hz, 3H) ppm. ^{13}C NMR (101 MHz, $CDCl_3$) δ: 182.7, 137.5, 132.4, 130.4, 128.7, 41.29, 38.6, 16.6 ppm. Spectral data match those previously reported[14].
HPLC analysis (OJ-H column, 98: 2: 0.1 hexanes: *i*-PrOH: TFA, 1.0 mL/min) indicated 99:1 e.r. using SgvM^{VAV}: t_R = 11.9 (major), 12.7 (minor) min.

(*R*)-3-(benzo[*d*][1,3]dioxol-5-yl)-2-methylpropanoic acid (3j)

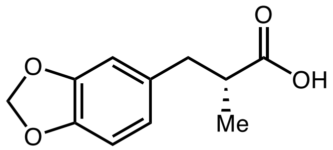

Purified by Biotage (Sfär C18 Duo, 0-100% MeOH/H_2O with 0.1% HCOOH for 8 CV) to afford the product as a white solid. ^{1}H NMR (500 MHz, $CDCl_3$) δ: 11.51 (s, 1H), 6.73 (d, J = 7.9 Hz, 1H), 6.68 (d, J = 1.7 Hz, 1H), 6.63 (dd, J = 7.9, 1.7 Hz, 1H), 5.93 (s, 2H), 2.98 (dd, J = 13.6, 6.6 Hz, 1H), 2.75 – 2.65 (m, 1H), 2.60 (dd, J = 13.6, 7.8 Hz, 1H), 1.17 (d, J = 7.0 Hz, 3H) ppm. ^{13}C NMR (126 MHz, $CDCl_3$) δ: 182.6, 147.7, 146.2, 132.9, 122.1, 109.5, 108.3, 101.0, 41.6, 39.2, 16.6 ppm. IR: 3446, 2924, 1705, 1494, 1443, 1417, 1247, 1192, 1099, 1039 cm^{-1}. HRMS (ESI): calcd. for $C_{11}H_{11}O_4^-$ [M-H]$^-$: 207.0663; found: 207.0656.
HPLC analysis (OJ-H column, 98: 2: 0.1 hexanes: *i*-PrOH: TFA, 1.0 mL/min) indicated 98:2 e.r. using SgvM^{VAV}: t_R = 25.7 (major), 30.3 (minor) min.

(*R*)-2-methyl-3-(thiophen-2-yl)propanoic acid (3k)

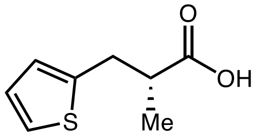

Purified by Biotage (Sfär C18 Duo, 0-100% MeOH/H_2O with 0.1% HCOOH for 8 CV) to afford the product as a colorless oil. ^{1}H NMR (400 MHz, $CDCl_3$) δ: 11.19 (s, 1H), 7.15 (dd, J = 5.2, 1.1 Hz, 1H), 6.93 (dd, J = 5.2, 3.4 Hz, 1H), 6.84 (d, J = 3.4 Hz, 1H), 3.26 (dd, J = 14.7, 6.6 Hz, 1H), 2.94 (dd, J = 14.7, 7.3 Hz, 1H), 2.88 – 2.70 (m, 1H), 1.24 (d, J = 6.8 Hz, 3H) ppm. ^{13}C NMR (101 MHz, $CDCl_3$) δ: 182.2, 141.4, 127.0, 125.9, 124.0, 41.8, 33.3, 16.7 ppm. IR: 3445, 2924, 2854, 2363, 1706, 1460, 1290, 1233, 1150, 1072 cm^{-1}. HRMS (ESI): calcd. for $C_8H_9O_2S^-$ [M-H]$^-$: 169.0329; found: 169.0332.
HPLC analysis (OJ-H column, 98: 2: 0.1 hexanes: *i*-PrOH: TFA, 1.0 mL/min) indicated 98:2 e.r. using SgvM^{VAV}: t_R = 12.6 (major), 15.5 (minor) min.

(*R*)-2-methylheptanoic acid (3l)

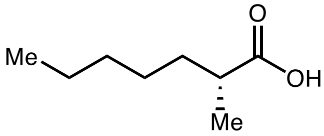

Purified by Biotage (Sfär C18 Duo, 0-100% MeOH/H_2O with 0.1% HCOOH for 8 CV) to afford the product as a colorless oil. ^{1}H NMR (500 MHz, $CDCl_3$) δ: 11.71 (s, 1H), 2.50 – 2.40 (m, 1H), 1.71 – 1.64 (m, 1H), 1.45 – 1.38 (m, 1H), 1.35 – 1.24 (m, 6H), 1.17 (d, *J* = 7.0 Hz, 3H), 0.88 (t, *J* = 6.9 Hz, 3H) ppm. ^{13}C NMR (126 MHz, $CDCl_3$) δ: 184.0, 39.6, 33.6, 31.8, 27.0, 22.6, 17.0, 14.2 ppm. IR: 3452, 2925, 2858, 1708, 1458, 1377, 1292, 1240, 1194, 1098 cm^{-1}. HRMS (ESI): calcd. for $C_8H_{15}O_2^-$ $[M-H]^-$: 143.1078; found: 143.1080.

GC analysis (cyclosil B column, isothermal at 120 °C for 35 min) indicated 99:1 e.r. using SgvM^{VAV}: t_R = 16.3 (minor), 16.9 (major) min.

(*R*)-3-cyclohexyl-2-methylpropanoic acid (3m)

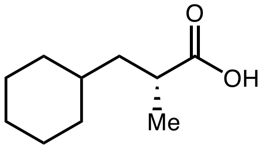

Purified by Biotage (Sfär C18 Duo, 0-100% MeOH/H_2O with 0.1% HCOOH for 8 CV) to afford the product as a colorless oil. ^{1}H NMR (400 MHz, $CDCl_3$) δ: 11.19 (s, 1H), 2.63 – 2.50 (m, 1H), 1.78 – 1.57 (m, 6H), 1.35 – 1.10 (m, 8H), 0.93 – 0.81 (m, 2H) ppm. ^{13}C NMR (101 MHz, $CDCl_3$) δ: 184.2, 41.4, 36.9, 35.4, 33.4, 33.2, 26.7, 26.4, 26.3, 17.5 ppm. IR: 2970, 2921, 2851, 1738, 1703, 1448, 1415, 1366, 1282, 1230 cm^{-1}. HRMS (ESI): calcd. for $C_{10}H_{17}O_2Na_2^+$ $[M-H+2Na]^+$: 215.1024; found: 215.1028.

GC analysis (cyclosil B column, isothermal at 135 °C for 35 min) indicated 99:1 e.r. using SgvM^{VAV}: t_R = 29.7 (major), 31.5 (minor) min.

(*R*)-2-benzylpent-4-enoic acid (3n)

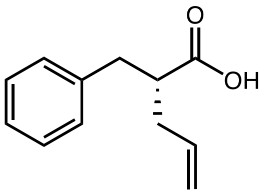

Purified by Biotage (Sfär C18 Duo, 0-100% MeOH/H_2O with 0.1% HCOOH for 8 CV) to afford the product as a yellow oil. ^{1}H NMR (400 MHz, $CDCl_3$) δ: 11.15 (s, 1H), 7.44 – 7.27 (m, 2H), 7.27 – 7.16 (m, 3H), 5.91 – 5.69 (m, 1H), 5.18 – 5.03 (m, 2H), 3.07 – 2.97 (m, 1H), 2.88 – 2.75 (m, 2H), 2.46 – 2.29 (m, 2H) ppm. ^{13}C NMR (101 MHz, $CDCl_3$) δ: 181.6, 138.9, 134.9, 129.1, 128.6, 126.6, 117.6, 47.2, 37.4, 35.7 ppm. IR: 3027, 2990, 2950, 1738, 1704, 1444, 1366, 1229, 1217, 1206 cm^{-1}. HRMS (ESI): calcd. for $C_{12}H_{13}O_2Na_2^+$ $[M-H+2Na]^+$: 235.0711; found: 235.0709. HPLC

analysis (OJ-H column, 98: 2: 0.1 hexanes: *i*-PrOH: TFA, 1.0 mL/min) indicated 98:2 e.r. using SgvM^{VAV}: t_R = 11.0 (minor), 13.0 (major) min.

(*R*)-2-benzylpent-4-ynoic acid (3o)

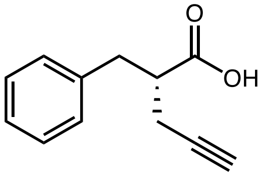

Purified by Biotage (Sfär C18 Duo, 0-100% MeOH/H_2O with 0.1% HCOOH for 8 CV) to afford the product as a colorless oil. ^1H NMR (500 MHz, $CDCl_3$) δ: 11.26 (s, 1H), 7.34 – 7.27 (m, 2H), 7.26 – 7.19 (m, 3H), 3.11 (dd, J = 13.7, 6.6 Hz, 1H), 2.98 (dd, J = 13.7, 7.5 Hz, 1H), 2.93 – 2.87 (m, 1H), 2.49 – 2.41 (m, 2H), 2.08 (t, J = 2.6 Hz, 1H) ppm. ^{13}C NMR (126 MHz, $CDCl_3$) δ: 179.8, 138.1, 129.2, 128.7, 126.9, 80.9, 70.8, 46.0, 36.4, 20.1 ppm. IR: 3465, 3291, 3028, 2968, 2950, 1738, 1433, 1366, 1228, 1217 cm^{-1}. HRMS (ESI): calcd. For $C_{12}H_{11}O_2Na_2^+$ [M-H+2Na]$^+$: 233.0555; found: 233.0558. HPLC analysis (OJ-H column, 95: 5 hexanes: *i*-PrOH, 1.0 mL/min) indicated 97:3 e.r. using SgvM^{VAV}: t_R = 9.3 (minor), 11.2 (major) min.

(*R*)-2-benzylbutanoic acid (3p)

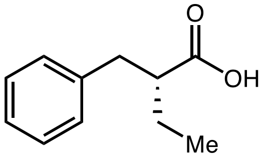

Purified by Biotage (Sfär C18 Duo, 0-100% MeOH/H_2O with 0.1% HCOOH for 8 CV) to afford the product as a colorless oil. ^1H NMR (400 MHz, $CDCl_3$) δ: 10.80 (s, 1H), 7.39 – 7.27 (m, 2H), 7.27 – 7.13 (m, 3H), 3.02 (dd, J = 13.8, 7.8 Hz, 1H), 2.79 (dd, J = 13.7, 7.0 Hz, 1H), 2.70 – 2.60 (m, 1H), 1.75 – 1.57 (m, 2H), 0.99 (t, J = 7.4 Hz, 3H) ppm. ^{13}C NMR (101 MHz, $CDCl_3$) δ: 182.3, 139.3, 129.0, 128.6, 126.5, 49.0, 37.8, 24.9, 11.7 ppm. IR: 3028, 2969, 2878, 1738, 1701, 1454, 1366, 1285, 1217 cm^{-1}. HRMS (ESI): calcd. For $C_{11}H_{13}O_2Na_2^+$ [M-H+2Na]$^+$: 223.0711; found: 223.0720.

HPLC analysis (OJ-H column, 98: 2: 0.1 hexanes: *i*-PrOH: TFA, 1.0 mL/min) indicated 99:1 e.r. using SgvM^{VAV}: t_R = 12.8 (major), 14.2 (minor) min.

IX. Procedure for derivatization reactions and characterization data.

Preparative scale biocatalytic transformation (100 mg scale)

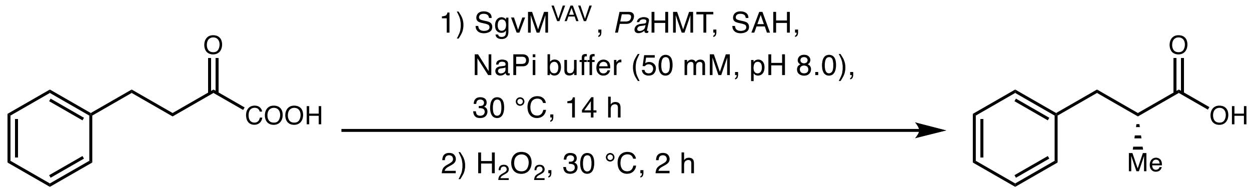

1a **3a**

To a 100 mL flask were added NaPi buffer (50 mM, pH 8.0), SAH (60 μL of 10 mM stock solution in DMSO), MeI (3 mL of 400 mM stock solution in DMSO), SgvM^{VAV}, *Pa*HMT, and substrate **1a** (15 mL of 40 mM stock solution in NaPi buffer) in succession. Final reaction volume was 30 mL; final concentrations were 20 mM substrate **1a**, 20 μM SAH, 40 mM MeI, 20 μM SgvM^{VAV}, 20 μM *Pa*HMT. The reaction flask was sealed and shaken at 30 °C and 250 rpm for 14 h. Upon the completion of the reaction, the reaction mixture was then treated with H_2O_2 (30% w/w) and allowed to stir at room temperature for 2 h. Excess H_2O_2 was decomposed with catalase. The resulting mixture was concentrated *in vacuo*, and the pH of the aqueous layer was adjusted to 3-4 using 1.0 M HCl (aq.). This mixture was then shaken vigorously and centrifuged (12,000 x g, 20 min).

The supernatant containing the product was filtered and loaded onto a Biotage Sfär C18 cartridge (12 g) that had been equilibrated to 10% MeOH/H_2O with 0.1% formic acid. The column was washed with 2 column volumes (CV) of 10% MeOH/H_2O (0.1% formic acid). Then, the product was flushed with a gradient from 10% to 100% methanol with 0.1% formic acid over 8 CV. Product-containing fractions were combined and concentrated *in vacuo*. The residual aqueous solution was lyophilized to afford the analytically pure product as a white powder in 81% yield (99:1 e.r.).

Cascade biotransformations leading to hydroxy acid 4a

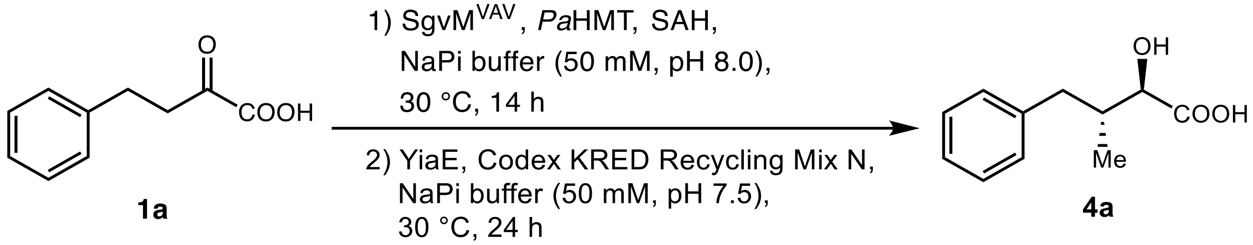

To a 100 mL flask were added NaPi buffer (50 mM, pH 8.0), SAH (20 μL of 10 mM stock solution in DMSO), MeI (1 mL of 400 mM stock solution in DMSO), SgvM^{VAV}, *Pa*HMT, and substrate **1a** (5 mL of 40 mM stock solution in NaPi buffer) in succession. Final reaction volume was 10 mL; final concentrations were 20 mM substrate, 20 μM SAH, 40 mM MeI, 20 μM SgvM^{VAV} and 20 μM *Pa*HMT. The reaction flask was sealed and shaken at 30 °C and 250 rpm for 14 h. The resulting mixture was concentrated *in vacuo* with the aid of a rotary evaporator. The pH value of the residual mixture was carefully adjusted to 7.5 by using *o*-phosphoric acid.

To a new 50 mL falcon tube were added NaPi buffer (50 mM, pH 7.5), residual reaction mixture obtained above, Codex® KRED Recycling Mix N (0.1 g), and YiaE (2 mol%) in succession. Final reaction volume was 10 mL. The tube was sealed and shaken at 30 °C and 250 rpm for 24 h. The reaction mixture was carefully adjusted to pH 3-4 with HCl (1.0 M), shaken vigorously, and centrifuged (12,000 x g, 20 min). The supernatant was filtered and lyophilized to afford crude product. The sample was then dissolved in 10% MeOH/H_2O with 0.1% formic acid and loaded onto a C18 column (12 g) that had been equilibrated to 10% MeOH/H_2O with 0.1% formic acid. The column was washed with 2 CV of 10% MeOH/H_2O with 0.1% formic acid. Then, the product was eluted with a gradient from 10% to 100% methanol with 0.1% formic acid over 8 CV. Product-containing fractions were combined and concentrated *in vacuo*. The residual aqueous solution was lyophilized to afford the analytically pure product as white powder in 76% yield (30 mg).

To further confirm the diastereoselectivity and enantioselectivity of the hydroxy acid **4a**, chemo-enzymatic synthesis of racemic **4a** was performed. The 10 mL reaction solution of SgvM^{VAV}-*Pa*HMT cascade (described above) was shaken at 30 °C and 250 rpm for 14 h. Sodium borohydride (10 equiv) and MeOH (10 mL) were then added. The reaction mixture was allowed to stire at room temperature for 12 h. After purification, the product was analyzed by HPLC.

(2*R*,3*R*)-2-hydroxy-3-methyl-4-phenylbutanoic acid (4a)

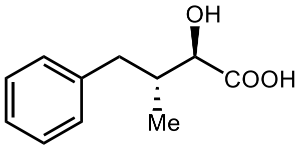

Purified by Biotage (Sfär C18 Duo, 0-100% MeOH/H_2O with 0.1% HCOOH for 8 CV) to afford the product as a white solid. ^{1}H NMR (400 MHz, D_2O) δ: 7.41 – 7.34 (m, 2H), 7.33 – 7.25 (m, 3H), 4.22 (d, *J* = 3.9 Hz, 1H), 2.78 (dd, *J* = 13.5, 5.2 Hz, 1H), 2.50 (dd, *J* = 13.5, 9.4 Hz, 1H), 2.33 – 2.23 (m, 1H), 0.93 (d, *J* = 7.0 Hz, 3H) ppm. ^{13}C NMR (101 MHz, D_2O) δ 177.5, 140.6, 129.4, 128.4, 126.2, 74.3, 38.7, 36.9, 15.1 ppm. IR: 3453, 3016, 2970, 2950, 1738, 1435, 1365, 1355, 1228, 1217 cm^{-1}. HRMS (ESI): calcd. For $C_{11}H_{13}O_3Na_2^+$ [M-H+2Na]$^+$: 239.0660; found: 239.0662.

HPLC analysis (AD-H column, 90:10:0.1 hexanes: *i*-PrOH: TFA, 1.0 mL/min) indicated 0.96:1 d.r. using chemo-enzymatic approach: t_R = 8.115 (major diastereomer 1), 4.504 (enantiomer of major diastereomer 1), 7.210 (major diastereomer 2), 6.623 (enantiomer of major diastereomer 2) min. Enantioenriched **4a** obtained by using multienzymatic cascade was analyzed under the same HPLC conditions, indicating 19:1 d.r. and >99:1 e.r. for for major diasterromer: t_R = 8.031 (major diastereomer), 6.538 (minor diastereomer),

Racemic **4a**: 0.96:1 d.r.

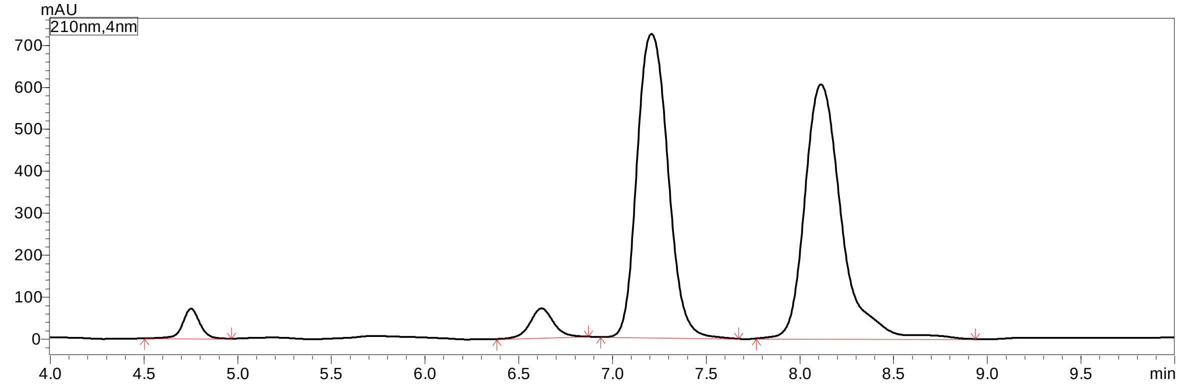

Peak#	Ret. Time	Area	Area %	Height
1	4.753	448385	2.553	70561
2	6.623	611984	3.484	70093
3	7.210	8285420	47.167	722661
4	8.115	8220278	46.796	605287

Enantioenriched **4a** prepared by biocatalytic cascades: 19:1 d.r., >99:1 e.r.

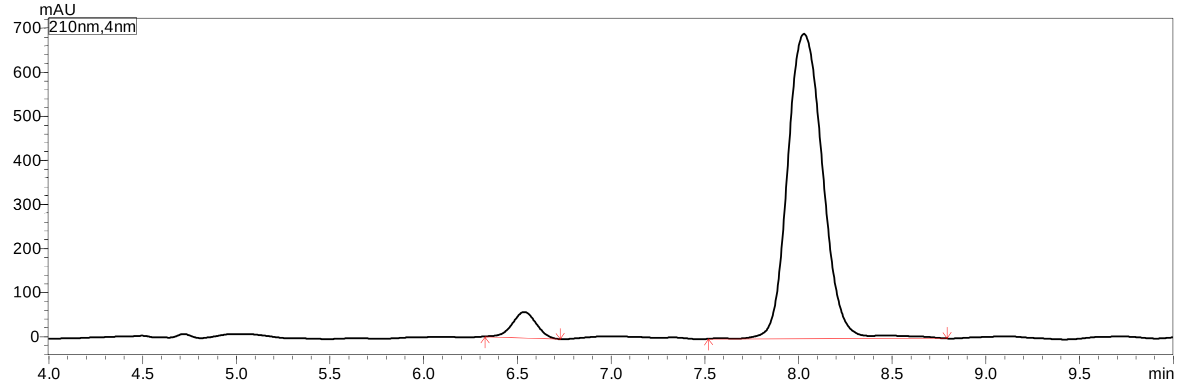

Peak#	Ret. Time	Area	Area %	Height
1	6.538	521490	5.453	58480
2	8.031	9042035	94.547	691945

Reductive amination of α-keto acid to chiral amino acid

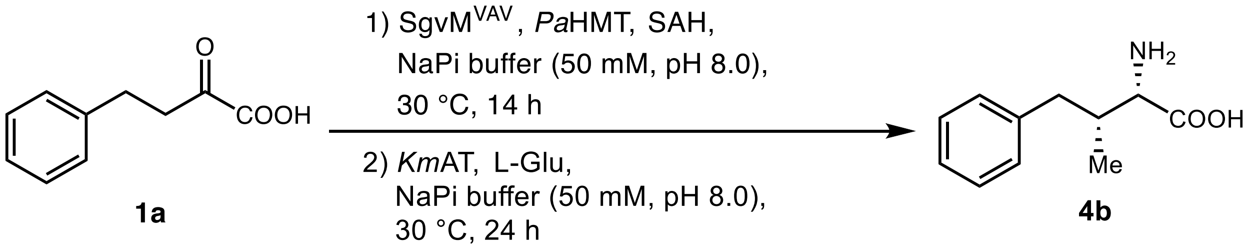

To a 50 mL falcon tube were added NaPi buffer (50 mM, pH 8.0), SAH (20 μL of 10 mM stock solution in DMSO), MeI (1 mL of 400 mM stock solution in DMSO), SgvM^{VAV}, *Pa*HMT, and substrate **1a** (5 mL of 40 mM stock solution in NaPi buffer) in succession. Final reaction volume was 10 mL; final concentrations were 20 mM substrate, 20 μM SAH, 40 mM MeI, 20 μM SgvM^{VAV} and 20 μM *Pa*HMT. The tube was sealed and shaken at 30 °C and 250 rpm for 14 h. The resulting mixture was concentrated *in vacuo* with the aid of a rotary evaporator.

To a new 50 mL falcon tube were added NaPi buffer (50 mM, pH 8.0), the residue from the former biotransformation, L-glutamic acid monosodium salt (0.51 g, final concentration 300 mM), pyridoxal 5′ -phosphate (10 μL of 100 mM stock solution in NaPi buffer) and *Km*AT (2 mol%) in succession. Final reaction volume was 10 mL. The tube was sealed and shaken at 30 °C and 250 rpm for 24 h. The pH of the reaction mixture was carefully adjusted to 3-4 with 1.0 M HCl (aq.),

shaken vigorously, and centrifuged (12,000 x g, 20 min). The supernatant was filtered and loaded onto a C18 column (12 g) that had been equilibrated to 10% MeOH/H_2O with 0.1% formic acid. The column was washed with 2 CV of 10% MeOH/H_2O with 0.1% formic acid. Then, the product was eluted with a gradient from 10% to 100% methanol with 0.1% formic acid over 8 CV. Product-containing fractions were combined and concentrated *in vacuo* with the aid of a rotary evaporator. The residual aqueous solution was lyophilized to afford the product as a white powder in 68% yield (26 mg). $[\alpha]_D^{20}$ = 23.2° (c = 0.11, H_2O). This result matched those previously reported[15], supporting the assigned relative and absolute stereochemistry of this compound.

To further confirm the diastereoselectivity and enantioselectivity of the amino acid **4b**, Marfey analysis was carried out. To a 2 mL vial were added Na_2HCO_3 (80 µL of 1 M stock solution), DMSO (50 µL), (*R*)-2-((5-fluoro-2,4-dinitrophenyl)amino)propenamide (D-Marfey's reagent, 50 µL of 0.02 M stock solution in acetone), and **4b** (25 µL of 0.02 M stock solution in 1 M HCl: MeCN=1:1) in succession. A parallel effort was performed using L-Marfey's reagent (50 µL of 0.02 M stock solution in acetone) in place of D-Marfey's reagent. The vials were sealed and shaken at room temperature and 800 rpm for 8-9 h. In a 2 mL Eppendorf tube, the reaction mixture was then diluted with a mixed solvent system (1M HCl: MeCN=1:1, 1 mL). After centrifugation (12,000 x g, 5 min), the supernatant was filtered through a PTFE membrane syringe filter (0.22 µm) and transferred to a 500 µL vial insert, which was then placed in a 2 mL HPLC vial and analyzed by LC-MS.

(2*S*,3*R*)-2-amino-3-methyl-4-phenylbutanoic acid (4b)

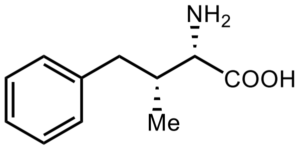

Purified by Biotage (Sfär C18 Duo, 0-100% MeOH/H_2O with 0.1% formic acid for 8 CV) to afford the product as a white solid. ^1H NMR (400 MHz, D_2O) δ: 7.51 – 7.38 (m, 2H), 7.37 – 7.19 (m, 3H), 3.70 (d, J = 3.3 Hz, 1H), 2.84 (dd, J = 13.4, 6.1 Hz, 1H), 2.61 (dd, J = 13.4, 9.3 Hz, 1H), 2.55 – 2.47 (m, 1H), 0.93 (d, J = 6.9 Hz, 3H) ppm. ^{13}C NMR (101 MHz, D_2O) δ: 174.1, 139.5, 129.1, 128.8, 126.6, 58.6, 38.6, 36.0, 13.2 ppm. IR: 3459, 3021, 2970, 2944, 1738, 1580, 1514, 1453, 1366, 1228 cm^{-1}. HRMS (ESI): calcd. For $C_{11}H_{14}NO_2Na_2^+$ $[M-H+2Na]^+$: 238.0820; found: 238.0820.

LC-MS analysis [Kromasil 100-5-C18 column; MeCN/H_2O/formic acid = 5/95/0.1 (0–0.5 min), 5/95/0.1 to 40/60/0.1 (0.5–20 min), 40/60/0.1 to 60/40/0.1 (20–40 min), 60/40/0.1 to 95/5/0.1 (40–

42 min), 95/5/0.1 (42–43 min), 95/5/0.1 to 5/95/0.1 (43–44 min), 5/95/0.1 (44–45 min); 0.7 mL/min; 40 ℃; ESI-negative; 330 nm) indicated >20:1 d.r.: t_R = 23.9 (major diastereomer), 24.2 (enantiomer 1 of the minor diastereomer), 26.7 (enantiomer 2 of the major diastereomer), and 27.2 (enantiomer 2 of the minor diastereomer) min. HPLC analysis using Marfey's method indicated >99:1 e.r. for the major diastereomer.

Derivatization of 4b with DL-Marfey's reagent:

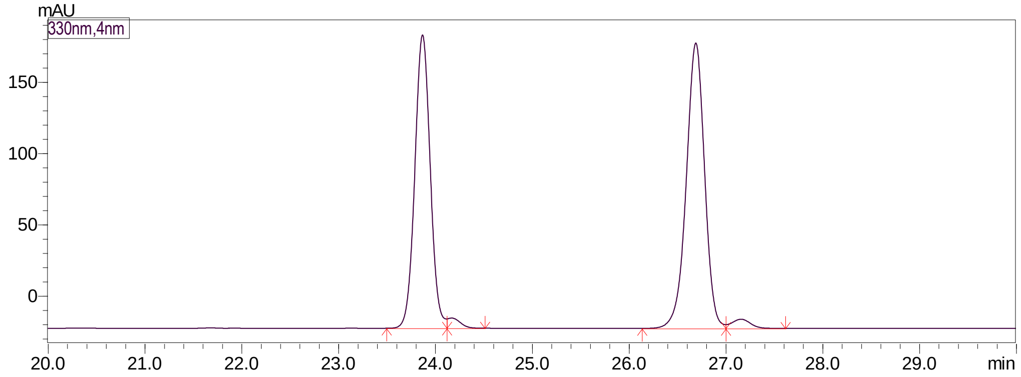

Peak#	Ret. Time	Area	Area %	Height
1	23.871	2329851	45.945	205775
2	24.173	14207	0.280	1966
3	26.693	2640180	52.065	200105
4	27.161	86694	1.710	6422

Derivatization of 4b with L-Marfey's reagent:

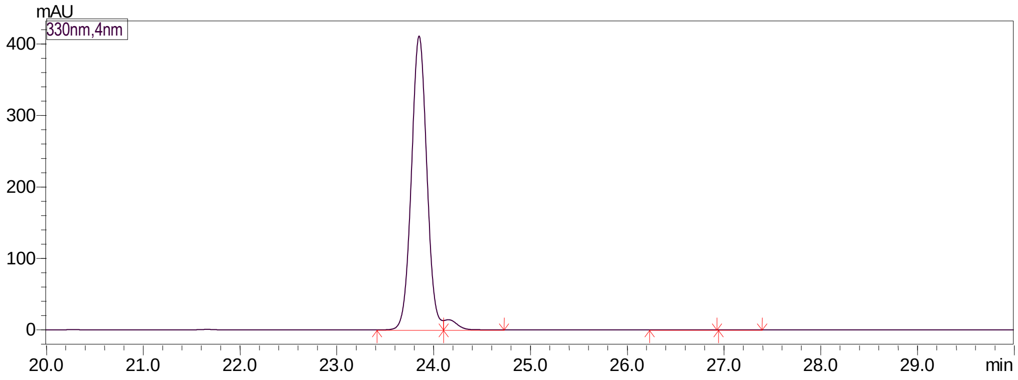

Peak#	Ret. Time	Area	Area %	Height
1	23.855	4553419	97.036	411151
2	24.159	134405	2.864	14204
3	26.658	3403	0.073	169
4	27.150	1302	0.028	106

Derivatization of 4b with D-Marfey's reagent:

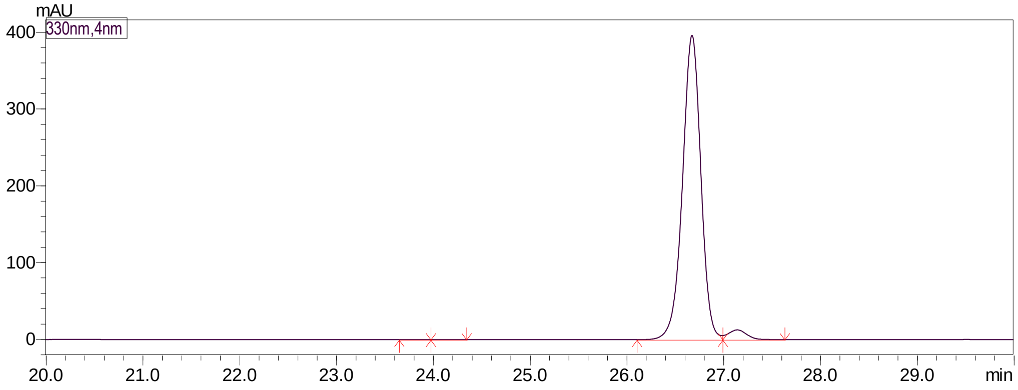

Peak#	Ret. Time	Area	Area %	Height
1	23.859	859	0.016	81
2	24.122	1450	0.027	123
3	26.677	5227451	96.846	396189
4	27.146	167952	3.112	12600

X. HPLC/GC calibration curves of asymmetric alkylation products

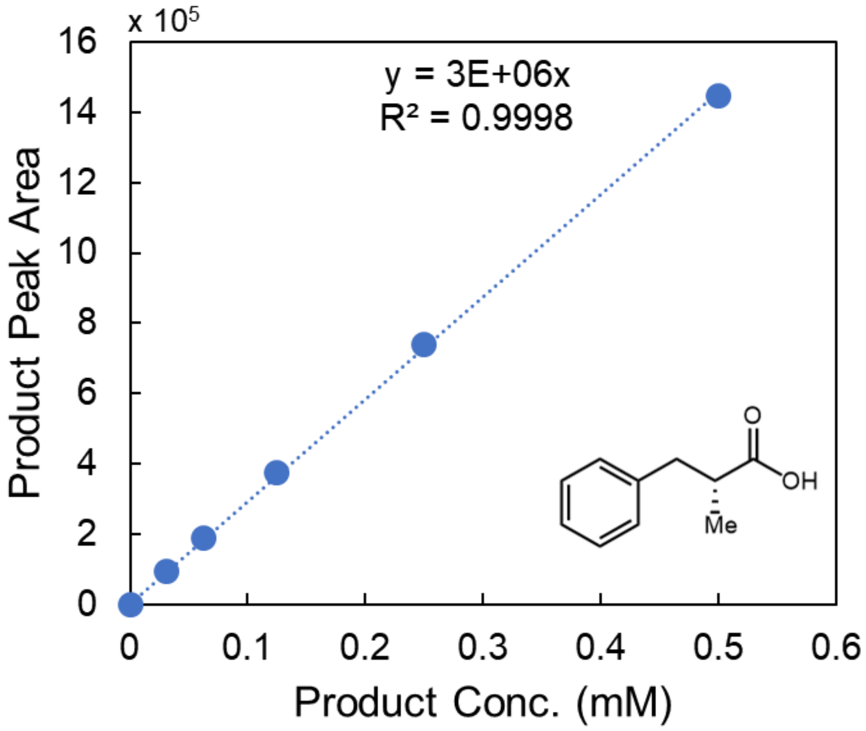

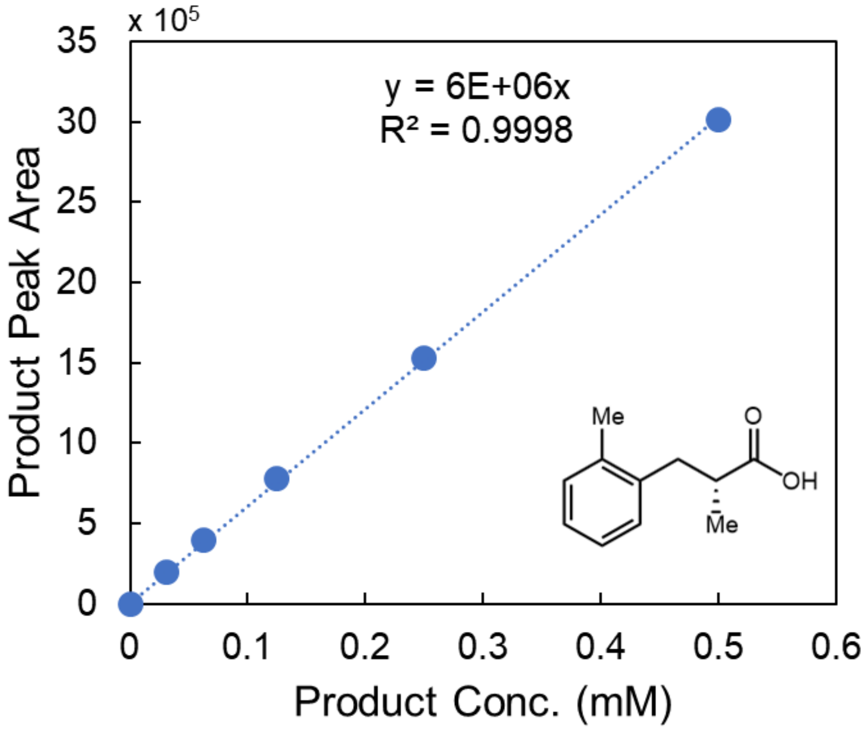

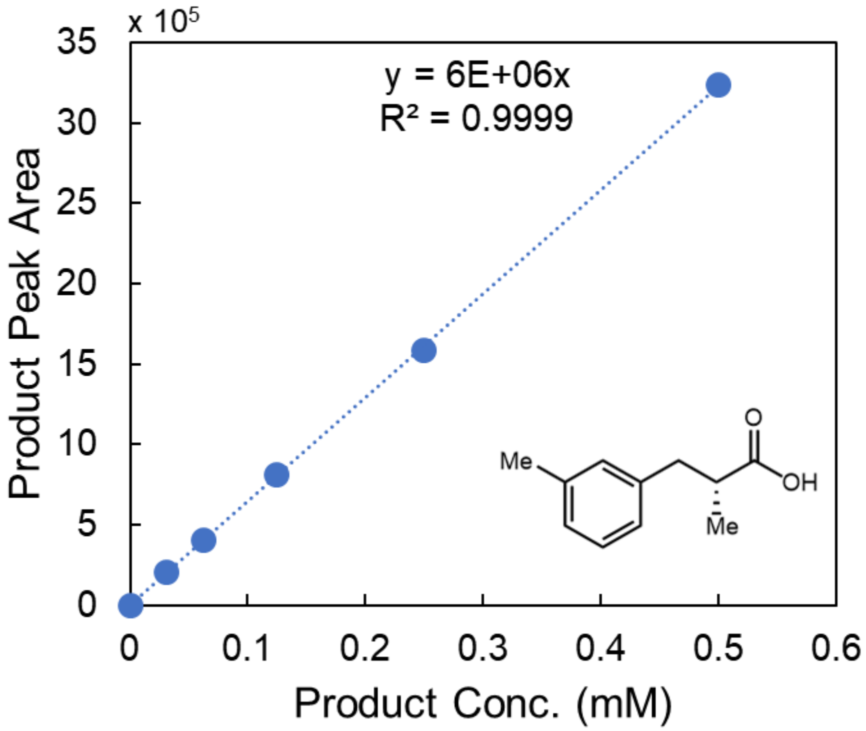

x 10^5
35
30
25
20
15
10
5
0
Product Peak Area
y = 6E+06x
R² = 0.9999
Me
O
OH
Me
0
0.1
0.2
0.3
0.4
0.5
0.6
Product Conc. (mM)

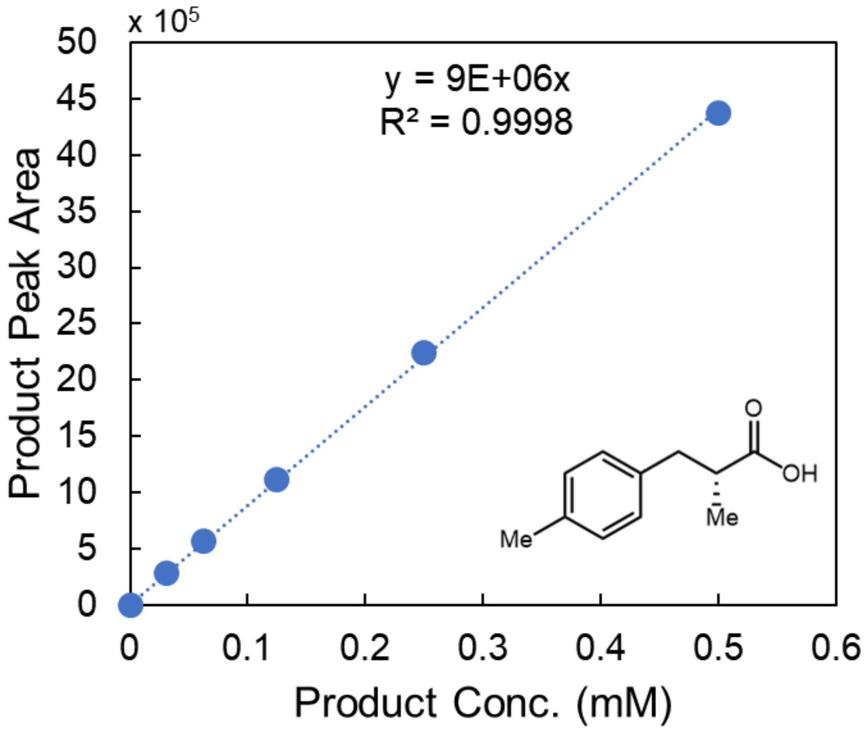

x 10^5
50
45
40
35
30
25
20
15
10
5
0
Product Peak Area
y = 9E+06x
R² = 0.9998
O
OH
Me
Me
0
0.1
0.2
0.3
0.4
0.5
0.6
Product Conc. (mM)

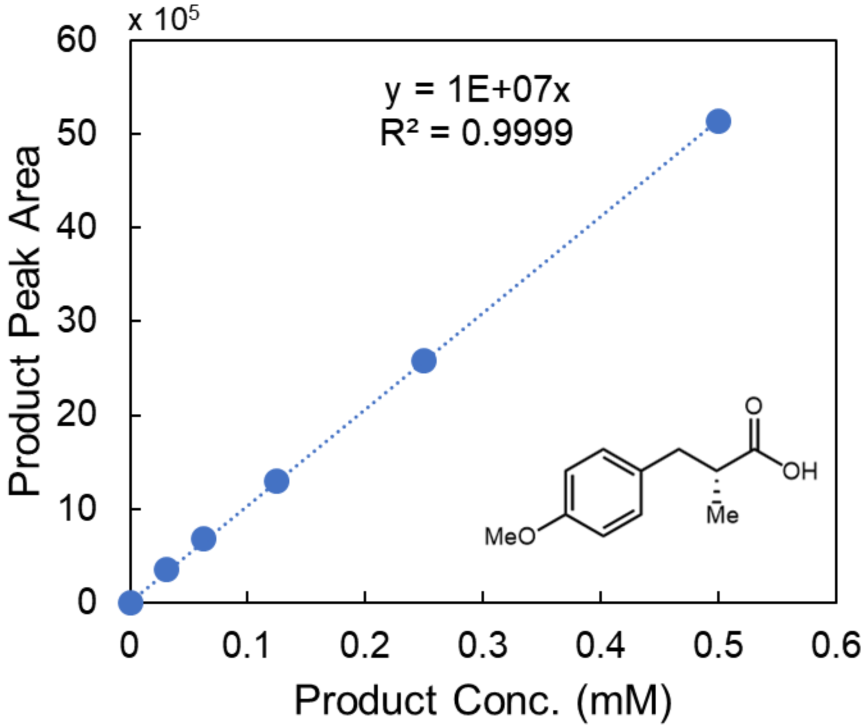
x 10^5
y = 1E+07x
R² = 0.9999
Product Peak Area
Product Conc. (mM)
0
10
20
30
40
50
60
0
0.1
0.2
0.3
0.4
0.5
0.6
MeO
O
OH
Me

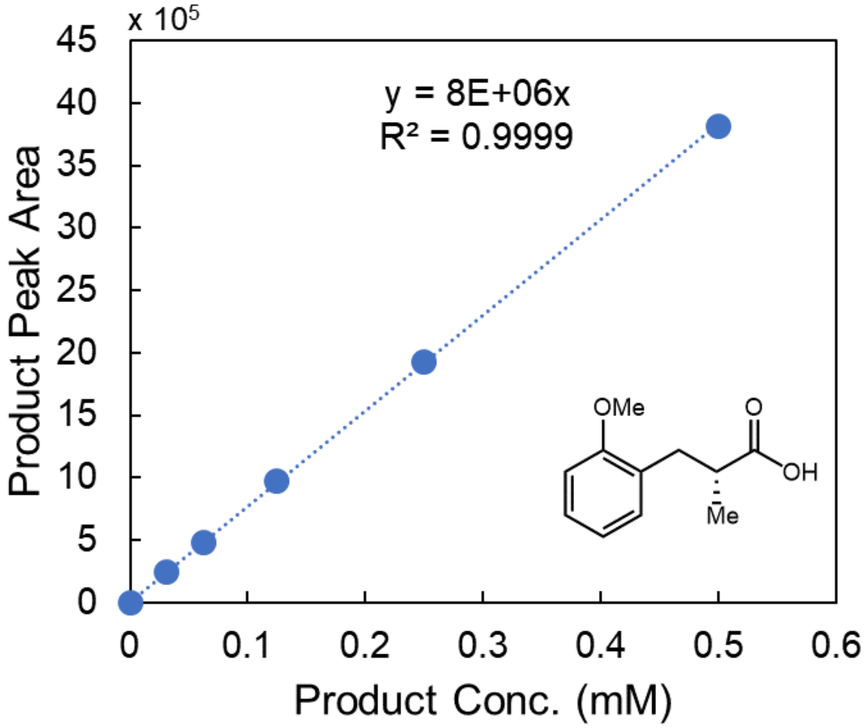
x 10^5
y = 8E+06x
R² = 0.9999
Product Peak Area
Product Conc. (mM)
0
5
10
15
20
25
30
35
40
45
0
0.1
0.2
0.3
0.4
0.5
0.6
OMe
O
OH
Me

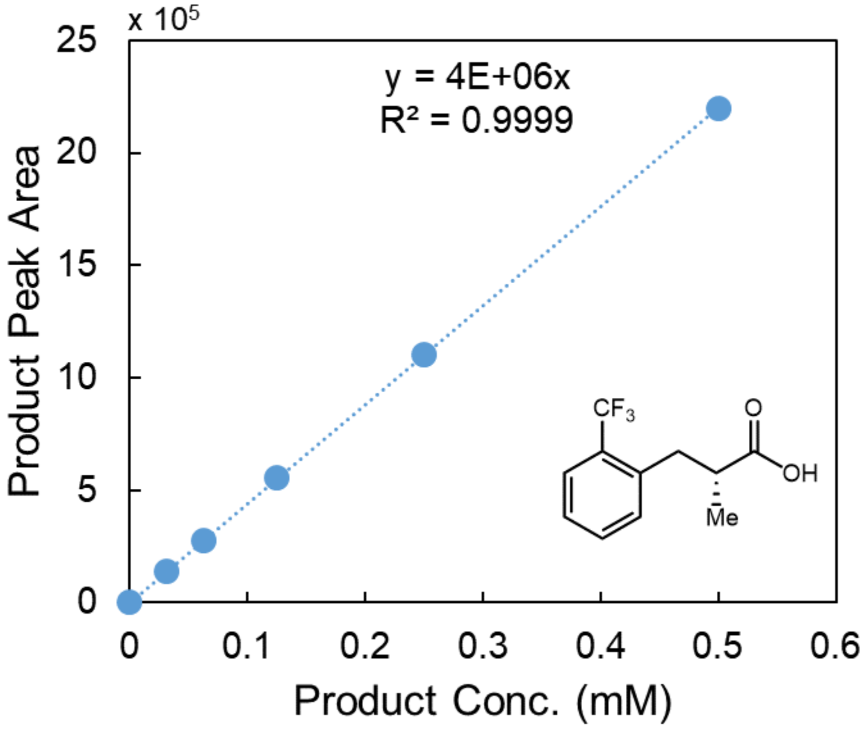
x 10^5
25
20
15
10
5
0
Product Peak Area
y = 4E+06x
R² = 0.9999
CF3
O
OH
Me
0
0.1
0.2
0.3
0.4
0.5
0.6
Product Conc. (mM)

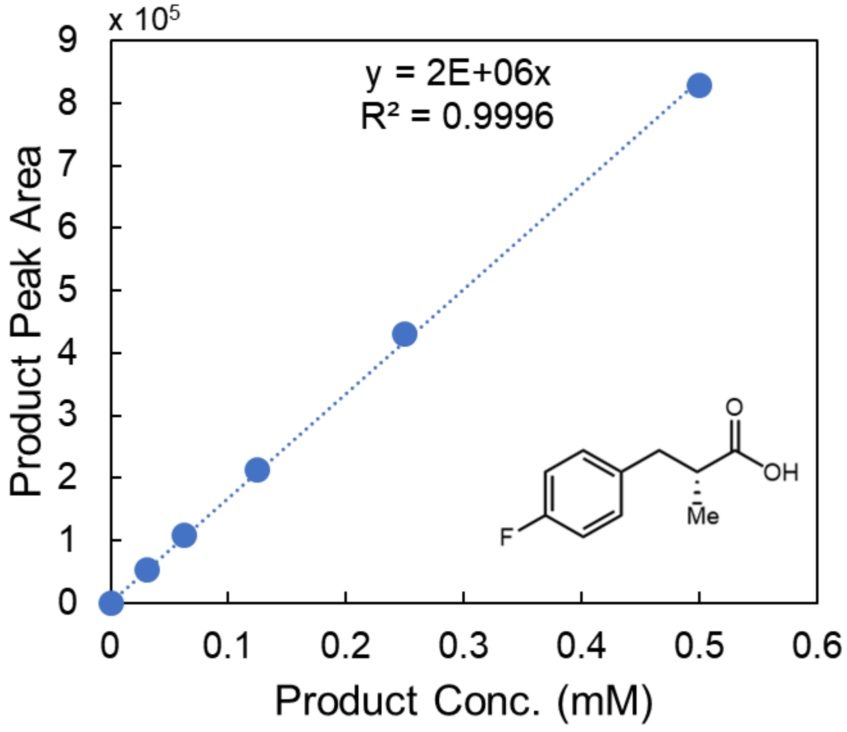
x 10^5
9
8
7
6
5
4
3
2
1
0
Product Peak Area
y = 2E+06x
R² = 0.9996
O
OH
F
Me
0
0.1
0.2
0.3
0.4
0.5
0.6
Product Conc. (mM)

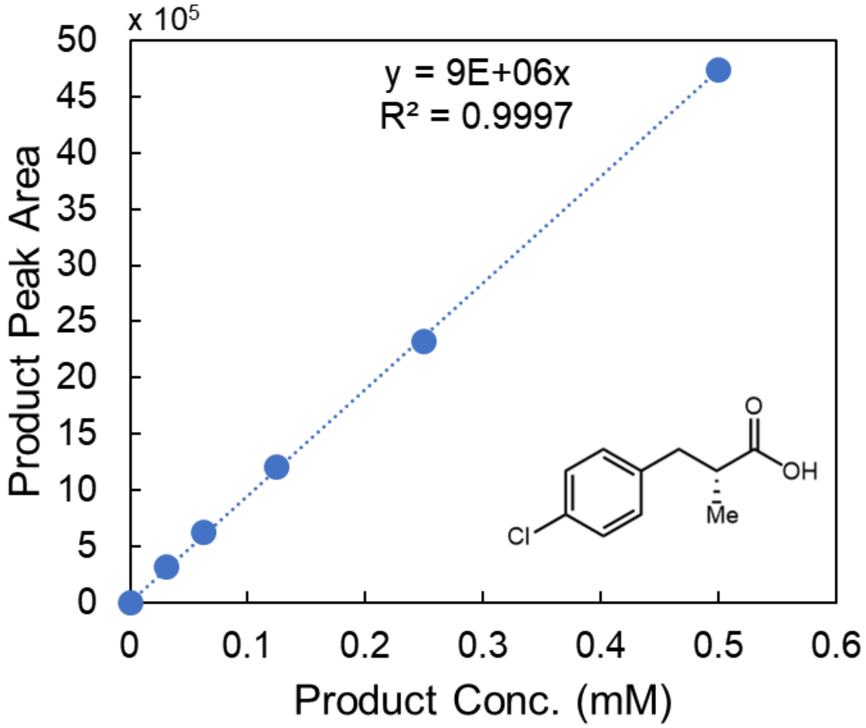
x 10^5
y = 9E+06x
R² = 0.9997
Product Peak Area
Product Conc. (mM)
0
5
10
15
20
25
30
35
40
45
50
0
0.1
0.2
0.3
0.4
0.5
0.6
O
OH
Me
Cl

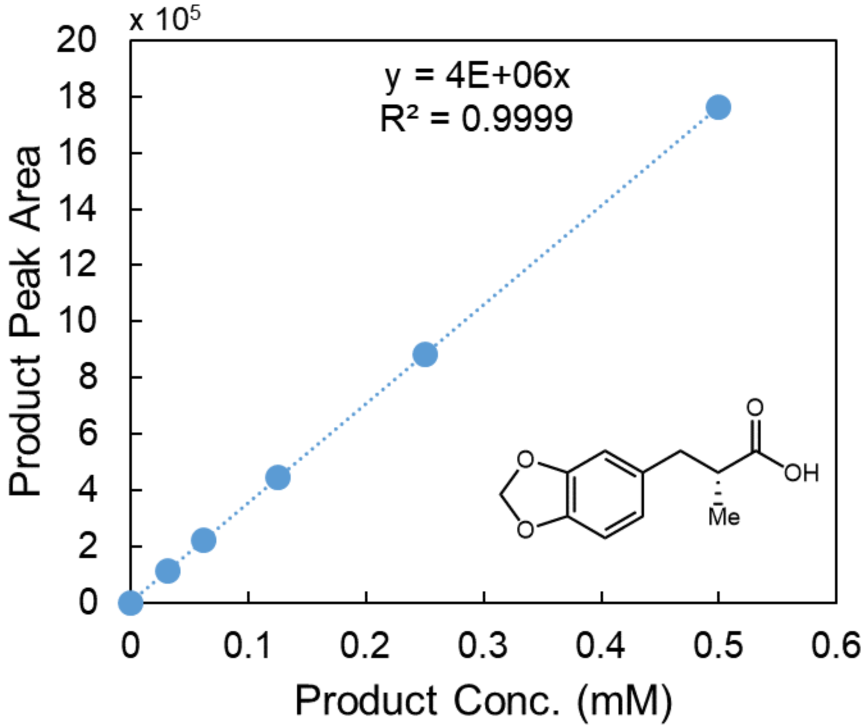
x 10^5
y = 4E+06x
R² = 0.9999
Product Peak Area
Product Conc. (mM)
0
2
4
6
8
10
12
14
16
18
20
0
0.1
0.2
0.3
0.4
0.5
0.6
O
O
O
OH
Me

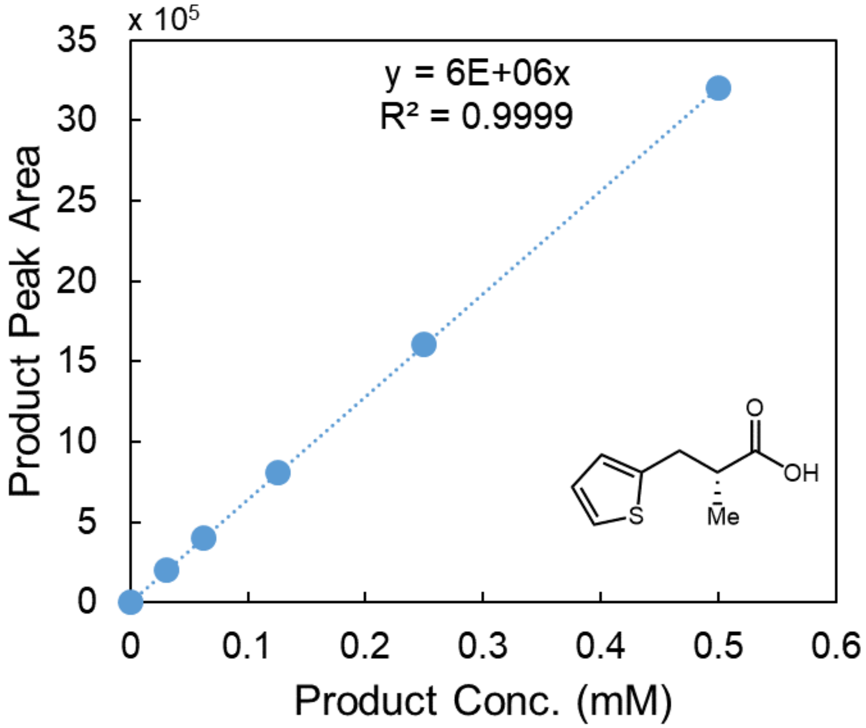

x 10^5
y = 6E+06x
R² = 0.9999
Product Peak Area
Product Conc. (mM)
0
5
10
15
20
25
30
35
0.1
0.2
0.3
0.4
0.5
0.6
O
OH
S
Me

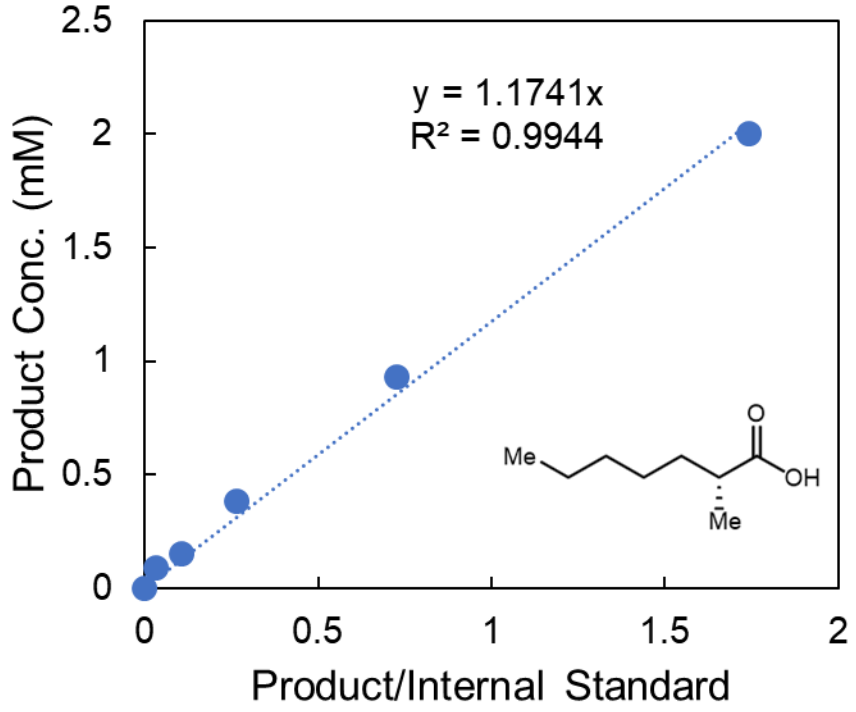

y = 1.1741x
R² = 0.9944
Product Conc. (mM)
Product/Internal Standard
0
0.5
1
1.5
2
2.5
Me
O
OH
Me

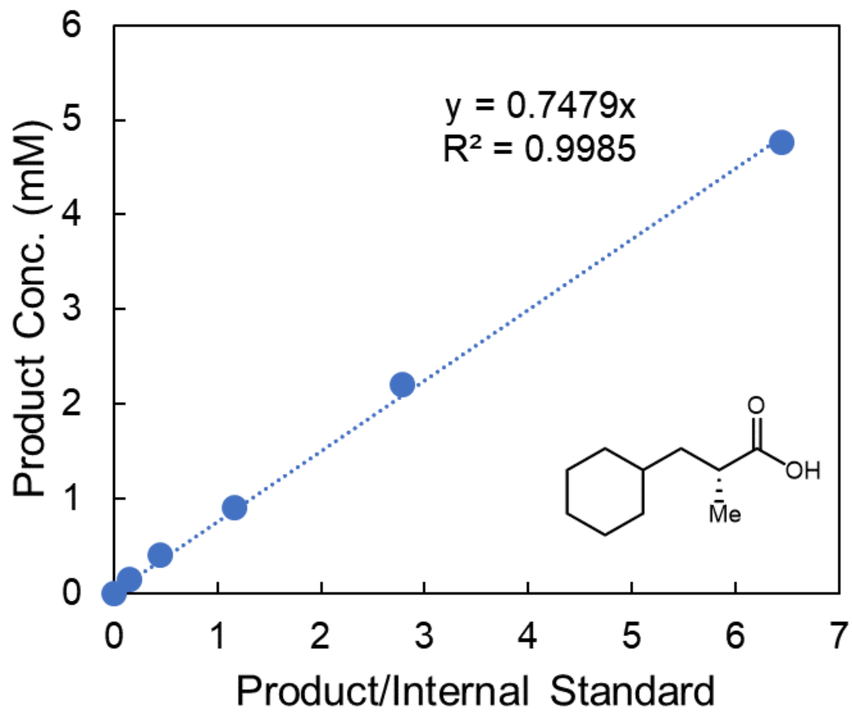

y = 0.7479x
R² = 0.9985
Product Conc. (mM)
Product/Internal Standard
0
1
2
3
4
5
6
7
O
OH
Me

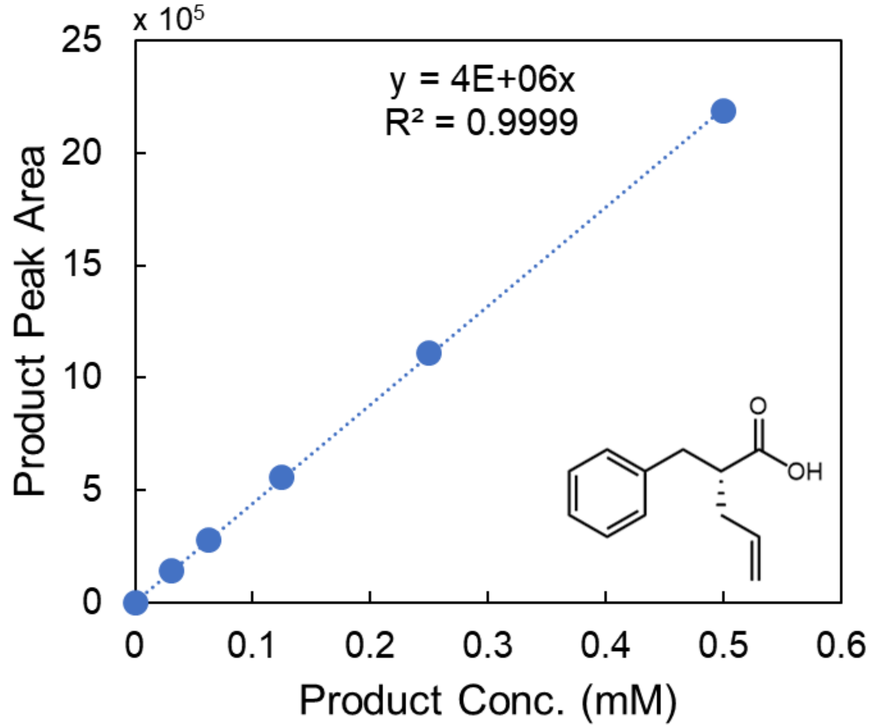

x 10^5
y = 4E+06x
R² = 0.9999
Product Peak Area
Product Conc. (mM)
0
5
10
15
20
25
0.1
0.2
0.3
0.4
0.5
0.6
O
OH

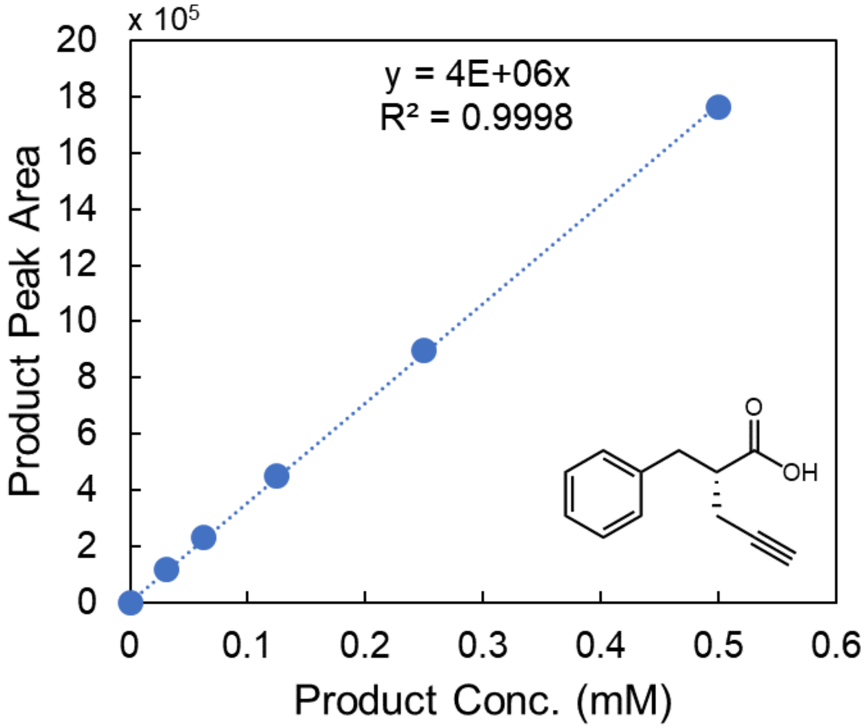
x 10^5
y = 4E+06x
R² = 0.9998
Product Peak Area
Product Conc. (mM)
O
OH
0
2
4
6
8
10
12
14
16
18
20
0
0.1
0.2
0.3
0.4
0.5
0.6

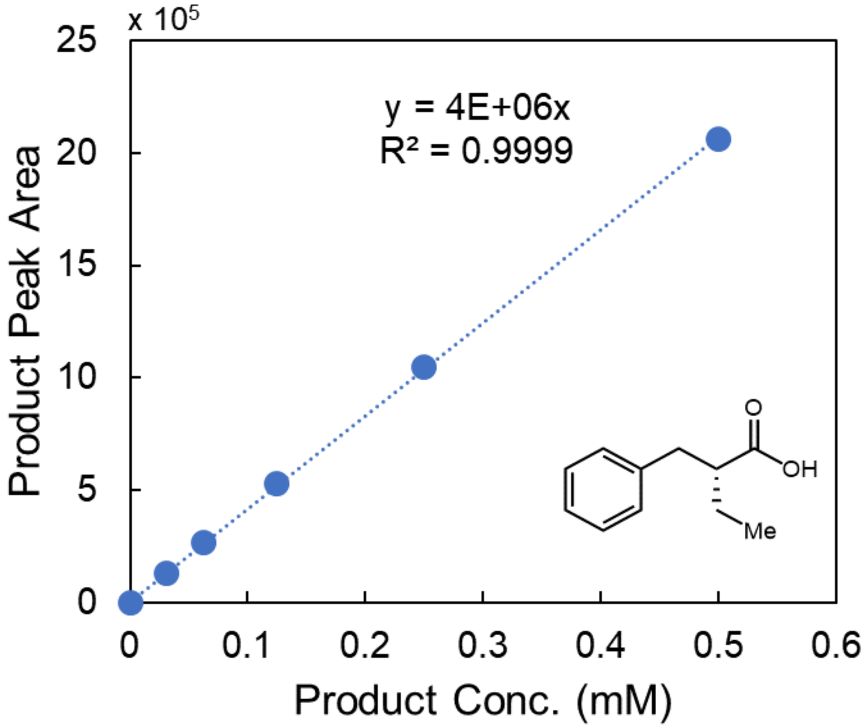
x 10^5
y = 4E+06x
R² = 0.9999
Product Peak Area
Product Conc. (mM)
O
OH
Me
0
5
10
15
20
25
0
0.1
0.2
0.3
0.4
0.5
0.6

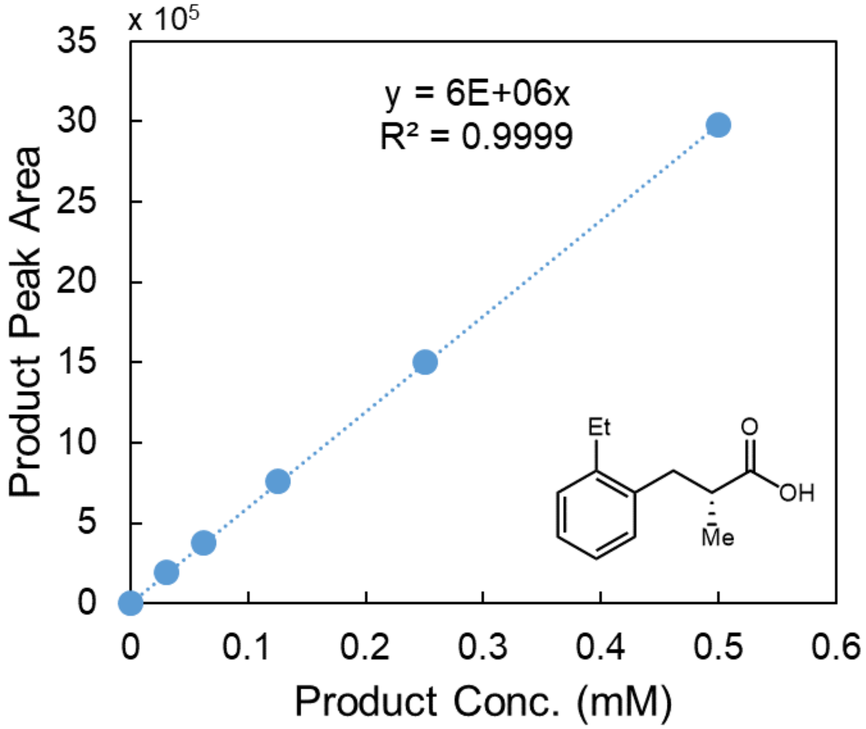
x 10^5
35
30
25
20
15
10
5
0
Product Peak Area
y = 6E+06x
R² = 0.9999
Et
O
OH
Me
0
0.1
0.2
0.3
0.4
0.5
0.6
Product Conc. (mM)

XI. Chiral HPLC and GC traces

(*R*)-2-methyl-3-phenylpropanoic acid (3a)

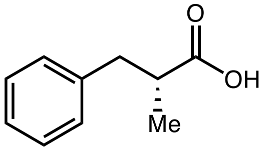

Racemic 3a:

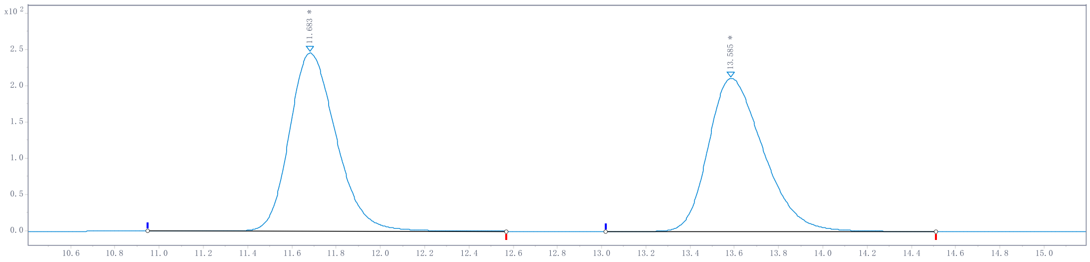

Peak#	Ret. Time	Area	Area %	Height
1	11.683	3604.979	49.737	245.708
2	13.585	3643.082	50.263	211.333

Enantioenriched 3a using SgvM^{VAV}: 99:1 e.r.

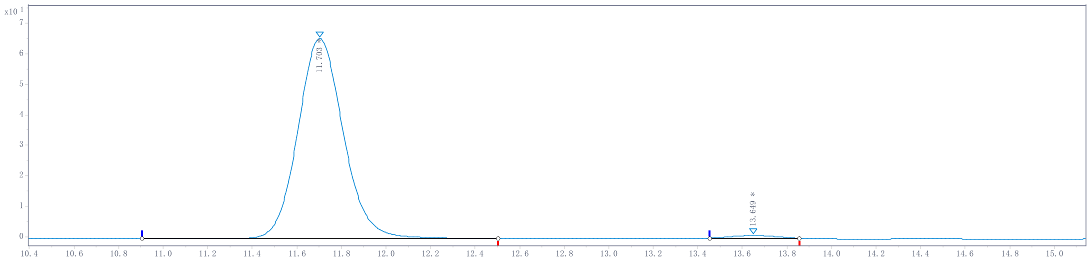

Peak#	Ret. Time	Area	Area %	Height
1	11.703	927.574	98.710	65.595
2	13.649	12.122	1.290	0.931

(*R*)-2-methyl-3-(*o*-tolyl)propanoic acid (3b)

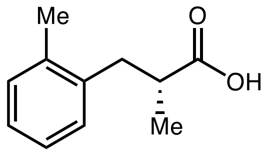

Racemic 3b:

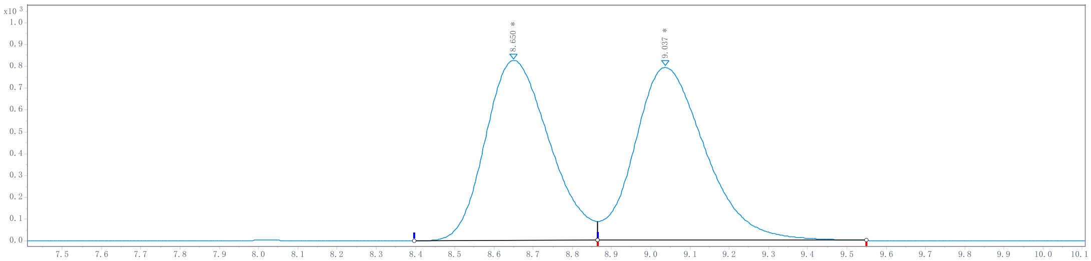

Peak#	Ret. Time	Area	Area %	Height
1	8.650	9362.171	48.632	824.465
2	9.037	9889.038	51.368	790.848

Enantioenriched 3b using SgvM^{VAV}: > 99:1 e.r.

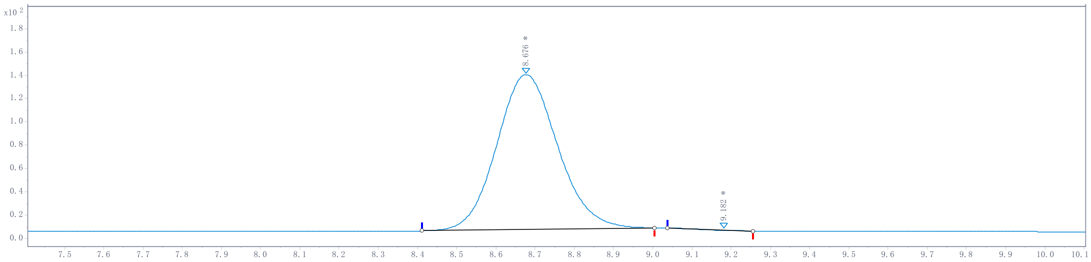

Peak#	Ret. Time	Area	Area %	Height
1	8.676	1433.949	99.870	132.789
2	9.182	1.870	0.130	0.368

(*R*)-2-methyl-3-(*m*-tolyl)propanoic acid (3c)

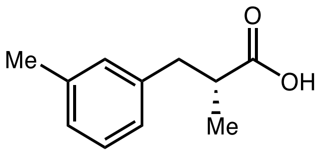

Racemic 3c:

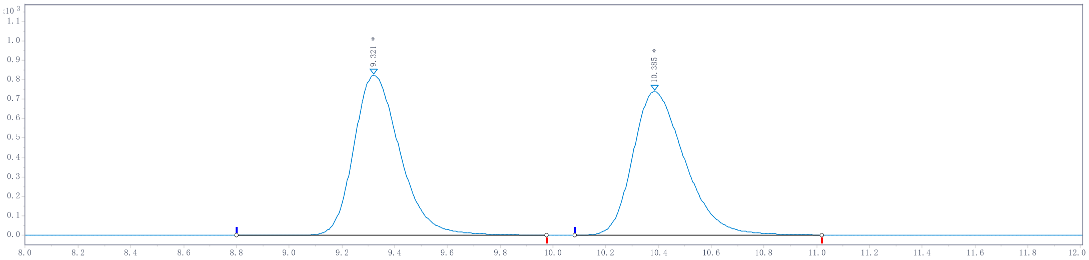

Peak#	Ret. Time	Area	Area %	Height
1	9.321	9957.112	49.750	822.146
2	10.385	10057.254	50.250	736.853

Enantioenriched 3c using SgvM^{VAV}: 98:2 e.r.

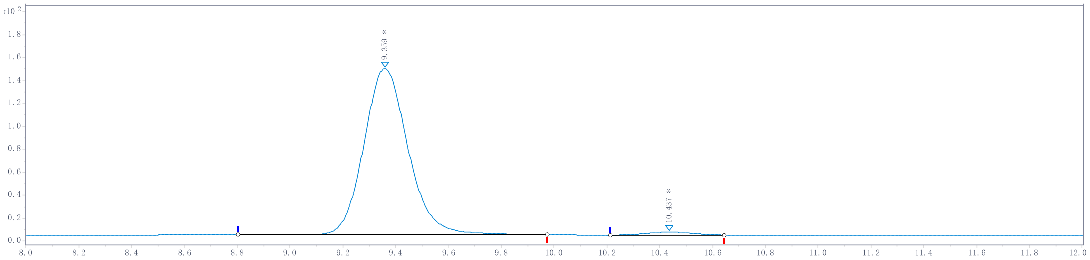

Peak#	Ret. Time	Area	Area %	Height
1	9.359	1681.736	98.397	144.961
2	10.437	27.390	1.603	2.363

(*R*)-2-methyl-3-(*p*-tolyl)propanoic acid (3d)

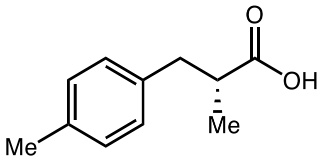

Racemic 3d:

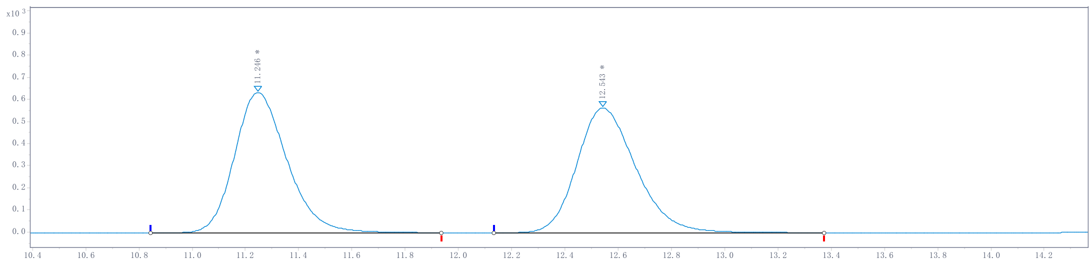

Peak#	Ret. Time	Area	Area %	Height
1	11.246	8694.767	49.955	634.101
2	12.543	8710.443	50.045	564.295

Enantioenriched 3d using SgvM^{VAV}: 99:1 e.r.

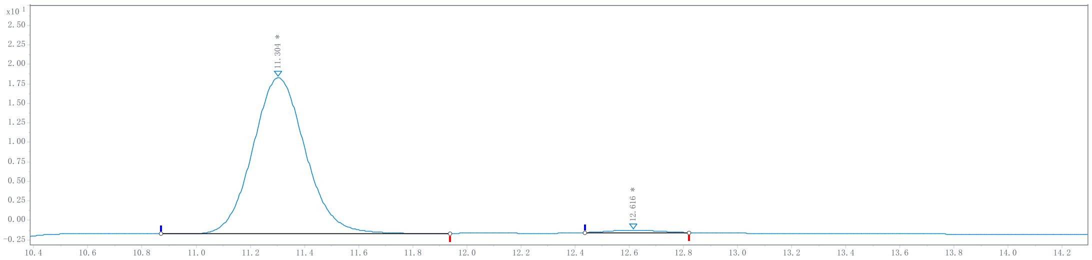

Peak#	Ret. Time	Area	Area %	Height
1	11.304	269.620	98.534	19.975
2	12.616	4.013	1.466	0.328

(*R*)-3-(4-methoxyphenyl)-2-methylpropanoic acid (3e)

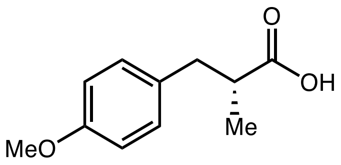

Racemic 3e:

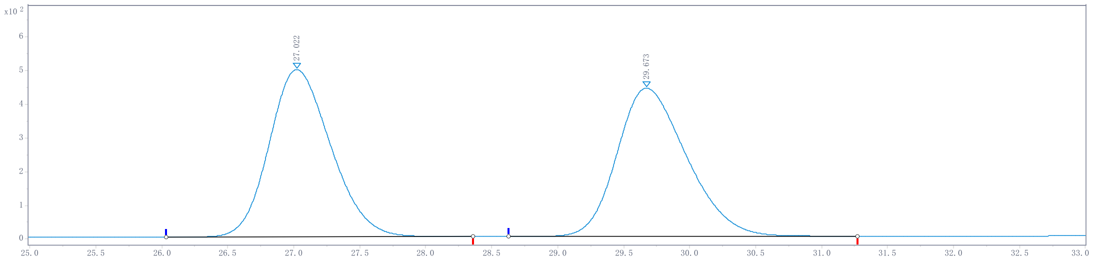

Peak#	Ret. Time	Area	Area %	Height
1	27.022	16108.904	49.985	495.444
2	29.673	16118.694	50.015	438.767

Enantioenriched 3e using SgvM^{VAV}: >99:1 e.r.

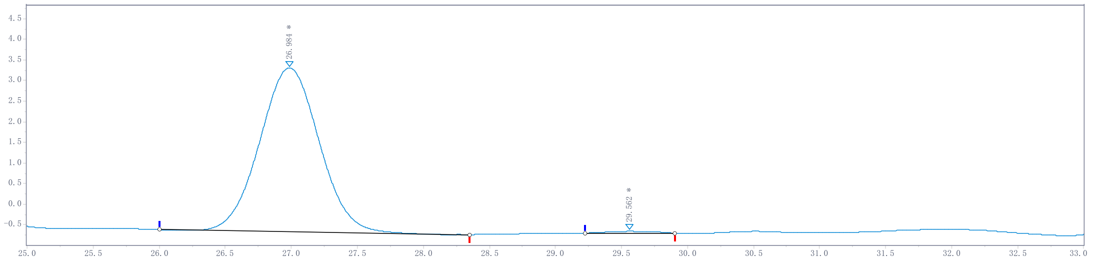

Peak#	Ret. Time	Area	Area %	Height
1	26.984	124.596	99.142	3.975
2	29.562	1.078	0.858	0.045

(*R*)-3-(2-methoxyphenyl)-2-methylpropanoic acid (3f)

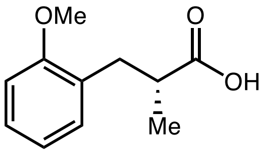

Racemic 3f:

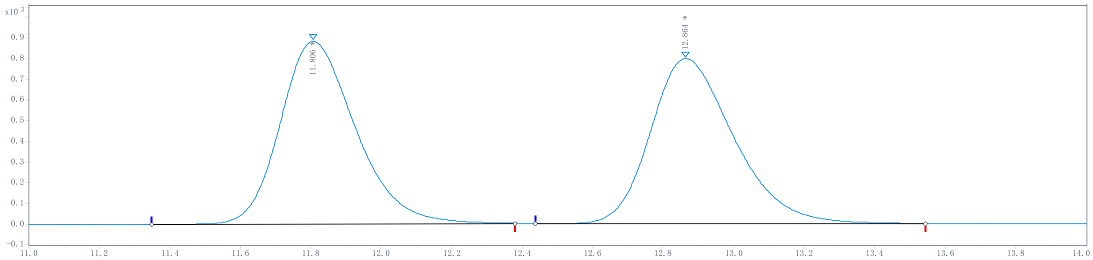

Peak#	Ret. Time	Area	Area %	Height
1	11.806	13070.542	49.961	881.464
2	12.864	13091.132	50.039	797.163

Enantioenriched 3f using SgvM^{VAV}: > 99:1 e.r.

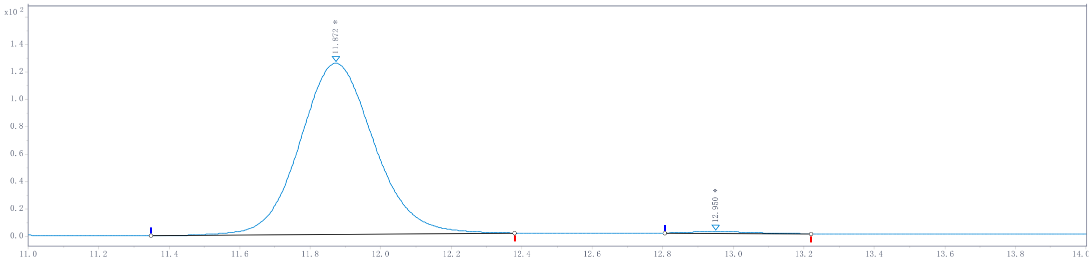

Peak#	Ret. Time	Area	Area %	Height
1	11.872	1832.694	99.223	125.016
2	12.950	14.356	0.777	1.158

(*R*)-2-methyl-3-(2-(trifluoromethyl)phenyl)propanoic acid (3g)

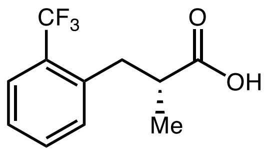

Racemic 3g:

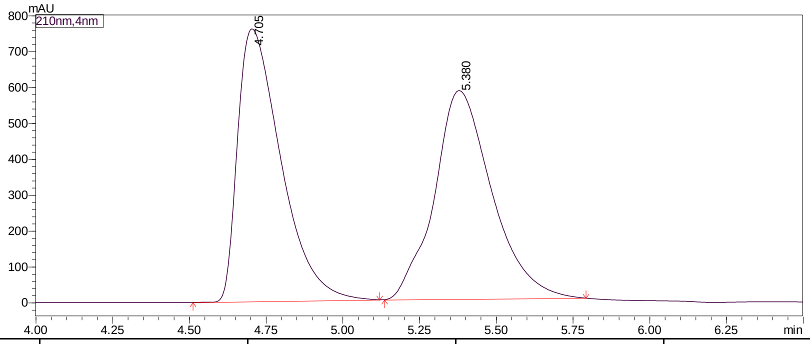

Peak#	Ret. Time	Area	Area %	Height
1	4.705	7439036	49.543	758444
2	5.380	7576224	50.457	580598

Enantioenriched 3g using SgvM^{VAV}: 98:2 e.r.

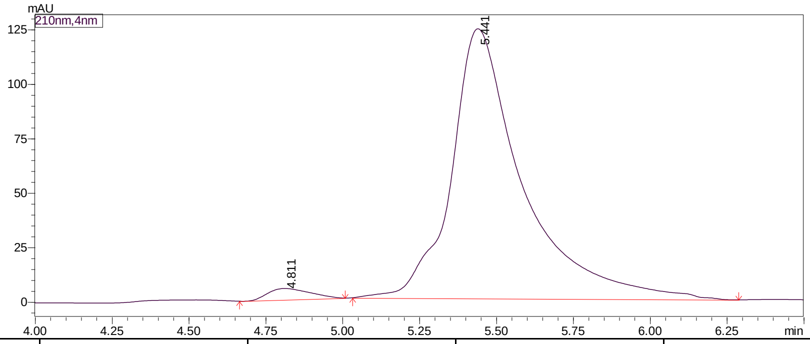

Peak#	Ret. Time	Area	Area %	Height
1	4.811	49342	2.406	5207
2	5.441	2001147	97.594	123752

(*R*)-3-(4-fluorophenyl)-2-methylpropanoic acid (3h)

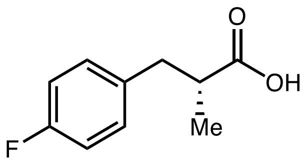

Racemic 3h:

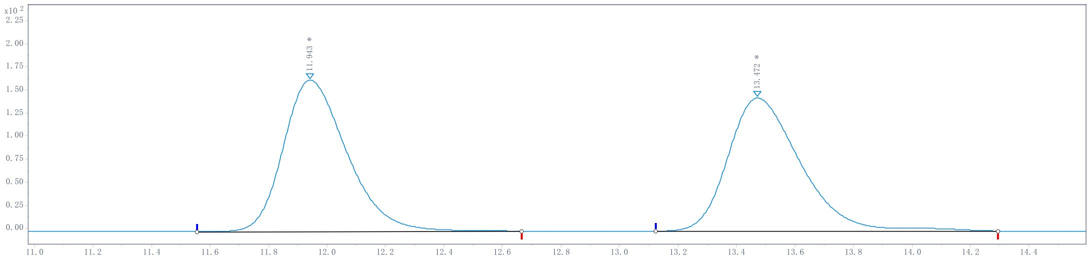

Peak#	Ret. Time	Area	Area %	Height
1	11.943	2486.757	50.076	164.112
2	13.472	2479.227	49.924	143.980

Enantioenriched 3h using SgvM^{VAV}: 98:2 e.r.

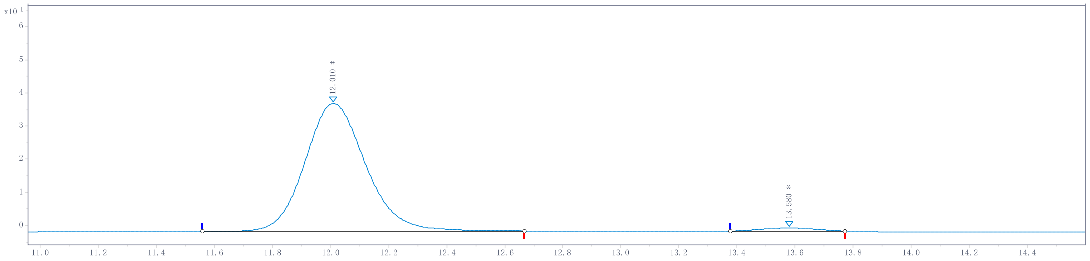

Peak#	Ret. Time	Area	Area %	Height
1	12.010	555.166	98.114	38.483
2	13.580	10.674	1.886	0.832

(*R*)-3-(4-chlorophenyl)-2-methylpropanoic acid (3i)

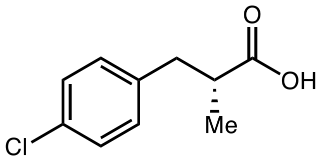

Racemic 3i:

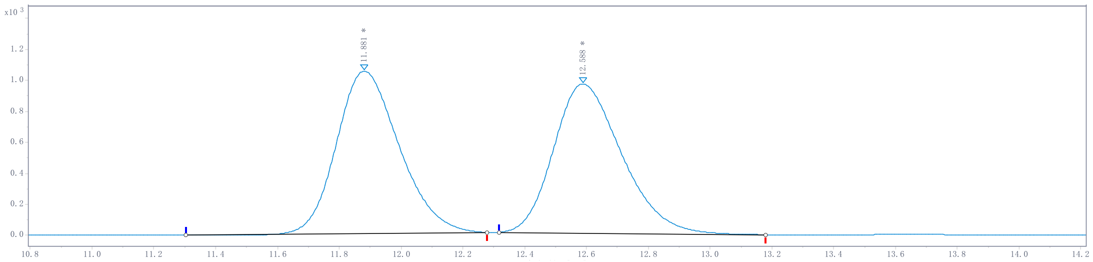

Peak#	Ret. Time	Area	Area %	Height
1	11.881	14817.757	50.028	1049.136
2	12.588	14801.346	49.972	963.152

Enantioenriched 3i using SgvM^{VAV}: 99:1 e.r.

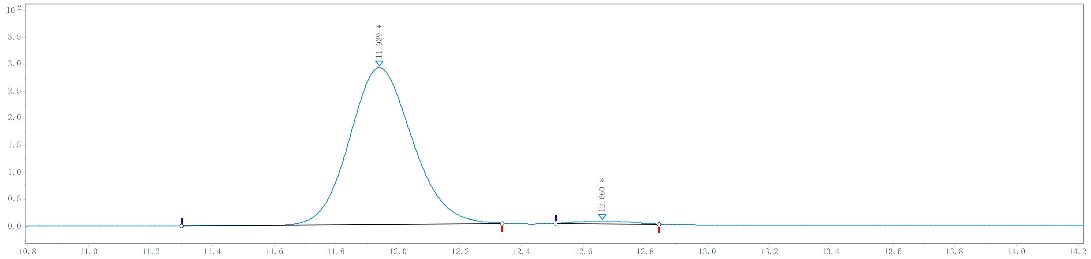

Peak#	Ret. Time	Area	Area %	Height
1	11.939	4063.839	98.617	289.454
2	12.660	56.990	1.383	5.107

(*R*)-3-(benzo[*d*][1,3]dioxol-5-yl)-2-methylpropanoic acid (3j)

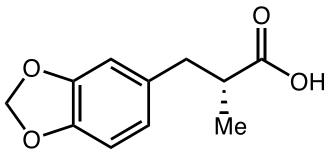

Racemic 3j:

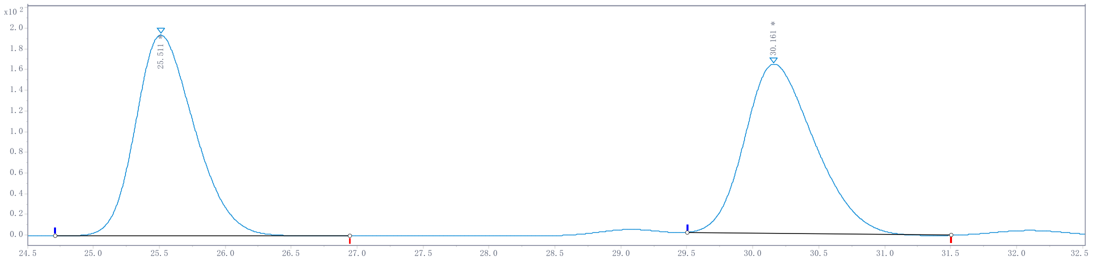

Peak#	Ret. Time	Area	Area %	Height
1	25.511	6052.981	50.164	194.065
2	30.161	6013.310	49.836	163.490

Enantioenriched 3j using SgvM^{VAV}: 98:2 e.r.

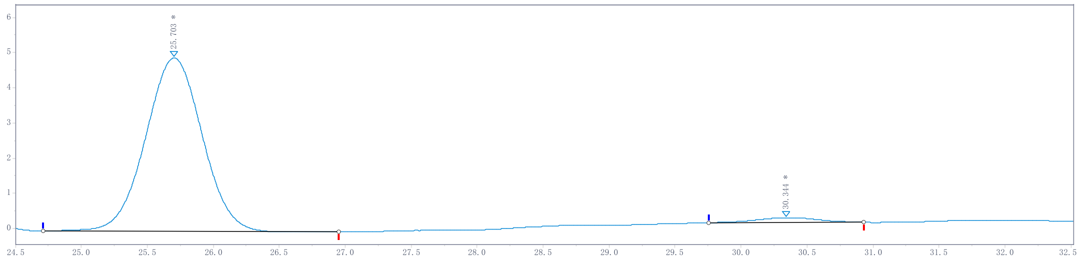

Peak#	Ret. Time	Area	Area %	Height
1	25.703	146.456	97.751	4.921
2	30.344	3.369	2.249	0.137

(*R*)-2-methyl-3-(thiophen-2-yl)propanoic acid (3k)

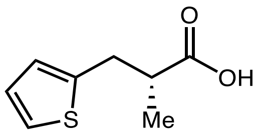

Racemic 3k:

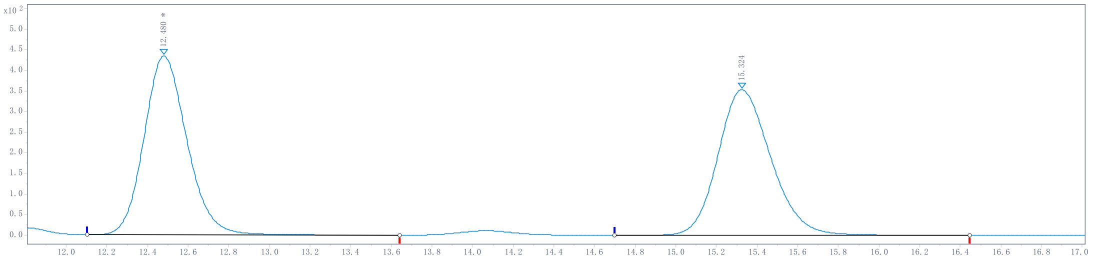

Peak#	Ret. Time	Area	Area %	Height
1	12.480	6373.168	49.968	434.375
2	15.324	6381.245	50.032	353.429

Enantioenriched 3k using SgvM^{VAV}: 98:2 e.r.

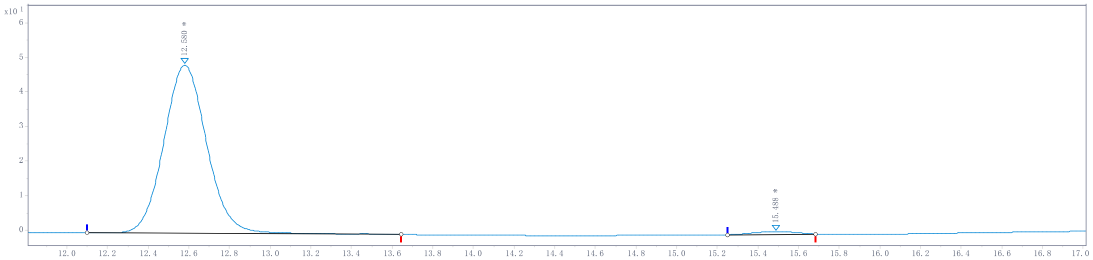

Peak#	Ret. Time	Area	Area %	Height
1	12.580	715.760	98.406	48.632
2	15.488	11.594	1.594	0.823

(*R*)-2-methylheptanoic acid (3l)

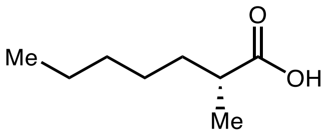

Racemic 3l (GC):

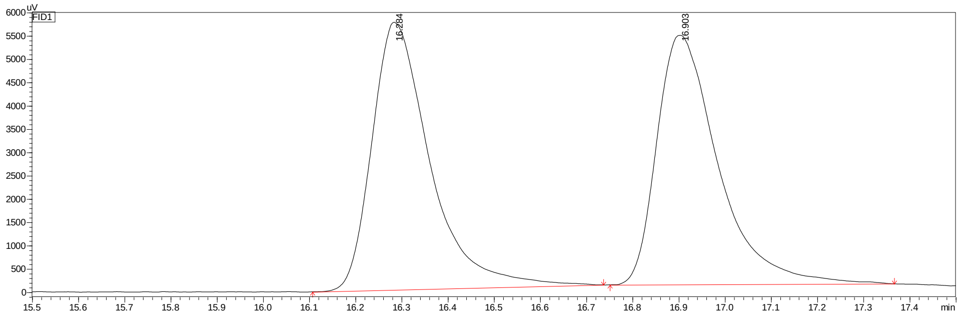

Peak#	Ret. Time	Area	Area %	Height
1	16.284	50193	50.422	5735
2	16.903	49352	49.578	5346

Enantioenriched 3l using SgvM^{VAV} (GC): 99:1 e.r.

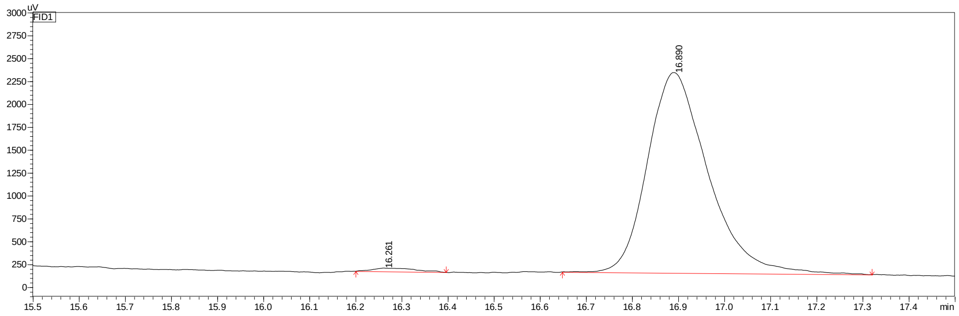

Peak#	Ret. Time	Area	Area %	Height
1	16.261	246	1.230	37
2	16.890	19960	98.770	2188

(*R*)-3-cyclohexyl-2-methylpropanoic acid (3m)

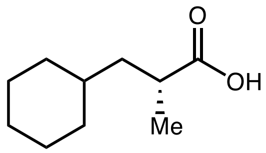

Racemic 3m (GC):

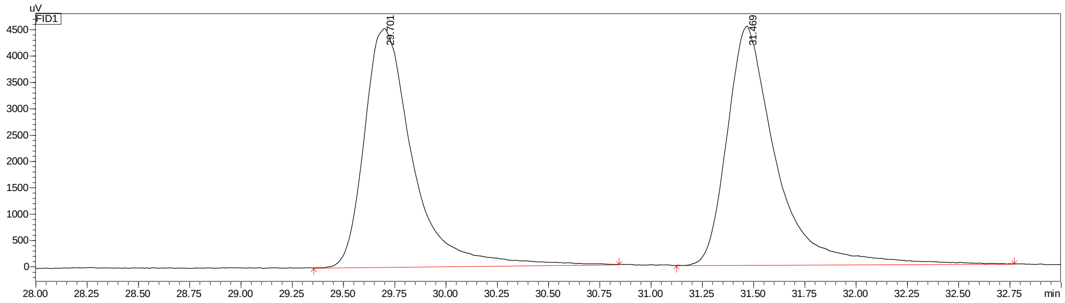

Peak#	Ret. Time	Area	Area %	Height
1	29.701	74066	49.864	4524
2	31.469	74470	50.136	4531

Enantioenriched 3m using SgvM^{VAV} (GC): 99:1 e.r.

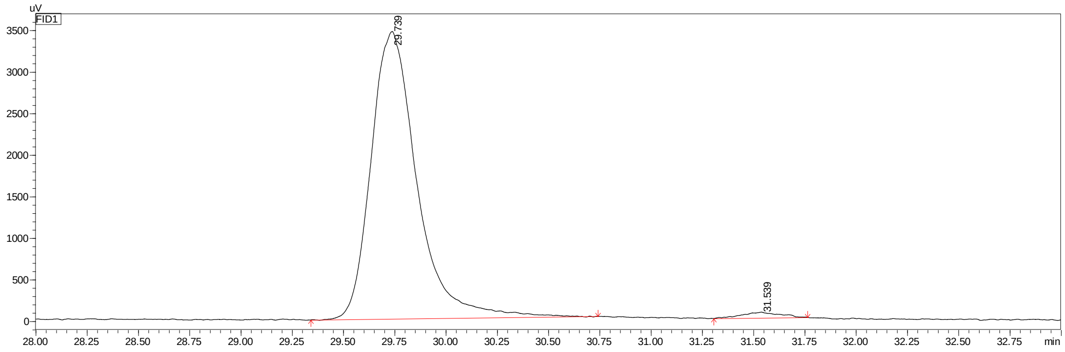

Peak#	Ret. Time	Area	Area %	Height
1	29.739	53116	98.688	3448
2	31.539	706	1.312	62

(*R*)-2-benzylpent-4-enoic acid (3n)

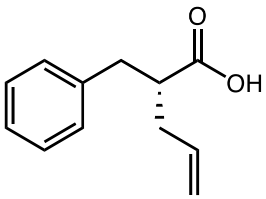

Racemic 3n:

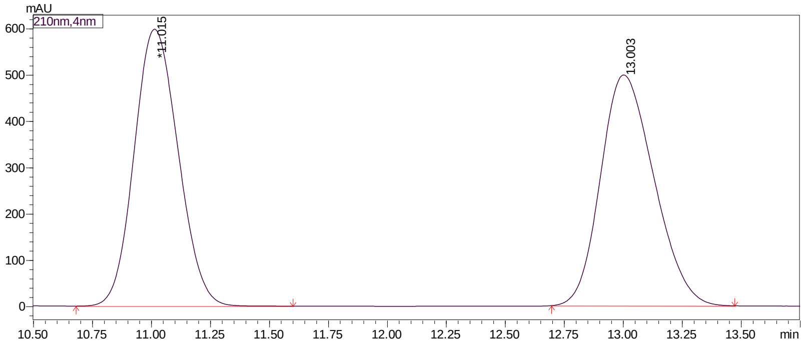

Peak#	Ret. Time	Area	Area %	Height
1	11.015	7761281	49.695	597950
2	13.003	7856427	50.305	498193

Enantioenriched 3n using SgvM^{VAV}: 98:2 e.r.

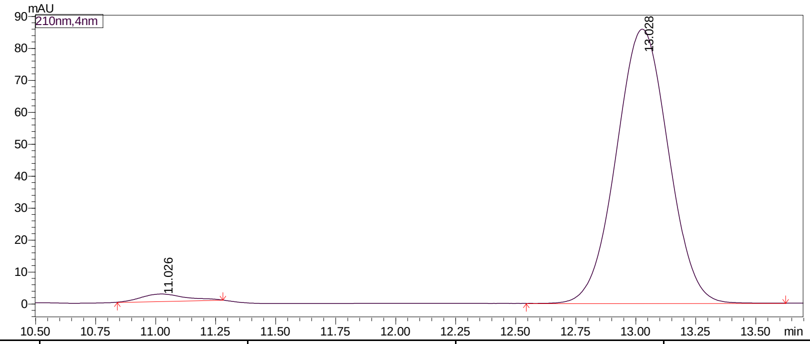

Peak#	Ret. Time	Area	Area %	Height
1	11.026	28532	2.154	2270
2	13.028	1296183	97.846	85787

(*R*)-2-benzylpent-4-ynoic acid (3o)

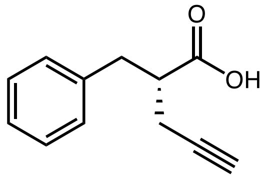

Racemic 3o:

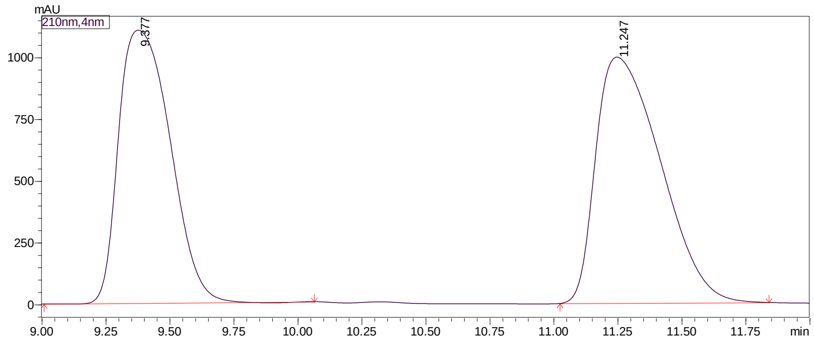

Peak#	Ret. Time	Area	Area %	Height
1	9.377	15469133	49.581	1104939
2	11.247	15730348	50.419	996077

Enantioenriched 3o using SgvM^{VAV}: 97:3 e.r.

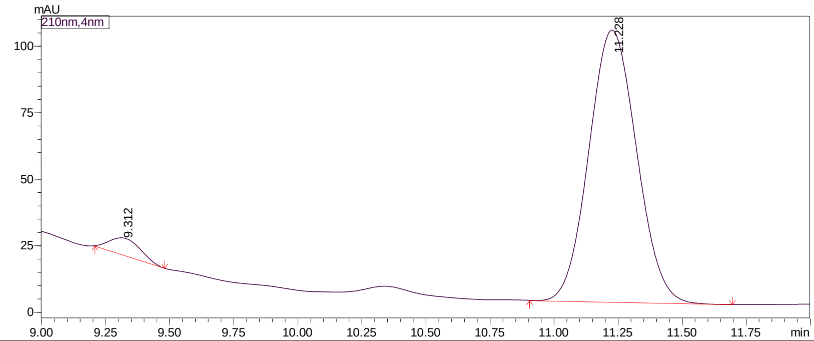

Peak#	Ret. Time	Area	Area %	Height
1	9.312	46423	3.389	6205
2	11.228	1323568	96.611	102139

(*R*)-2-benzylbutanoic acid (3p)

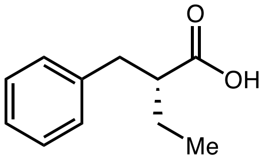

Racemic 3p:

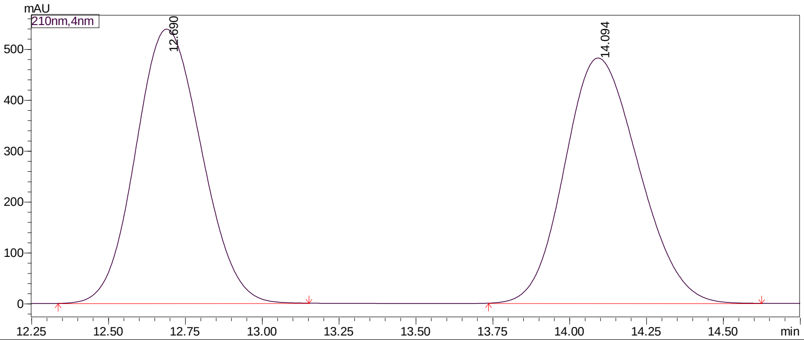

Peak#	Ret. Time	Area	Area %	Height
1	12.690	8026863	49.808	538095
2	14.094	8088805	50.192	481547

Enantioenriched 3p using SgvM^{VAV}: 99:1 e.r.

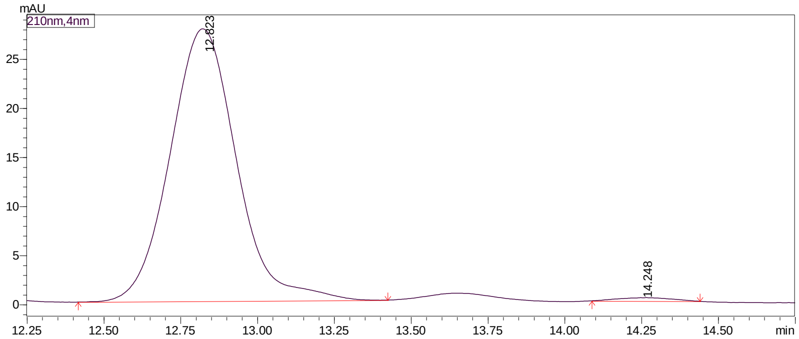

Peak#	Ret. Time	Area	Area %	Height
1	12.823	416393	98.999	27749
2	14.248	4210	1.001	341

(R)-3-(2-ethylphenyl)-2-methylpropanoic acid

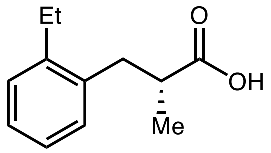

Racemic sample:

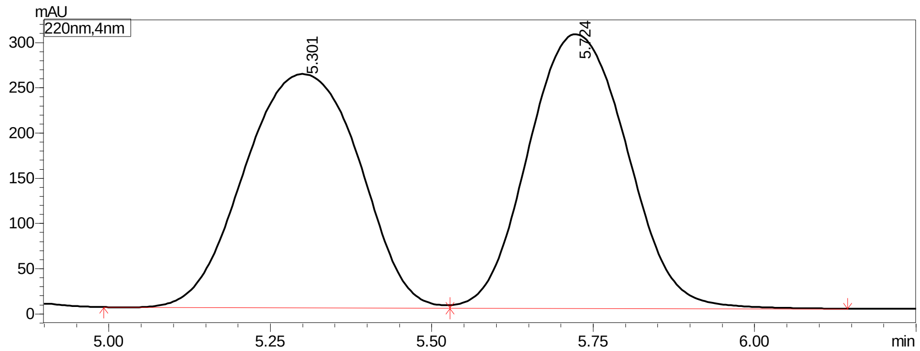

Peak#	Ret. Time	Area	Area %	Height
1	5.301	3198238	49.819	258102
2	5.724	3221447	50.181	302761

Enantioenriched sample using SgvM^{VAV}: 97:3 e.r.

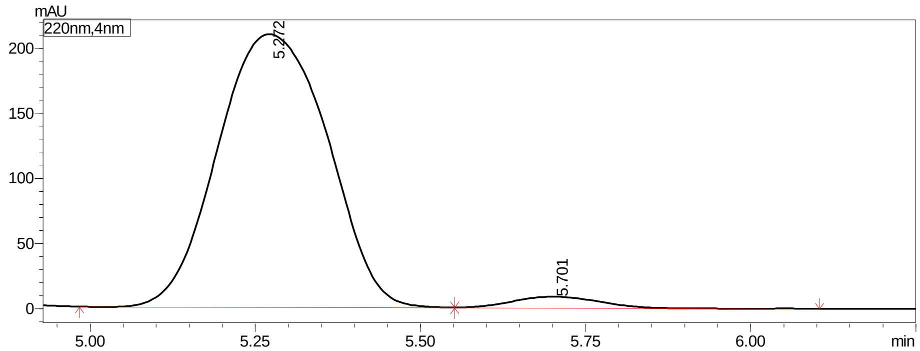

Peak#	Ret. Time	Area	Area %	Height
1	5.272	2428714	96.985	209946
2	5.701	75506	3.015	8642

XII. Protein crystallization and structural determination

Expression, purification, and crystallization of SgvM and variant. The pET-28a-His_6-SgvM and the triple variant $SgvM^{VAV}$ overexpression vector was used to transform *E. coli* Rosetta™(DE3) cells for protein expression. Transformed cells were grown in LB media (supplemented with kanamycin) at 37 °C to OD_{600} = 0.6 - 0.8. Cultures were cooled in an ice bath for 10 min before induction with IPTG at a final concentration of 0.4 mM. Cells were allowed to grow overnight at 18 °C before harvesting. The cell pellet was resuspended in suspension buffer (500 mM NaCl, 20 mM Tris pH 8.0, 10% glycerol).

Cells were lysed by sonication, and the lysates were clarified by centrifugation at 10,000 *g* for 30 min. The supernatant was then passed through a 5 mL HisTrap HP column (Cytiva Life Sciences) pre-equilibrated with wash buffer (1 M NaCl, 20 mM Tris pH 8.0 and 30 mM imidazole). The column was washed with 8 column volumes (CV) of wash buffer, and His_6-SgvM or the triple variant was eluted from the column on an ÄKTAprime plus system (Cytiva Life Sciences) using a linear gradient from 30 mM imidazole to 250 mM imidazole over 8 CV at a flow rate of 2 mL/min. The purest fractions as judged by SDS-PAGE were pooled and concentrated down to 5 mL prior to injection onto an 16/60 Superdex® 200 column (Cytiva Life Sciences) equilibrated in 300 mM KCl, and 20 mM Tris pH 7.5. Fractions containing pure protein were pooled and concentrated to ~10 mg/mL.

Initial crystallization conditions were determined using sitting-drop sparse matrix screening (using the Crystal Gryphon LCP, Art Robbins Instruments). Diffraction quality crystals were obtained by the hanging drop method at 9 °C. Briefly, 1 μL protein solution, was mixed with 1 μL precipitant solution (100 mM Tris pH 8.0, 250 mM lithium sulfate, and 30% (w/v) polyethylene glycol 3,350) and equilibrated against a reservoir of the precipitant solution. Crystals appeared over the course of three to five days.

Structure determination of SgvM and complexes with bound ligands. Fully grown crystals were vitrified by direct immersion into liquid nitrogen. All crystallographic measurements were collected at Sector 21-ID (LS-CAT, Advanced Photon Source, Argonne National Labs, IL). Data was indexed, scaled, and integrated using autoPROC[16]. Crystallographic phases of SgvM were obtained by single wavelength anomalous diffraction methods using data collected from a crystal

soaked with methyl mercury bromide [17]. Phases were determined using Crank2[18] as implemented in CCP4 (initial Figure of Merit of 0.511). The initial solvent flattened and NCS averaged map was of sufficient quality to permit the building of a preliminary model using Parrot/Buccaneer[19]. The structure was further improved through rounds of manual rebuilding using Coot[20], interspersed with refinement using REFMAC5[21]. Phases for the triple variant and ligand bound structures were determined using a preliminary structure of the unliganded wild-type SgvM as a starting model. The initial models were refined against the structure factors using REFMAC5[21], and subsequently manually rebuilt using Coot[20]. Models for ligands were only added after the free R factors[22] were below 0.30. For crystallographic and data collection statistics, see Supplementary Table 5.

Supplementary Table 5. Crystallographic and data collection statistics

	w.t. SgvM SAM+α-ketoleucine	w.t. SgvM SAH+α-ketoleucine	$SgvM^{VAV}$ SAH+**1a**	$SgvM^{VAV}$ SAH+phenylpyruvate
**Data collection**				
Space Group	$P4_32_12$	$P4_32_12$	$P4_32_12$	$P4_32_12$
Cell: a, b, c (Å)	67.1, 67.1, 184.4	68.3, 68.3, 186.2	67.6, 67.6, 185.4	67.4, 67.4, 184.7
Resolution (Å)[1]	54.3 – 2.13 (2.14 – 2.13)	38.1 – 2.20 (2.21 – 2.20)	37.8 – 2.04 (2.043 – 2.04)	54.4 – 2.27 (2.73 – 2.27)
Total reflections	463,810 (4532)	502,426 (5110)	748,607 (6921)	317,364 (2618)
Unique reflections	23,766 (226)	23,134 (230)	28,470 (281)	20,125 (203)
R_{sym} (%)	9.8 (175.0)	11.3 (182.3)	7.2 (165.0)	12.4 (143.0)
R_{pim} (%)[2]	2.3 (39.3)	3.5 (39.4)	1.4 (33.5)	3.2 (41.2)
$CC_{1/2}$[2]	1.00 (0.753)	0.999 (0.792)	0.999 (0.820)	0.998 (0.877)
I/σ(I)	23.3 (2.1)	15.8 (1.5)	27.2 (2.1)	16.0 (2.0)
Completeness (%)	96.8 (100)	99.4 (100.0)	100 (100)	97.3 (100)
Redundancy	19.5 (20.1)	21.7 (22.2)	26.3 (24.6)	15.8 (12.9)
**Refinement**				
Resolution (Å)	25.0 – 2.13	25.0 – 2.2	25.0 – 2.04	25.0 – 2.27
No. reflections	22,528	21,977	26,939	19,142
R_{work} / R_{free}[3]	20.8 / 25.4	21.1 / 24.6	20.7 / 25.6	19.6 / 24.4
**No. of atoms**				
Protein	2,554	2,546	2,557	2,541
Cofactor/Ligand	20/10	26/10	26/14	26/13
Water	67	90	124	111
**B-factors**				
Protein	54.5	62.8	54.6	61.1
Cofactor/Ligand	67.5/53.4	53.9/60.5	53.3/55.1	60.4/48.4
Water	43.1	55.8	50.7	53.6
**R.m.s deviations**				
Bond lengths (Å)	0.006	0.004	0.005	0.006

Bond angles (°)	1.38	1.16	1.27	1.45

[1] Highest resolution shell is shown in parenthesis.

[2] Used as additional indicators of data quality for the highly redundant data sets reported here.

[3] R-factor = $\Sigma(|F_{obs}|-k|F_{calc}|)/\Sigma |F_{obs}|$and R-free is the R value for a test set of reflections consisting of a random 5% of the diffraction data not used in refinement.

XIII. ^{1}H, ^{19}F and ^{13}C NMR spectra of compounds

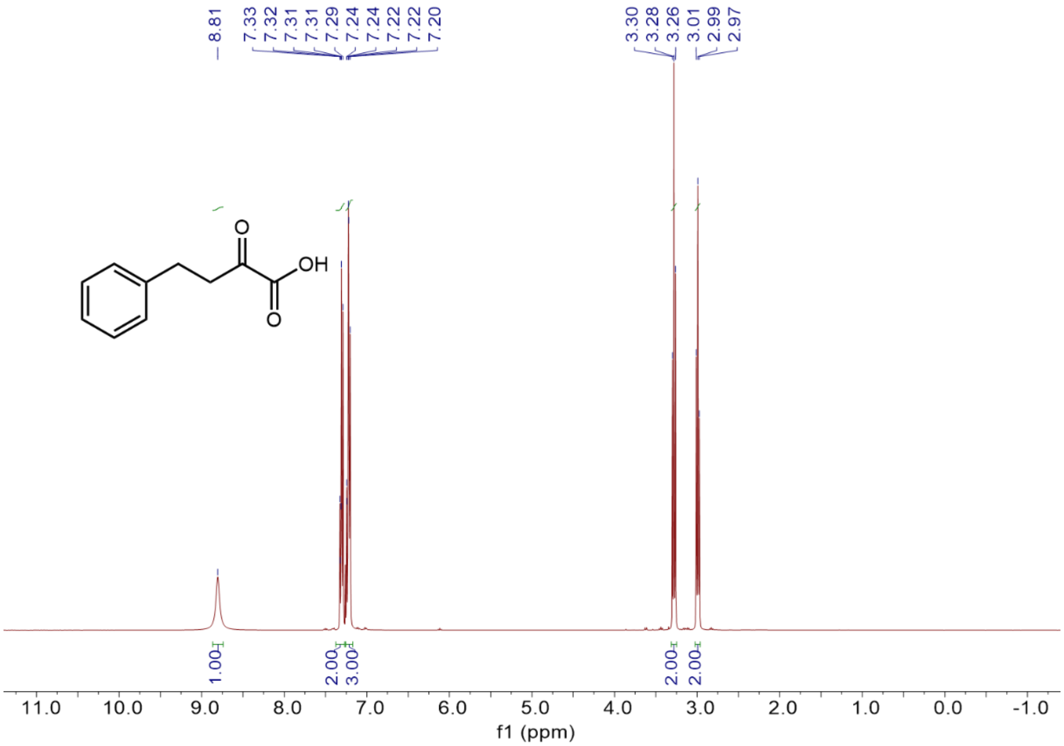

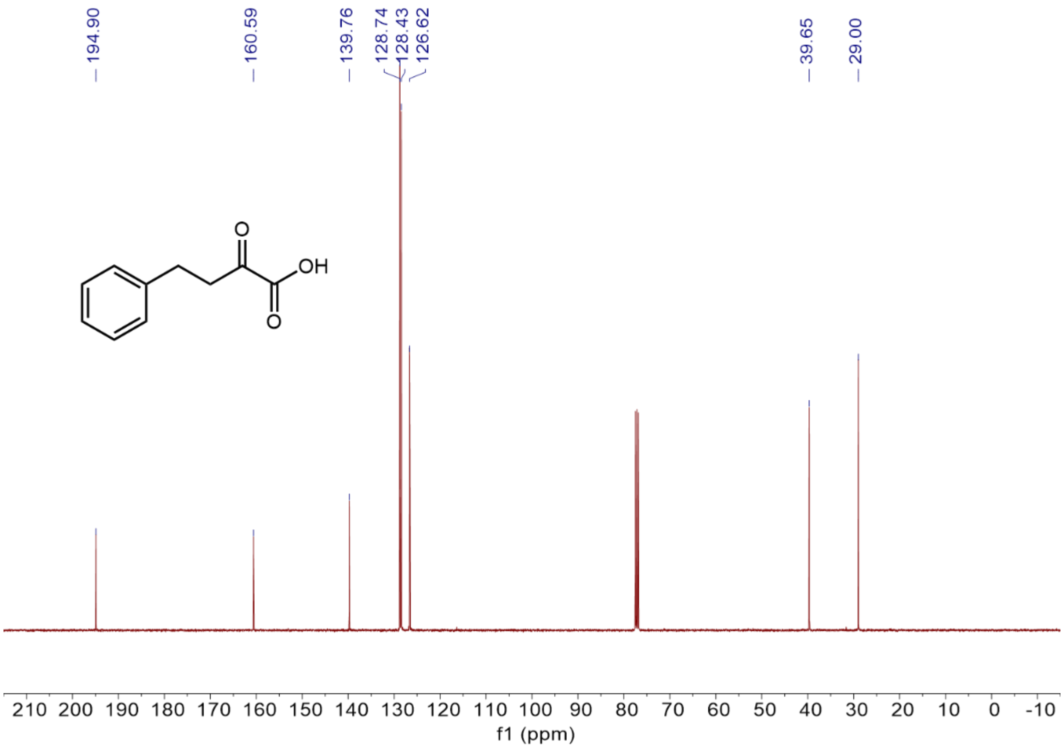

Supplementary Figure 11. NMR spectra of 2-oxo-4-phenylbutanoic acid (1a)

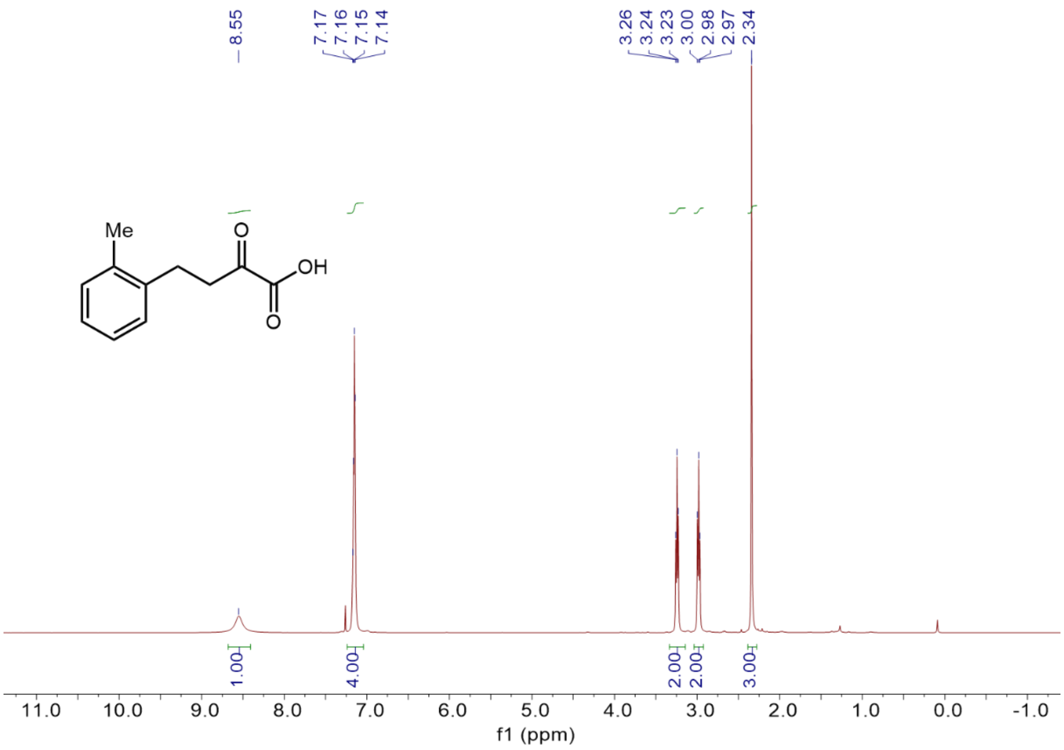

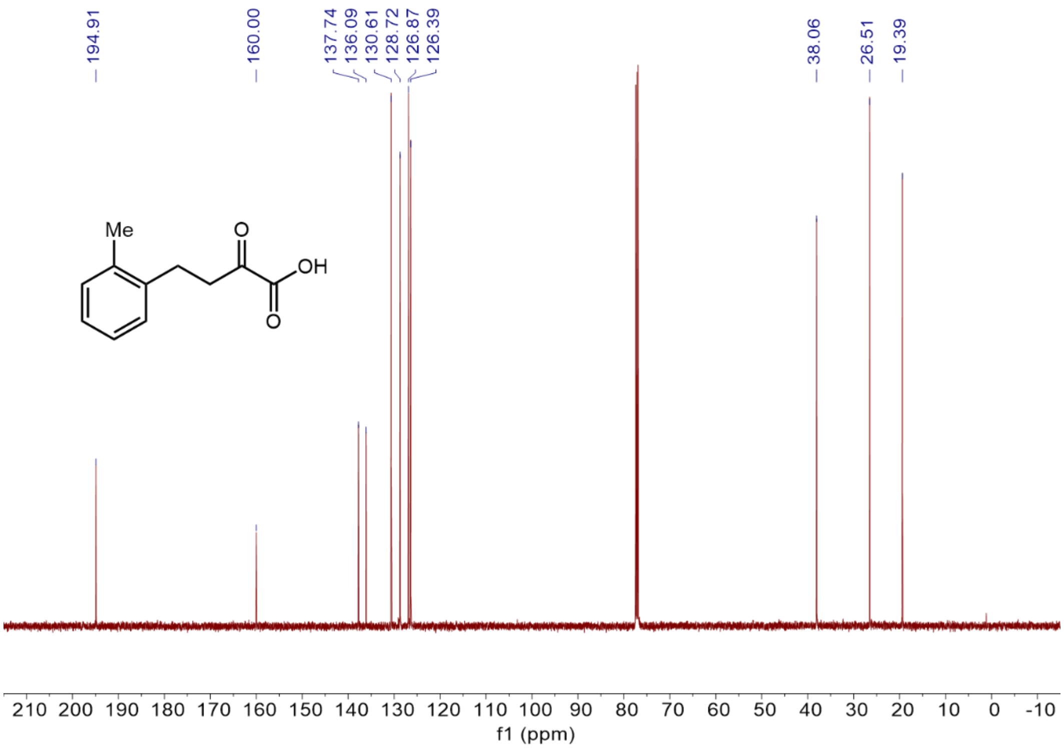

Supplementary Figure 12. NMR spectra of 2-oxo-4-(*o*-tolyl)butanoic acid (1b)

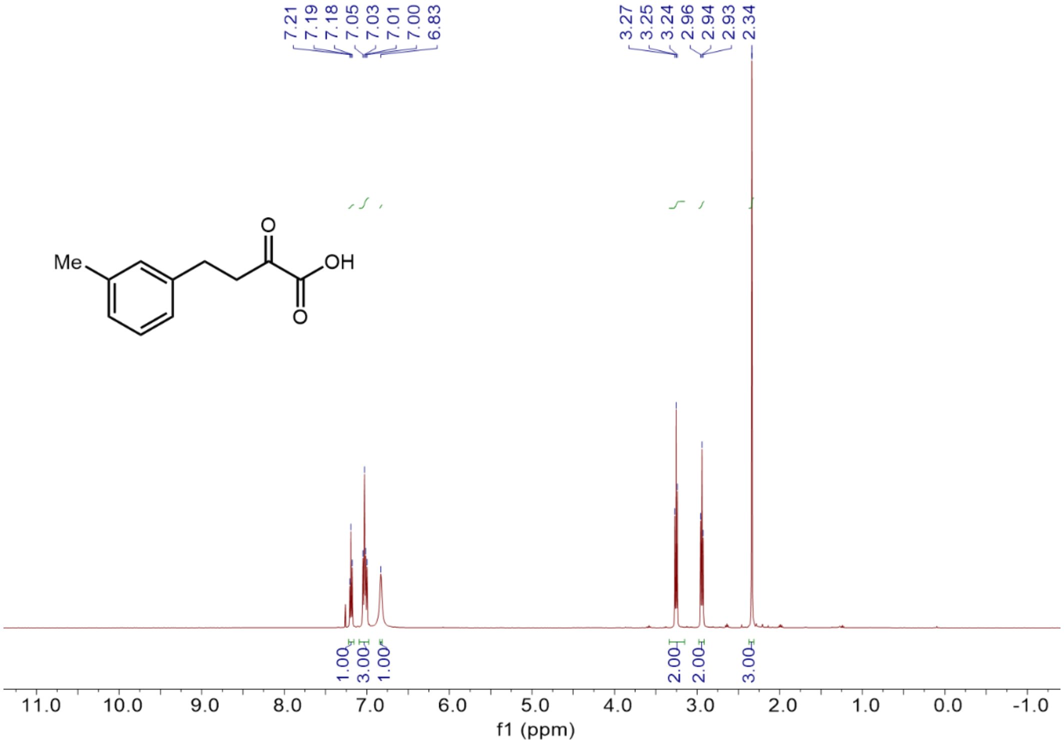

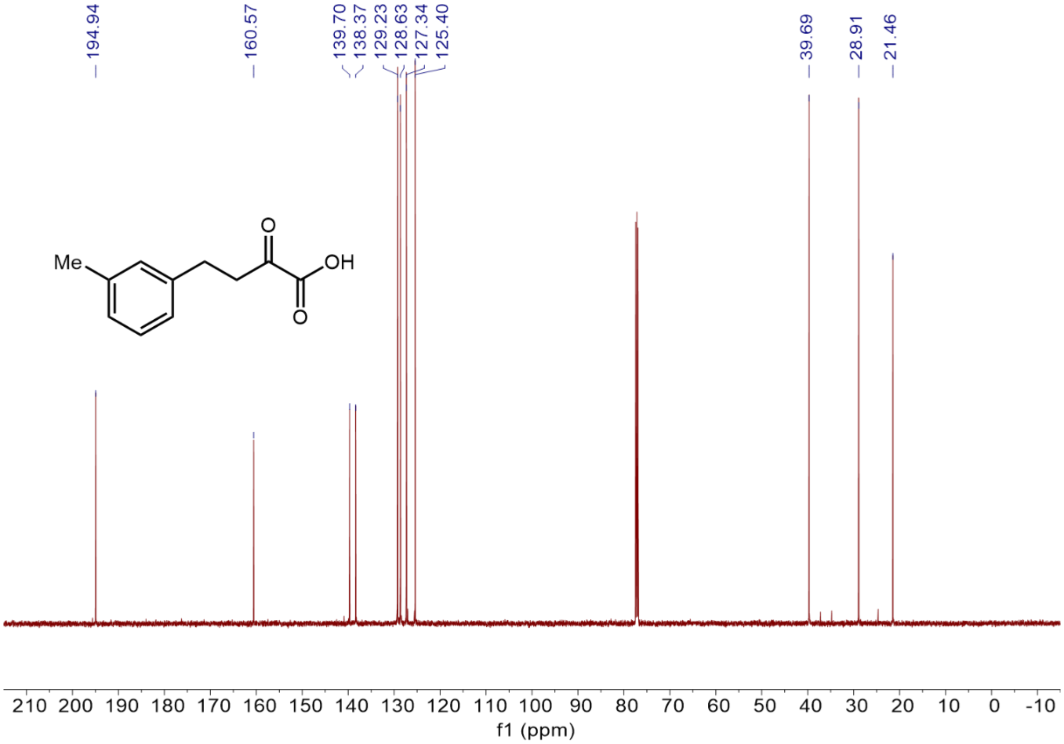

Supplementary Figure 13. NMR spectra of 2-oxo-4-(*m*-tolyl)butanoic acid (1c)

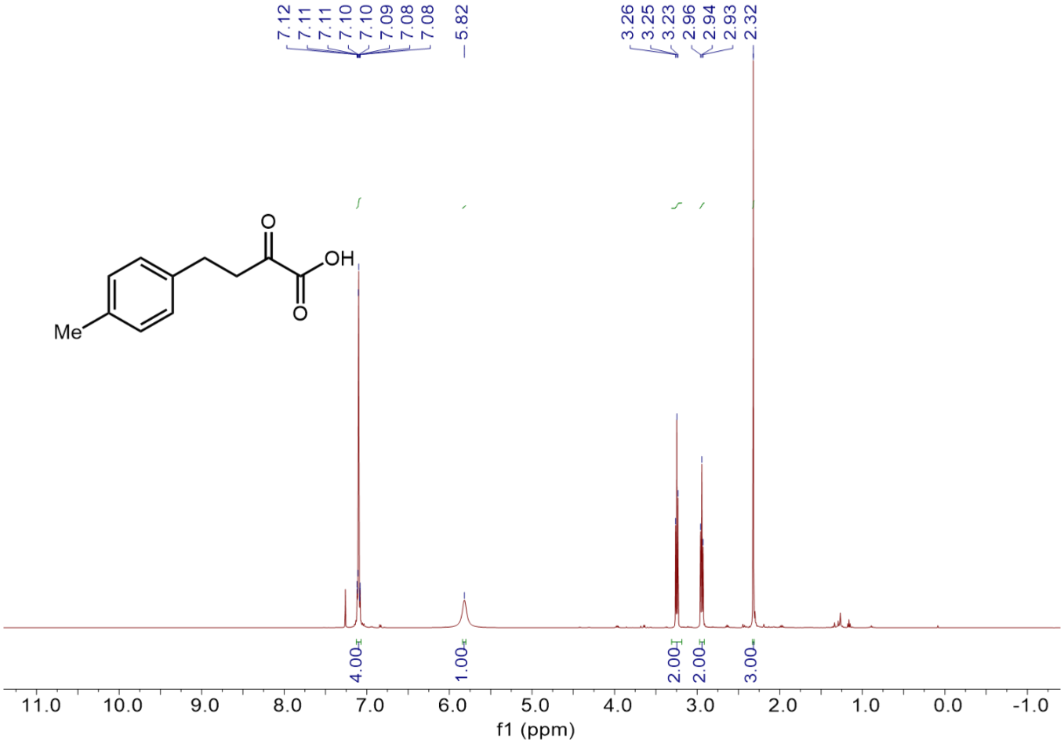

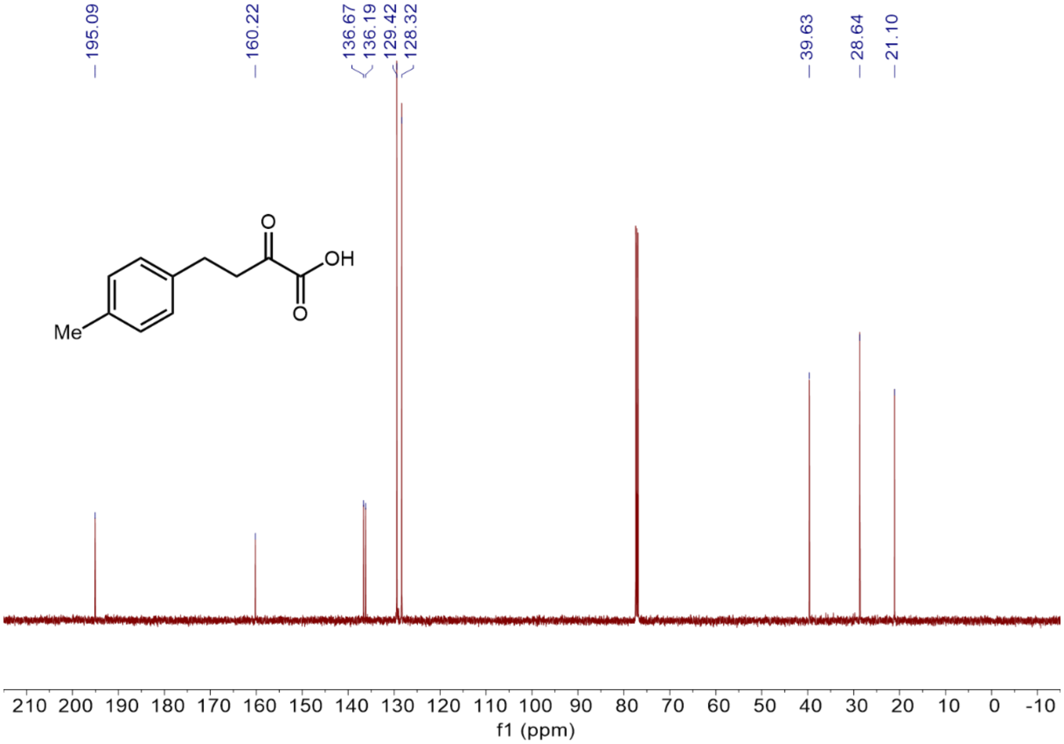

Supplementary Figure 14. NMR spectra of 2-oxo-4-(*p*-tolyl)butanoic acid (1d)

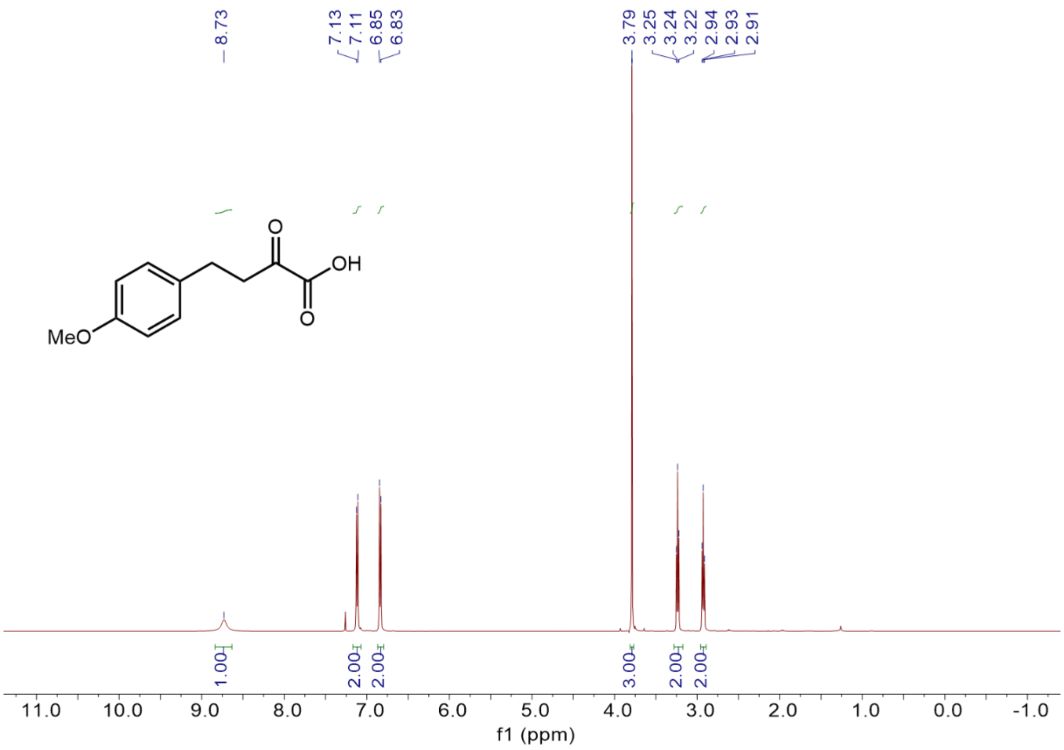

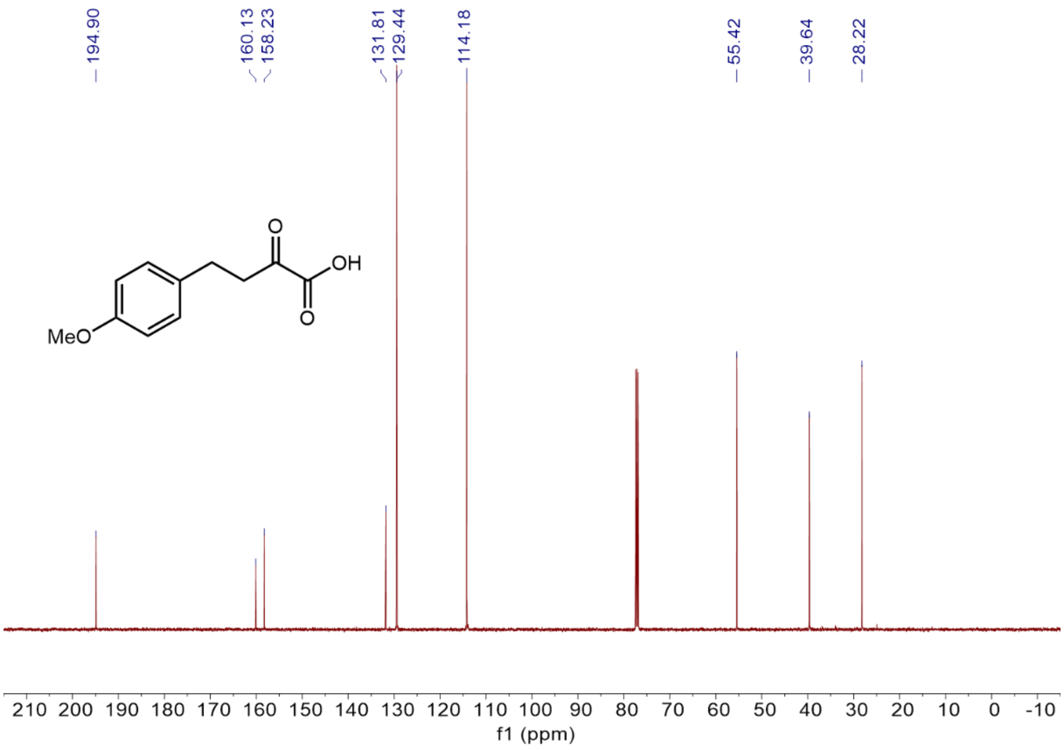

Supplementary Figure 15. NMR spectra of 4-(4-methoxyphenyl)-2-oxobutanoic acid (1e)

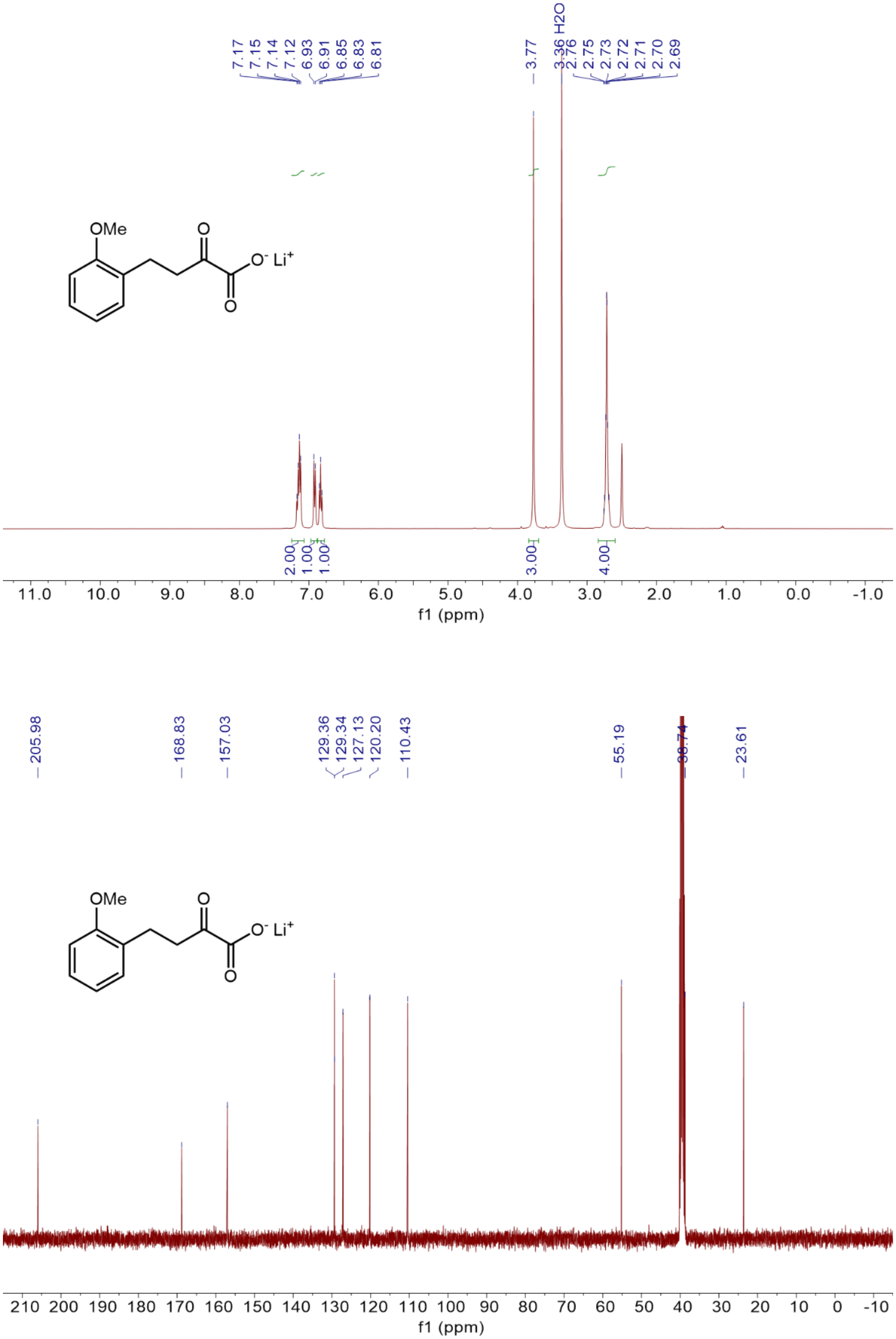

Supplementary Figure 16. NMR spectra of lithium 4-(2-methoxyphenyl)-2-oxobutanoate (1f)

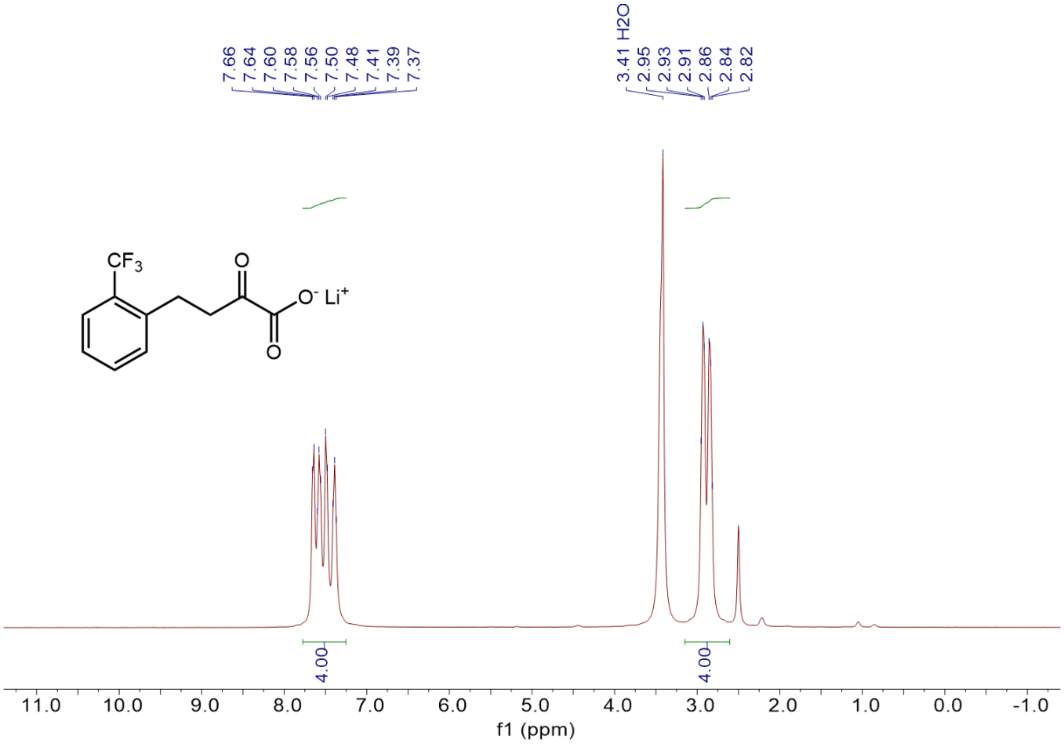
7.66
7.64
7.60
7.58
7.56
7.50
7.48
7.41
7.39
7.37
3.41 H2O
2.95
2.93
2.91
2.86
2.84
2.82
CF3
O
O- Li+
O
4.00
4.00
11.0 10.0 9.0 8.0 7.0 6.0 5.0 4.0 3.0 2.0 1.0 0.0 -1.0
f1 (ppm)

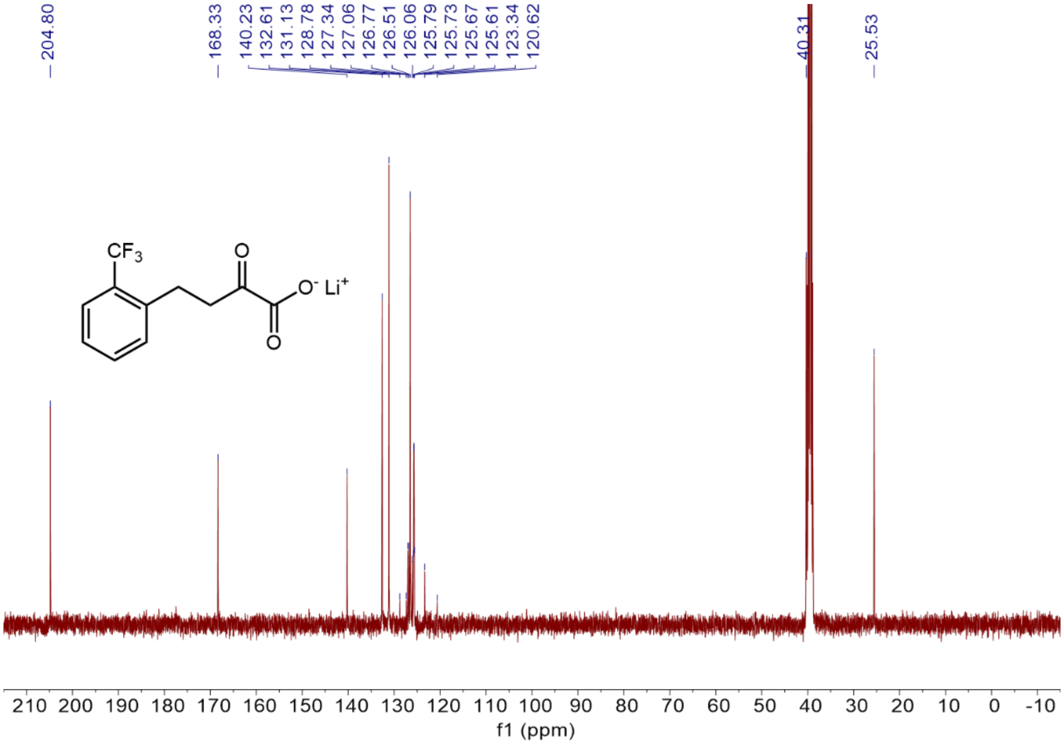
204.80
168.33
140.23
132.61
131.13
128.78
127.34
127.06
126.77
126.51
126.06
125.79
125.73
125.67
125.61
123.34
120.62
40.31
25.53
CF3
O
O- Li+
O
210 200 190 180 170 160 150 140 130 120 110 100 90 80 70 60 50 40 30 20 10 0 -10
f1 (ppm)

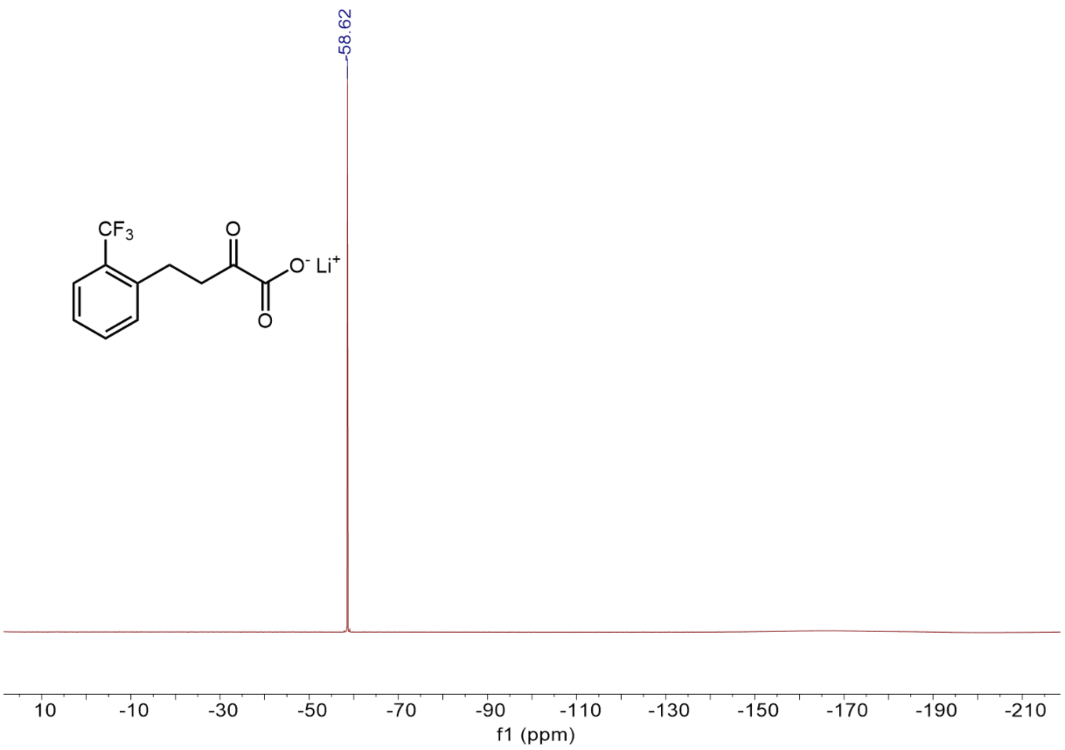

Supplementary Figure 17. NMR spectra of lithium 2-oxo-4-(2-(trifluoromethyl)phenyl)butanoate (1g)

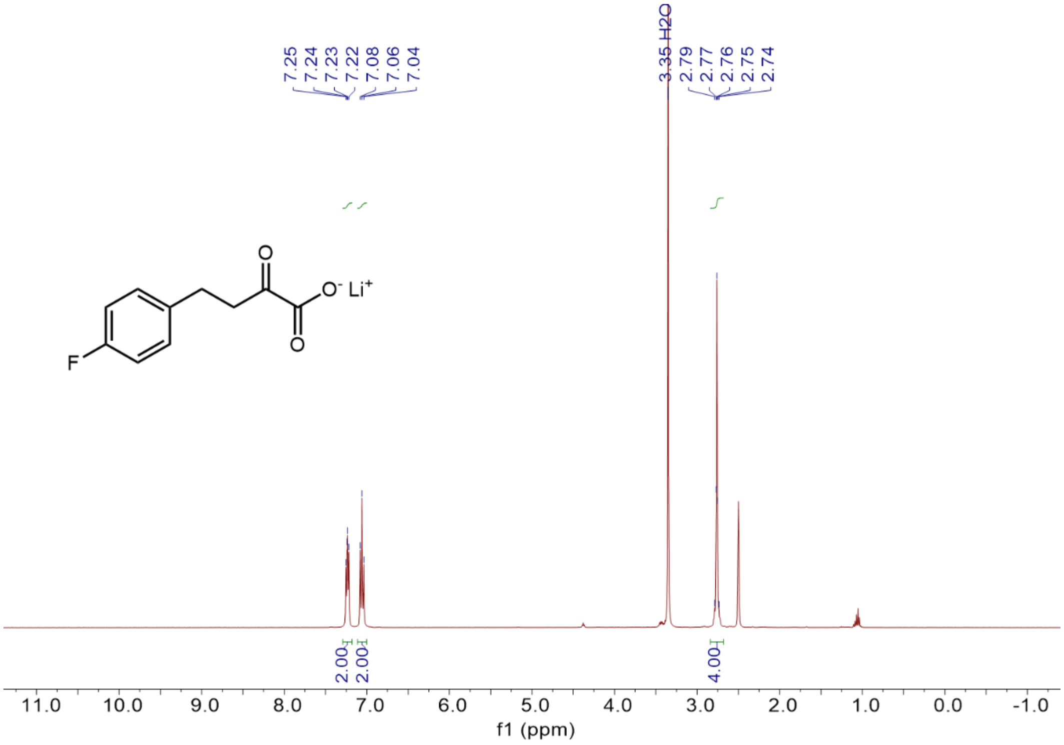
7.25
7.24
7.23
7.22
7.08
7.06
7.04
3.35 H2O
2.79
2.77
2.76
2.75
2.74
O
O- Li+
O
F
2.00
2.00
4.00
11.0 10.0 9.0 8.0 7.0 6.0 5.0 4.0 3.0 2.0 1.0 0.0 -1.0
f1 (ppm)

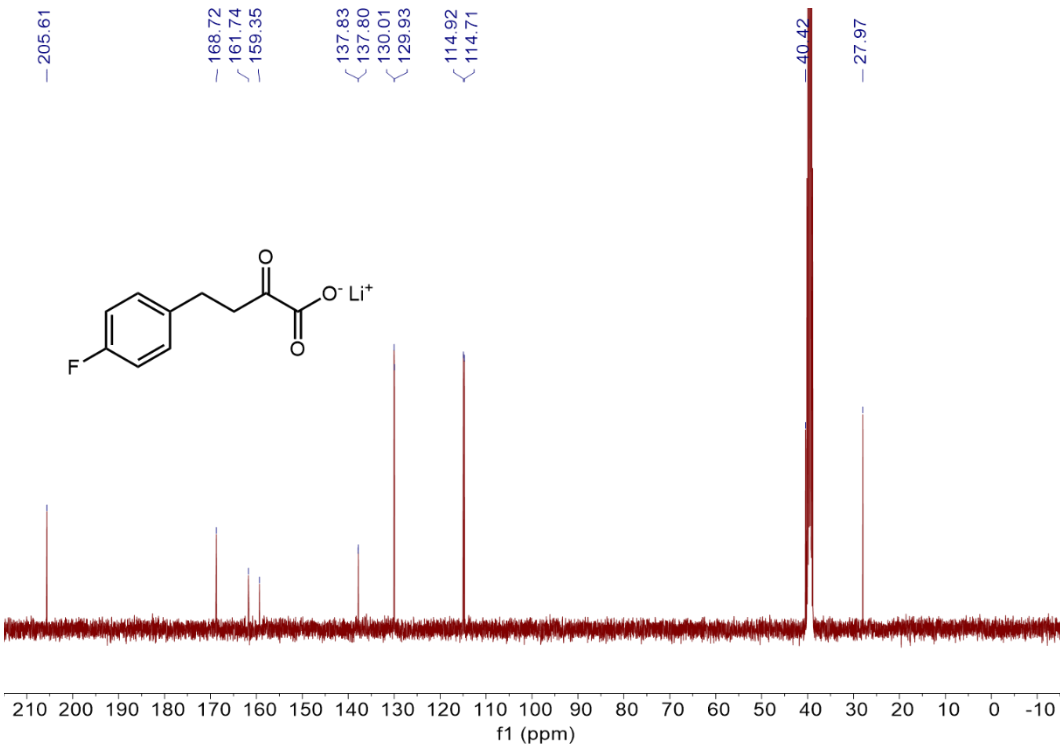
205.61
168.72
161.74
159.35
137.83
137.80
130.01
129.93
114.92
114.71
40.42
27.97
O
O- Li+
O
F
210 200 190 180 170 160 150 140 130 120 110 100 90 80 70 60 50 40 30 20 10 0 -10
f1 (ppm)

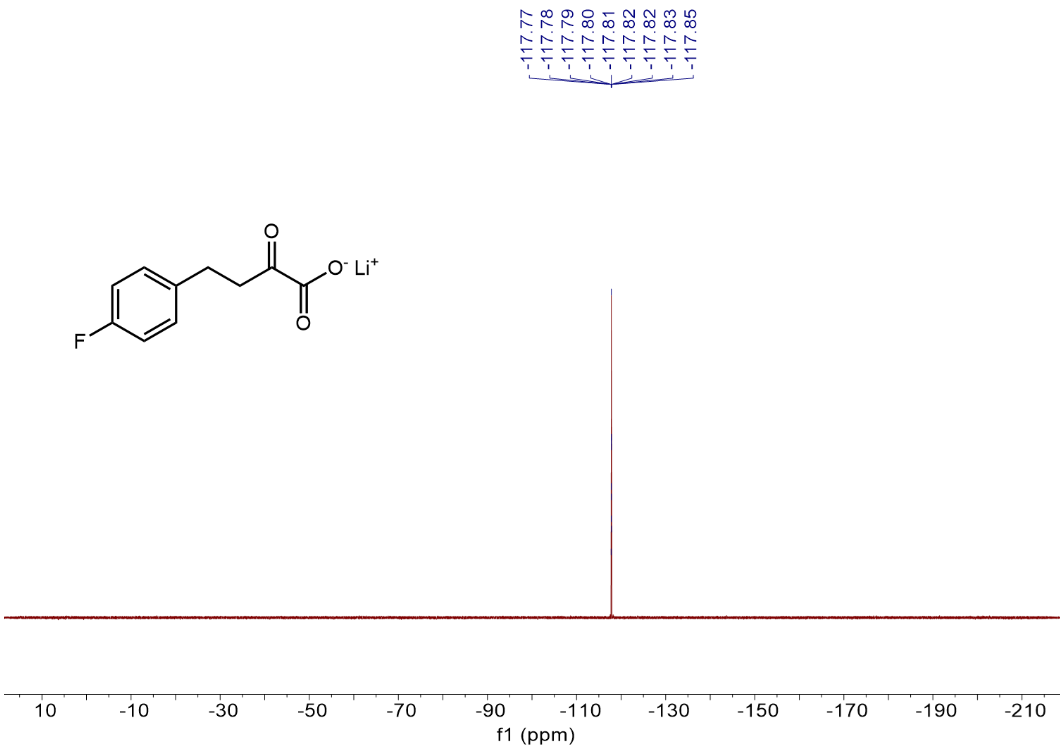

Supplementary Figure 18. NMR spectra of lithium 4-(4-fluorophenyl)-2-oxobutanoate (1h)

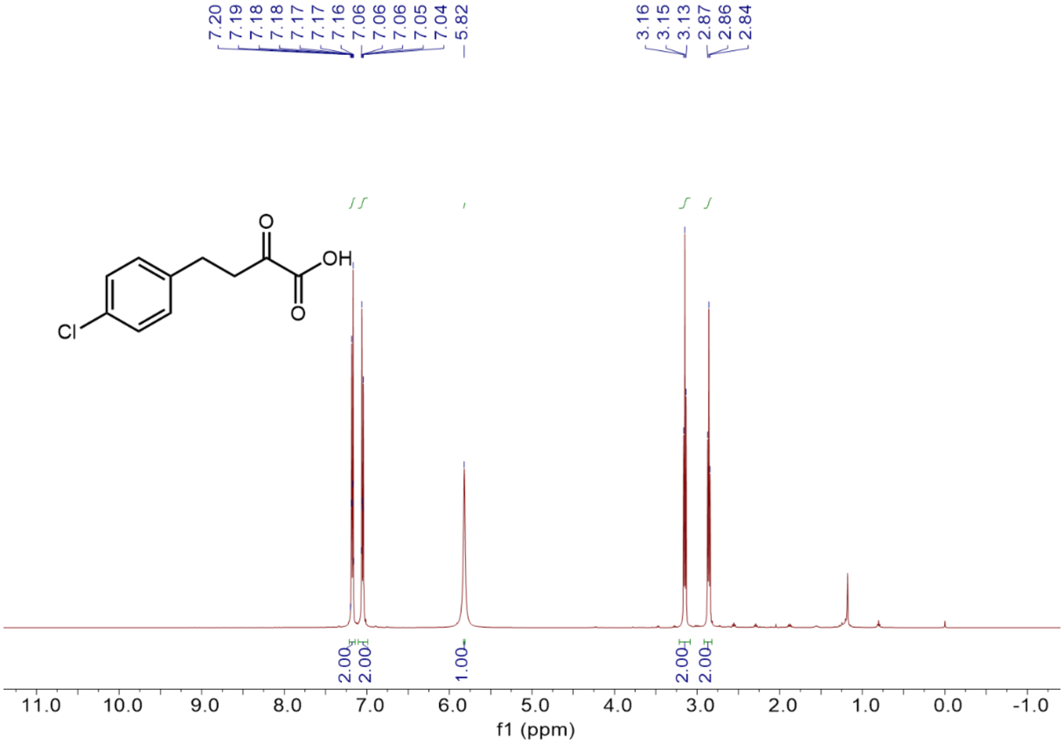

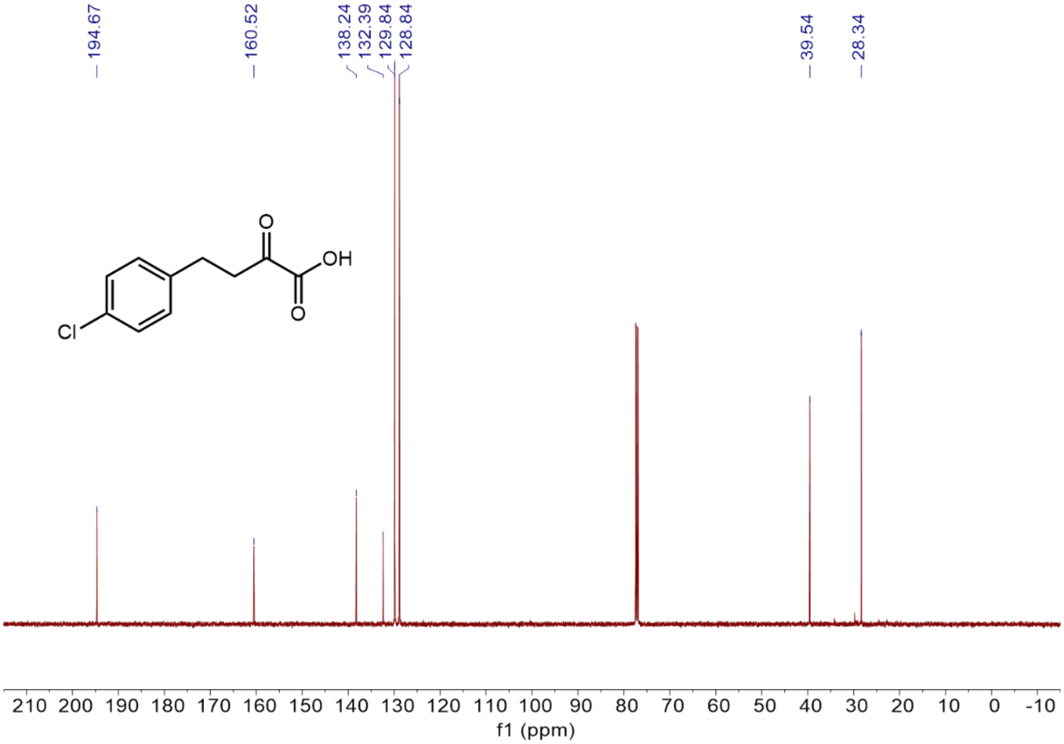

Supplementary Figure 19. NMR spectra of 4-(4-chlorophenyl)-2-oxobutanoic acid (1i)

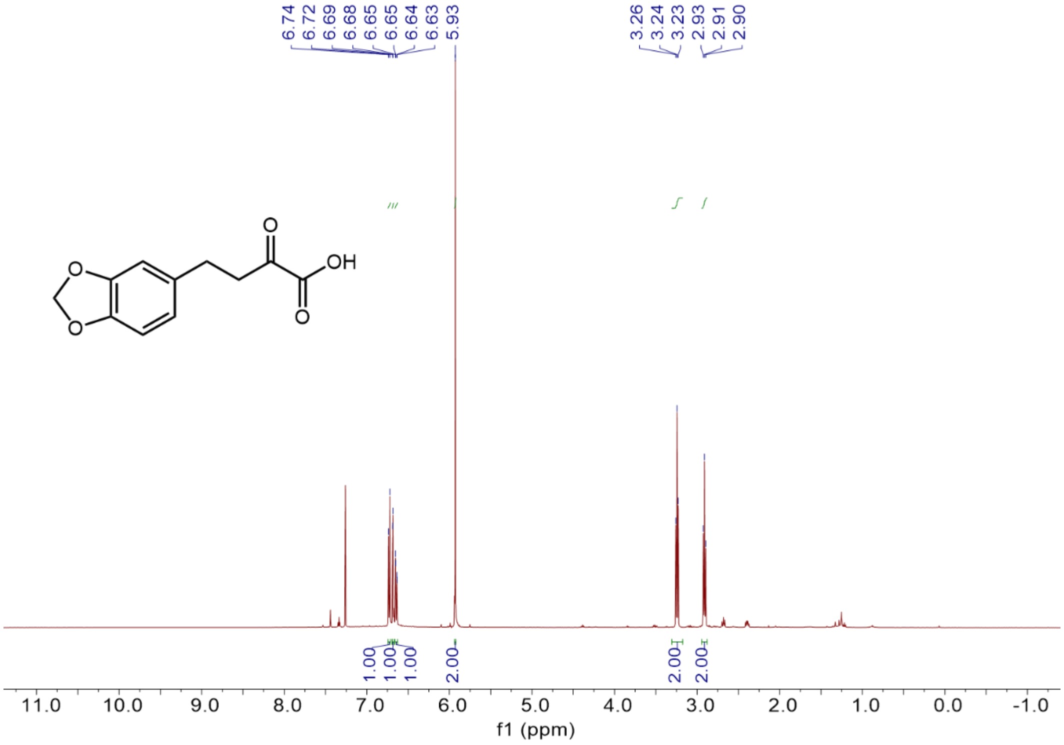

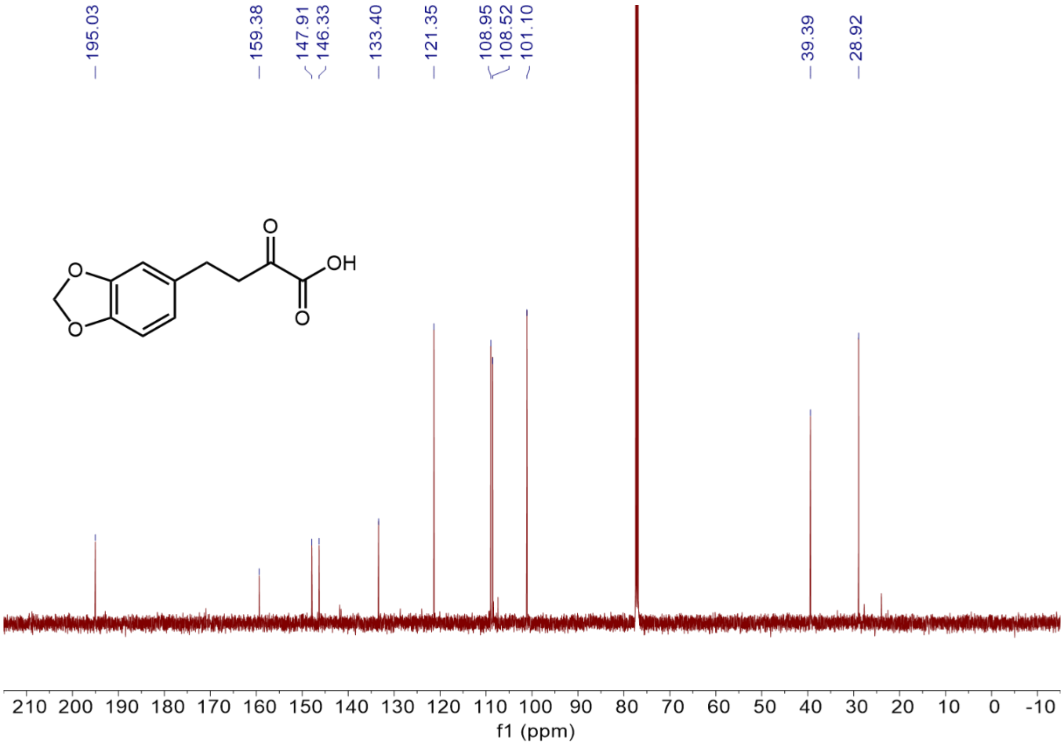

Supplementary Figure 20. NMR spectra of 4-(benzo[*d*][1,3]dioxol-5-yl)-2-oxobutanoic acid (1j)

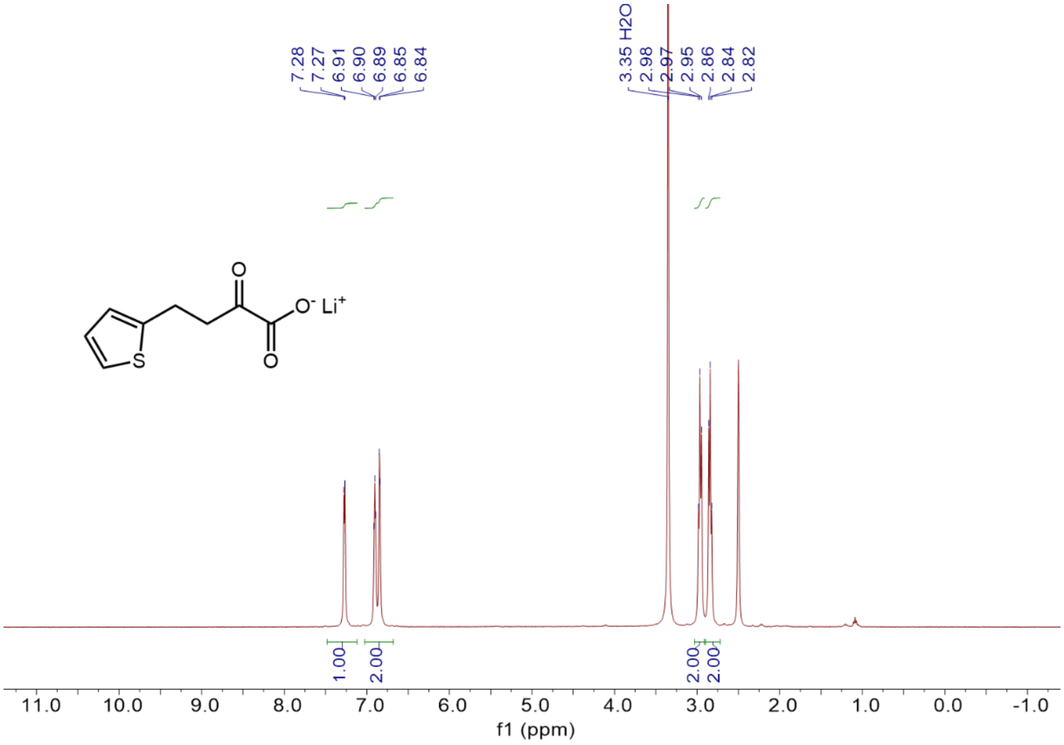

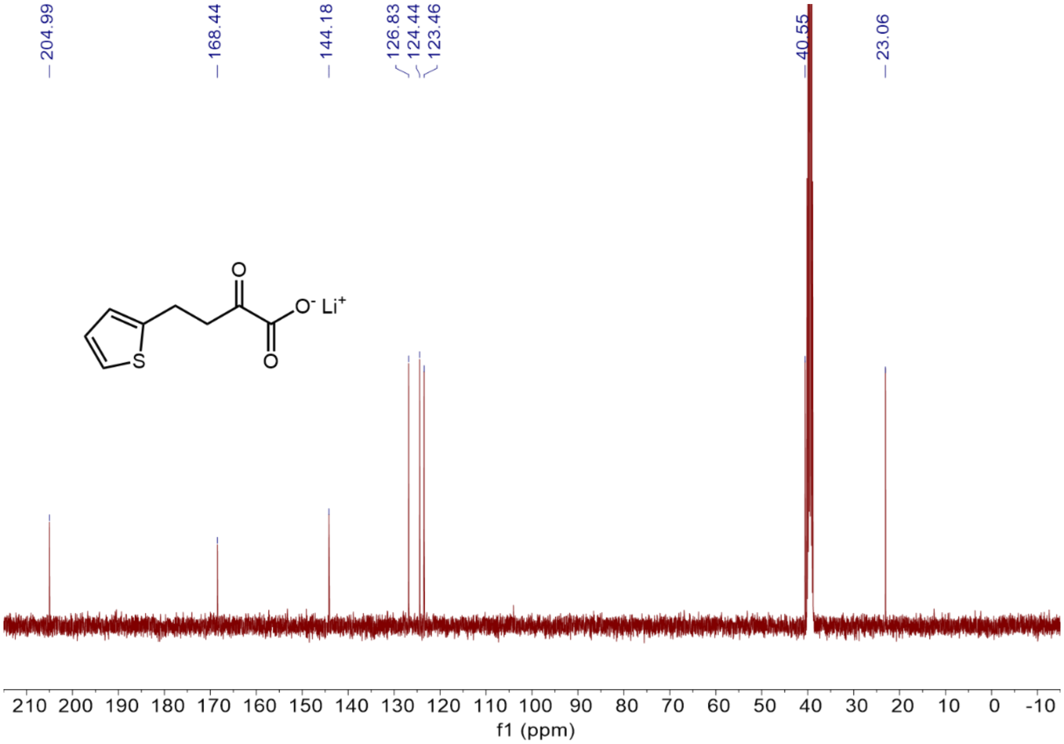

Supplementary Figure 21. NMR spectra of lithium 2-oxo-4-(thiophen-2-yl)butanoate (1k)

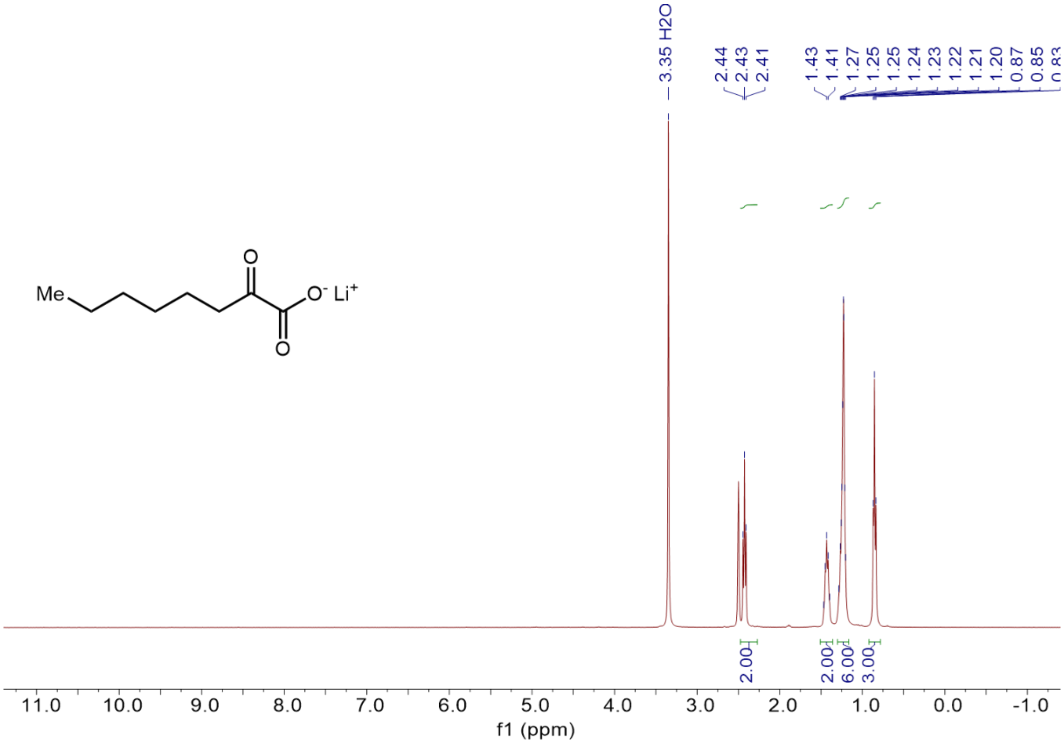

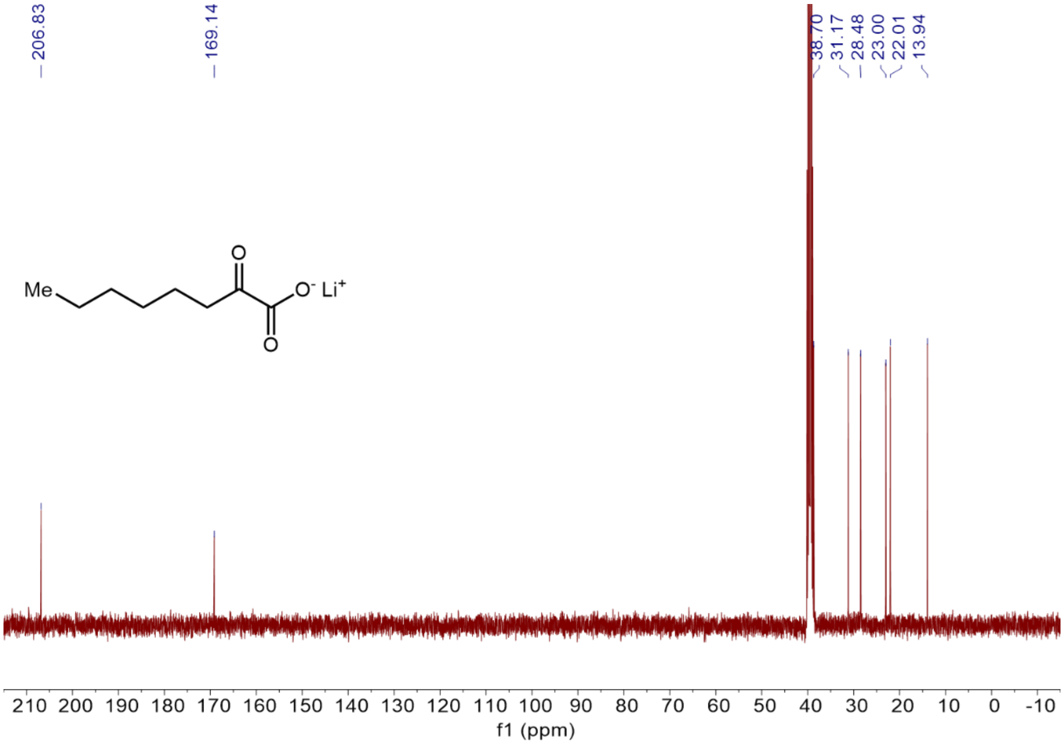

Supplementary Figure 22. NMR spectra of lithium 2-oxooctanoate (1l)

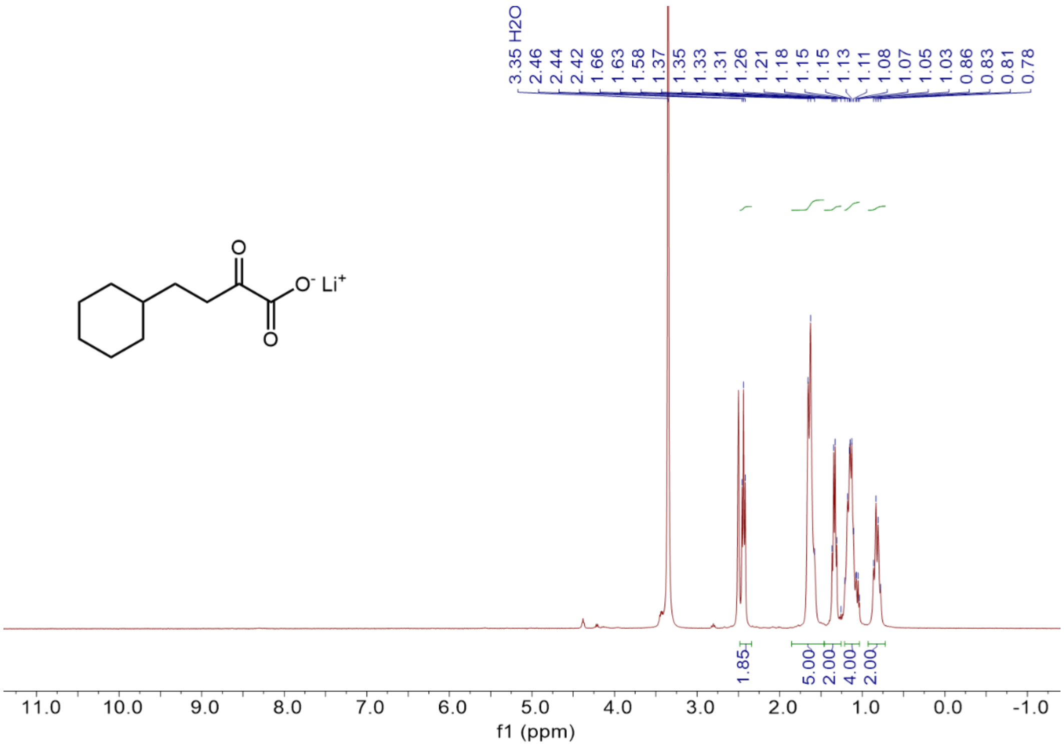

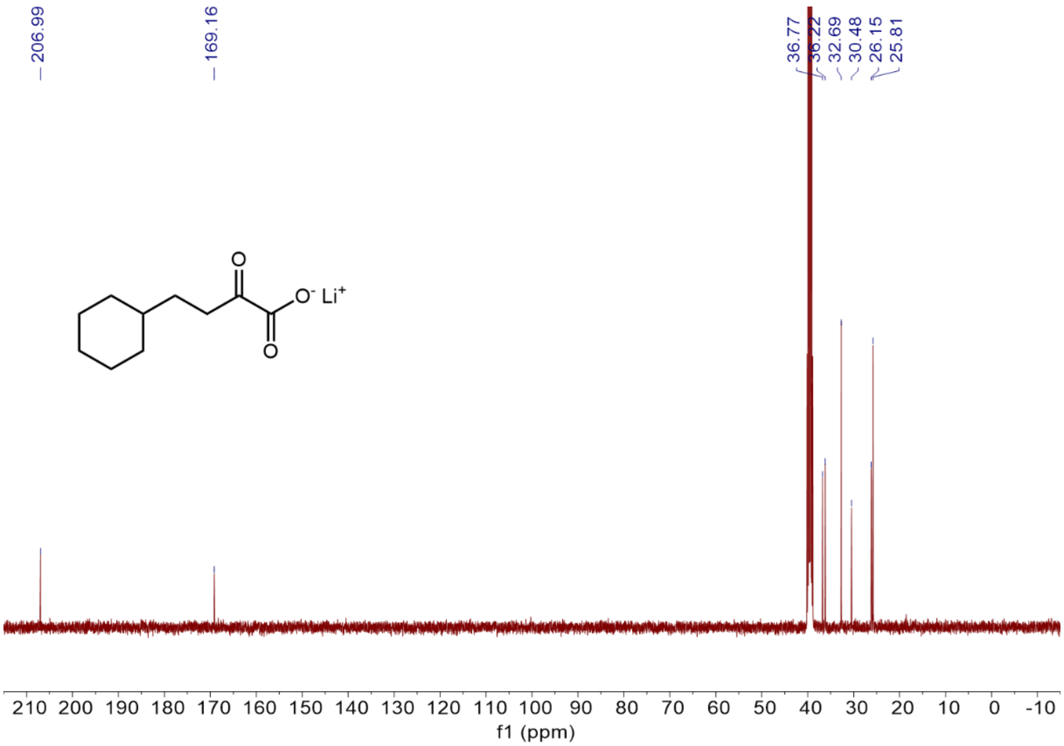

Supplementary Figure 23. NMR spectra of lithium 4-cyclohexyl-2-oxobutanoate (1m)

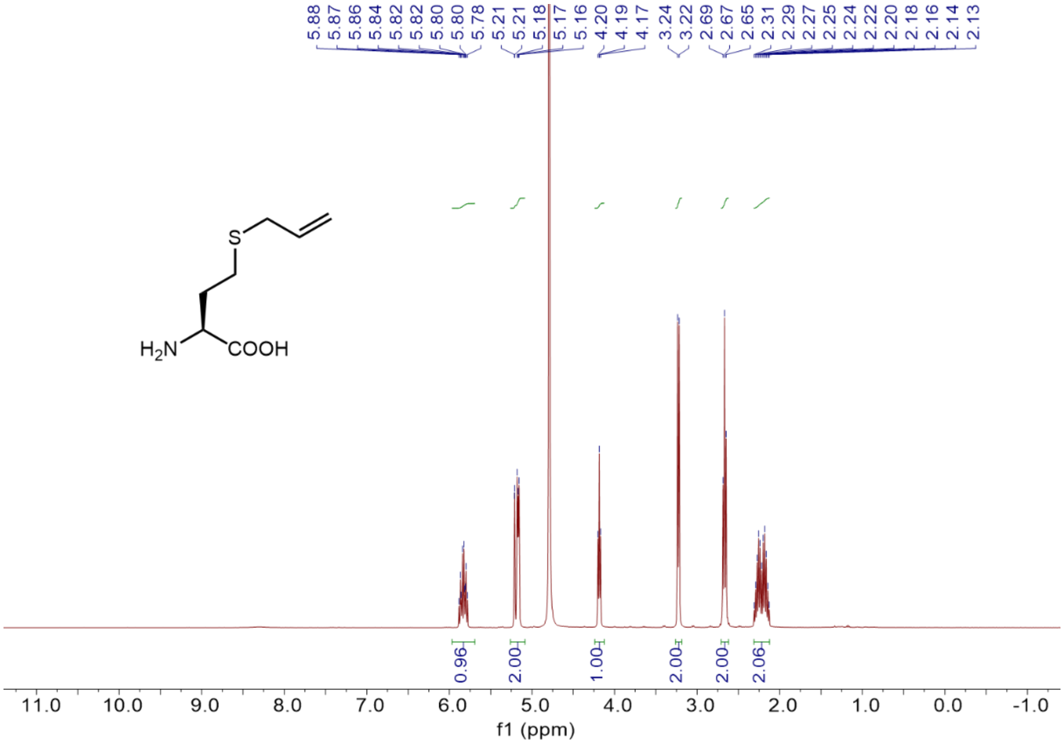

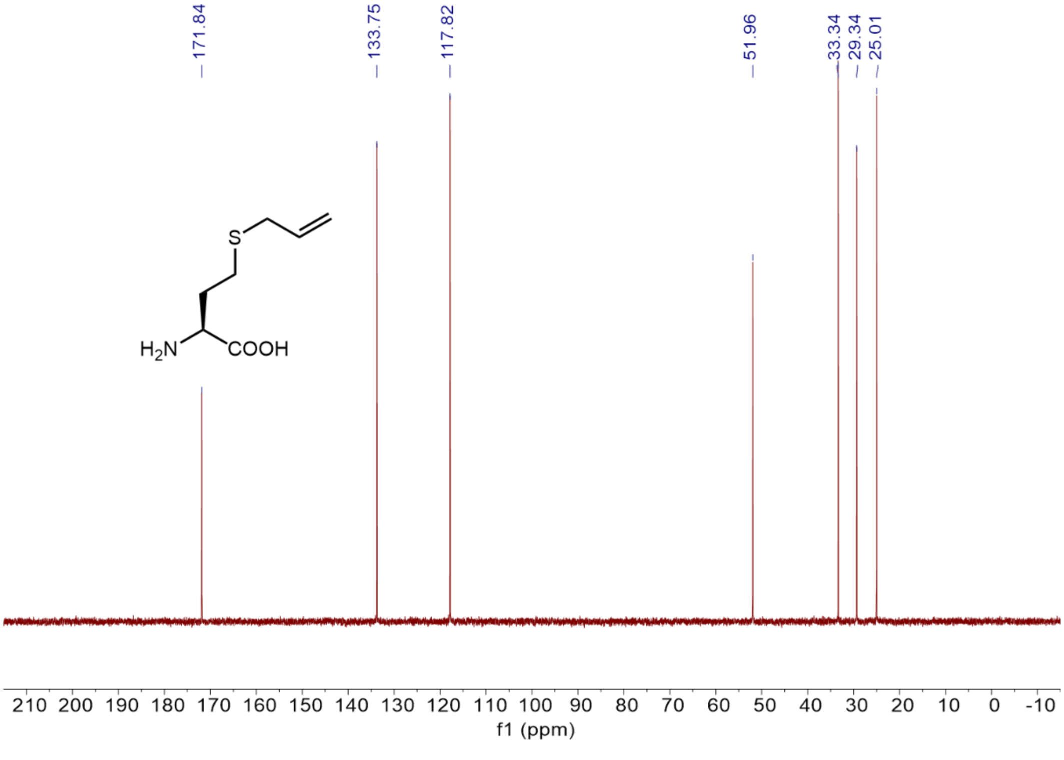

Supplementary Figure 24. NMR spectra of *S*-allyl-L-homocysteine (SI-1a)

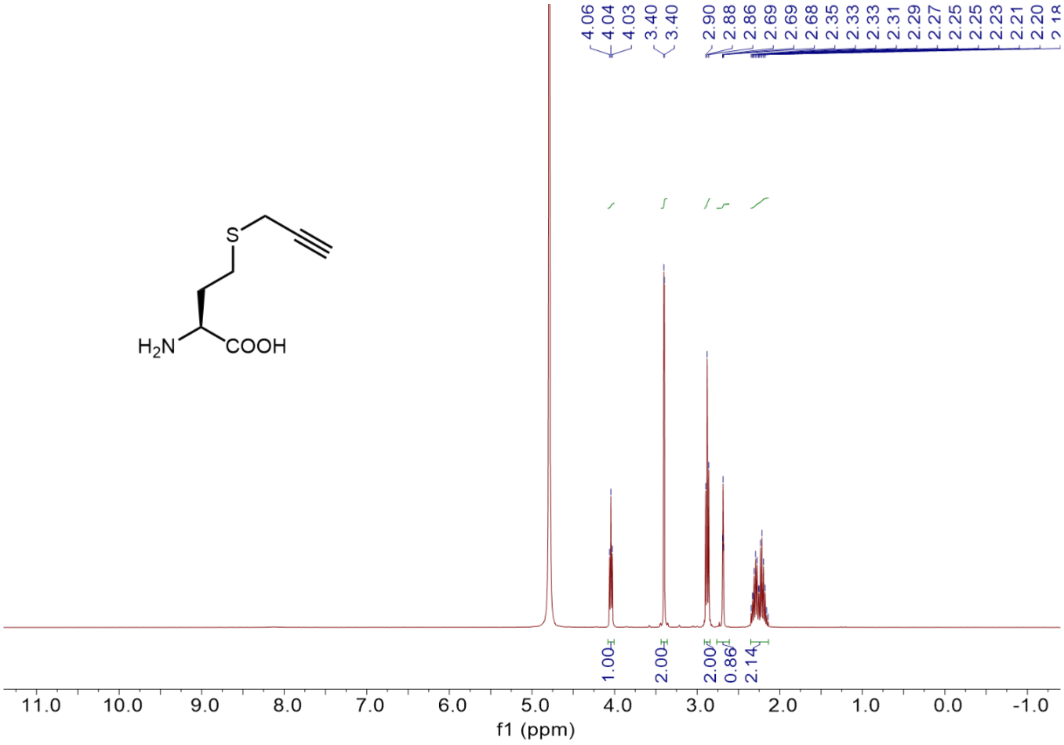

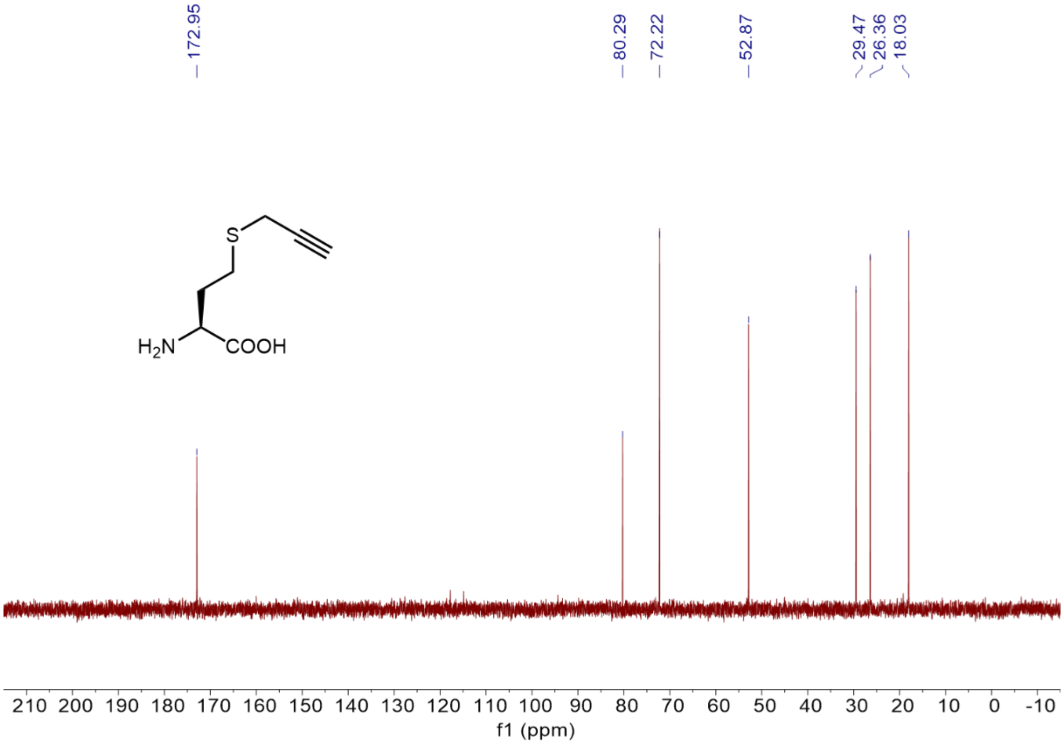

Supplementary Figure 25. NMR spectra of *S*-(prop-2-yn-1-yl)-L-homocysteine (SI-1b)

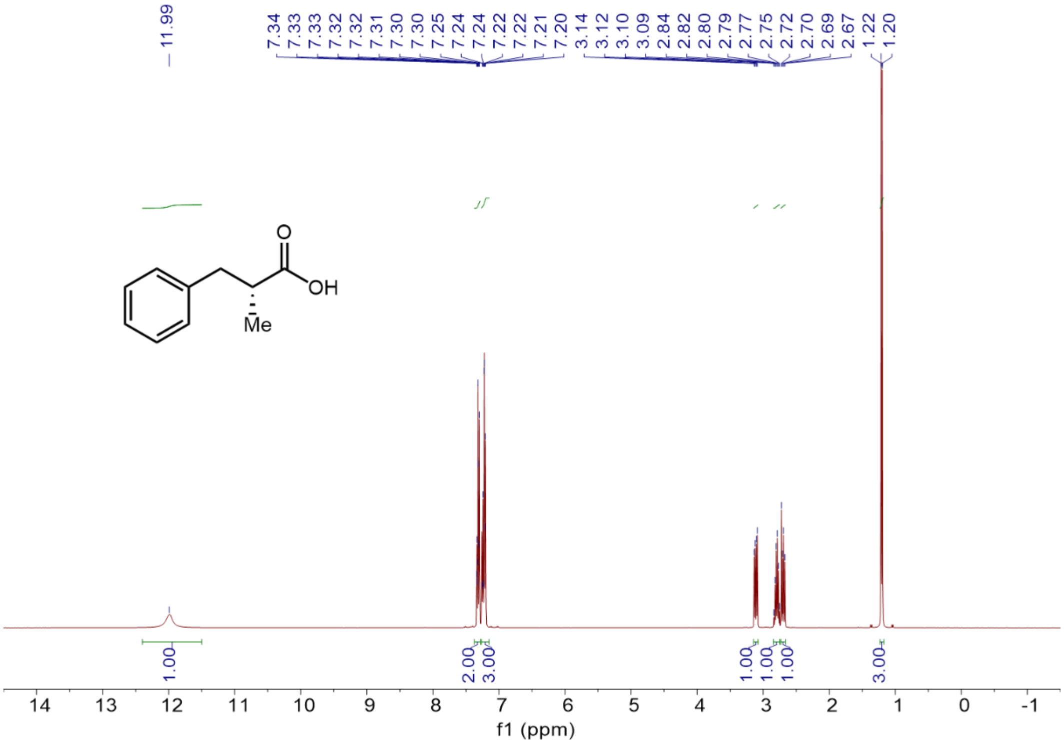

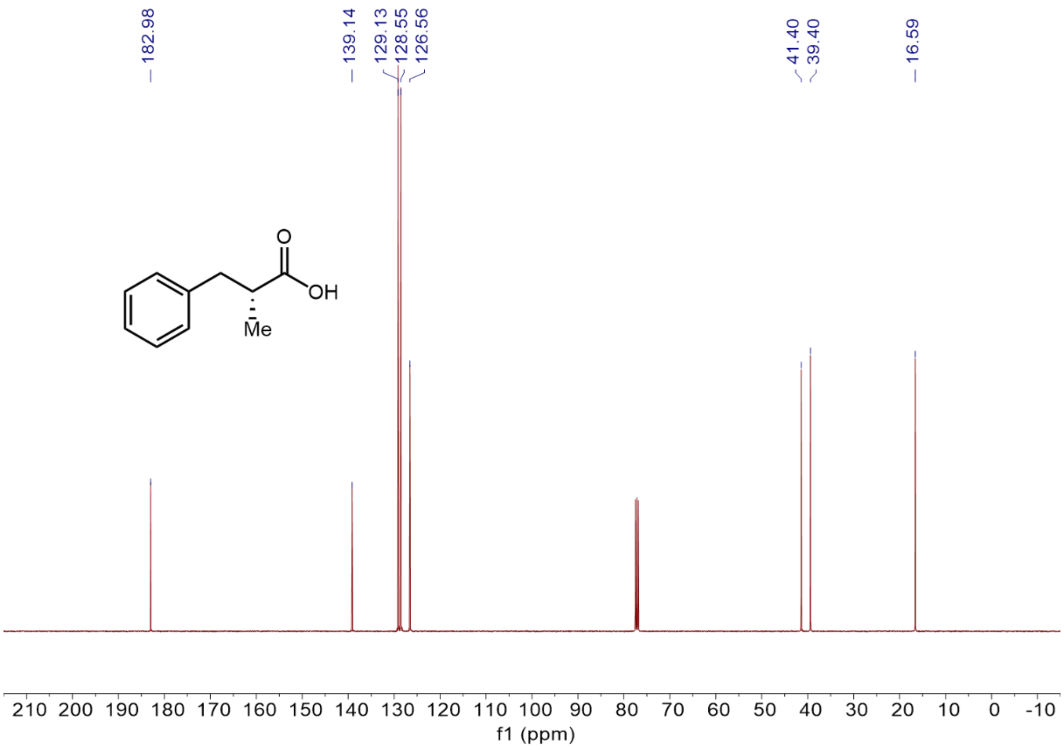

Supplementary Figure 26. NMR spectra of (*R*)-2-methyl-3-phenylpropanoic acid (3a)

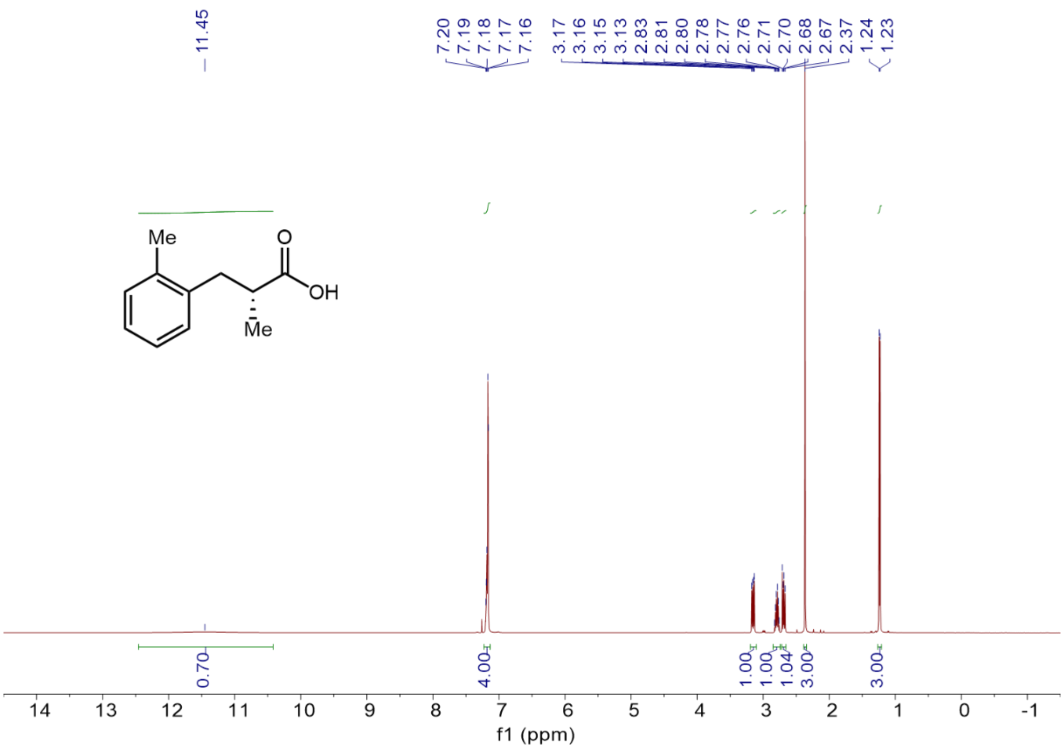

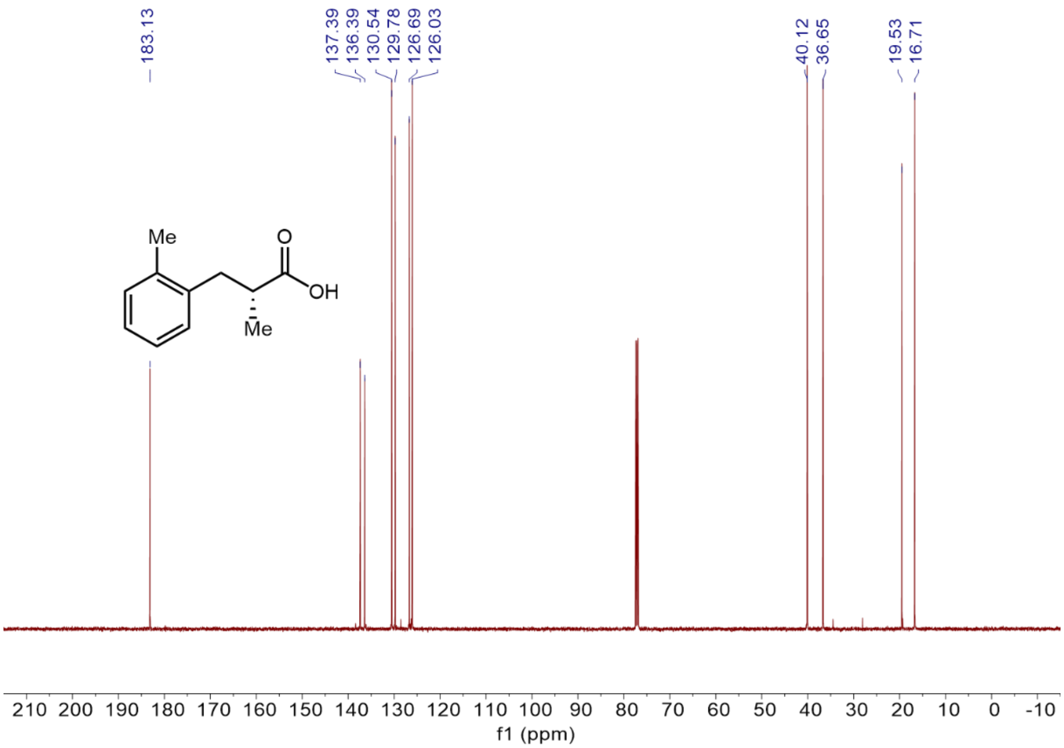

Supplementary Figure 27. NMR spectra of (*R*)-2-methyl-3-(*o*-tolyl)propanoic acid (3b)

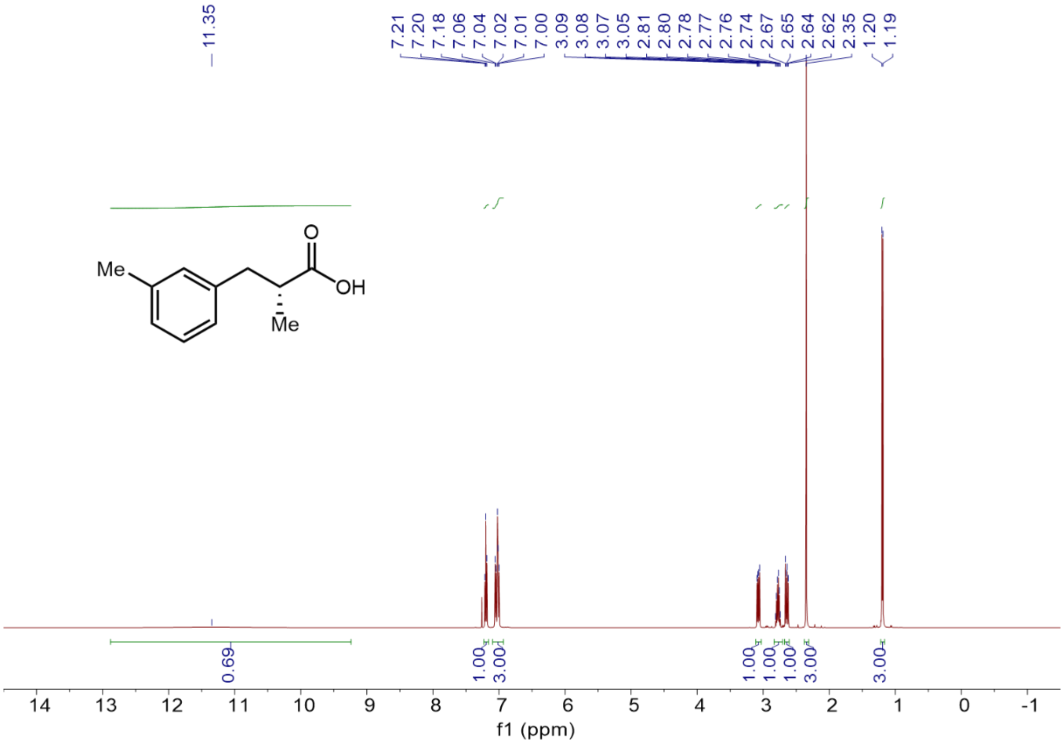

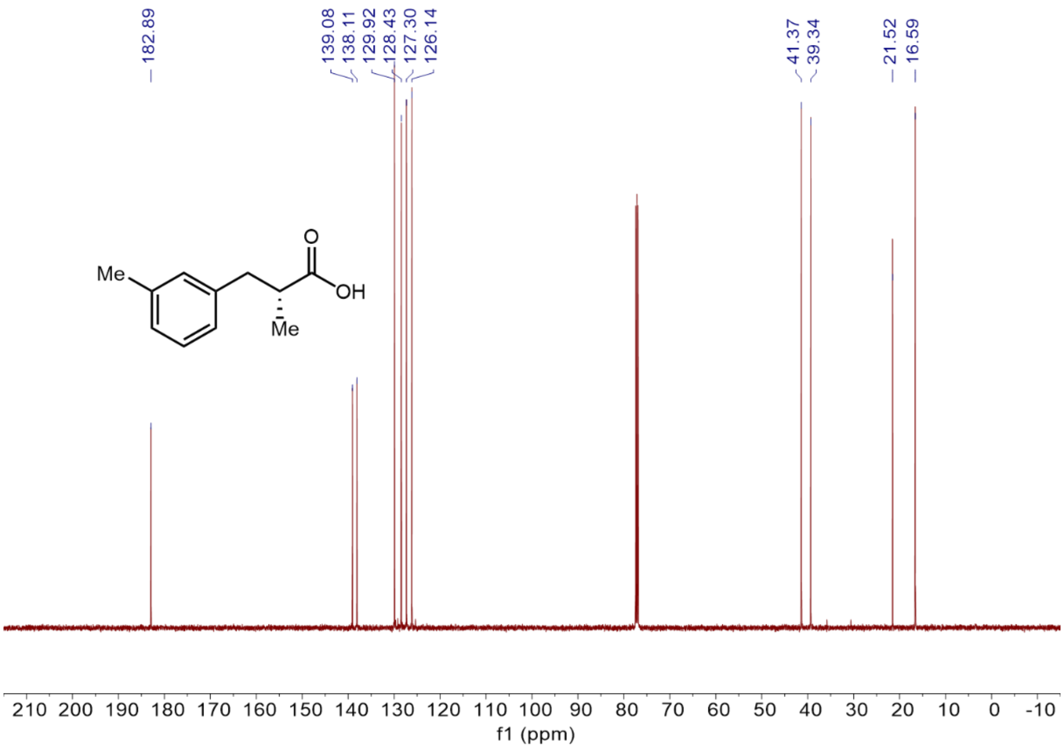

Supplementary Figure 28. NMR spectra of (*R*)-2-methyl-3-(*m*-tolyl)propanoic acid (3c)

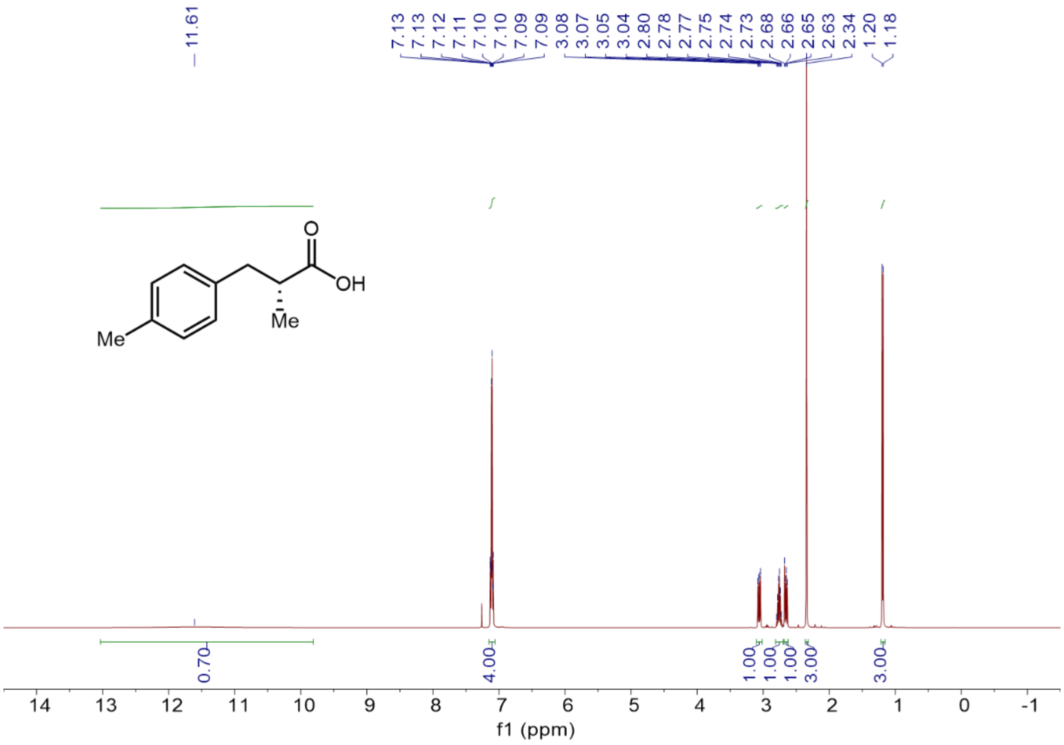

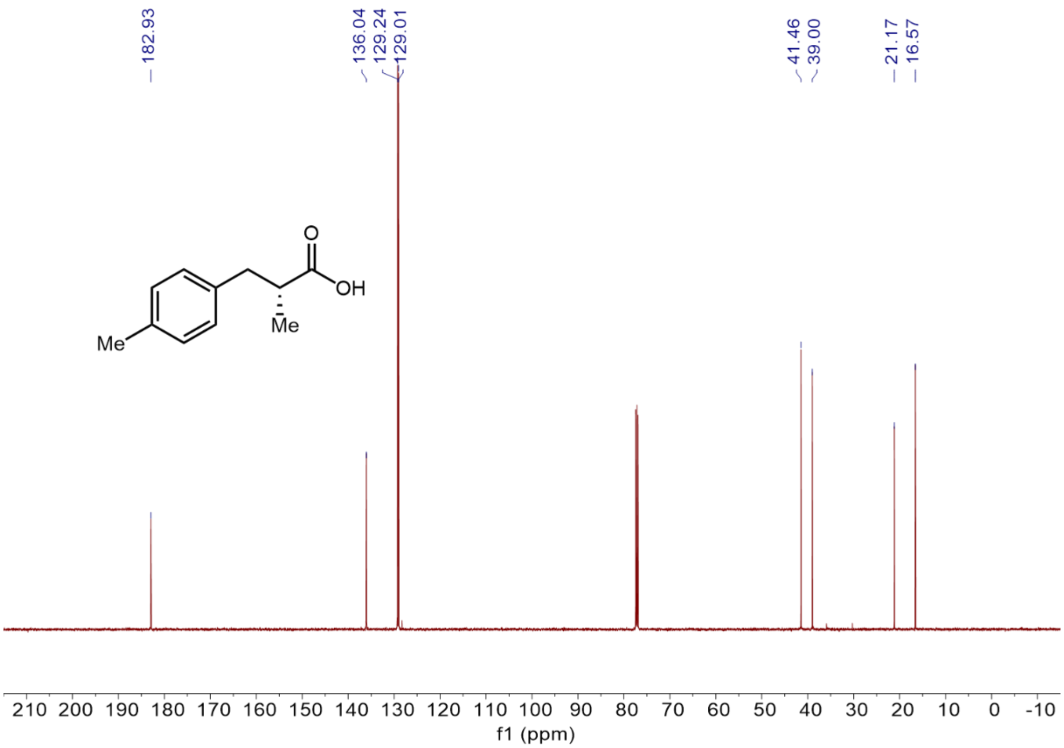

Supplementary Figure 29. NMR spectra of (*R*)-2-methyl-3-(*p*-tolyl)propanoic acid (3d)

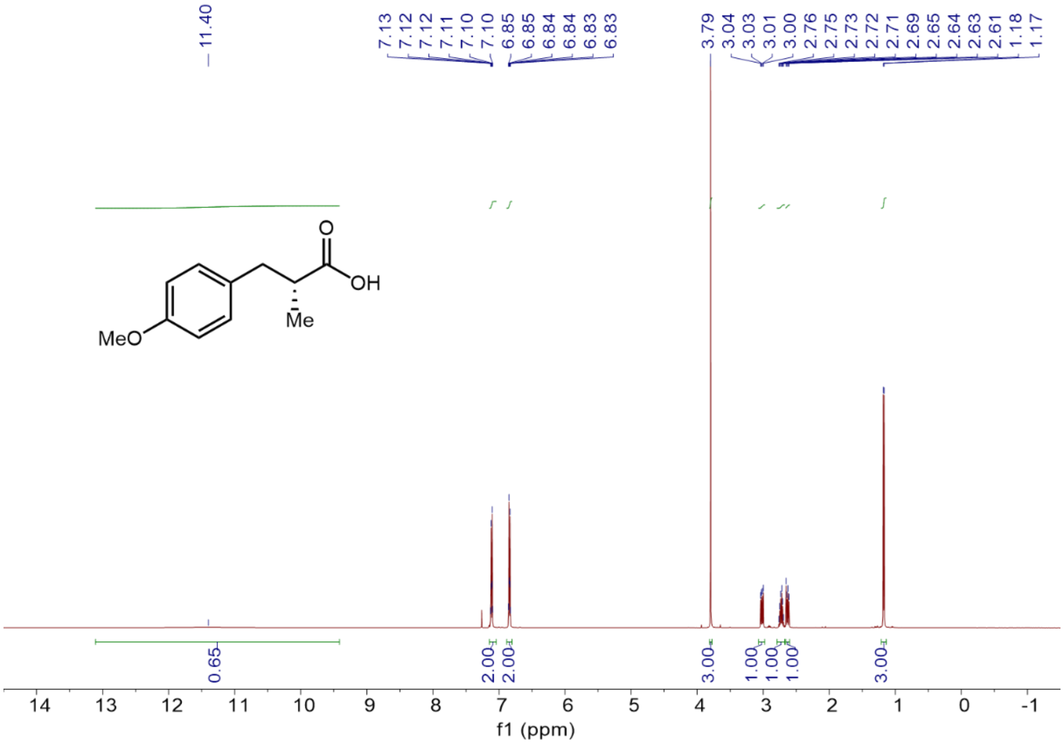

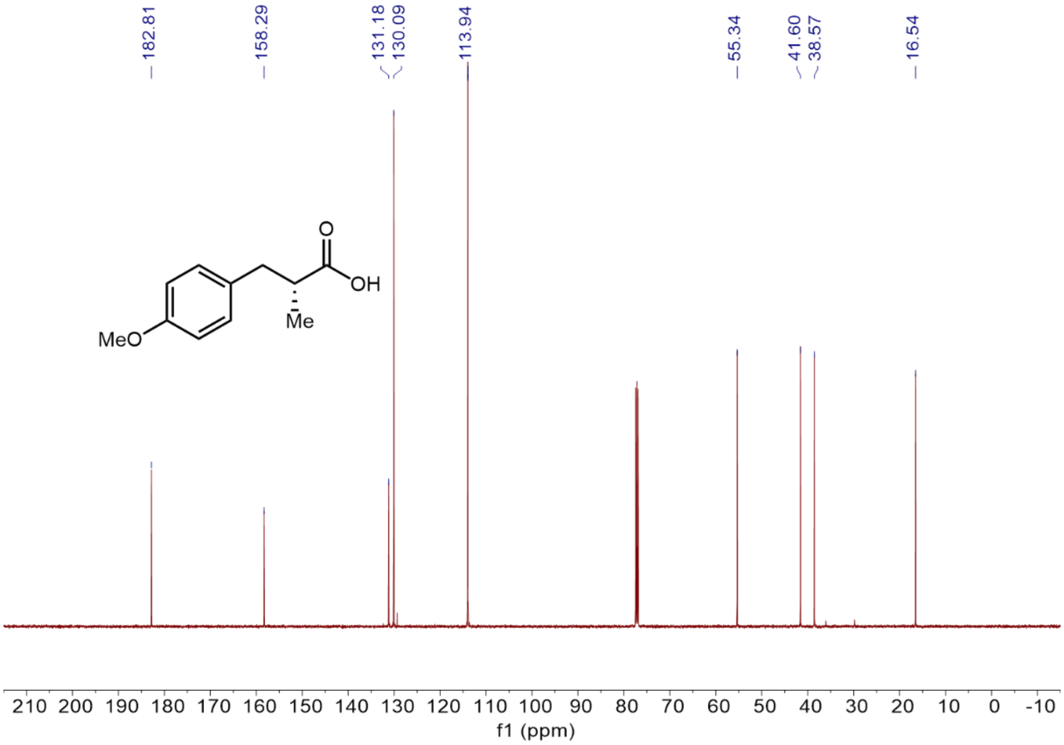

Supplementary Figure 30. NMR spectra of (*R*)-3-(4-methoxyphenyl)-2-methylpropanoic acid (3e)

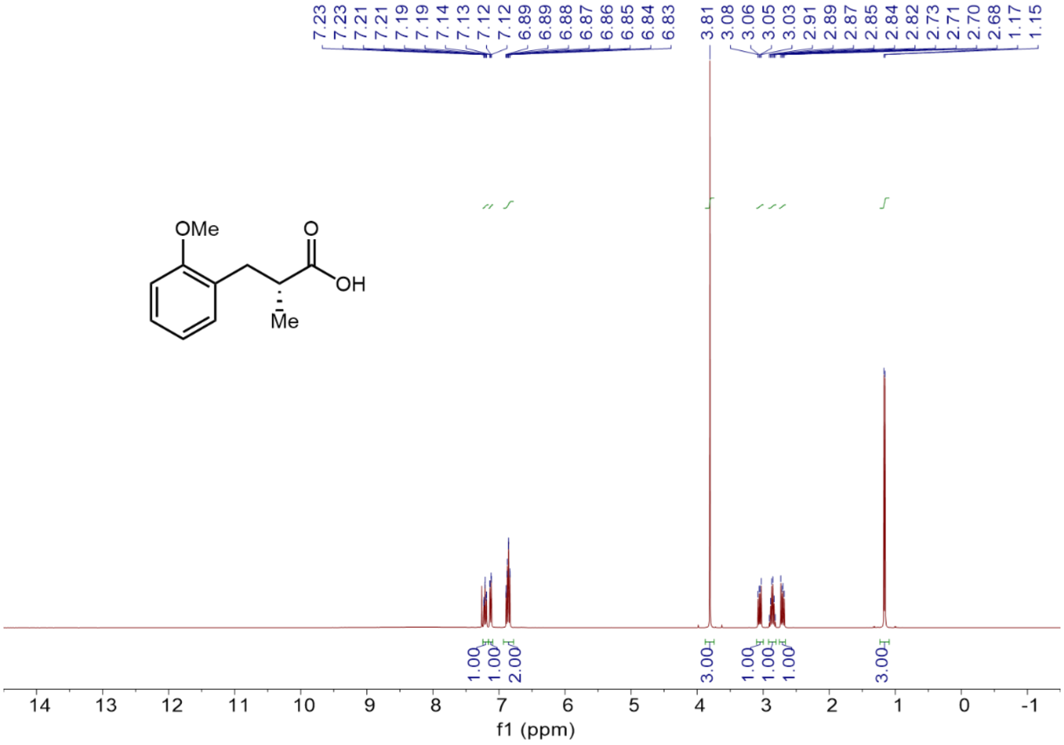

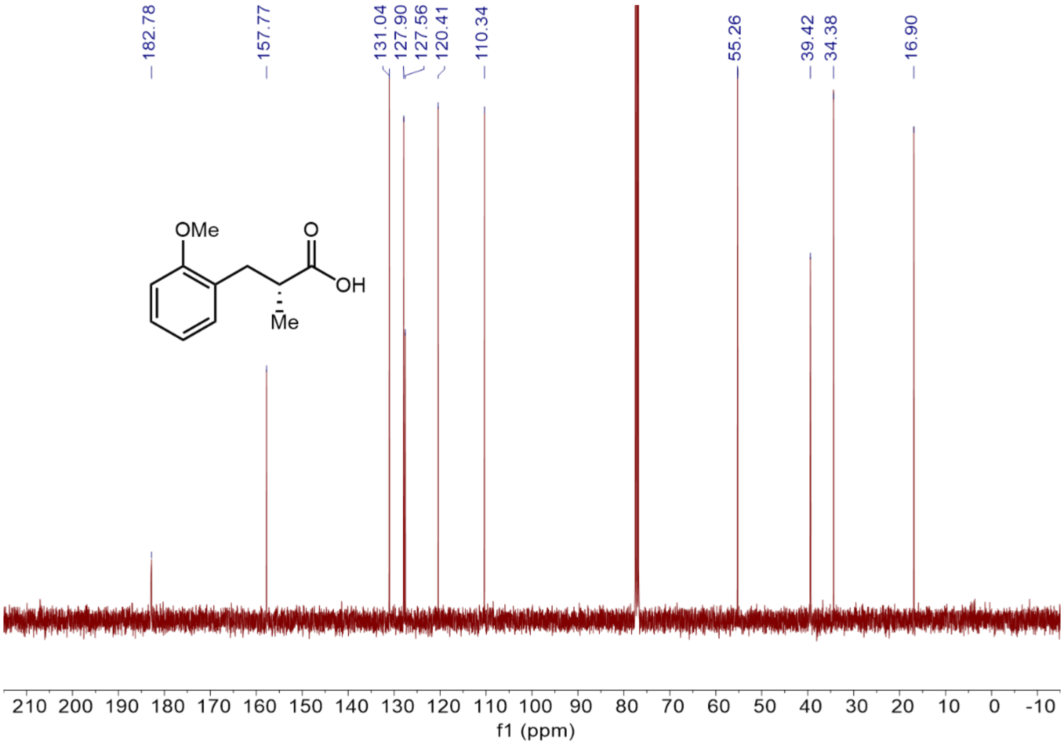

Supplementary Figure 31. NMR spectra of (*R*)-3-(2-methoxyphenyl)-2-methylpropanoic acid (3f)

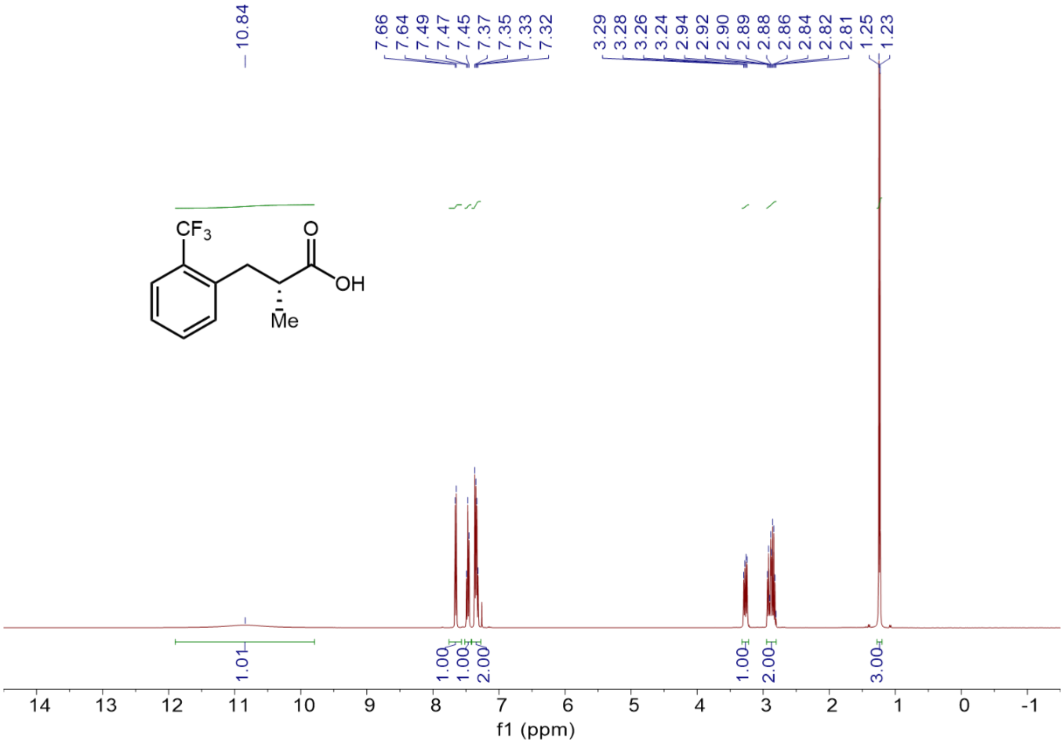
10.84
7.66
7.64
7.49
7.47
7.45
7.37
7.35
7.33
7.32
3.29
3.28
3.26
3.24
2.94
2.92
2.90
2.89
2.88
2.86
2.84
2.82
2.81
1.25
1.23
CF3
O
OH
Me
1.01
1.00
1.00
2.00
1.00
2.00
3.00
14 13 12 11 10 9 8 7 6 5 4 3 2 1 0 -1
f1 (ppm)

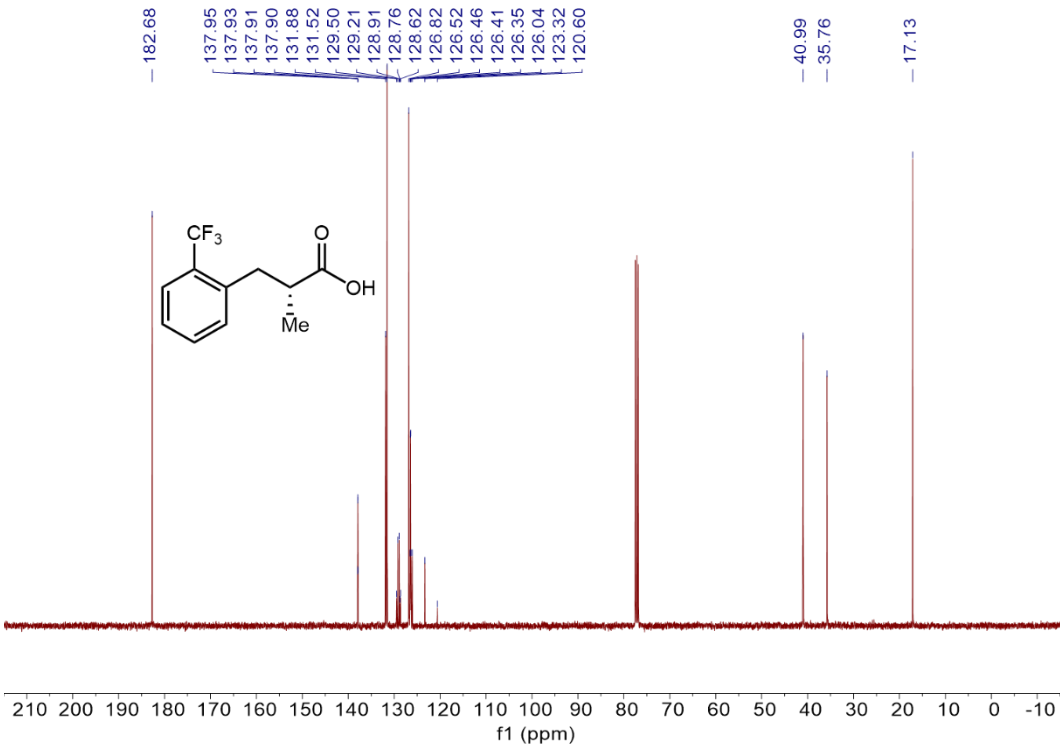
182.68
137.95
137.93
137.91
137.90
131.88
131.52
129.50
129.21
128.91
128.76
128.62
126.82
126.52
126.46
126.41
126.35
126.04
123.32
120.60
40.99
35.76
17.13
CF3
O
OH
Me
210 200 190 180 170 160 150 140 130 120 110 100 90 80 70 60 50 40 30 20 10 0 -10
f1 (ppm)

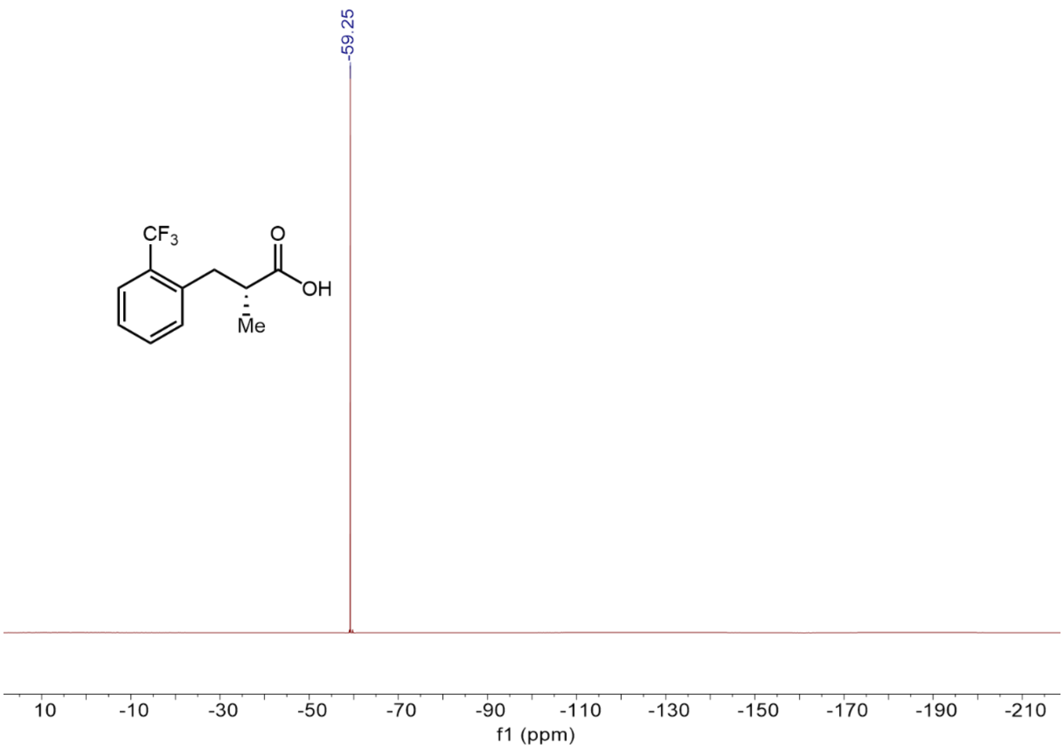

Supplementary Figure 32. NMR spectra of (*R*)-2-methyl-3-(2-(trifluoromethyl)phenyl)propanoic acid (3g)

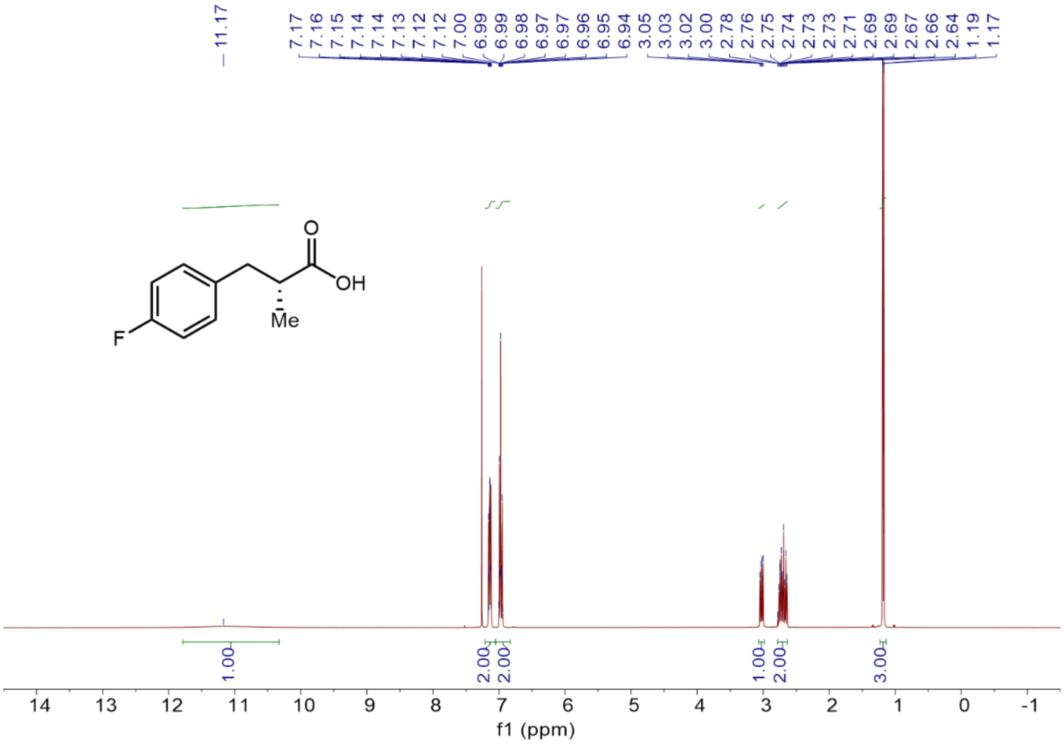
11.17
7.17 7.16 7.15 7.14 7.14 7.13 7.12 7.12 7.00 6.99 6.99 6.98 6.97 6.97 6.96 6.95 6.94 3.05 3.03 3.02 3.00 2.78 2.76 2.75 2.74 2.73 2.73 2.71 2.69 2.69 2.67 2.66 2.64 1.19 1.17
O
OH
Me
F
1.00
2.00
2.00
1.00
2.00
3.00
14 13 12 11 10 9 8 7 6 5 4 3 2 1 0 -1
f1 (ppm)

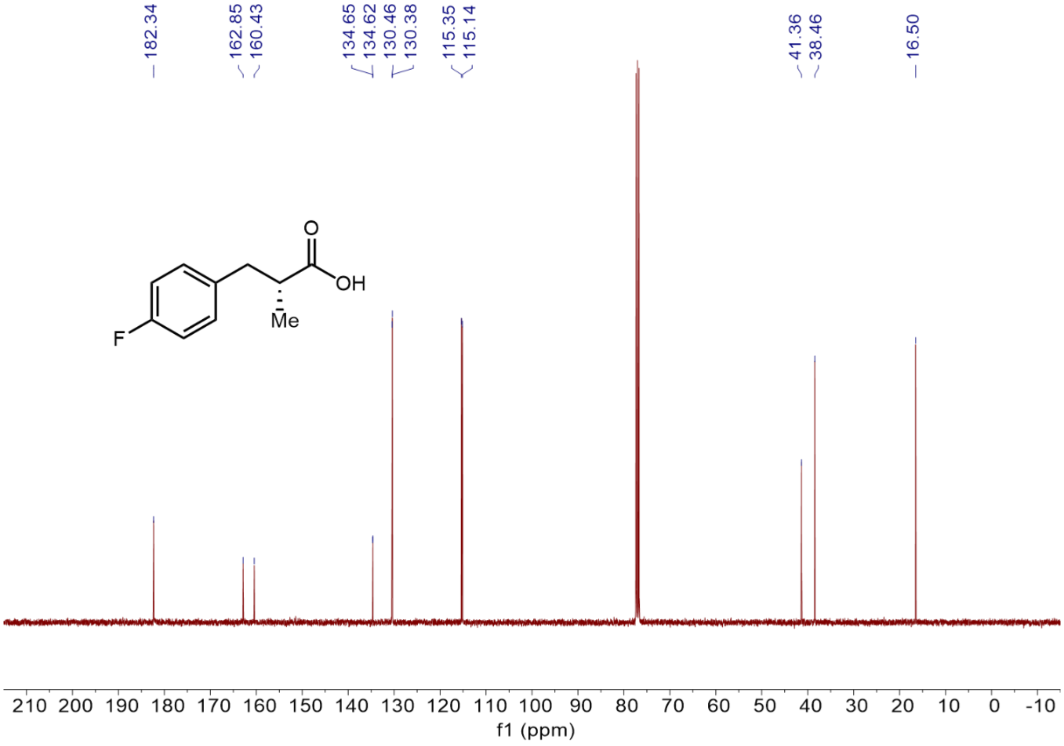
182.34
162.85 160.43
134.65 134.62 130.46 130.38
115.35 115.14
41.36 38.46
16.50
O
OH
Me
F
210 200 190 180 170 160 150 140 130 120 110 100 90 80 70 60 50 40 30 20 10 0 -10
f1 (ppm)

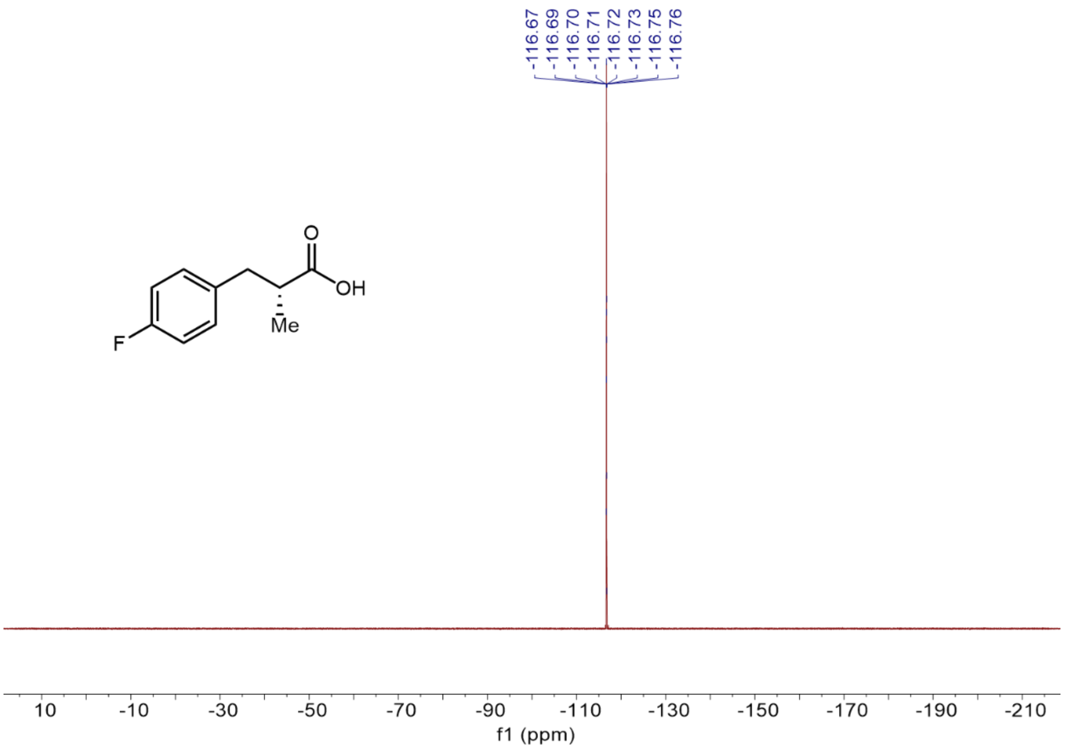

Supplementary Figure 33. NMR spectra of (*R*)-3-(4-fluorophenyl)-2-methylpropanoic acid (3h)

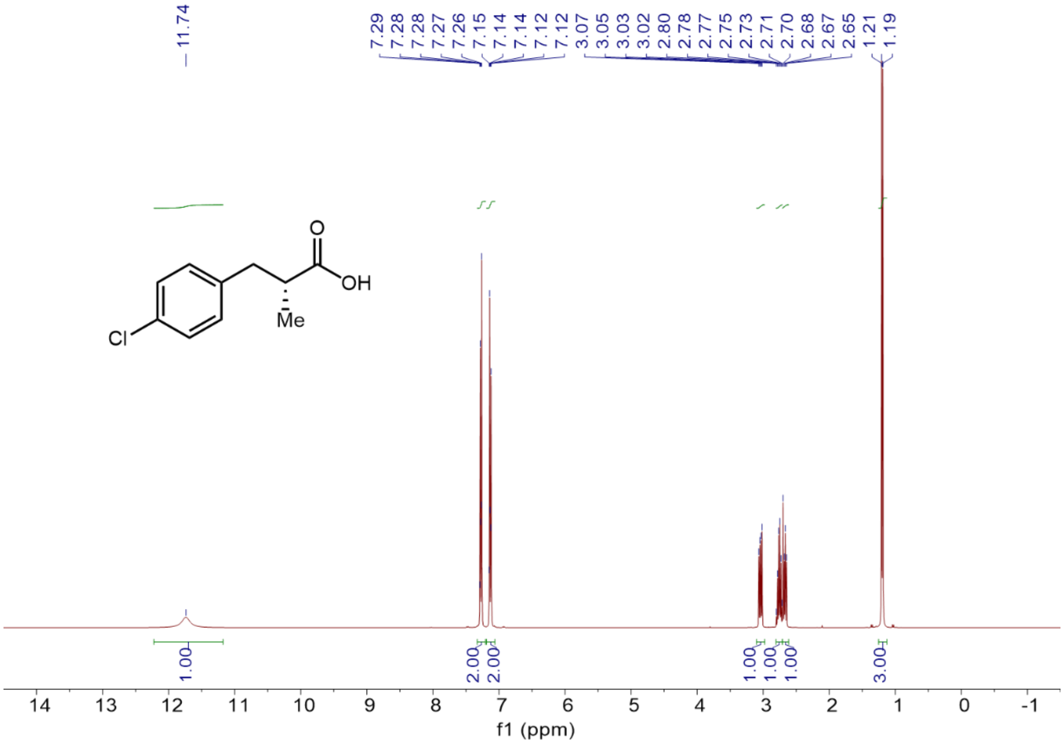

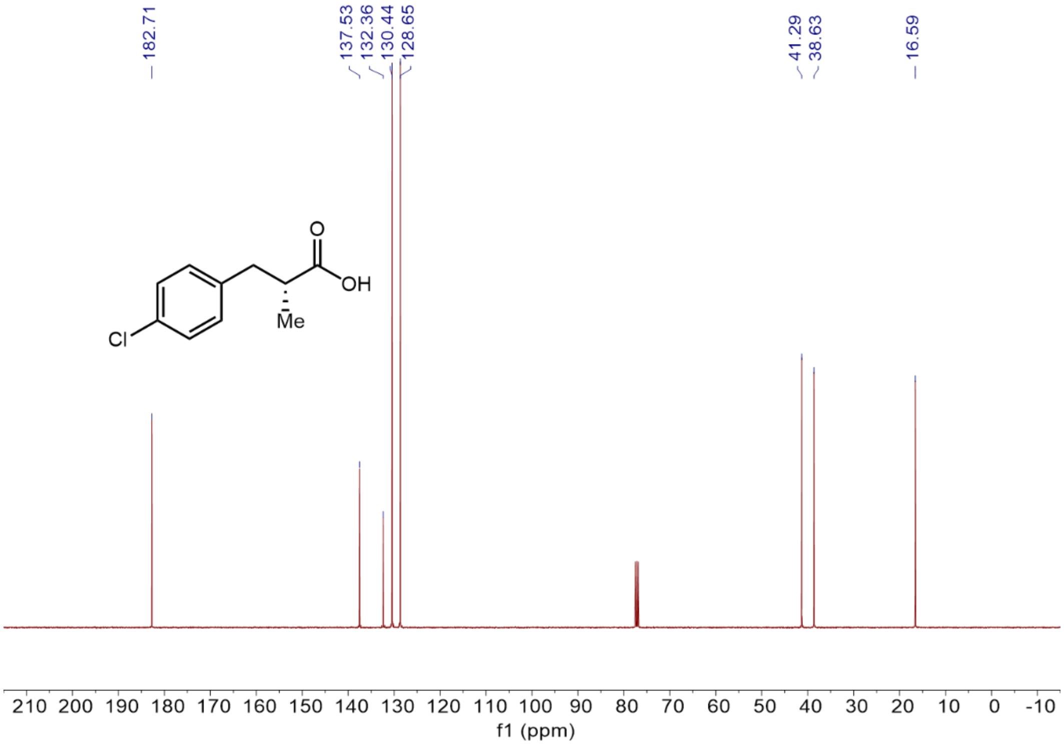

Supplementary Figure 34. NMR spectra of (*R*)- 3-(4-chlorophenyl)-2-methylpropanoic acid (3i)

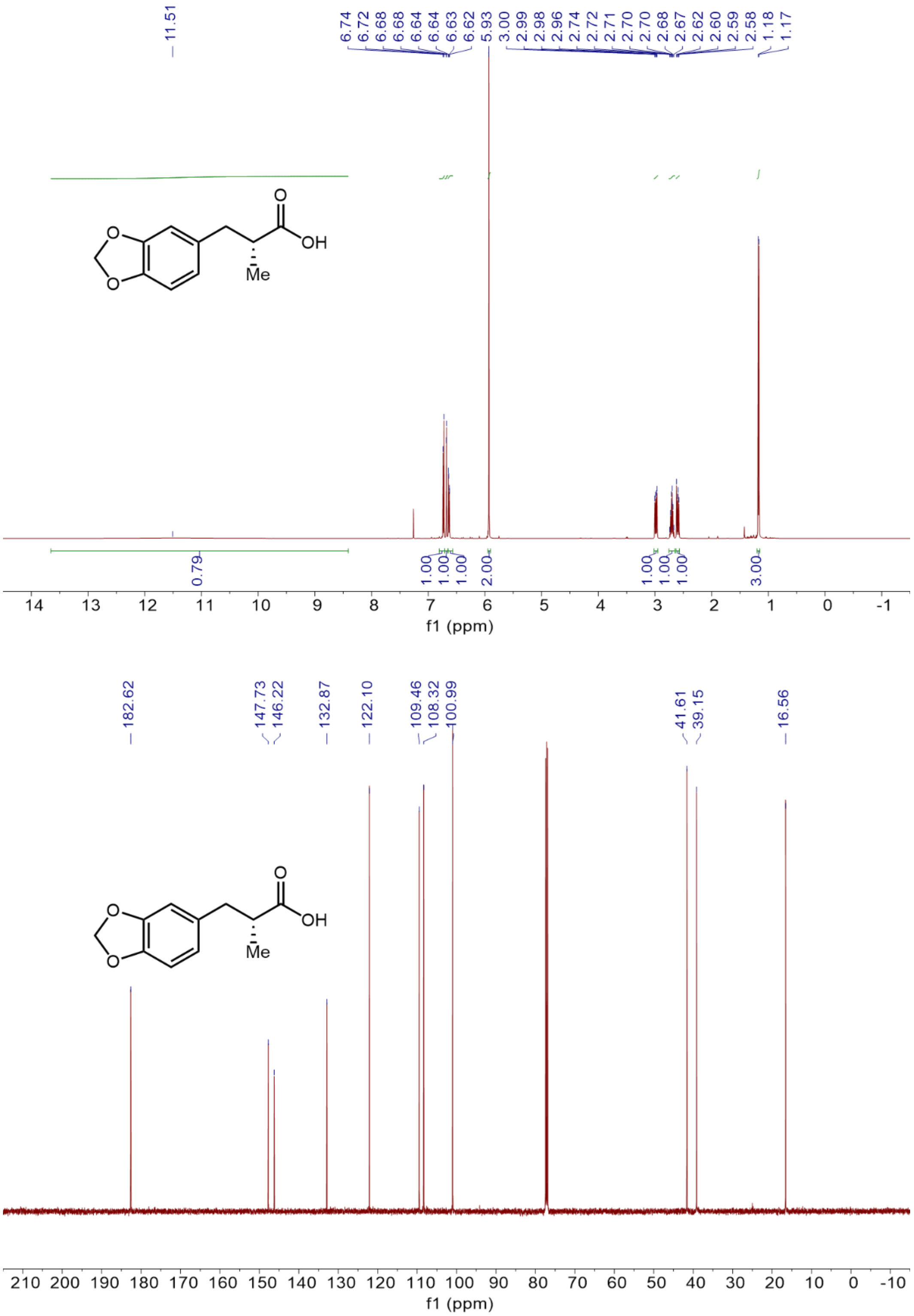

Supplementary Figure 35. NMR spectra of (*R*)-3-(benzo[*d*][1,3]dioxol-5-yl)-2-methylpropanoic acid (3j)

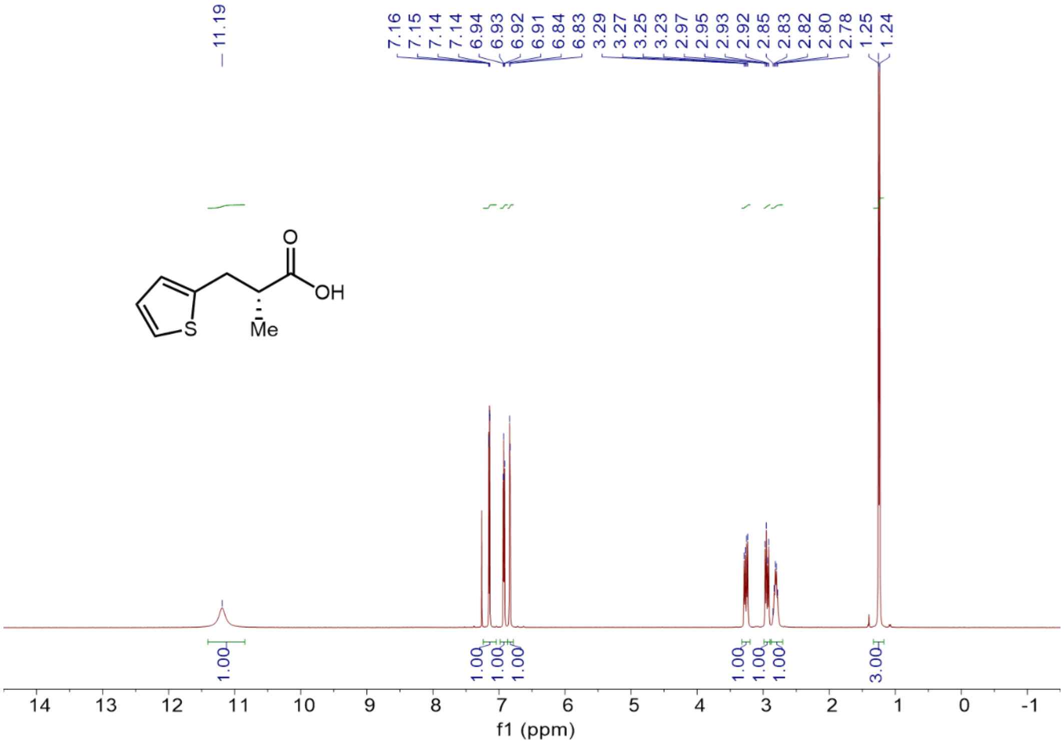

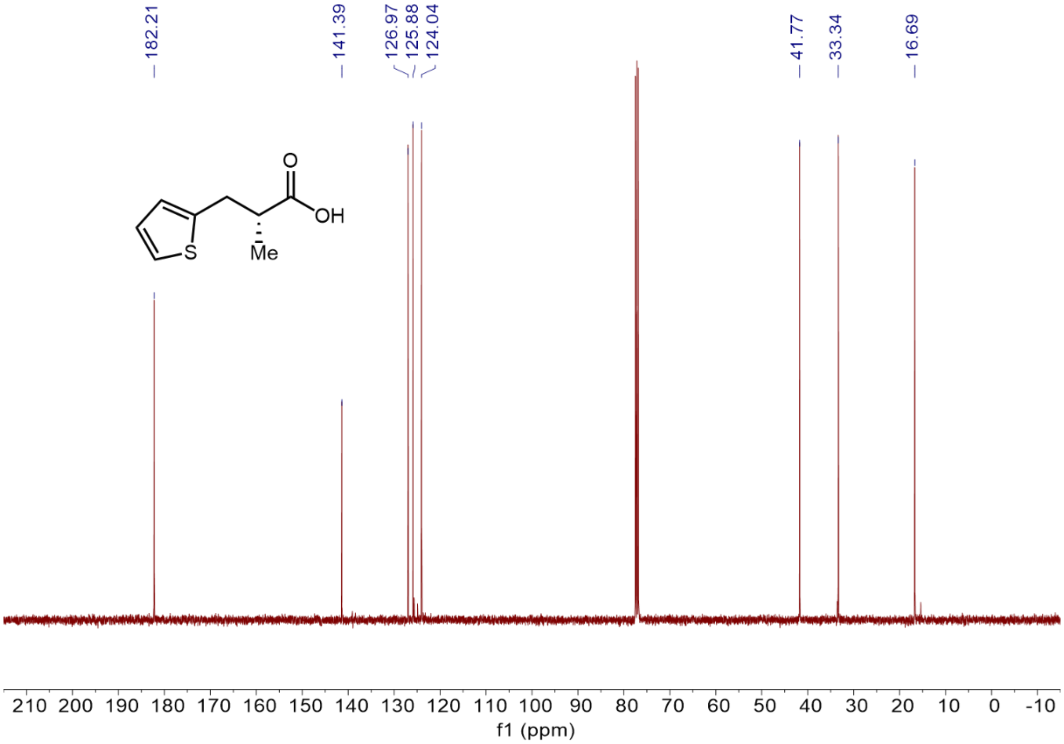

Supplementary Figure 36. NMR spectra of (*R*)-2-methyl-3-(thiophen-2-yl)propanoic acid (3k)

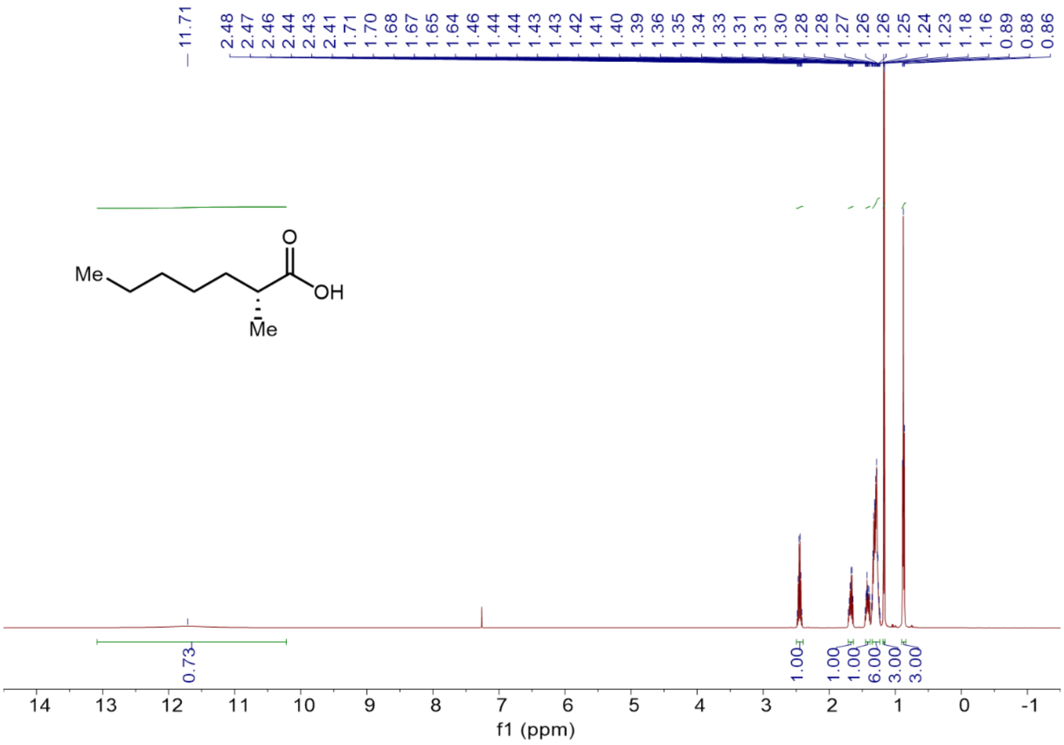

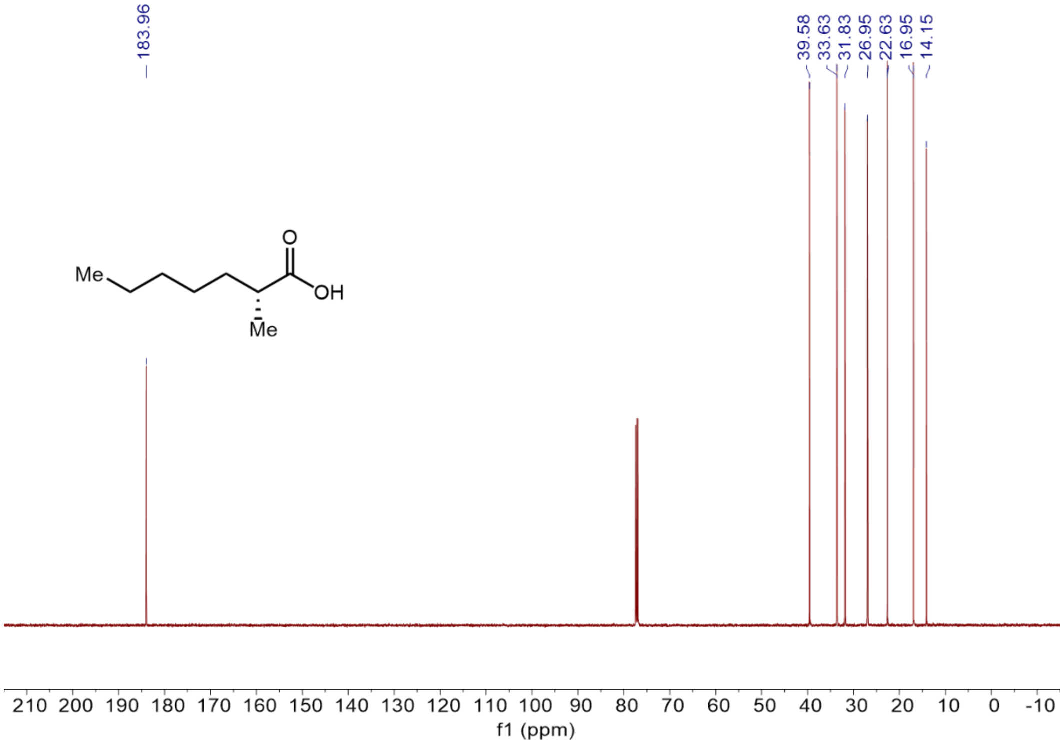

Supplementary Figure 37. NMR spectra of (*R*)-2-methylheptanoic acid (3l)

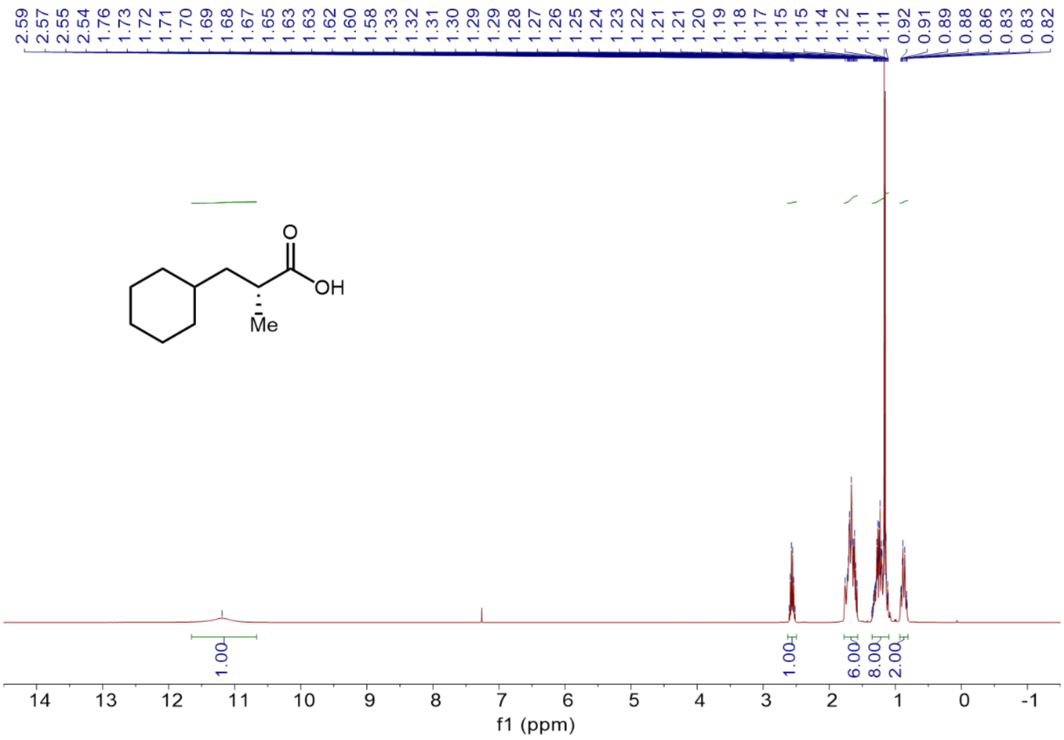

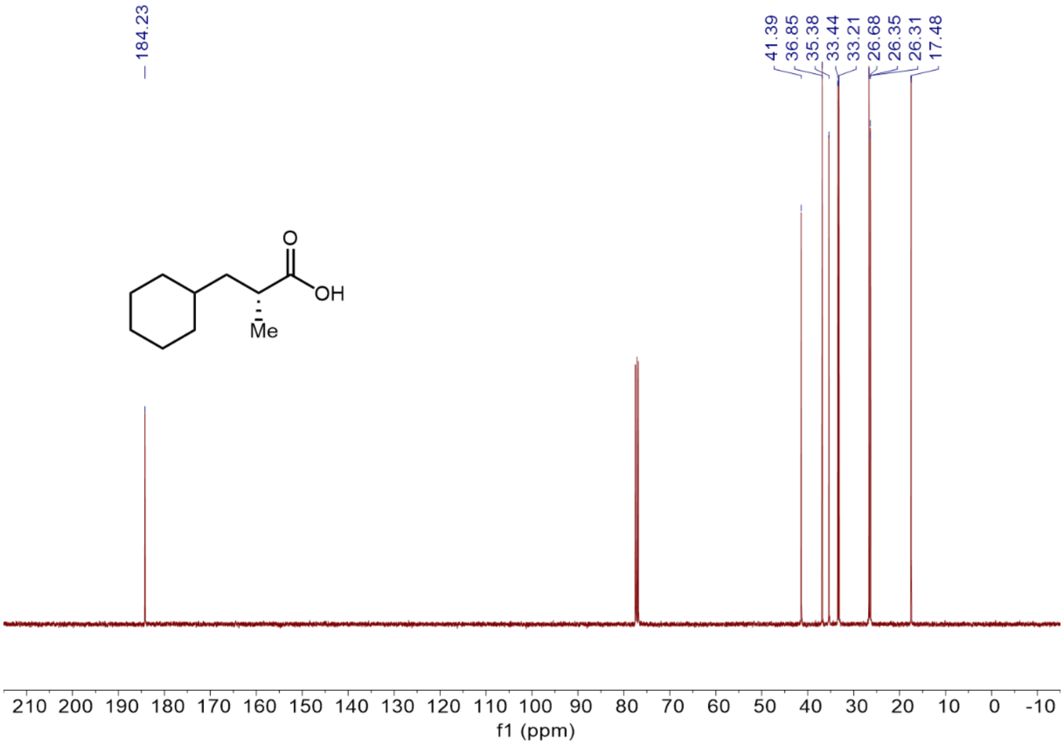

Supplementary Figure 38. NMR spectra of (*R*)-3-cyclohexyl-2-methylpropanoic acid (3m)

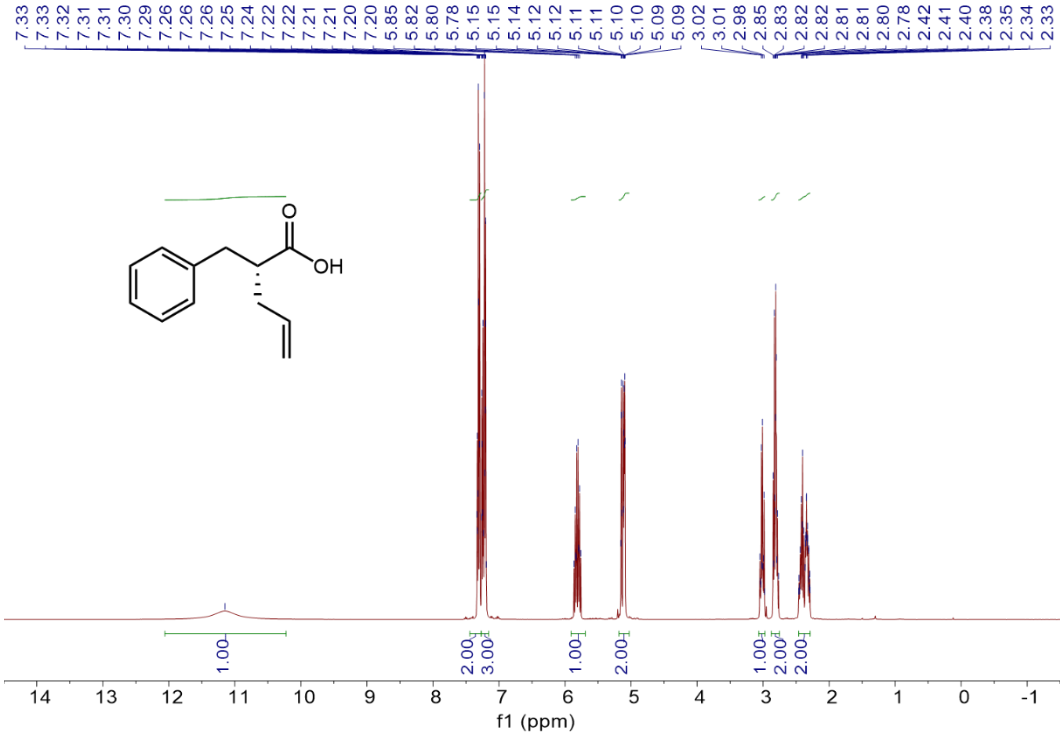

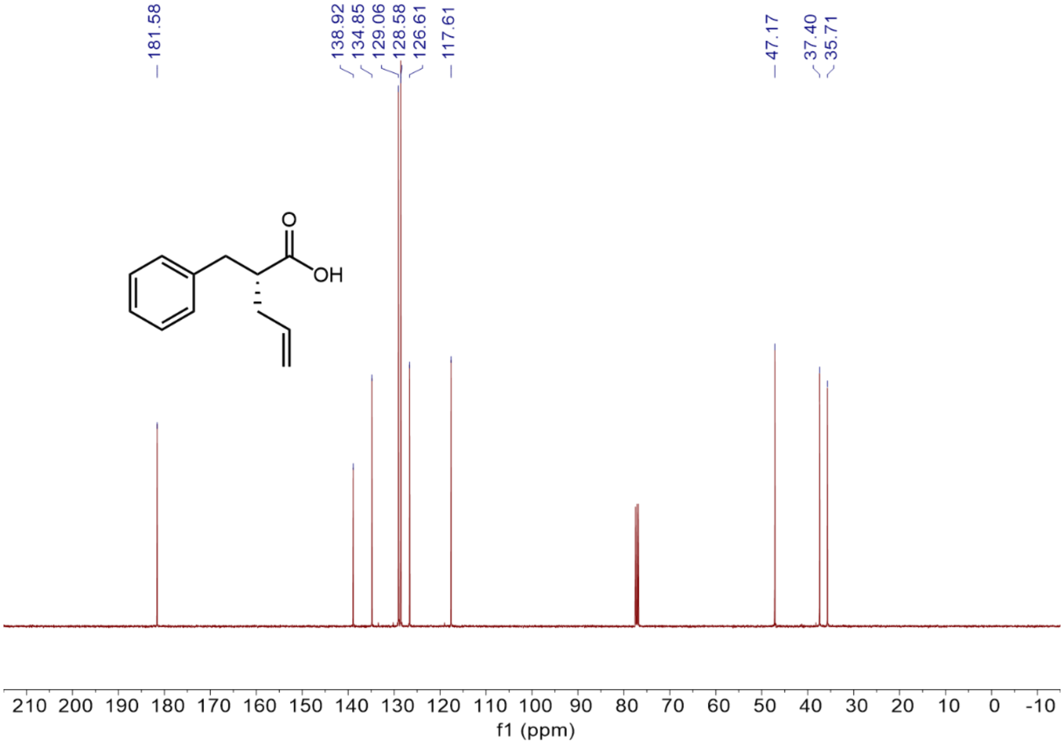

Supplementary Figure 39. NMR spectra of (*R*)-2-benzylpent-4-enoic acid (3n)

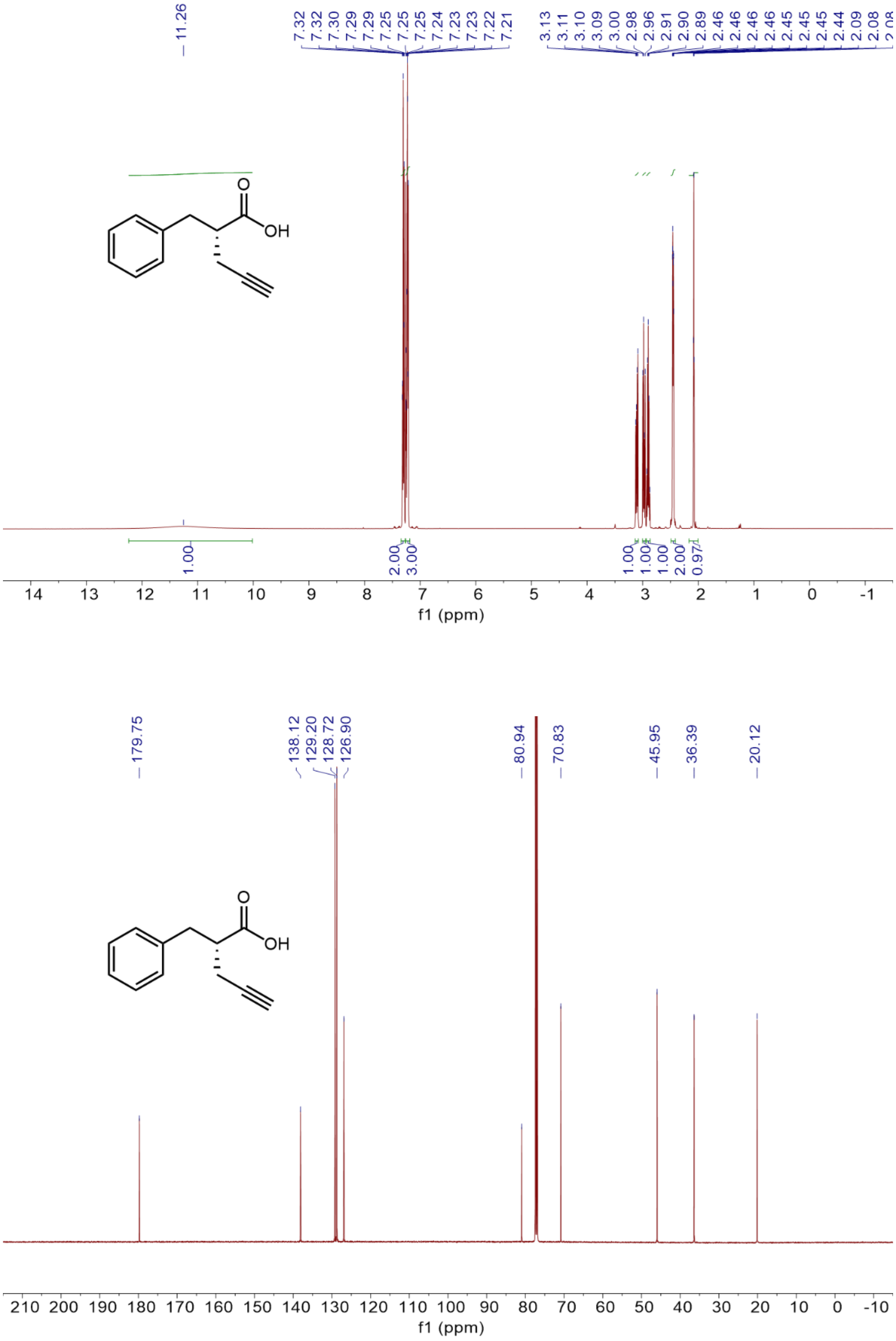

Supplementary Figure 40. NMR spectra of (*R*)-2-benzylpent-4-ynoic acid (3o)

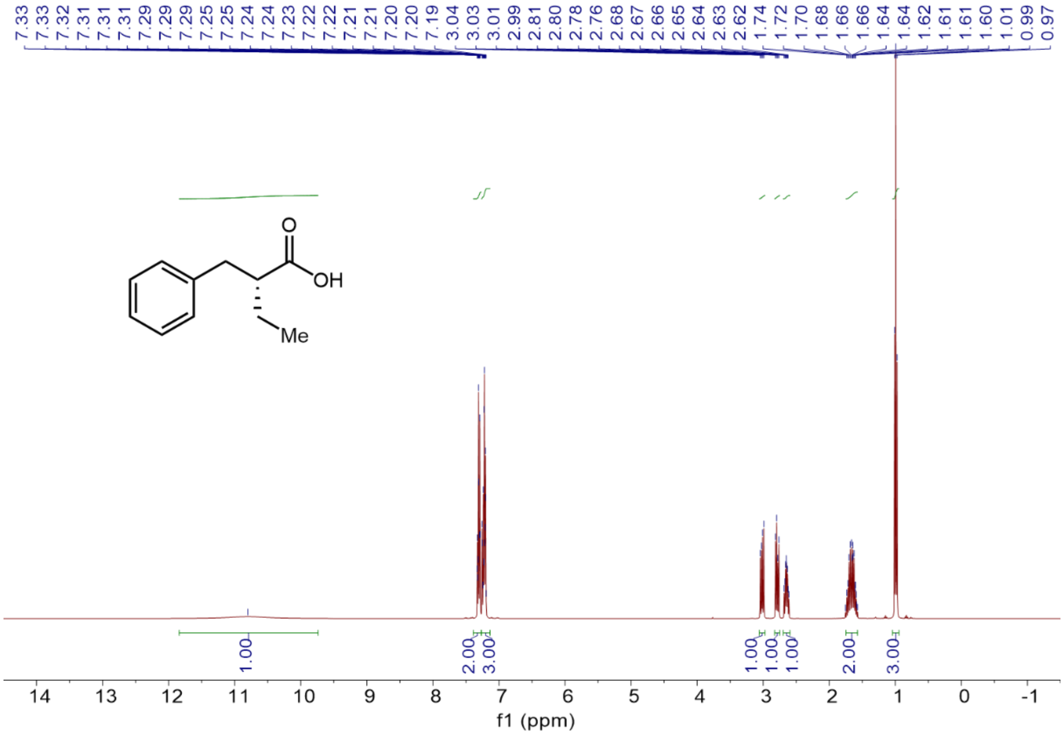

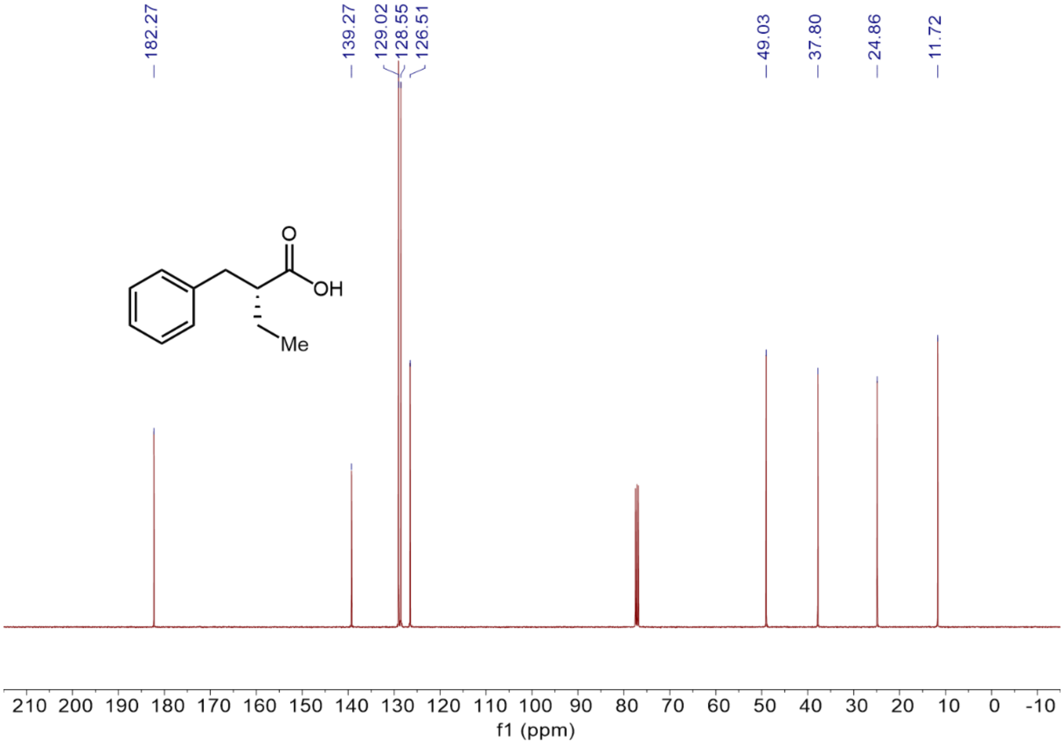

Supplementary Figure 41. NMR spectra of (*R*)-2-benzylbutanoic acid (3p)

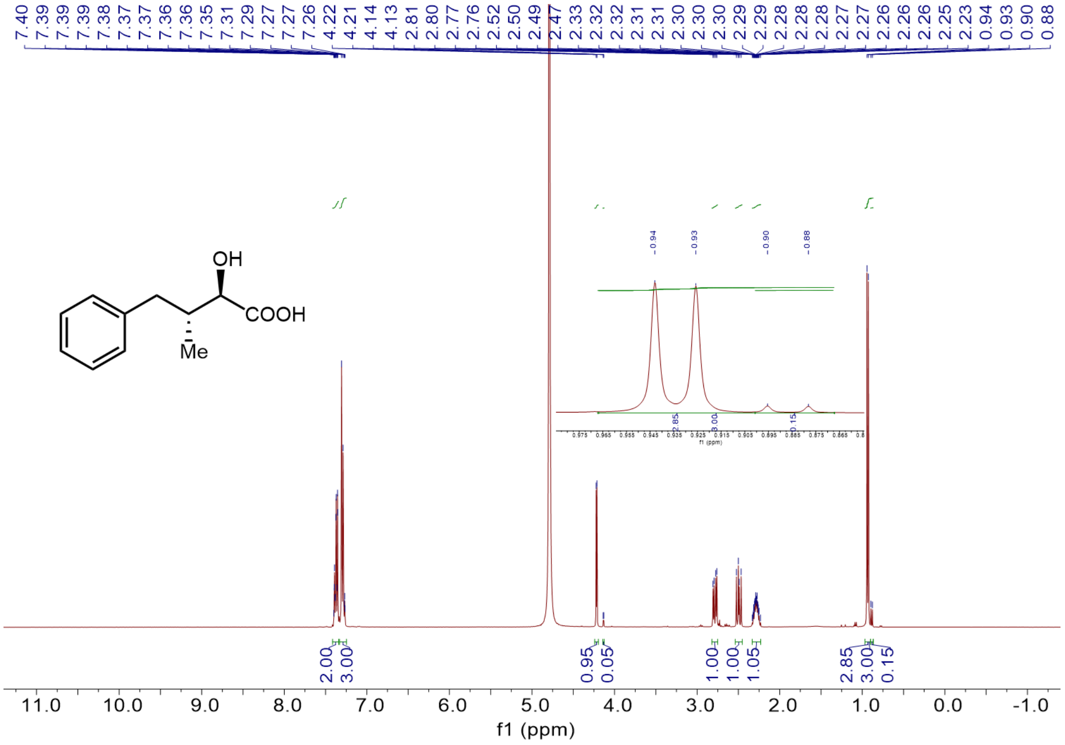

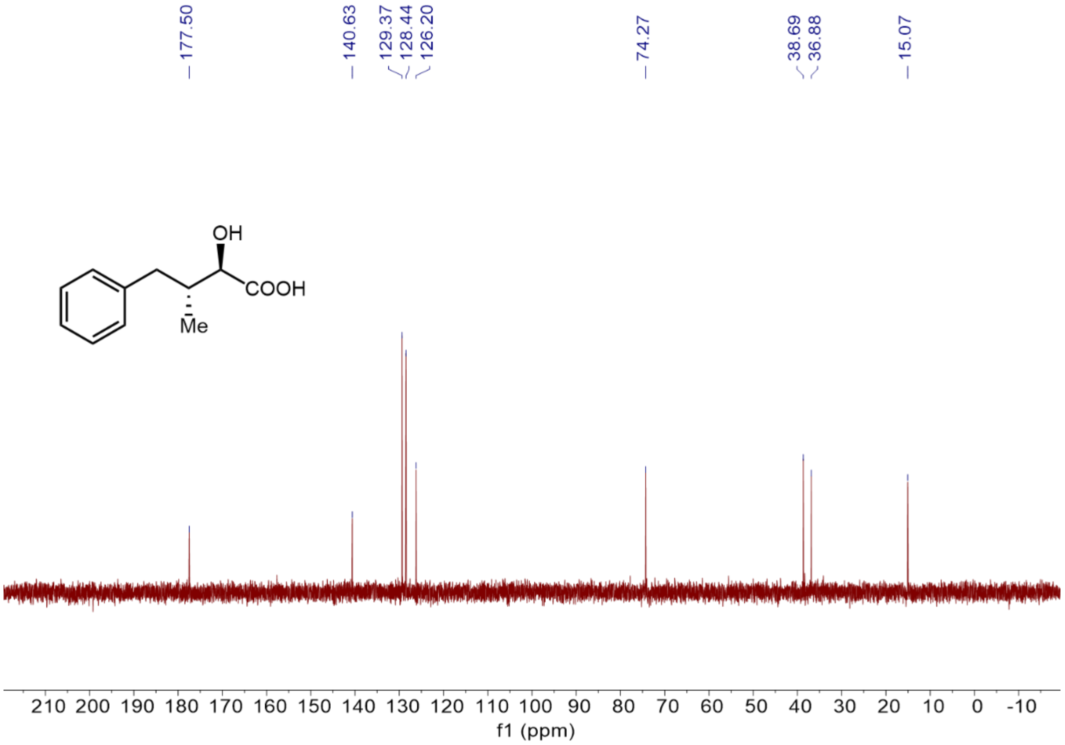

Supplementary Figure 42. NMR spectra of (2*R*,3*R*)-2-hydroxy-3-methyl-4-phenylbutanoic acid (4a)

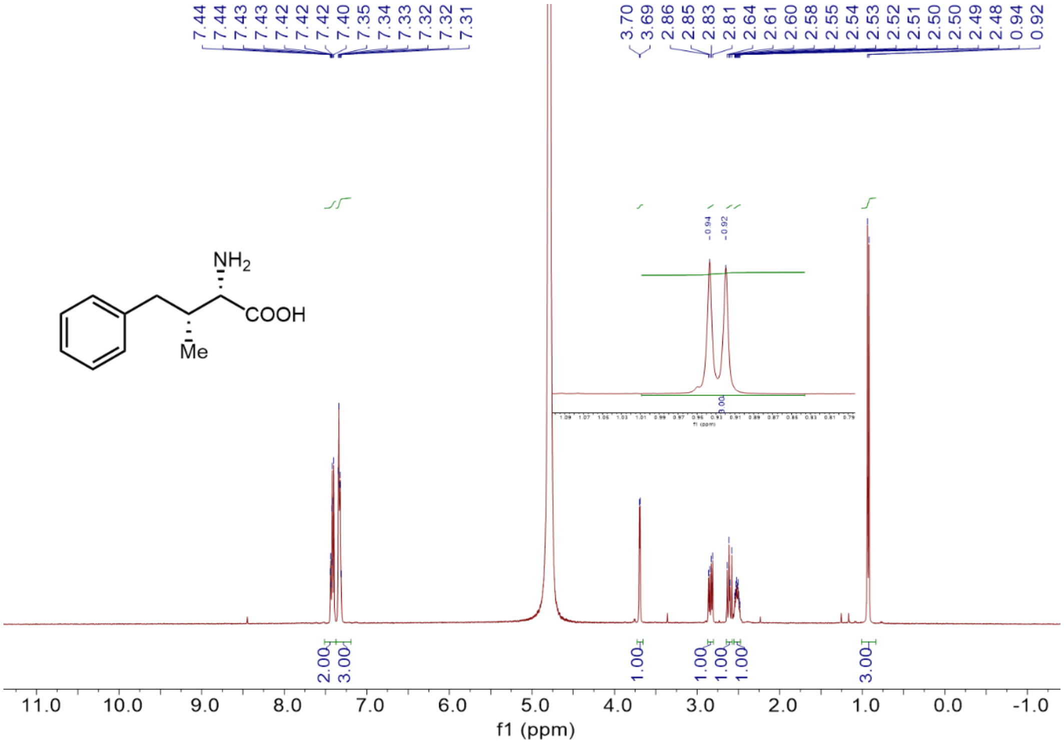

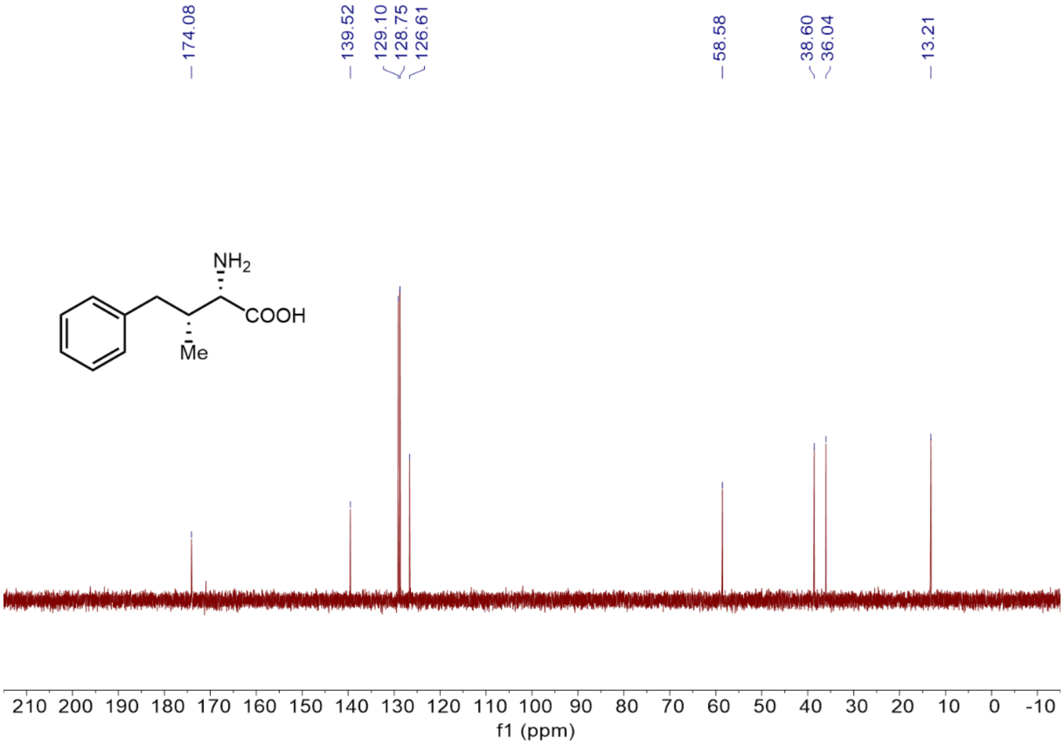

Supplementary Figure 43. NMR spectra of (2*S*,3*R*)-2-amino-3-methyl-4-phenylbutanoic acid (4b)

XIV. References

1. Liao, C. & Seebeck, F.P. *S*-adenosylhomocysteine as a methyl transfer catalyst in biocatalytic methylation reactions. *Nat. Catal.* **2**, 696-701 (2019).
2. Li, Q., Sun, B., Chen, J., Zhang, Y., Jiang, Y. & Yang, S. A modified pCas/pTargetF system for CRISPR-Cas9-assisted genome editing in *Escherichia coli*. *Acta Biochim. Biophys. Sin.* **53**, 620-627 (2021).
3. Gibson, D. G., Young, L., Chuang, R. Y., Venter, J. C., Hutchison III, C. A. & Smith, H. O. Enzymatic assembly of DNA molecules up to several hundred kilobases. *Nat. Methods.* **6**, 343-345 (2009).
4. Liao, C. & Seebeck, F.P. Asymmetric β-methylation of L- and D-α-amino acids by a self-contained enzyme cascade. *Angew. Chem. Int. Ed.* **59**, 7184-7187 (2020).
5. Tang, Q., Grathwol, C. W., Aslan-Üzel, A. S., Wu, S., Link, A., Pavlidis, I.V., Badenhorst, C.P.S. & Bornscheuer, U. T. Directed evolution of a halide methyltransferase enables biocatalytic synthesis of diverse SAM analogs. *Angew. Chem. Int. Ed.* **60**, 1524-1527 (2021).
6. Bengel, L. L., Aberle, B., Egler-Kemmerer, A. N., Kienzle, S., Hauer, B. & Hammer, S. C. Engineered enzymes enable selective *N*-alkylation of pyrazoles with simple haloalkanes. *Angew. Chem. Int. Ed.* **60**, 5554-5560 (2021).
7. Laskowski, R.A. & Swindells, M.B. LigPlot+: multiple ligand-protein interaction diagrams for drug discovery. *J. Chem. Inf. Model.* **51**, 2778-2786 (2011).
8. Dippe, M., Brandt, W., Rost, H., Porzel, A., Schmidt, J. & Wessjohann, L. A. Rationally engineered variants of *S*-adenosylmethionine (SAM) synthase: reduced product inhibition and synthesis of artificial cofactor homologues. *Chem. Commun.* **51**, 3637-3640 (2015).
9. Zhu, L., Chen, H., Meng, Q., Fan, W., Xie, X. & Zhang, Z.. Highly enantioselective hydrogenation of 2-oxo-4-arybutanoic acids to 2-hydroxy-4-arylbutanoic acids. *Tetrahedron* **67**, 6186-6190 (2011).
10. Bhushan, B., Lin, Y. A., Bak, M., Phanumartwiwath, A., Yang, N., Bilyard, M. K., Tanaka, T., Hudson, K.L., Lercher, L., Stegmann, M., Mohammed,S. & Davis, B. G. Genetic incorporation of olefin cross-metathesis reaction tags for protein modification. *J. Am. Chem. Soc.* **140**, 14599-14603 (2018).

11. Furukawa, K., Inada, H., Shibuya, M. & Yamamoto, Y. Chemoselective conversion from α-hydroxy acids to α-keto acids enabled by nitroxyl-radical-catalyzed aerobic oxidation. *Org. Lett.* **18**, 4230-4233 (2016).

12. Singh, S., Zhang, J., Huber, T. D., Sunkara, M., Hurley, K., Goff, R. D., Sunkara, M., Hurley, K., Goff, R.D., Wang, G., Zhang, W., Liu, C., Rohr, J., Van Lanen, S.G., Morris, A.J. & Thorson, J. S. Facile chemoenzymatic strategies for the synthesis and utilization of *S*-adenosyl-L-methionine analogues. *Angew. Chem. Int. Ed.* **53**, 3965-3969 (2014).

13. Zhang, Z., Han, Y., Zhan, B., Wang, S. & Shi, B. Synthesis of bicyclo [*n*.1.0] alkanes by a cobalt-catalyzed multiple C(sp^3)-H activation strategy. *Angew. Chem. Int. Ed.* **56**, 13145-13149 (2017).

14. Yang, P., Sun, Y., Fu, K., Zhang, L., Yang, G., Yue, J., Ma, Y., Zhou, J. & Tang, B. Enantioselective synthesis of chiral carboxylic acids from alkynes and formic acid by nickel-catalyzed cascade reactions: facile synthesis of profens. *Angew. Chem. Int. Ed.* **61**, e202111778 (2022).

15. Kimura, J., Takada, Y., Inayoshi, T., Nakao, Y., Goetz, G., Yoshida, W. Y. & Scheuer, P. J. Kulokekahilide-1, a cytotoxic depsipeptide from the cephalaspidean mollusk Philinopsis speciosa. *J. Org. Chem.* **67**, 1760-1767 (2002).

16. Vonrhein, C., Flensburg, C., Keller, P., Sharff, A., Smart, O., Paciorek, W., Womack, T. & Bricogne, G. Data processing and analysis with the autoPROC toolbox. *Acta Crystallogr. D* **67**, 293-302 (2011).

17. Terwilliger, T.C., Adams, P.D., Read, R.J., McCoy, A.J., Moriarty, N.W., Grosse-Kunstleve, R.W., Afonine, P.V., Zwart, P.H. & Hung, L.W. Decision-making in structure solution using Bayesian estimates of map quality: the PHENIX AutoSol wizard. *Acta Crystallogr. D* **65**, 582-601 (2009).

18. Skubák, P., Araç, D., Bowler, M.W., Correia, A.R., Hoelz, A., Larsen, S., Leonard, G.A., McCarthy, A.A., McSweeney, S., Mueller-Dieckmann, C. & Otten, H. A new MR-SAD algorithm for the automatic building of protein models from low-resolution X-ray data and a poor starting model. *IUCrJ* **5**, 166-171 (2018).

19. Cowtan K. The Buccaneer software for automated model building. 1. Tracing protein chains. *Acta Crystallogr. D* **62**, 1002-1011 (2006).

20. Emsley P., Lohkamp B., Scott W.G. & Cowtan K. Features and development of Coot. *Acta. Crystallogr. D* **66**, 486-501 (2010).

21. Murshudov, G.N., Skubák, P., Lebedev, A.A., Pannu, N.S., Steiner, R.A., Nicholls, R.A., Winn, M.D., Long, F. & Vagin, A.A. REFMAC5 for the refinement of macromolecular crystal structures. *Acta. Crystallogr. D* **67**, 355-367 (2011).

22. Brünger AT. [19] Free R value: Cross-validation in crystallography. In: *Methods in Enzymology* (Vol. 277, pp. 366-396). Academic Press (1997).
